# Intramolecular Cobalt Porphyrin-Catalyzed Alkylation of 1-Isoindolinones by Site-Selective Insertion into a C(sp^3^)–H Bond

**DOI:** 10.1021/acs.orglett.4c02270

**Published:** 2024-08-27

**Authors:** Christoph Buchelt, Julian Zuber, Thorsten Bach

**Affiliations:** Technische Universität München, TUM School of Natural Sciences, Department Chemie and Catalysis Research Center (CRC), 85747 Garching, Germany

## Abstract

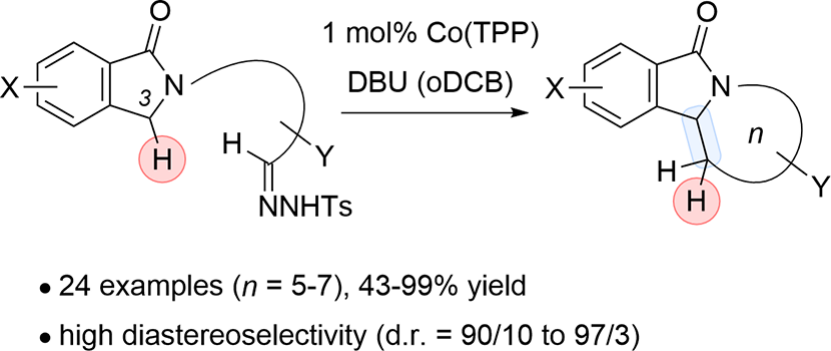

1-Isoindolinones with a reactive hydrazone tether attached to the nitrogen atom underwent an intramolecular alkylation in the presence of cobalt(tetraphenylporphyrin) and a base. Products display saturated heterocyclic rings of various sizes (*n* = 5–7), and the method was applied to a short synthesis of the azepane alkaloid lennoxamine. The reaction likely involves a diazoalkane intermediate that undergoes dediazotation and a formal insertion into the C3–H bond. If a stereogenic center is present in the tether, a high degree of diastereoselectivity is recorded.

Conventional synthetic wisdom suggests to close the ring of saturated heterocyclic compounds by formation of a carbon–heteroatom bond.^1^ Large ring lactams and lactones, for example, are typically synthesized by macrocyclization strategies aiming at the formation of the C–N or C–O bond.^2^ With the advent of new reactions, which allow for an efficient, catalytic formation of C–C bonds, alternative routes have emerged, and there is an increasing number of macrolactams and macrolactones being prepared by intramolecular C–C bond formation.^3^ By the same token, cyclic ethers and amines are not necessarily formed any longer by ring closure of a carbon–heteroatom bond. Alternative strategies have received attention, among which the insertion into an existing C–H bond is particularly straightforward and concise.^4^ There is no need for prefunctionalization as the C–C bond is formed directly by an attack from a low-valent carbon intermediate.

On the basis of the interest of our group in the synthesis of pharmaceutically relevant heterocycles by C–H bond activation reactions,^5^ we have now explored synthetic access to tricyclic isoindolone derivatives^6^ of general structure **1** (Scheme 1). There are a large number of compounds with this structural element, many of which have been synthesized in the context of new lead identification campaigns. We envisaged position C3 of 1-isoindolinones to be suited for an intramolecular C–H insertion reaction ideally catalyzed by a transition metal M via a carbene type intermediate. A survey of the literature on the topic showed there is no precedent for this strategy.^7^ Typical transformations aiming at C–C bond formation at this position employ the weak C–H acidity at C3 to generate a carbanion that undergoes consecutive substitution or conjugate addition reactions.^8^

**Scheme 1:**
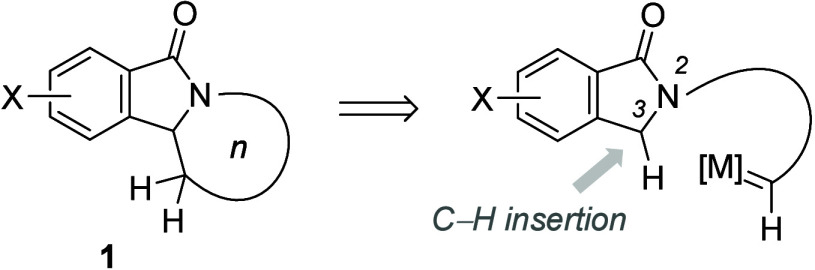
Possible Synthetic Route to Tricyclic Isoindolone Derivatives **1** from 1-Isoindolinones by Intramolecular C–H Insertion into the C3–H Bond

We envisioned diazoalkanes to be suitable precursors for the required carbene intermediates. They are typically formed by base-mediated 1,1-elimination from *N*-toluenesulfonyl (Ts)-substituted hydrazones.^9^ Accordingly, the preparation of the starting materials for this study commenced in most cases with condensation of a 2-benzofuran-1(3*H*)-one (phthalide) and an ω-functionalized amine.^10^ The terminal ω-carbon atom in the side chain linked to 1-isoindolinone atom N2 was then further manipulated. A protected aldehyde, for example, was deproteced, or an alcohol oxidized. Condensation with Ts hydrazide (TsNHNH_2_) completed the synthesis (for details, see the Supporting Information).

Optimization studies toward the desired cyclization were performed with hydrazone **2a**, which was obtained from parent phthalide in three steps (28% yield). With regard to the choice of metal catalyst, we were inspired by seminal contributions of the Zhang and de Bruin groups, who had successfully used cobalt(II) porphyrin complexes as catalysts to facilitate C–H insertion reactions.^11,12^ We consequently started to screen possible conditions with 5,10,15,20-tetraphenyl-21*H*,23*H*-porphincobalt(II) [Co(TPP)] as the catalyst (Table 1).

**Table 1:**
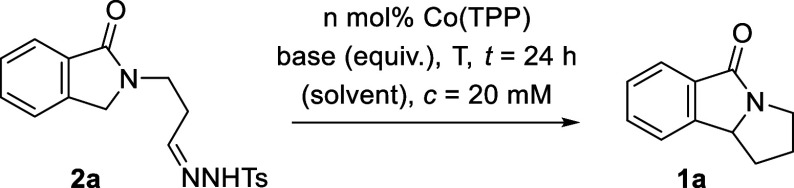
Optimization of the Reaction Parameters for the Alkylation of 1-Isoindolinone **1a**^a^

	mol %^b^	base	equiv	*T* (°C)	solvent	yield (%)^c^
1	5.0	Cs_2_CO_3_	1.5	40	PhMe	–
2	5.0	Cs_2_CO_3_	1.5	60	PhMe	41
3	5.0	Cs_2_CO_3_	1.5	60	PhCl	59
4	5.0	Cs_2_CO_3_	1.5	60	oDCB	65
5	5.0	Cs_2_CO_3_	2.0	60	oDCB	71
6	5.0	Cs_2_CO_3_	2.5	60	oDCB	73
7	5.0	Cs_2_CO_3_	5.0	60	oDCB	71
8	2.5	Cs_2_CO_3_	2.5	60	oDCB	58
9	1.0	Cs_2_CO_3_	2.5	60	oDCB	78
10	–	Cs_2_CO_3_	2.5	60	oDCB	–
11	1.0	DBU	2.5	60	oDCB	83

a The reactions were performed on a 0.10 mmol scale employing 5,10,15,20-tetraphenyl-21*H*,23*H*-porphincobalt(II) as the catalyst. oDCB = *o*-dichlorobenzene. DBU = 1,8-diazabicyclo[5.4.0]undec-7-ene.

b Amount of catalyst.

c Yield of the isolated product.

Cesium carbonate was used as the ancillary base,^13^ and toluene as the solvent. Because the projected reaction was intramolecular, the influence of the concentration was considered marginal. The chosen concentration (*c*) of 20 mM allowed the substrate to be soluble in most solvents (see the Supporting Information for a complete set of optimization data). The reaction was consistently run for 24 h, enabling a meaningful comparison of the reaction profiles. While no reaction was observed at 40 °C in toluene (entry 1), a notable conversion was achieved at 60 °C with 5 mol % catalyst (entry 2). Chlorinated benzenes as solvents resulted in a better yield, and *o*-dichlorobenzene was found to be the solvent of choice (entries 3 and 4). In all cases, the reaction remained incomplete and starting materials were detected. An increase in the amount of base had a beneficial influence on the yield, and the conversion was complete when 2.5 equiv of base was used. A further increase in the amount of base did not improve the performance (entries 5–7). The catalyst loading could be decreased to 1.0 mol % without compromising the yield (entries 8 and 9), and it was shown that the catalyst was required to achieve the desired transformation (entry 10). With the conditions of entry 6, other cobalt(II) tetraarylporphyrin complexes were tested but the influence of the aryl group was found to be insignificant [67–75% yield (see the Supporting Information)]. A screening of bases revealed DBU to perform slightly better than Cs_2_CO_3_ (entry 11).

The conditions of entry 11 were considered to be best suited for studying the scope of the intramolecular alkylation with various hydrazones **2** (Scheme 2). Five-membered ring formation was facile for almost any of the tested substrates, and high yields were recorded (77–95%). The tolerated functional groups include bromo (products **1c**, **1f**, **1h**, and **1m**), chloro (**1e**), fluoro (**1g**), cyano (**1j**), methoxycarbonyl (**1k**), methoxy (**1l**), and trifluoromethyl (**1n**) substituents. Only nitro-substituted substrate **2i** reacted sluggishly and gave a lower product yield (43%). The reaction to 9-bromo-1,2,3,9b-tetrahydro-5*H*-pyrrolo[2,1-*a*]isoindol-5-one (**1m**) was also performed on a millimole scale and required an elongated reaction time of 48 h. C–C bond formation at a C3-substituted isoindolinone was possible, as was the alkylation of an aza-1-isoindolinone (products **1o** and **1p**).

**Scheme 2:**
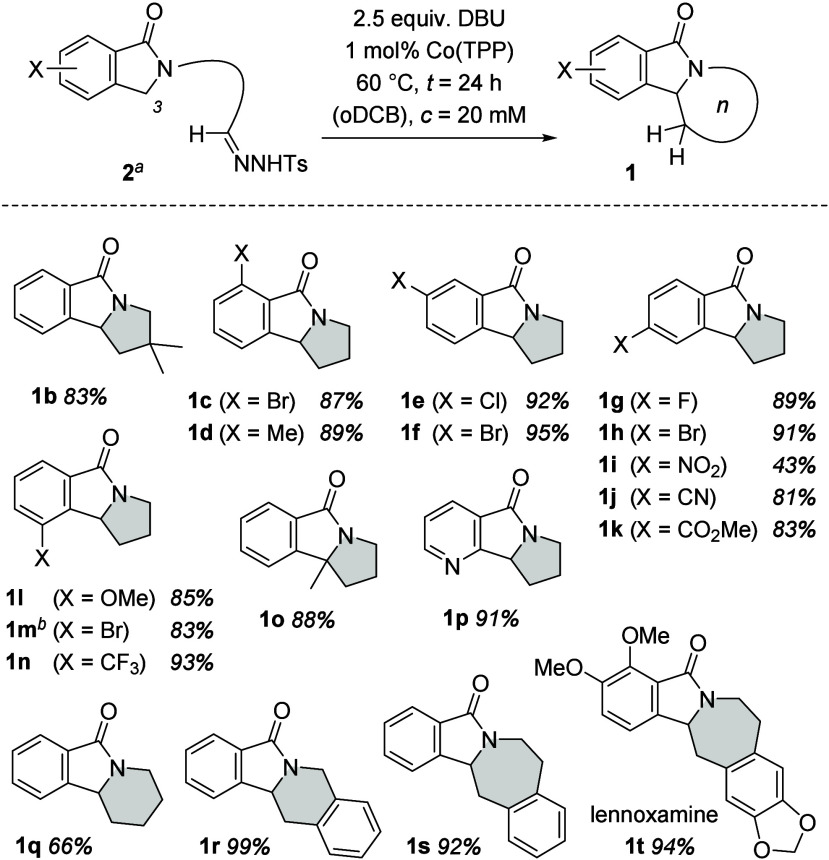
Intramolecular, Cobalt(II) Porphyrin-Catalyzed Alkylation of 1-Isoindolinones Starting from Hydrazones **2** as Precursors The reactions were performed on a 0.10 mmol scale employing 5,10,15,20-tetraphenyl-21*H*,23*H*-porphincobalt(II) as the catalyst. The reaction was also performed on a 1.00 mmol scale in 81% yield (*t* = 48 h; see the Supporting Information for details).

Encouraged by the results in the pyrrolo series, we attempted to apply the reaction conditions to the formation of six-membered rings, and 1,3,4,10b-tetrahydropyrido[2,1-*a*]isoindol-6(2*H*)-one (**1q**) was obtained in 66% yield. In contrast to products **1a**–**1p**, a small amount of a side product could be detected, which was identified as 2-(but-3-en-1-yl)isoindolin-1-one [**4q**, 10% (see Scheme 3)]. The observation is in agreement with previous studies of the formation of piperidines from hydrazones^11e^ and indicates a second competitive 1,5-hydrogen abstraction of the radical intermediate (*vide supra*). It was suggested that the pathway could be suppressed if no abstractable hydrogen atom was available at the α-position of the hydrazone. This hypothesis was verified with hydrazone **2r**, which was derived from a substituted benzaldehyde substrate and provided tetracyclic product **1r** in almost perfect yield (99%). The benzene ring within the tether likely rigidifies the chain and facilitates a smooth reaction. Along the same lines, the formation of a seven-membered ring, starting from a precursor with an aliphatic linker, was not productive. Once the rotational freedom was restricted by an *o*-benzene ring in the tether, the alkylation became feasible. The reactivity pattern was initially probed with an otherwise unsubstituted starting material **2s**, which led to a remarkable yield of 92% of the desired product **1s**. The success encountered with this reaction inspired an application of the method to the synthesis of the alkaloid lennoxamine (**1t**), an azepane alkaloid isolated from the Chilean barberry.^14,15^ Here, the respective substrate **2t** required both the benzene ring of the indolinone and the benzene ring within the tether to be substituted with oxygen. The Co-catalyzed alkylation proceeded smoothly and provided the natural product in 94% yield.

**Scheme 3:**
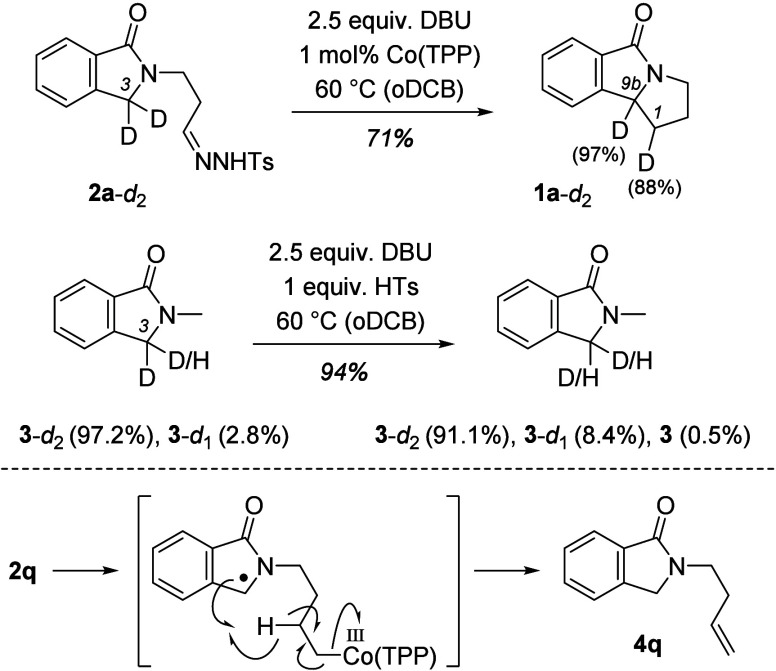
Experiments with Deuterated 1-Isoindolinones **2a**-*d*_2_ and **3**-*d*_2_ Indicating That Position C3 Is Susceptible to H/D Exchange and the Putative Formation of Side Product **4q**

Limitations of the method include the formation of smaller and larger rings. Four-membered ring formation was not successful, nor could aliphatic seven-membered ring formation be observed (*vide infra*). Hydrazones derived from ketones did not react. A practical advantage is the use of an *in situ* procedure for the generation of the hydrazones.^11e^ Starting directly from the aldehyde, treatment with *p*-toluenesulfonyl hydrazide generates hydrazone **2a***in situ* and enables a one-pot reaction to afford compound **1a**. Following this procedure, the desired alkylation product was isolated in 81% yield.

To shed some light on the course of the reaction, we studied a selection of deuterated 1-isoindolones (Scheme 3). Subjecting deuterated substrate **2a**-*d*_2_ to the optimized conditions afforded expected product **1a**-*d*_2_ in 71% yield. While the degree of deuteration at C9b remained unaffected (97%), the extent of incorporation of deuterium at C1 of the product was slightly diminished (88%). On the basis of literature precedent,^8,16^ we suspected that position C3 of **2a** would display a weak C–H acidity and, thus, be responsible for the loss of deuterium. In fact, when we subjected N-methylated substrate **3**-*d*_2_ to the standard reaction conditions, we observed a slight decrease in the deuterium content. Although the latter observation made the accurate determination of a primary kinetic isotope effect impossible, we could clearly show for monodeuterated substrate **2a**-*d*_1_ that the C–H bond is significantly more reactive in the alkylation reaction than the C–D bond (see the Supporting Information).

Deuterium incorporation combined with the previous observation of hydrogen abstraction product **4q** agrees with the reaction course established for cobalt porphyrin-catalyzed alkylation reactions^11^ (Scheme 4). Accordingly, we assume that the base is responsible for the generation of diazoalkane **5** by 1,1-elimination. The latter expels a nitrogen molecule (N_2_) upon coordination to cobalt, and ensuing radical **6** initiates intramolecular hydrogen abstraction at the C3–H bond of the 1-isoindolinone. Radical **7** releases the cobalt fragment by homolytic ring closure but can also abstract a hydrogen atom in the α-position to the Co-substituted carbon atom. The side reaction manifests itself by leading to olefinic byproducts such as **4q** (*vide infra*).

**Scheme 4:**
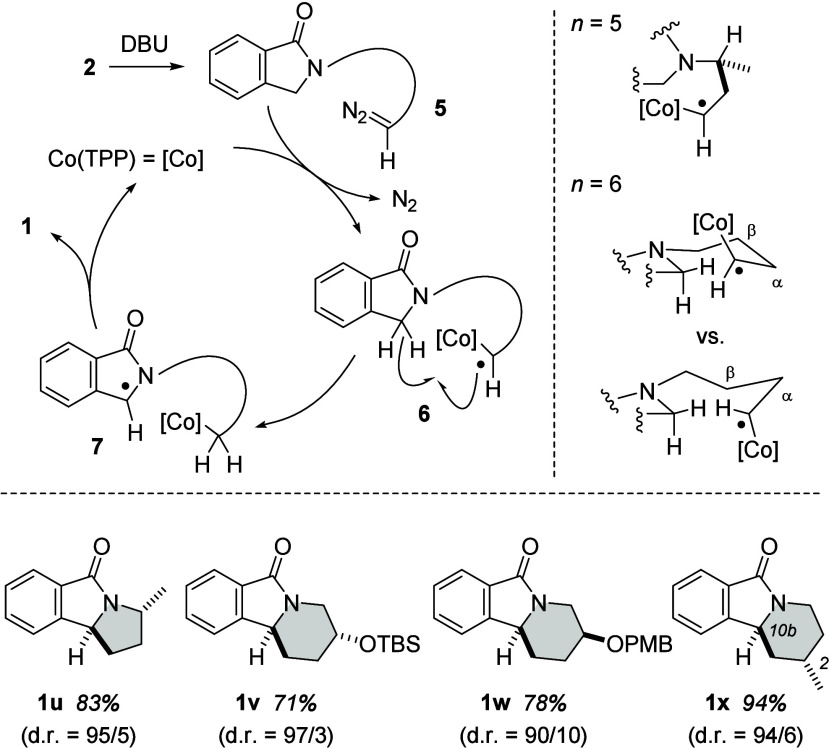
Mechanistic Proposal for the Intramolecular Alkylation, Possible Conformations of Intermediates, and Diastereoselective Reactions

The hydrogen abstraction/ring closure event is responsible for any diastereoselectivity observed in the alkylation reactions. We probed the outcome of the reaction with a selection of substrates displaying a stereogenic center in the tether. Configuration assignments were based on nuclear Overhauser exchange spectroscopy (NOESY) experiments. In the pyrrolo series, a stereogenic center adjacent to the nitrogen atom induced a high diastereoselectivity, and five-membered product **1u** was obtained in a diastereomeric ratio (dr) of 95/5. The diastereoselectivity can be explained by the conformation of the chain as enforced by 1,3-allylic strain^17^ (*n* = 5). For the closure of a piperidine ring (*n* = 6), we observed divergent diastereoselectivity depending on the substituent within the chain. With an oxygen-substituted stereogenic center in the β-position to the radical, formation of products **1v** and **1w** was observed. Product **1v** adopts a chair conformation, as one can also see in the crystal structures of related 1,3,4,10b-tetrahydropyrido[2,1-*a*]isoindol-6(2*H*)-ones.^18^ In the major diastereoisomer, the *tert*-butyldimethylsilyloxy (OTBS) group resides in an equatorial position, which supports the idea that hydrogen abstraction and cyclization had also occurred in a chairlike transition state. In contrast, major product **1v** of a related substrate with a *p*-methoxybenzyl (PMB)-protected oxygen atom adopts a boat-like conformation, possibly also in the transition state. The major diastereoisomer of product **1x** displayed a strong NOE contact between the C10b hydrogen atom and the methyl group residing in an axial position at C2. The relative configuration of this compound and its conformation in the solid state were corroborated by single-crystal X-ray crystallography (see the Supporting Information for details). Here, a 1,2-repulsion with the bulky [Co] fragment might enforce the axial position in a chair type transition state. In any case, the preliminary results indicate that a high degree of diastereoselectivity can be expected in the alkylation reaction and that the outcome might even be tunable by a judicious choice of protecting groups.

In summary, the cobalt porphyrin-catalyzed alkylation has been shown to be a useful and reliable method for the intramolecular alkylation of 1-isoindolinones at position C3. The starting materials are readily available, and the method is compatible with a wide array of functional groups. The differentiation of diastereotopic hydrogen atoms is possible if a stereogenic center is implemented in the reactive tether.

## Data Availability

The data underlying this study are available in the published article and its Supporting Information.

## Supporting Information Available

Materials and methods, optimization of reaction conditions, synthetic procedures and full characterization for all starting materials and products (**2a**–**2x** and **1a**–**1x**), spectroscopic data, NMR spectra, and X-ray crystallographic details (PDF)
FAIR data, including the primary NMR FID files, for compounds **2a–2x**, **2a**-*d*, **2a**-*d*_2_, **SI-1a**-*d*, **Si-1b**, **Si-1e**, **Si-1f**, **Si-1l**, **Si-1u**, **Si-1x**, **SI-2c**, **SI-2d**, **SI-2g**, **SI-2h**, **SI-2j**, **SI-2m**, **SI-2s**, **SI-3a**, **SI-3i**, **SI-3k**, **SI-3n–SI-3p**, **SI-4r**, **SI-4s**, **SI-6**, **SI-7a**, **SI-7b**, **SI-9**, **SI-11**, **SI-13**, **SI-15**, **1a–1x**, **1a**-*d*_1_, **1a**-*d*_2_, and **3**-*d*_2_ (ZIP)
