# Supplementary material for: Intramolecular Cobalt Porphyrin-Catalyzed Alkylation of 1-Isoindolinones by Site-Selective Insertion into a C(sp3)–H Bond
Source: Org Lett. 2024 Aug 27;26(35):7302–6. doi: 10.1021/acs.orglett.4c02270 (PMC11385374; doi:10.1021/acs.orglett.4c02270)
Supplement: Supplementary file 1 — ol4c02270_si_001.pdf [file ol4c02270_si_001.pdf]

Supporting Information

Intramolecular Cobalt Porphyrin-catalyzed Alkylation of 1-Isoindolinones by Site-selective Insertion into a C(sp^3)–H Bond

Christoph Buchelt,[a] Julian Zuber,[a] and Thorsten Bach*[a]

[a]*Technical University Munich, School of Natural Sciences, Department of Chemistry and Catalysis Research Center, Lichtenbergstrasse 4, 85747 Garching, Germany.*

*** Corresponding Author**

Email: thorsten.bach@ch.tum.de

Table of Contents

1. General Remarks 3

2. Preparation of Hydrazones 5

3. Optimization of the reaction conditions with hydrazone 48

4. Catalysis 50

5. Deuteration Experiments 66

6. Application: Synthesis of Lennoxamine 73

7. Crystallographic Data 79

8. NMR Spectra of New Compounds 81

9. References 175

1. General Remarks

All experiments were performed in flame-dried glassware under argon atmosphere and under anhydrous conditions using *Schlenk* techniques unless otherwise stated. The heating of the reactions was carried out by means of an oil bath containing silicon oil.

Solvents and reagents: Dry dichloromethane (CH_2Cl_2) and tetrahydrofuran (THF) were obtained from an MBraun MB-SPS 800 solvent purification system. Dry benzene dimethylformamide (DMF), dimethylsulfoxide (DMSO), ethanol (EtOH), o-dichlorobenzene (*o*DCB) and methanol (MeOH) were obtained from either Sigma-Aldrich or Acros in the highest available purity (>99%, extra dry over molecular sieves) and used without further purification. Technical solvents used for aqueous workup and purification by column chromatography [acetone (ac), dichloromethane (CH_2Cl_2), ethyl acetate (EtOAc), hexane, methanol (MeOH)] were distilled prior to use. Commercially available chemicals were obtained from AlfaAesar, ABCR, BLDPharm, Sigma Aldrich and TCI Europe and used as received unless otherwise stated.

Catalyst synthesis: The employed Cobalt(II) porphyrin catalysts were synthesized according to literature procedures or purchased from Sigma Aldrich.[1] All analytical data were in agreement with the reported data.

Chromatography: Thin layer chromatography (TLC) was performed on pre-coated glass-backed Merck Kieselgel 60 F254 plates with visualization effected with ultra-violet irradiation (λ = 254, 366 nm) and/or staining using potassium permanganate (KMnO4) solution prepared from potassium permanganate (3.00 g), potassium carbonate (20.0 g) and 5% aqueous sodium hydroxide solution (5.00 mL) in water (300 mL). Flash column chromatography was performed on silica 60 (Merck, 230–400 mesh) with the indicated eluent mixtures. Automated flash column chromatography was performed on a Büchi C-815 Flash chromatography instrument for purification of all products unless otherwise noted. In all cases, Biotage® pre-packed silica cartridges (Biotage® Sfär Silica D Duo 60 µm 10 g, manufacturer number FSRD-0445-0050, FSRD-0445-0025, FSRD-0445-0010, FSRD-0445-0005) were used in combination UV detection at 265, 280 and 320 nm.

NMR spectroscopy: ^{1}H NMR spectra were recorded on Bruker AVHD-300, AVHD-400 or AVHD-500 spectrometers at 303 K operating at 300 MHz, 400 MHz and 500 MHz, respectively. Data is reported in the following manner: chemical shift [in parts per million (ppm) relative to residual $CHCl_3$ (δ_H = 7.26 ppm) or DMSO-d_5 (δ_H = 2.50 ppm)], number of protons, multiplicity and coupling constant *J* (measured in Hz to the nearest 0.1 Hz). The multiplicity of a signal is indicated as: s-singlet, bs-broad singlet, d-doublet, t-triplet, q-quartet, quint-quintet, m-multiplet, or combinations of these. Apparent multiplets which occur because of coupling constant equality between magnetically non-equivalent protons are marked as virtual (*virt*.). ^{13}C NMR spectra were recorded on a AVHD-400 or AVHD-500 spectrometers at 303 K operating at 101 MHz and 126 MHz respectively with proton decoupling. The chemical shift [in parts per million (ppm)] is reported relative to residual $CHCl_3$ (δ_C = 77.16 ppm) or DMSO-d_5 (δ_C = 39.52 ppm). ^{19}F NMR spectra were recorded on a Bruker AVHD-400 or AVHD-500 spectrometers at 303 K operating at 376 MHz and 476 MHz and are given without reference. Spectra are reported based on appearance, not on theoretical multiplicities derived from structural information.

Mass Spectroscopy (ESI): High-resolution mass spectra (HRMS) were recorded on a Q Exactive Plus from *Thermo Fisher Scientific* (HRMS-ESI) with an orbitrap mass analyzer. Each value obtained was within 7 ppm of the calculated mass.

Melting points (m.p.): Determined using a Kofler heating bar designed by L. Kofler (Reichert) without correction, with range quoted to the nearest whole number

Infrared spectroscopy (IR): Spectra were recorded on a Perkin Elmer Frontier Optica+SP10 spectrometer by ATR technique. The signal intensity is assigned using the following abbreviations: vs (very strong), s (strong), m (medium), w (weak).

2. Preparation of Hydrazones

2.1. General procedure 1 (GP 1): Synthesis of Isoindolinones by Condensation of Phthalide with Aminoalcohols

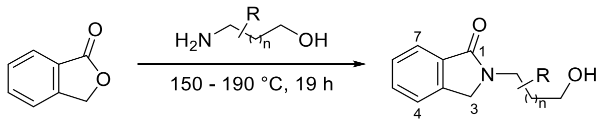

Based on a modified procedure by *Norman et al.*,[2] phthalide (1.34 g, 10.0 mmol, 1.00 equiv.) and the corresponding aminoalcohols (11.0 mmol, 1.10 equiv.) were combined in a flask a heated to 150 °C for 4 h and afterwards to 190 °C for 15 h. The reaction mixture was cooled to room temperature and dissolved in CH_2Cl_2 (100 mL). The organic layer was washed with H_2O (100 mL), dried over Na_2SO_4, filtered and the solvent was removed under reduced pressure. The crude products were either used without further purification of subjected to automated flash column chromatography (EtOAc/MeOH = 9/1) to yield the entitled free alcohol.

*2.1.1. 2-(3-Hydroxypropyl)isoindolin-1-one (**SI-1a**)*

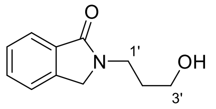

Following GP 1, compound **SI-1a** was obtained as a yellow oil (1.79 g, 9.35 mmol, 94%).

^{1}H NMR (500 MHz, $CDCl_3$): δ [ppm] = 1.80 – 1.86 (m, 2H, H-2’), 3.57 (t, ^3J = 5.6 Hz, 2H, H-1’), 3.78 (t, ^3J = 6.3 Hz, 2H, H-3’), 7.44 – 7-50 (m, 2H, H-, H-‘), 7.55 (*virt.* td, $^3J \approx {}^3J$ = 7.4 Hz, ^4J = 1.2 Hz, 1H, H-5), 7.84 (d, ^3J = 7.6 Hz, 1H, H-7).

^{13}C NMR (126 MHz, $CDCl_3$): δ [ppm] = 169.9 (C-1), 141.3 (C-3a), 132.3 (C-7a), 131.6 (C-4), 128.3 (C-6), 123.8 (C-7), 122.9 (C-4), 58.3 (C-1’), 50.5 (C-3), 38.8 (C-3’), 30.9 (C-2’).

Spectral data matched those reported in the literature.[2]

2.1.2. *2-(3-Hydroxy-2,2-dimethylpropyl)isoindolin-1-one (**SI-1b**)*

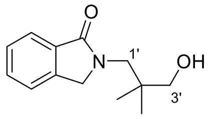

Following GP 1, compound **SI-1b** was obtained as a colorless solid (1.48 g, 6.73 mmol, 67%).

^{1}H NMR (500 MHz, $CDCl_3$): δ [ppm] = 1.01 [s, 6H, $(CH_3)_2$], 3.26 (s, 2H, H-3'), 3.46 (s, 2H, H-1'), 4.55 (s, 2H, H-3), 7.47 (*virt.* dt, ^3J = 7.5 Hz, $^4J \approx {^5J}$ = 1.0 Hz, 1H, H-4), 7.50 (*virt.* td, $^3J \approx {^3J}$ = 7.5 Hz, ^4J = 1.0 Hz, 1H, H-6), 7.56 (*virt.* td, $^3J \approx {^3J}$ = 7.5 Hz, ^4J = 1.2 Hz, 1H, H-5), 7.85 (d, ^3J = 7.5 Hz, 1H, H-7).

^{13}C NMR (126 MHz, $CDCl_3$): δ [ppm] = 171.0 (C-1), 141.6 (C-3a), 132.1 (C-7a), 131.9 (C-5), 128.4 (C-6), 123.9 (C-7), 122.7 (C-4), 68.2 (C-3'), 54.2 (C-3), 50.9 (C-1'), 38.5 (C-2'), 23.6 [$(CH_3)_2$].

HRMS (ESI) *m/z*: $[M+H]^+$ Calcd. for $C_{13}H_{18}NO_2$ 220.1332; Found 220.1334.

IR (ATR): $\tilde{\nu}$ [cm^{-1}] = 3318 (m, OH), 3050 (w), 2959 (w), 2931 (w), 2907 (w), 2855 (w), 1654 (vs, C=O), 1619 (m, C=C), 1471 (s), 1418 (s), 1306 (m), 1050 (vs), 879 (m), 734 (vs, sp^2 C–H), 685 (vs), 670 (s).

2.1.3. *2-(4-Hydroxybutyl)isoindolin-1-one (**SI-1q**)*

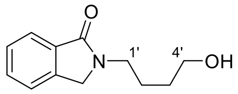

Following GP 1, compound **SI-1q** was obtained as a yellow oil (1.23 g, 5.98 mmol, 60%).

^{1}H NMR (500 MHz, $CDCl_3$): δ [ppm] = 1.58 – 1.66 (m, 2H, H-2'), 1.74 – 1.82 (m, 2H, H-3'), 2.28 (bs, 1H, OH), 3.64 – 3.74 (m, 4H, H-1', H-4'), 4.39 (s, 2H, H-3), 7.41 – 7.48 (m, 2H, H-4, H-6), 7.49 – 7.55 (m, 1H, H-5), 7.81 -7.85 (m, 1H, H-7).

^{13}C NMR (126 MHz, $CDCl_3$): δ [ppm] = 168.9 (C-1), 141.2 (C-3a), 133.0 (C-7a), 131.4 (C-5), 128.2 (C-6), 123.8 (C-7), 122.8 (C-4), 62.5 (C-4'), 50.0 (C-3), 42.2 (C-1), 29.6 (C-2'), 25.2 (C-3').

HRMS (ESI) *m/z*: $[M+H]^+$ Calcd. for $C_{12}H_{16}NO_2$ 206.1176; Found 206.1174.

IR (ATR): $\tilde{\nu}$ [cm^{-1}] = 3395 (m, OH), 3051 (w), 2930 (w), 2864 (w), 1663 (vs, C=O), 1619 (m, C=C), 1472 (s), 1456 (s), 1414 (s), 1303 (m), 1057 (m), 732 (vs, sp^2 C–H), 683 (s).

Spectral data matched those reported in the literature.[3]

*2.1.4. 2-(4-Hydroxybutan-2-yl)isoindolin-1-one (**SI-1u**)*

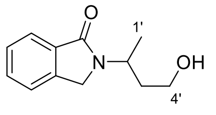

Following GP 1, compound **SI-1u** was obtained as a yellow oil (967 mg, 4.71 mmol, 47%).

***R*f** (EtOAc): 0.31 (UV, $KMnO_4$].

^{1}H NMR (500 MHz, $CDCl_3$): δ [ppm] = 1.34 (d, ^3J = 6.9 Hz, 3H, H-1'), 1.60 (*virt.* ddt, ^2J = 14.4 Hz, ^3J = 11.5 Hz, $^3J \approx {}^3J$ = 3.0 Hz, 1H, H^a-3',), 1.91 (dddd, ^2J = 14.4 Hz, ^3J = 11.2 Hz, ^3J =5.3 Hz, ^3J = 3.4 Hz, 1H, H^b-3'), 2,79 (bs, 1H, OH), 3.34 (ddd, ^2J = 12.1 Hz, ^3J = 11.2 Hz, ^3J = 3.0 Hz, 1H, H^a-4'), 3.59 (ddd, ^2J = 12.1 Hz, ^3J = 5.3 Hz, ^3J = 3.0 Hz, 1H, H^b-4'), 4.26 (d, ^2J = 17.1 Hz, 1H, H^a-3), 4.39 (d, ^2J = 17.1 Hz, 1H, H^b-3), 4.73 (dqd, ^3J = 11.50 Hz, ^3J =6.9 Hz, ^3J = 3.5 Hz, 1H, H-2'), 7.46 – 7.50 (m, 2H, H-4, H-6), 7.56 (*virt.* td, $^3J \approx {}^3J$ = 7.5 Hz, ^4J = 1.1 Hz, 1H, H-5), 7.85 (*virt.* dt, ^3J = 7.5 Hz, $^4J \approx {}^5J$ = 1.1 Hz, 1H, H-7).

^{13}C NMR (126 MHz, $CDCl_3$): δ [ppm] = 169.8 (C-1), 141.3 (C-3a), 132.5 (C-7a), 131.6 (C-5), 128.4 (C-6), 124.0 (C-7), 123.0 (C-4), 58.6 (C-4'), 45.5 (C-3), 43.5 (C-2'), 38.0 (C-3'), 19.5 (C-1').

HRMS (ESI) *m/z*: $[M+H]^+$ Calcd. for $C_{12}H_{16}NO_2$ 206.1176; Found 206.1173.

IR (ATR): $\tilde{\nu}$ [cm^{-1}] = 3395 (m, OH), 2969 (w), 2933 (w), 2876 (w), 1659 (vs, C=O), 1470 (s), 1453 (s), 1413 (s), 1214 (m), 1052 (m), 736 (s, sp^2 C–H), 685 (m).

*2.1.5. 2-(4-Hydroxy-3-methylbutyl)isoindolin-1-one (**SI-1x**)*

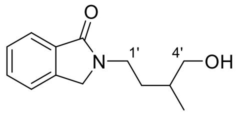

Following GP 1, compound **SI-1x** was obtained as a yellow oil (1.35 g, 6.14 mmol, 61%).

***R*f** (EtOAc/MeOH = 9/1): 0.68 (UV, $KMnO_4$].

^{1}H NMR (500 MHz, $CDCl_3$): δ [ppm] = 0.92 (d, ^3J = 6.8 Hz, 3H, Me), 1.46 (*virt.* ddt, ^2J = 13.9 Hz, ^3J = 8.0 Hz, $^3J \approx {}^3J$ = 6.8 Hz, 1H, H^a-2'), 1.58 - 1.69 (m, 1 H, H-3'), 1.78 (*virt.* ddt, ^2J = 13.9 Hz, ^3J = 8.0 Hz, $^3J \approx {}^3J$ = 6.3 Hz, 1H, H^b-2'), 3.44 (dd, ^2J = 10.7 Hz, ^3J = 6.6 Hz, 1H, H^a-4'), 3.52 (dd, ^2J = 10.7 Hz, ^3J = 5.5 Hz, 1H, H^b-4'), 3.59 (ddd, ^2J = 14.2 Hz, ^3J = 8.0 Hz, ^3J =6.3 Hz, 1H, H^a-1'), 3.73 (ddd, ^2J = 14.2 Hz, ^3J = 8.0 Hz, ^3J = 6.6 Hz, 1H, H^b-1'), 4.33 (s, 2H, H-3), 7.36 – 7.42 (m, 2H, H-4, H-6), 7.46 (*virt*. td, $^3J \approx {}^3J$ = 7.4 Hz, ^4J = 1.1 Hz, 1H, H-5), 7.77 (*virt*. dt, ^3J = 7.5 Hz, $^4J \approx {}^5J$ = 1.1 Hz, 1H, H-7).

^{13}C NMR (126 MHz, $CDCl_3$): δ [ppm] = 168.9 (C-1), 141.2 (C-3a), 133.0 (C-7a), 131.4 (C-5), 128.2 (C-6), 123.8 (C-7), 122.8 (C-4), 68.2 (C-4'), 50.0 (C-3), 40.7 (C-1'), 33.3 (C-3'), 32.4 (C-2'), 17.1 (Me).

HRMS (ESI) *m/z*: $[M+H]^+$ Calcd. for $C_{13}H_{18}NO_2$ 220.1332; Found 220.1335.

IR (ATR): $\tilde{\nu}$ [cm^{-1}] = 3400 (m, OH), 3051 (w), 2956 (w), 2919 (w), 2872 (w), 1664 (vs, C=O), 1619 (m, C=C), 1472 (s), 1456 (s), 1416 (s), 1303 (m), 1211 (m), 1045 (s), 731 (vs, sp^2 C–H), 683 (s).

2.2. General Procedure 2 (*GP 2*): *N*-Alkylation of Isoindolinones with Protected 3-Bromopropan-1-ol

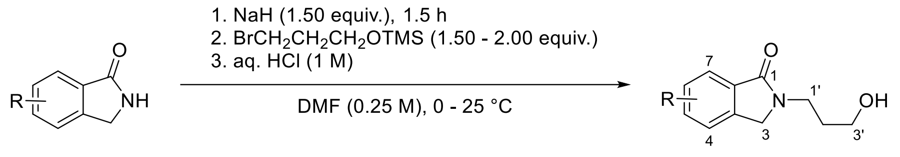

Based on a procedure by *Patil et al.*,[4] sodium hydride (60 wt% on mineral oil, 4.50 mmol, 1.50 equiv.) was added to a solution of the corresponding isoindolinone (3.00 mmol, 1.00 equiv.) in DMF (12 mL, 0.25 M) at 0 °C. The reaction mixture was allowed to warm to ambient temperature and stirred for an additional 1.5 h. The alkyl bromide (1.50 – 2.00 equiv.) was then added dropwise and the reaction mixture was stirred for 16 h. The reaction was quenched by addition of aq. HCl solution (1 M, 3.0 mL/mmol) and stirred for 1 h. The solvent was removed under reduced pressure and the residue was dissolved in CH_2Cl_2 (50 mL) and the resulting solution was washed with H_2O (50 mL). The aqueous layer was extracted with CH_2Cl_2 (2 × 30 mL). The combined organic layers were dried over Na_2SO_4, filtered and the solvent was removed under reduced pressure. The crude product was purified by automated flash column chromatography (hexanes/EtOAc) to yield the entitled free alcohol.

*2.2.1. 6-Chloro-2-(3-hydroxypropyl)isoindolin-1-one (**SI-1e**)*

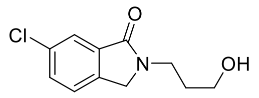

Following GP 1, compound **SI-1e** was obtained as a colorless solid (391 mg, 1.73 mmol, 58%).

***R*f** (EtOAc/MeOH = 9/1): 0.36 [UV, $KMnO_4$].

^{1}H NMR (500 MHz, $CDCl_3$): δ [ppm] = 1.82 – 1.87 (m, 2H, H-2'), 2.15 (bs, 1H, OH), 3.58 (t, ^3J = 5.7 Hz, 2H, H-3'), 3.78 (t, ^3J = 6.3 Hz, 2H, H-1'), 4.39 (s, 2H, H-3), 7.39 (dd, ^3J = 8.1 Hz, ^5J = 0.8 Hz, 1H, H-4), 7.52 (dd, ^3J = 8.1 Hz, ^4J = 2.0 Hz, 1H, H-5), 7.82 (d, ^3J = 2.0 Hz, 1H, H-7).

^{13}C NMR (126 MHz, $CDCl_3$): δ [ppm] = 168.6 (C-1), 139.4 (C-7a), 134.7 (C-3a), 134.2 (C-6), 131.9 (C-5), 124.1 (C-4), 124.1 (C-7), 58.4 (C-3'), 50.2 (C-3), 39. (C-1'), 30.9 (C-2').

HRMS (ESI) *m/z*: $[M+H]^+$ Calcd. for $C_{11}H_{13}ClNO_2$ 226.0629; Found 226.0629.

IR (ATR): $\tilde{\nu}$ [cm^{-1}] = 3386 (m, OH), 3073 (w), 2930 (w), 2866 (w), 1667 (vs, C=O), 1613 (w, C=C), 1584 (w, C=C), 1466 (s), 1410 (s), 1319 (m), 1266 (s), 1065 (s, C–Cl), 909 (s, sp^2 C–H), 843 (w, sp^2 C–H), 772 (s), 662 (s).

m.p. = 100-102 °C.

*2.2.2. 6-Bromo-2-(3-hydroxypropyl)isoindolin-1-one (**SI-1f**)*

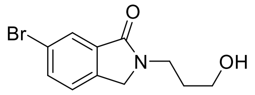

Following GP 1, compound **SI-1f** was obtained as a colorless solid (528 mg, 1.96 mmol, 65%).

***R*f** (EtOAc/MeOH = 9/1): 0.47 [UV, $KMnO_4$].

^{1}H NMR (500 MHz, $CDCl_3$): δ [ppm] = 1.82 – 1.87 (m, 2H, H-2'), 2.44 (bs, 1H, OH), 3.58 (t, ^3J = 5.7 Hz, 2H, H-1'), 3.77 (t, ^3J = 6.2 Hz, 2H, H-3'), 4.37 (s, 2H, H-3), 7.34 (dd, ^3J = 8.0 Hz, ^5J = 0.8 Hz, 1H, H-4), 7.67 (dd, ^3J = 8.0 Hz, ^4J = 1.9 Hz, 1H, H-5), 7.98 (d, ^3J = 1.9 Hz, 1H, H-7).

^{13}C NMR (126 MHz, $CDCl_3$): δ [ppm] = 168.5 (C-1), 139.9 (C-3a), 134.7 (C-5), 134.5 (C-7a), 127.1 (C-7), 124.5 (C-4), 122.5 (C-6), 58.4 (C-1'), 50.3 (C-3), 39.1 (C-3'), 30.9 (C-2').

HRMS (ESI) *m/z*: $[M+H]^+$ Calcd. for $C_{11}H_{13}BrNO_2$ 270.0124; Found 270.0120

IR (ATR): $\tilde{\nu}$ [cm^{-1}] = 3379 (m, OH), 3070 (w), 2925 (w), 2863 (w), 1666 (vs, C=O), 1608 (m, C=C), 1586 (m, C=C), 1459 (s), 1411 (m), 1315 (m), 1203 (m), 1052 (vs, C–Br), 819 (m, sp^2 C–H), 767 (vs), 746 (m).

m.p. = 107-108 °C

*2.2.3. 4-Methoxy-2-(3-hydroxypropyl)isoindolin-1-one (**SI-1l**)*

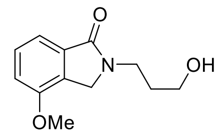

Following GP 1, compound **SI-1l** was obtained as a colorless solid (250 mg, 1.13 mmol, 38%).

***R*f** (EtOAc/MeOH = 9/1): 0.51 [UV, $KMnO_4$].

^{1}H NMR (500 MHz, $CDCl_3$): δ [ppm] = 1.79 – 1.85 (m, 2H, H-2'), 3.55 (t, ^3J = 5.6 Hz, 2H, H-1'), 3.78 (t, ^3J = 6.1 Hz, 2H, H-3'), 3.91 (s, 3H, OMe), 4.39 (s, 2H, H-3), 7.00 – 7.04 (m, 1H, H-6), 7.43 – 7.46 (m, 2H, H-5, H-7).

^{13}C NMR (126 MHz, $CDCl_3$): δ [ppm] = 170.0 (C-1), 154.6 (C-4), 134.0 (C-7a), 130.0 (C-6), 129.4 (C-3a), 116.0 (C-7), 112.9 (C-5), 58.2 (C-1'),55.6 (OMe), 48.4 (C-3), 38.8 (C-3'), 30.9 (C-2').

HRMS (ESI) *m/z*: $[M+H]^+$ Calcd. for $C_{12}H_{16}NO_3$ 222.1125; Found 226.1125

IR (ATR): $\tilde{\nu}$ [cm^{-1}] = 3396 (m, OH), 2933 (m), 2873 (w), 2842 (w), 1665 (vs, C=O), 1605 (s, C=C), 1494 (s), 1416 (m), 1271 (vs, Ar–O–CH_3), 1060 (m), 805 (w, sp^2 C–H), 750 (s, sp^2 C–H).

m.p. = 123-125 °C.

2.3. General procedure 3 (GP 3): *N*-Alkylation of Isoindolinones with 2-(2-Bromoethyl)-1,3-dioxolane

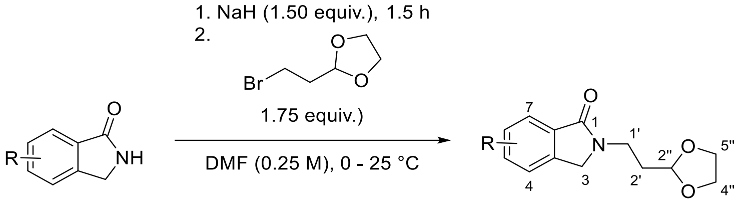

Based on a procedure of *Patil et al.*,[4] sodium hydride (60 wt% on mineral oil, 1.50 equiv.) was added to a solution of the corresponding isoindolinone (1.00 equiv.) in DMF (0.25 M) at 0 °C. The reaction mixture was allowed to warm to ambient temperature and stirred for an additional 1.5 h. The alkyl bromide (1.75 equiv.) was then added dropwise and the reaction mixture was stirred for 16 h. The solvent was removed under reduced pressure and the residue was dissolved in CH_2Cl_2 (50 mL). The resulting solution was washed with H_2O (30 mL) and the aqueous layer was extracted with CH_2Cl_2 (2 × 30 mL). The combined organic layers were washed with brine (50 mL), dried over Na_2SO_4, filtered and the solvent was removed under reduced pressure. The crude product was subjected to automated flash column chromatography (hexanes/EtOAc) to yield the entitled protected aldehydes.

*2.3.1. 2-(2-(1,3-dioxolan-2-yl)ethyl)-7-bromoisoindolin-1-one (**SI-2c**)*

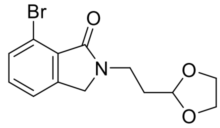

Following GP 3 starting from 7-bromoisoindolinone (1.06 g, 5.00 mmol), compound **SI-2c** was obtained as a pale yellow solid (1.34 g, 4.28 mmol, 86%).

***R*f** (Hex/EtOAc = 1/4): 0.35 (UV, $KMnO_4$].

^{1}H NMR (500 MHz, $CDCl_3$): δ [ppm] = 2.05 (td, ^3J = 7.1 Hz, ^3J = 4.5 Hz, 2H, H-2'), 3.76 (t, ^3J = 7.1 Hz, 2H, H-1'), 3.81 – 3.89 (m, 2H, H^a-4'', H^a-5''), 3.92 – 4.00 (m , 2H, H^b-4'', H^b-5''), 4.39 (s, 2H, H-3), 4.95 (t, ^3J = 4.5 Hz, 1H, H-1''), 7.57 – 7.62 (m, 2H, H-4, H-5), 7.70 (dd, ^3J = 7.9 Hz, ^4J = 0.8 Hz, 1H, H-6).

^{13}C NMR (126 MHz, $CDCl_3$): δ [ppm] = 167.87 (C-1), 143.1 (C-7a), 132.0 (C-3a), 131.6 (C-5), 126.2 (C-4), 126.0 (C-7), 125.2 (C-6), 102.7 (C-1''), 65.1 (C-4'', C-5''), 49.8 (C-3), 38.0 (C-1'), 32.6 (C-2').

HRMS (ESI) *m/z*: $[M+H]^+$ Calcd. for $C_{13}H_{15}BrNO_3$ 312.0230; Found 312.0226.

IR (ATR): $\tilde{\nu}$ [cm^{-1}] = 3078 (w), 2969 (w), 2937 (w), 2878 (m), 1637 (vs, C=O), 1603 (m, C=C), 1575 (m, C=C), 1458 (s), 1400 (s), 1383 (m, O–C–O), 1212 (s), 1129 (s), 1029 (vs, C–Br), 985 (s), 883 (vs, sp^2 C–H), 791 (vs), 778 (s), 675 (s).

m.p. = 77-78 °C.

*2.3.2. 2-(2-(1,3-dioxolan-2-yl)ethyl)-7-methylisoindolin-1-one (**SI-2d**)*

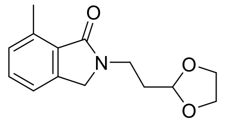

Following GP 3starting from 7-methylisoindolinone (265 mg, 1.80 mmol), compound **SI-2d** was obtained as a pale yellow oil (319 mg, 1.29 mmol, 72%).

***R*f** (Hex/EtOAc = 1/1): 0.24 (UV, $KMnO_4$].

^{1}H NMR (500 MHz, $CDCl_3$): δ [ppm] = 2.02 – 2.07 (m, 2H, H-2′), 2.72 (s, 3H, CH_3), 3.73 (t, ^3J = 7.3 Hz, 2H, H-1′), 3.82 – 3.90 (m, 2H, H^a-4′′, H^a-5′′), 3.93 – 4.01 (m , 2H, H^b-4′′, H^b-5′′), 4.84 (s, 2H, H-3), 4.96 (t, ^3J = 4.6 Hz, 1H, H-1′′), 7.17 (dd, ^3J = 7.5 Hz, 1H, H-6), 7.23 (d, ^3J = 7.5 Hz, 1H, H-4), 7.37 (*virt*. t, $^3J \approx {}^3J$ = 7.5 Hz, 1H, H-5).

^{13}C NMR (126 MHz, $CDCl_3$): δ [ppm] = 169.4 (C-1), 141.9 (C-3a), 137.7 (C-7), 130.8 (C-5), 130.1 (C-7a), 130.0 (C-6), 120.2 (C-4), 102.8 (C-1′′), 65.1 (C-4′′, C-5′′), 49.7 (C-3), 37.8 (C-1′), 32.8 (C-2′), 17.4 (CH_3).

HRMS (ESI) *m/z*: $[M+H]^+$ Calcd. for $C_{14}H_{18}NO_3$ 248.1281; Found 248.1281.

IR (ATR): $\tilde{\nu}$ [cm^{-1}] = 2928 (w), 2884 (w), 1672 (vs, C=O), 1907 (m, C=C), 1460 (m), 1409 (m), 1328 (m, O–C–O), 1283 (m), 1204 (m), 1131 (s, O–C–O), 1025 (s), 897 (m), 769 (vs, sp^2 C–H), 684 (s)

*2.3.3. 2-(2-(1,3-dioxolan-2-yl)ethyl)-5-fluoroisoindolin-1-one (**SI-2g**)*

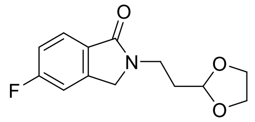

Following GP 3 starting from 5-fluoroisoindolinone (605 mg, 4.00 mmol), compound **SI-2g** was obtained as a pale yellow solid (650 mg, 2.59 mmol, 65%).

***R*f** (EtOAc): 0.49 (UV, $KMnO_4$].

^{1}H NMR (500 MHz, $CDCl_3$): δ [ppm] = 2.05 (td, ^3J = 7.2 Hz, ^3J = 4.5 Hz, 2H, H-2′), 3.75 (t, ^3J = 7.2 Hz, 2H, H-1′), 3.81 – 3.89 (m, 2H, H^a-4′′, H^a-5′′), 3.93 – 4.00 (m , 2H, H^b-4′′, H^b-5′′), 4.39 (s, 2H, H-3), 4.95 (t, ^3J = 4.5 Hz, 1H, H-1′′). 7.11 – 7.17 (m, 2H, H-4, H-6), 7.80 (dd, $^3J_{H–H}$ = 8.3 Hz, $^4J_{H–F}$ = 5.0 Hz, 1H, H-7).

^{13}C NMR (126 MHz, $CDCl_3$): δ [ppm] = 167.6 (C-1), 165.0 (d, $^1J_{C–F}$ = 250.5 Hz, C-5), 143.6 (d, $^3J_{C–F}$ = 10.1 Hz, C-3a), 129.1 (d, $^4J_{C–F}$ = 2.0 Hz, C-7a), 125.7 (d, $^3J_{C–F}$ = 9.8 Hz, C-7), 115.9 (d, $^2J_{C–F}$ = 23.5 Hz, C-6), 110.2 (d, $^2J_{C–F}$ = 24.1 Hz, C-4), 102.7 (C-1′′), 65.1 (C-4′′, C-5′′), 49.9 (d, $^4J_{C–F}$ = 2.6 Hz, C-3), 38.0 (C-1′), 32.7 (C-2′).

^{19}F NMR (376 MHz, $CDCl_3$): δ [ppm] = −108.3 (td, $^3J \approx {}^3J$ = 8.7 Hz, ^4J = 5.1 Hz, 1F).

HRMS (ESI) *m/z*: $[M+H]^+$ Calcd. for $C_{13}H_{15}FNO_3$ 252.1030; Found 252.1026.

IR (ATR): $\tilde{\nu}$ [cm^{-1}] = 3047 (w), 2947 (w), 2910 (w), 2879 (m), 1672 (vs, C=O), 1625 (s, C=C), 1603 (m, C=C), 1464 (vs), 1417 (vs), 1325 (m, O–C–O), 1247 (vs, C–F), 1120 (vs), 1087 (s), 1033 (s), 1022 (s), 939 (vs, sp^2 C–H), 883 (vs, sp^2 C–H), 767 (vs), 708 (s), 674 (vs).

m.p. = 117-118 °C.

*2.3.4. 2-(2-(1,3-dioxolan-2-yl)ethyl)-5-bromoisoindolin-1-one (**SI-2h**)*

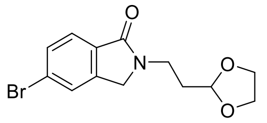

Following GP 3 starting from 5-bromoisoindolinone (1.06 g, 5.00 mmol), compound **SI-2h** was obtained as a pale yellow solid (1.34 g, 4.28 mmol, 86%).

***R*f** (Hex/EtOAc = 1/4): 0.34 (UV, $KMnO_4$].

^{1}H NMR (500 MHz, $CDCl_3$): δ [ppm] = 2.05 (td, ^3J = 7.1 Hz, ^3J = 4.5 Hz, 2H, H-2'), 3.76 (t, ^3J = 7.1 Hz, 2H, H-1'), 3.81 – 3.89 (m, 2H, H^a-4'', H^a-5''), 3.92 – 4.00 (m , 2H, H^b-4'', H^b-5''), 4.39 (s, 2H, H-3), 4.95 (t, ^3J = 4.5 Hz, 1H, H-1''). 7.57 – 7.61 (m, 2H, H-4, H-6), 7.80 (dd, ^3J = 7.9 Hz, ^5J = 0.8 Hz, 1H, H-7).

^{13}C NMR (126 MHz, $CDCl_3$): δ [ppm] = 167.8 (C-1), 143.1 (C-3a), 132.0 (C-7a), 131.6 (C-6), 126.2 (C-4), 126.0 (C-5), 125.2 (C-7), 102.7 (C-1''), 65.1 (C-4'', C-5''), 49.8 (C-3), 38.0 (C-1'), 32.6 (C-2').

HRMS (ESI) *m/z*: $[M+H]^+$ Calcd. for $C_{13}H_{15}BrNO_3$ 312.0230; Found 312.0225.

IR (ATR): $\tilde{\nu}$ [cm^{-1}] = 2956 (w), 2937 (w), 2885 (m), 1674 (vs, C=O), 1609 (m, C=C), 1585 (w), 1445 (s), 1436 (s), 1377 (s, O–C–O), 1316 (m), 1126 (s, O–C–O), 1059 (s, C–Br), 1012 (s), 952 (m), 895 (vs, sp^2 C–H), 764 (vs), 672 (s).

m.p. = 96-97 °C.

*2.3.5. 2-(2-(1,3-dioxolan-2-yl)ethyl)-1-oxoisoindoline-5-carbonitrile (**SI-2j**)*

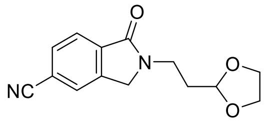

Following GP 3 starting from 1-oxoisoindoline-5-carbonitrile (253 mg, 1.60 mmol), compound **SI-2j** was obtained as a pale yellow solid (267 mg, 1.03 mmol, 65%).

***R*f** (Hex/EtOAc = 2/3): 0.13 (UV, $KMnO_4$].

^{1}H NMR (500 MHz, $CDCl_3$): δ [ppm] = 2.08 (td, ^3J = 7.1 Hz, ^3J = 4.4 Hz, 2H, H-2'), 3.80 (t, ^3J = 7.1 Hz, 2H, H-1'), 3.81 – 3.89 (m, 2H, H^a-4'', H^a-5''), 3.92 – 4.00 (m , 2H, H^b-4'', H^b-5''), 4.48 (s, 2H, H-3), 4.96 (t, ^3J = 4.4 Hz, 1H, H-1''). 7.74 – 7.78 (m, 2H, H-4, H-6), 7.94 (d, ^3J = 8.1 Hz, 1H, H-7).

^{13}C NMR (126 MHz, $CDCl_3$): δ [ppm] = 166.7 (C-1), 141.7 (C-7a), 137.0 (3a), 132.2 (C-6), 126.9 (C-4), 124.7 (C-7), 118.5 (CN), 114.8 (C-6), 102.6 (C-1''), 65.2 (C-4'', C-5''), 50.0 (C-3), 38.1 (C-1'), 32.5 (C-2').

HRMS (ESI) *m/z*: $[M+H]^+$ Calcd. for $C_{14}H_{14}N_2O_3$ 259.1077; Found 259.1076.

IR (ATR): $\tilde{\nu}$ [cm^{-1}] = 2960 (w), 2935 (w), 2895 (m), 2228 (m, C≡N), 1673 (vs, C=O), 1323 (m, C=C), 1590 (w, C=C), 1468 (s), 1436 (s), 1414 (s), 1324 (m, O–C–O), 1286 (m), 1131 (s), 1369 (s), 1019 (s), 977 (s), 934 (vs), 883 (vs, sp^2 C–H), 850 (s, sp^2 C–H), 771 (s), 680 (vs).

m.p. = 152-153 °C.

*2.3.6. 2-(2-(1,3-dioxolan-2-yl)ethyl)-4-bromoisoindolin-1-one (**SI-2m**)*

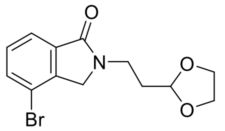

Following GP 3 starting from 4-bromoisoindolinone (636 mg, 3.00 mmol), compound **SI-2m** was obtained as a yellow oil (747 mg, 2.39 mmol, 80%).

***R*f** (Hex/EtOAc = 1/1): 0.37 (UV, $KMnO_4$].

^{1}H NMR (500 MHz, $CDCl_3$): δ [ppm] = 2.08 (ddd, ^{3}J = 8.0 Hz, ^{3}J = 6.8 Hz, ^{3}J = 4.5 Hz, 1H, H-2'), 3.76 – 3.81 (m, 2H, H-1'), 3.83 – 3.88 (m, 2H, H^a-4'', H^a-5''), 3.96 – 4.00 (m, 2H, H^b-4'', H^b-5''), 4.34 (s, 2H, H-3), 4.97 (t, ^{3}J = 4.5 Hz, 1H, H-1''), 7.36 (*virt*. t, $^{3}J \approx ^{3}J$ = 7.7 Hz, 1H, H-6), 7.65 (dd, ^{3}J = 7.7 Hz, ^{4}J = 0.9 Hz, 1H, H-5), 7.79 (dd, ^{3}J = 7.7 Hz, ^{4}J = 0.9 Hz, 1H, H-7)

^{13}C NMR (126 MHz, $CDCl_3$): δ [ppm] = 167.8 (C-1), 141.8 (C-3a), 135.2 (C-7a), 134.3 (C-5), 130.0 (C-6), 122.8 (C-7), 117.7 (C-4), 102.7 (C-1''), 65.2 (C-4'', C-5''), 51.0 (C-3), 38.0 (C-1'), 32.6 (C-2').

HRMS (ESI) *m/z*: $[M+H]^+$ Calcd. for $C_{13}H_{15}BrNO_3$ 312.0230; Found 312.0227.

IR (ATR): $\tilde{\nu}$ [cm^{-1}] = 2933 (w), 2884 (w), 1678 (vs, C=O), 1612 (w, C=C), 1579 (m, C=C), 1460 (m), 1411 (m), 1332 (w, O–C–O), 1269 (m), 1195 (m), 1129 (s), 1029 (m, C–Br), 803 (m), 748 (vs, sp^2 C–H).

2.4. General procedure 4 (GP 4): Synthesis of Isoindolin-1-ones from 2-Bromomethylbenzoates

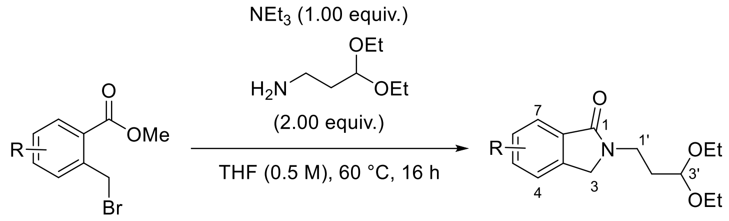

Based on a modified procedure by *Vermote et al.*,[5] 1-amino-3,3-diethoxypropane (2.00 equiv.) was added dropwise to a solution of benzoate (1.00 equiv.) and trimethylamine (1.00 equiv.) in THF (0.5 M) and the resulting reaction mixture was stirred at 60 °C for 16 h. After cooling to ambient temperature, the solvent was removed under reduced pressure. The residue was dissolved in CH_2Cl_2 (50 mL) and washed with H_2O (30 mL). The aqueous layer was extracted with CH_2Cl_2 (2 × 30 mL). The combined organic layers were dried over Na_2SO_4, filtered and the solvent was removed under reduced pressure. The crude product was subjected to automated flash column chromatography (Hex/EtOAc) to yield the desired isoindolin-1-ones.

*2.4.1. 2-(3,3-Diethoxypropyl) isoindolin-1-one (**SI-3a**)*

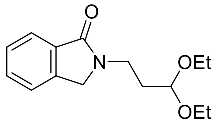

Following GP 4 starting from methyl 2-(bromomethyl)benzoate (916 mg, 4.00 mmol), compound **SI-3a** was obtained as a colorless oil (945 mg, 3.59 mmol, 90%).

***R*f** (Hex/EtOAc = 1/1): 0.28 [UV, $KMnO_4$].

^{1}H NMR (500 MHz, $CDCl_3$): δ [ppm] = 1.19 (t, ^3J = 7.1 Hz, 6H, CH_3), 2.02 (td, ^3J = 7.2 Hz, ^3J = 5.6 Hz, 2H, H-2’), 3.53 (dq, ^2J = 9.4 Hz, ^3J = 7.1 Hz, 2H, OCH_2^a), 3.68 (dq, ^2J = 9.4 Hz, ^3J = 7.1 Hz, 2H, OCH_2^b), 3.73 (t, ^3J = 7.2 Hz, 2H, H-1’), 4.42 (s, 2H, H-3), 4.60 (t, ^3J = 5.6 Hz, 1H, H-3’), 7.44 – 7.50 (m, 2H, H-4, H-6), 7.54 (*virt*. td, $^3J \approx {}^3J$ = 7.5 Hz, ^4J = 1.2 Hz, 1H, H-5), 7.86 (d, ^3J = 7.5 Hz, 1H, H-7).

^{13}C NMR (126 MHz, $CDCl_3$): δ [ppm] = 168.7 (C-1), 141.3 (C-3a), 133.1 (C-7a), 131.3 (C-5), 128.1 (C-6), 123.7 (C-7), 122.8 (C-4), 101.5 (C-3’), 62.0 (OCH_2), 50.3 (C-3), 38.8 (C-1’), 32.8 (C-2’), 15.4 (CH_3).

HRMS (ESI) *m/z*: $[M+Na]^+$ Calcd. for $C_{15}H_{21}NO_3Na$ 286.1414; Found 286.1410.

IR (ATR): $\tilde{\nu}$ [cm^{-1}] = 2974 (w), 2930 (w), 2878 (w), 1682 (vs, C=O), 1619 (w, C=C), 1472 (m), 1456 (m), 1412 (m), 1374 (m), 1303 (m), 1123 (s, O–C–O), 1056 (vs, O–C–O), 952 (m), 873 (w), 798 (w), 734 (vs, sp^2 C–H), 684 (m).

*2.4.2. 2-(3,3-Diethoxypropyl)-5-nitroisoindolin-1-one (**SI-3i**)*

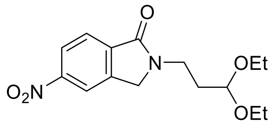

Following GP 4 starting from methyl 2-(bromomethyl)-4-nitrobenzoate (480 mg, 1.75 mmol), compound **SI-3i** was obtained as a pale yellow solid (432 mg, 1.40 mmol, 80%).

***R*f** (Hex/EtOAc = 1/1): 0.29 [UV, $KMnO_4$].

^{1}H NMR (500 MHz, $CDCl_3$): δ [ppm] = 1.15 (t, ^3J = 7.1 Hz, 6H, CH_3), 2.02 (td, ^3J = 7.0 Hz, ^3J = 5.4 Hz, 2H, H-2’), 3.49 (dq, ^2J = 9.3 Hz, ^3J = 7.0 Hz, 2H, OCH_2^a), 3.66 (dq, ^2J = 9.3 Hz, ^3J = 7.0 Hz, 2H, OCH_2^b), 3.79 (t, ^3J = 7.1 Hz, 2H, H-1’), 4.53 (s, 2H, H-3), 4.58 (t, ^3J = 5.4 Hz, 1H, H-3’), 7.98 (d, ^3J = 8.2 Hz, 1H, H-7), 8.32 (dd, ^4J = 2.0 Hz, ^5J = 0.9 Hz, 1H, H-4), 8.35 (dd, ^3J = 8.2 Hz, ^4J = 2.0 Hz, 1H, H-6).

^{13}C NMR (126 MHz, DMSO-d_6): δ [ppm] = 166.4 (C-1), 150.1 (C-5), 142.2 (C-3a), 138.5 (C-7a), 124.7 (C-7), 124.0 (C-6), 118.5 (C-4), 101.4 (C-3’), 62.1 (OCH_2), 50.2 (C-3), 39.2 (C-1’), 32.6 (C-2’), 15.4 (CH_3).

IR (ATR): $\tilde{\nu}$ [cm^{-1}] = 2980 (w), 2935 (w), 2917 (w), 2872(w), 1673 (vs, C=O), 1624 (w, C=C), 1525 (vs, NO_2), 1470 (m), 1456 (m), 1441 (m), 1340 (vs, NO_2), 1223 (m), 1156 (m), 1116 (s, O–C–O), 1061 (vs), 964 (s), 893 (m, sp^2 C–H), 814 (m, sp^2 C–H), 730 (vs), 666 (m).

m.p. = 99-102 °C

*2.4.3. Methyl 2-(3,3-diethoxypropyl)-1-oxoisoindoline-5-carboxylate (**SI-3k**)*

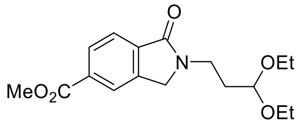

Following GP 4 starting from **SI-9** (237 mg, 975 µmol), compound **SI-3k** was obtained as a white solid (282 mg, 877 µmol, 90%).

***R*f** (Hex/EtOAc = 1/1): 0.30 [UV, $KMnO_4$].

^{1}H NMR (500 MHz, DMSO-d_6): δ [ppm] = 1.08 (t, ^3J = 7.0 Hz, 6H, CH_3), 1.87 (td, ^3J = 7.1 Hz, ^3J = 5.5 Hz, 2H, H-2’), 3.43 (dq, ^2J = 9.5 Hz, ^3J = 7.0 Hz, 2H, OCH_2^a), 3.53 – 3.62 (m, 4H, H-1’, OCH_2^b), 3.90 (s, 3H, OMe), 4.54 (t, ^3J = 5.5 Hz, 1H, H-3’), 4.55 (s, 2H, H-3), 7.78 (*d*, ^3J = 7.9 Hz, 1H, H-7), 8.05 (dd, ^3J = 7.9 Hz, ^4J = 1.5 Hz, 1H, H-6), 8.17 (dd, ^4J = 1.5 Hz, ^5J = 0.8 Hz, 1H, H-4).

^{13}C NMR (126 MHz, DMSO-d_6): δ [ppm] = 166.2 (C-1), 165.9 (*C*OOMe), 142.3 (C-3a), 136.6 (C-7a), 131.9 (C-5), 128.9 (C-6), 124.4 (C-4), 123.0 (C-7), 100.7 (C-3’), 60.9 (OCH_2), 52.5 (OMe), 49.7 (C-3), 38.2 (C-1’), 32.1 (C-2’), 15.3 (CH_3).

HRMS (ESI) *m/z*: $[M+Na]^+$ Calcd. for $C_{17}H_{23}NO_5Na$ 344.1468; Found 344.1464.

IR (ATR): $\tilde{\nu}$ [cm^{-1}] = 2975 (w), 2945 (w), 2917 (w), 2879 (w), 1721 (vs, COO), 1677 (vs, CON), 1623 (w, C=C), 1595 (w, C=C), 1434 (m), 1298 (s), 1273 (s), 1194 (m), 1123 (s, O–C–O), 1063 (vs), 966 (m), 867 (m, sp^2 C–H), 803 (m, sp^2 C–H), 742 (vs), 674 (m).

m.p. = 80-81 °C.

*2.4.4. 2-(3,3-Diethoxypropyl)-4-(trifluoromethyl)isoindolin-1-one (**SI-3n**)*

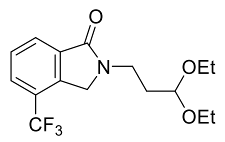

Following GP 4 starting from methyl 2-(bromomethyl)-3-(trifluoromethyl)benzoate (249 mg, 837 µmol), compound **SI-3n** was obtained as a colorless oil (258 mg, 778 µmol, 93%).

***R*f** (Hex/EtOAc = 1/1): 0.34 [UV, $KMnO_4$].

^{1}H NMR (500 MHz, DMSO-d_6): δ [ppm] = 1.06 (t, ^3J = 7.1 Hz, 6H, CH_3), 1.90 (td, ^3J = 7.1 Hz, ^3J = 5.4 Hz, 2H, H-2'), 3.43 (dq, ^2J = 9.5 Hz, ^3J = 7.0 Hz, 2H, OCH_2^a), 3.53 – 3.62 (m, 4H, H-1', OCH_2^b), 4.55 (t, ^3J = 5.4 Hz, 1H, H-3'), 4.68 (s, 2H, H-3), 7.73 (*virt*. t, $^3J \approx ^3J$ = 7.3 Hz, 1H, H-6), 7.94 (d, ^3J = 7.3 Hz, 1H, H-5), 7.97 (d, ^3J = 7.3 Hz, 1H, H-7).

^{13}C NMR (126 MHz, DMSO-d_6): δ [ppm] = 165.7 (C-1), 139.0 (q, $^3J_{C-F}$ = 2.7 Hz, C-4a), 134.3 (C-7a), 129.2 (C-6), 128.2 (q, $^3J_{C-F}$ = 4.3 Hz, C-5), 127.0 (C-7), 124.0 (q, $^2J_{C-F}$ = 32.9 Hz, C-4), 123.9 (q, $^1J_{C-F}$ = 273.1 Hz, CF_3), 100.8 (C-3'), 60.9 (OCH_2), 48.9 (q, $^4J_{C-F}$ = 2.4 Hz, C-3), 38.1 (C-2'), 31.9 (C-1'), 15.3 (CH_3).

^{19}F NMR (376 MHz, DMSO-d_6): −60.3 (s, 3F).

HRMS (ESI) *m/z*: $[M+Na]^+$ Calcd. for $C_{16}H_{20}F_3NO_3Na$ 354.1287; Found 354.1284.

IR (ATR): $\tilde{\nu}$ [cm^{-1}] = 2979 (w), 2937 (w), 1692 (s, C=O), 1619 (w, C=C), 1442 (m), 1418 (m), 1327 [vs, RC–H(OEt)$_2$], 1163 (s, O–C–O), 1113 (vs, C–F), 1058 (m), 820 (m), 753 (s, sp^2 C–H).

*2.4.5. 6-(3,3-Diethoxypropyl)-6,7-dihydro-5H-pyrrolo[3,4-b]pyridin-5-one (**SI-3p**)*

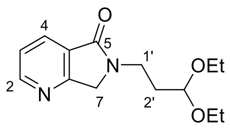

Following GP 4 starting from **SI-14** (928 mg, 5.00 mmol), compound **SI-3p** was obtained as a colorless oil (1.23 g, 4.65 mmol, 93%).

***R*f** (EtOAc): 0.22 [UV, $KMnO_4$].

^{1}H NMR (500 MHz, DMSO-d_6): δ [ppm] = 1.16 (t, ^3J = 7.1 Hz, 6H, CH_3), 2.02 (td, ^3J = 7.2 Hz, ^3J = 5.5 Hz, 2H, H-2'), 3.50 (dq, ^2J = 9.2 Hz, ^3J = 7.1 Hz, 2H, OCH_2^a), 3.66, (dq, ^2J = 9.2 Hz, ^3J = 7.1 Hz, 2H, OCH_2^b), 3.75 (t, ^3J = 7.2 Hz, 2H, H-1'), 4.47 (s, 2H, H-7), 4.59 (t, ^3J = 5.5 Hz, 1H, H-3'), 7.39 (dd, ^3J = 7.7 Hz, ^3J = 4.9 Hz, 1H, H-3), 8.10 (dd, ^3J = 7.7 Hz, ^4J = 1.6 Hz, 1H, H-4), 8.71 (dd, ^3J = 4.9 Hz, ^4J = 1.6 Hz, 1H, H-2).

^{13}C NMR (126 MHz, DMSO-d_6): δ [ppm] = 166.9 (C-5), 162.3 (C-7a), 152.5 (C-2), 131.8 (C-4), 126.8 (C-4a), 123.3 (C-3), 101.4 (C-3'), 61.7 (OCH_2), 51.9 (C-7), 38.7 (C-1'), 32.7 (C-2'), 15.4 (CH_3).

HRMS (ESI) *m/z*: $[M+H]^+$ Calcd. for $C_{14}H_{21}N_2O_3$ 265.1547; Found 265.1543.

IR (ATR): $\tilde{\nu}$ [cm^{-1}] = 2975 (w), 2930 (w), 2879 (w), 1687 (vs, C=O), 1609 (w, C=C), 1586 (w, C=N), 1474 (w), 1417 (m), 1374 (m), 1170 (m), 1122 (s, O–C–O), 1056 (vs, O–C–O), 922 (w), 820 (w), 750 (s, sp^2 C–H), 730 (s, sp^2 C–H).

2.5. General procedure 5 (GP 5): *Swern* Oxidation of Isoindolin-1-one based Alcohols

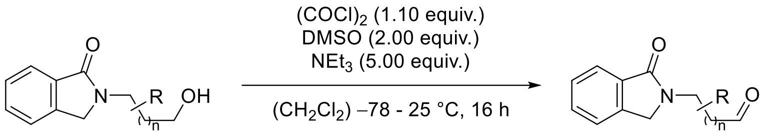

Based on a modified procedure by *Omura et al.*,[6] DMSO (2.00 equiv.) was added dropwise to a solution of $(COCl)_2$ (1.10 equiv.) in CH_2Cl_2 (200 mM) at −78 °C. After 15 min, a solution of alcohol (1.00 equiv.) in CH_2Cl_2 (1.0 M) was added dropwise and the reaction mixture was stirred for 30 min at −78 °C. Then, NEt_3 (5.00 equiv.) was added dropwise and the mixture was allowed to warm to r.t. overnight (16 h). The reaction was quenched by addition of H_2O (3 mL/mmol) and diluted with CH_2Cl_2 (3 mL/mmol). The aqueous layer was extracted with CH_2Cl_2 (3 × 15 mL). The combined organic layers were washed with brine (20 mL) dried over Na_2SO_4, filtered and the solvent was removed under reduced pressure. The crude product was used in the next step without further purification.

*2.5.1. 3-(1-Oxoisoindolin-2-yl)propanal (**SI-4a**)*

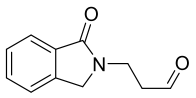

Following GP 5 compound **SI-4a** was obtained as a yellow oil (567 mg, 3.00 mmol, quant.).

*2.5.2. 2,2-Dimethyl-3-(1-oxoisoindolin-2-yl)propanal (**SI-4b**)*

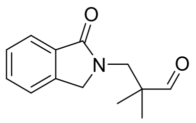

Following GP 5 compound **SI-4b** was obtained as a yellow oil (434 mg, 2.00 mmol, quant.).

*2.5.3. 3-(6-Chloro-1-oxoisoindolin-2-yl)propanal (**SI-4e**)*

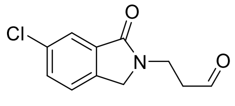

Following GP 5 compound **SI-4e** was obtained as a yellow oil (225 mg, 1.00 mmol, quant.).

*2.5.4. 3-(6-Bromo-1-oxoisoindolin-2-yl)propanal (**SI-4f**)*

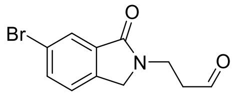

Following GP 5 compound **SI-4f** was obtained as a yellow oil (268 mg, 1.00 mmol, quant.).

*2.5.5. 3-(4-Methoxy-1-oxoisoindolin-2-yl)propanal (**SI-4l**)*

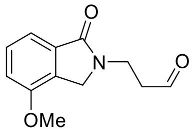

Following GP 5 compound **SI-4l** was obtained as a yellow oil (220 mg, 1.00 mmol, quant.).

*2.5.6. 4-(1-Oxoisoindolin-2-yl)butanal (**SI-4q**)*

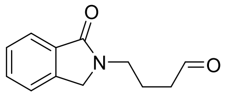

Following GP 5 compound **SI-4q** was obtained as a yellow oil (203 mg, 1.00 mmol, quant.).

*2.5.7. 3-(1-Oxoisoindolin-2-yl)butanal (**SI-4u**)*

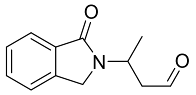

Following GP 5 compound **SI-4u** was obtained as a yellow oil (400 mg, 1.97 mmol, 97%).

*2.5.8. 2-Methyl-4-(1-oxoisoindolin-2-yl)butanal (**SI-4x**)*

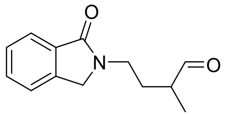

Following GP 5 compound **SI-4x** was obtained as a yellow oil (182 mg, 825 µmol, 84%).

2.6. General procedure 6 (GP 6): Deprotection of Diethyl Acetals and Dioxolanes

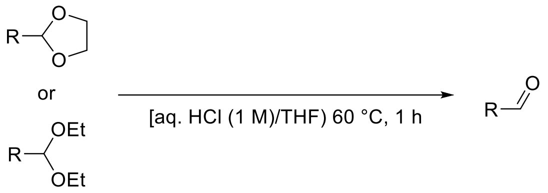

Based on a modified procedure by *Levin et al.*,[7] the corresponding acetal (1.00 equiv.) was suspended in aq. HCl (1 M, 2 mL/mmol) and heated to 60 °C. If necessary, THF was added at 60 °C, until all solids were dissolved, and the reaction mixture was stirred at 60 °C for 1 h. Afterwards, the aqueous layer was extracted with CH_2Cl_2 (3 × 20 mL). The combined organic layers were dried over Na_2SO_4, filtered and the solvent was removed under reduced pressure. The crude product was used in the next step without further purification.

*2.6.1. 3-(7-Bromo-1-oxoisoindolin-2-yl)propanal (**SI-4c**)*

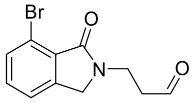

Following GP 6, compound **SI-4c** was obtained as a yellow oil (404 mg, 1.51 mmol, quant.).

*2.6.2. 3-(7-Methyl-1-oxoisoindolin-2-yl)propanal (**SI-4d**)*

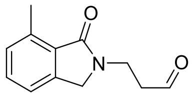

Following GP 6, compound **SI-4d** was obtained as a yellow oil (243 mg, 1.20 mmol, 99%).

*2.6.3. 3-(5-Fluoro-1-oxoisoindolin-2-yl)propanal (**SI-4g**)*

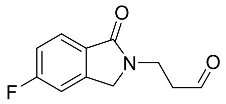

Following GP 6, compound **SI-4g** was obtained as a yellow oil (207 mg, 1.00 mmol, quant.).

*2.6.4. 3-(5-Bromo-1-oxoisoindolin-2-yl)propanal (**SI-4h**)*

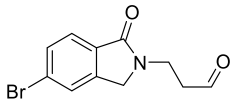

Following GP 6, compound **SI-4h** was obtained as a yellow oil (536 mg, 2.00 mmol, quant.).

*2.6.5. 3-(5-Nitro-1-oxoisoindolin-2-yl)propanal (**SI-4i**)*

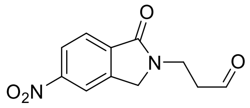

Following GP 6, compound **SI-4i** was obtained as a yellow oil (239 mg, 1.02 mmol, 96%).

*2.6.6. 1-Oxo-2-(3-oxopropyl)isoindoline-5-carbonitrile (**SI-4j**)*

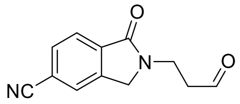

Following GP 6, compound **SI-4j** was obtained as a yellow oil (199 mg, 929 µmol, quant.).

*2.6.7. Methyl 1-oxo-2-(3-oxopropyl)isoindoline-5-carboxylate (**SI-4k**)*

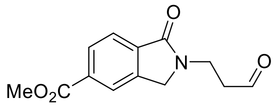

Following GP 6, compound **SI-4k** was obtained as a yellow oil (144 mg, 787 µmol, 98%).

*2.6.8. 3-(4-Bromo-1-oxoisoindolin-2-yl)propanal (**SI-4m**)*

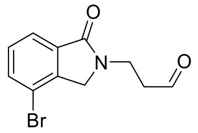

Following GP 6, compound **SI-4m** was obtained as a yellow oil (1.08 g, 4.04 mmol, quant.).

*2.6.9. 3-(1-Oxo-4-(trifluoromethyl)isoindolin-2-yl)propanal (**SI-4n**)*

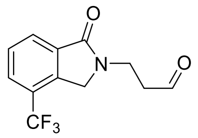

Following GP 6, compound **SI-4n** was obtained as a yellow oil (186 mg, 725 µmol, 91%).

*2.6.10. 3-(1-Methyl-3-oxoisoindolin-2-yl)propanal (**SI-4o**)*

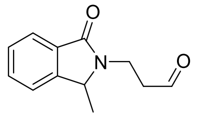

Following GP 6, compound **SI-4o** was obtained as a yellow oil (287 mg, 1.52 mmol, 97%).

*2.6.11. 3-(5-Oxo-5,7-dihydro-6H-pyrrolo[3,4-b]pyridin-6-yl)propanal (**SI-4p**)*

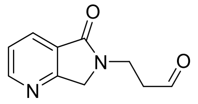

Following GP 6, the aqueous layer was collected and the solvent was removed under reduced pressure to obtain compound **SI-4p** as a yellow oil (551 mg, 2.90 mmol, 97%).

*2.6.12. 2-(2-(1-Oxoisoindolin-2-yl)ethyl)benzaldehyde (**SI-4r**)*

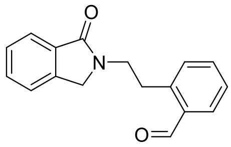

Following GP 6, compound **SI-4r** was obtained as a yellow oil (345 mg, 1.30 mmol, 92%).

***R*f** (Hex/EtOAc = 1/1): 0.43 (UV, $KMnO_4$].

^{1}H NMR (500 MHz, DMSO-d_6): δ [ppm] = 2.31 (s, 3H, CH_3), 2.47 (td, ^3J = 6.7 Hz, ^3J = 5.1 Hz, 2H, H-2'), 3.61 (t, ^3J = 6.7 Hz, 2H, H-3'), 4.34 (s, 2H, H-3''), 7.21 – 7.25 (m, 2H, H-3), 7.27 (t, ^3J = 5.1 Hz, 1H, H-1'), 7.48 (*virt*. td, $^3J \approx {}^3J$ = 7.5 Hz, 1H, H-6''), 7.53 (d, ^3J = 7.4 Hz, 1H, H-4''), 7.58 – 7.62 (m, 3H, H-2, H-5''), 7.64 (*virt*. dt, ^3J = 7.5 Hz, $^4J \approx {}^5J$ = 1.0 Hz, 1H, H-7''), 11.05 (s, 1H, NH).

^{13}C NMR (126 MHz, DMSO-d_6): δ [ppm] = 167.3 (C-1''), 149.3 (C-1'), 143.1 (C-4), 141.9 (C-7a''), 136.2 (C-1), 132.2 (C-3a''), 131.3 (C-5''), 129.5 (C-3), 127.8 (C-6''), 127.0 (C-2), 123.3 (C-4''), 122.7 (C-7''), 49.5 (C-3''), 38.7 (C-3'), 31.1 (C-2'), 21.1 (CH_3).

HRMS (ESI) *m/z*: $[M+H]^+$ Calcd. for $C_{17}H_{16}NO_2$ 358.1220; Found 358.1214.

IR (ATR): $\tilde{\nu}$ [cm^{-1}] = 2939 (w), 2916 (w), 2775 (w, CHO), 2740 (w, CHO), 1690 (s, CH=O), 1665 (vs, CON), 1618 (w, C=C), 1600 (w, C=C), 1475 (m), 1451 (m), 1412 (s), 1361 (m), 1308 (m), 1250 (m), 1194 (s), 1011 (m), 873 (m), 773 (s, sp^2 C–H), 732 (vs, sp^2 C–H), 685 (m).

m.p. 133-135 °C.

2.7. General procedure 7 (GP 7): Synthesis of Hydrazones

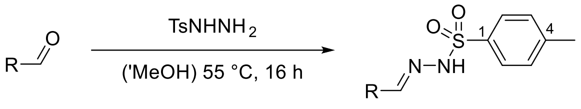

Based on a modified procedure by *Zhang et al.*,[8] the aldehyde (1.00 equiv.) and *p*-toluenesulfonyl hydrazide (1.00 equiv.) were suspended in MeOH (1 M) and the suspension was heated to 55 °C to become a clear solution. After 16 h at 55 °C, the reaction mixture was cooled to ambient temperature. The precipitate formed during the reaction was either collected directly or the solvent was removed under reduced pressure. The solid thus obtained was collected and washed with Et_2O (2 mL) and ice-cold MeOH (1 mL) to yield the entitled hydrazone as colorless solids.

*2.7.1. N'-(3-(1-Oxoisoindolin-2-yl)propylidene)-4-methylbenzenesulfonohydrazide (**2a**)*

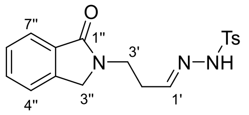

Following GP 7, compound **2a** was obtained as a colorless solid (1.09 g, 3.06 mmol, 87%).

***R*f** (CH_2Cl_2/ac = 9/1): 0.21 [UV, $KMnO_4$].

^{1}H NMR (500 MHz, DMSO-*d*6): δ [ppm] = 2.31 (s, 3H, CH_3), 2.47 (td, ^3J = 6.7 Hz, ^3J = 5.1 Hz, 2H, H-2'), 3.61 (t, ^3J = 6.7 Hz, 2H, H-3'), 4.34 (s, 2H, H-3''), 7.21 – 7.25 (m, 2H, H-3), 7.27 (t, ^3J = 5.1 Hz, 1H, H-1'), 7.48 (*virt*. td, $^3J \approx {}^3J$ = 7.5 Hz, ^4J =1.1 Hz, 1H, H-6''), 7.53 (d, ^3J = 7.4 Hz, 1H, H-4''), 7.58 – 7.62 (m, 3H, H-2, H-5''), 7.64 (*virt*. dt, ^3J = 7.5 Hz, $^4J \approx {}^5J$ = 1.0 Hz, 1H, H-7''), 11.05 (s, 1H, NH).

^{13}C NMR (126 MHz, DMSO-*d*6): δ [ppm] = 167.3 (C-1''), 149.3 (C-1'), 143.1 (C-4), 141.9 (C-3a''), 136.2 (C-1), 132.2 (C-7a''), 131.3 (C-5''), 129.5 (C-3), 127.8 (C-6''), 127.0 (C-2), 123.3 (C-4''), 122.7 (C-7''), 49.5 (C-3''), 38.7 (C-3'), 31.1 (C-2'), 21.1 (CH_3).

HRMS (ESI) *m/z*: $[M+H]^+$ Calcd. for $C_{18}H_{20}N_3O_3S$ 358.1220; Found 358.1214.

IR (ATR): $\tilde{\nu}$ [cm^{-1}] = 3090 (m, NH), 2886 (w), 2776 (w), 1664 (vs, C=O), 1619 (m, C=C), 1594 (m, C=C), 1474 (m), 1454 (s), 1422 (m), 1365 (m), 1341 (s, RSO_2N), 1322 (m), 1161 (vs, RSO_2N), 1068 (s), 1025 (m), 941 (m), 809 (m, sp^2 C–H), 734 (vs, sp^2 C–H), 664 (vs).

m.p. > 168 °C (decomposition).

*2.7.2. N'-(2,2-Dimethyl-3-(1-oxoisoindolin-2-yl)propylidene)-4-methylbenzenesulfonohydrazide (**2b**)*

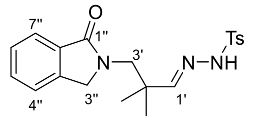

Following GP 7, compound **2b** was obtained as a colorless solid (411 mg, 1.07 mmol, 54%).

***R*f** (CH_2Cl_2/ac = 9/1): 0.36 [UV, $KMnO_4$].

^{1}H NMR (500 MHz, DMSO-*d*6): δ [ppm] = 0.97 [s, 6H, $(CH_3)_2$], 2.20 (s, 3H, CH_3), 3.44 (s, 2H, C-3'), 4.05 (s, 2H, H-3''), 7.21 – 7.25 (m, 2H, H-3), 7.29 (s, 1H, H-1'), 7.40 (d, ^3J = 7.6 Hz, 1H, H-4''), 7.48 (*virt.* td $^3J \approx ^3J$ = 7.5 Hz, ^4J = 1.0 Hz, 1H, H-6''), 7.59 – 7.66 (m, 4H, H-2, H-5'', H-7''), 10.95 (s, 1H, NH).

^{13}C NMR (126 MHz, DMSO-*d*6): δ [ppm] = 168.1 (C-1''), 156.5 (C-1'), 143.2 (C-4), 141.8 (C-3a''), 136.0 (C-1), 131.6 (C-7a''), 131.4 (C-5''), 129.4 (C-3), 127.8 (C-6''), 127.1 (C-2), 123.1 (C-4''), 122.9 (C-7''), 51.6 (C-3''), 50.8 (C-3'), 40.2 (C-2'), 23.4 [$(CH_3)_2$], 21.0 (CH_3).

HRMS (ESI) *m/z*: $[M+H]^+$ Calcd. for $C_{20}H_{24}N_3O_3S$ 386.1533; Found 386.1535.

IR (ATR): $\tilde{\nu}$ [cm^{-1}] = 3044 (m, NH), 2970 (w), 2882 (w), 2774 (w), 1660 (s, C=O), 1639 (m, C=N), 1697 (w, C=C), 1472 (m), 1452 (m), 1436 (m), 1354 (m), 1323 (vs, RSO_2N), 1159 (vs, RSO_2N), 1046 (m), 811 (m, sp^2 C–H), 736 (vs, sp^2 C–H), 704 (s), 658 (s).

m.p. > 180 °C (decomposition).

*2.7.3. N'-(3-(7-Bromo-1-oxoisoindolin-2-yl)propylidene)-4-methylbenzenesulfonohydrazide (**2c**)*

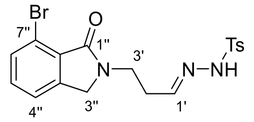

Following GP 7, compound **2c** was obtained as a colorless solid (513 mg, 1.18 mmol, 79%).

***R*f** (CH_2Cl_2/ac = 9/1): 0.21 [UV, $KMnO_4$].

^{1}H NMR (500 MHz, DMSO-*d*6): δ [ppm] = 2.31 (s, 3H, CH_3), 2.48* (dt, ^3J = 6.7 Hz, ^3J = 5.1 Hz, 2H, H-2'), 3.59 (t, ^3J = 6.7 Hz, 2H, H-3'), 4.31 (s, 2H, H-3''), 7.20 – 7.24 (m, 2H, H-3), 7.27 (t, ^3J = 5.1 Hz, 1H, H-1'), 7.50 (*virt.* t, ^3J= 7.5 Hz, 1H, H-5''), 7.54 (d, ^3J= 7.5 Hz, 1H, H-4''), 7.57 – 7.61 (m, 2H, H-2), 7.64 (d, ^3J= 7.5 Hz, 1H, H-6''), 11.07 (s, 1H, NH).

* signal overlaps with the residual solvent signal

^{13}C NMR (126 MHz, DMSO-*d*6): δ [ppm] = 165.2 (C-1''), 149.2 (C-1'), 145.0 (C-3a''), 143.1 (C-4), 136.2 (C-1), 132.8 (C-5''), 132.4 (C-6''), 129.5 (C-3), 129.3 (C-7a''), 127.0 (C-2), 122.9 (C-4''), 117.3 (C-7), 48.5 (C-3''), 38.9 (C-3'), 30.9 (C-2'), 21.1 (CH_3).

HRMS (ESI) *m/z*: $[M+H]^+$ Calcd. for $C_{18}H_{19}BrN_3O_3S$ 436.0320; Found 436.0325.

IR (ATR): $\tilde{\nu}$ [cm^{-1}] = 3026 (w, NH), 2933 (w), 2917 (w), 2872 (w), 2774 (w), 1663 (vs, C=O), 1607 (w, C=C), 1596 (w, C=C), 1465 (m), 1414 (m), 1324 (s, RSO_2N), 1200 (m), 1162 (vs, RSO_2N), 1069 (s, C–Br), 922 (m), 887 (m), 838 (m, sp^2 C–H), 814 (s, sp^2 C–H), 766 (s, sp^2 C–H), 665 (vs).

m.p. > 216 °C (decomposition).

*2.7.4. N'-(3-(7-methyl-1-oxoisoindolin-2-yl)propylidene)-4-methylbenzenesulfonohydrazide (**2d**)*

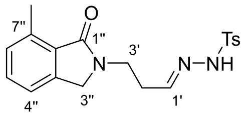

Following GP 7, compound **2d** was obtained as a colorless solid (346 mg, 931 µmol, 82%).

***R*f** (CH_2Cl_2/ac = 9/1): 0.16 [UV, $KMnO_4$].

^{1}H NMR (500 MHz, DMSO-*d*6): δ [ppm] = 2.31 (s, 3H, CH_3), 2.46 (dt, ^3J = 6.7 Hz, ^3J = 5.1 Hz, 2H, H-2'), 3.57 (t, ^3J = 6.7 Hz, 2H, H-3'), 4.26 (s, 2H, H-3''), 7.19 – 7.25 (m, 3H, H-3, H-6''), 7.27 (t, ^3J = 5.1 Hz, 1H, H-1'), 7.30 (d, ^3J = 7.6 Hz, 1H, H-4''), 7.44 (*virt*. t, ^3J= 7.5 Hz, 1H, H-5''), 7.58 – 7.63 (m, 2H, H-2), 11.05 (s, 1H, NH).

^{13}C NMR (126 MHz, DMSO-*d*6): δ [ppm] = 168.1 (C-1''), 149.3 (C-1'), 143.1 (C-4), 142.3 (C-7''), 136.2 (C-1), 136.0 (C-3a''), 130.8 (C-5''), 129.5 (2C, C-3, C-6''), 129.1 (C-7a''), 127.0 (C-2), 120.7 (C-4''), 48.9 (C-3''), 38.6 (C-3'), 31.1 (C-2'), 21.0 (CH_3), 16.7 (C-7''-CH_3).

HRMS (ESI) *m/z*: $[M+H]^+$ Calcd. for $C_{19}H_{22}N_3O_3S$ 372.1373; Found 372.1376.

IR (ATR): $\tilde{\nu}$ [cm^{-1}] = 3011 (w, NH), 2932 (w), 2917 (w), 2864 (w), 2835 (w), 2762 (w), 1648 (vs, C=O), 1613 (w, C=C), 1596 (w, C=C), 1458 (m), 1416 (m), 1325 (s. RSO_2N), 1289 (m), 1206 (m), 1163 (vs, RSO_2N), 1068 (s), 813 (s, sp^2 C–H), 762 (vs, sp^2 C–H), 667 (vs).

m.p. > 199 °C (decomposition).

*2.7.5. N'-(3-(6-Chloro-1-oxoisoindolin-2-yl)propylidene)-4-methylbenzenesulfonohydrazide (**2e**)*

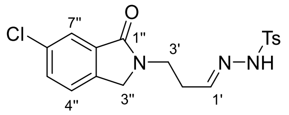

Following GP 7, compound **2e** was obtained as a colorless solid (220 mg, 561 µmol, 56%).

***R*f** (CH_2Cl_2/ac = 9/1): 0.17 [UV, $KMnO_4$].

^{1}H NMR (500 MHz, DMSO-*d*6): δ [ppm] = 2.31 (s, 3H, CH_3), 2.48* (dt, ^3J = 6.7 Hz, ^3J = 5.1 Hz, 2H, H-2'), 3.61 (t, ^3J = 6.7 Hz, 2H, H-3'), 4.34 (s, 2H, H-3''), 7.20 – 7.24 (m, 2H, H-3), 7.26 (t, ^3J = 5.1 Hz, 1H, H-1'), 7.56 – 7.61 (m, 3H, H-2, H-4''), 7.62 (d, ^4J = 1.8 Hz, 1H, H-7''), 7.66 (dd, ^3J = 8.1 Hz, ^4J = 1.8 Hz, 1H, H-5''), 11.06 (s, 1H, NH).

* signal overlaps with the residual solvent signal

^{13}C NMR (101 MHz, DMSO-*d*6): δ [ppm] = 165.9 (C-1''), 149.0 (C-1'), 143.0 (C-4), 140.5 (C-3a''), 136.2 (C-1), 134.2 (C-7a''), 132.7 (C-6''), 131.2 (C-5''), 129.4 (C-3), 126.9 (C-2), 125.3 (C-4''), 122.3 (C-7''), 49.3 (C-3''), 38.8 (C-3'), 31.0 (C-2'), 21.0 (CH_3).

HRMS (ESI) *m/z*: $[M+H]^+$ Calcd. for $C_{20}H_{22}N_3O_5S$ 416.1275; Found 416.1270.

IR (ATR): $\tilde{\nu}$ [cm^{-1}] = 3057 (w, NH), 2879 (w), 1664 (s, C=O), 1598 (w, C=C), 1584 (w, C=C), 1462 (m), 1447 (m), 1354 (m), 1323 (s, RSO_2N), 1294 (m), 1163 (vs, RSO_2N), 1051 (s, C–Cl), 957 (m), 931 (m), 813 (vs, sp^2 C–H), 770 (s, sp^2 C–H), 731 (s), 664 (s).

m.p. > 197 °C (decomposition).

*2.7.6. N'-(3-(6-Bromo-1-oxoisoindolin-2-yl)propylidene)-4-methylbenzenesulfonohydrazide (**2f**)*

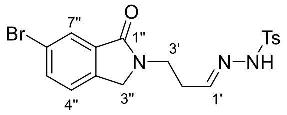

Following GP 7, compound **2f** was obtained as a colorless solid (272 mg, 654 µmol, 85%).

***R*f** (CH_2Cl_2/ac = 9/1): 0.22 [UV, $KMnO_4$].

^{1}H NMR (500 MHz, DMSO-*d*$_6$): δ [ppm] = 2.31 (s, 3H, CH_3), 2.47* (dt, ^{3}J = 6.7 Hz, ^{3}J = 5.1 Hz, 2H, H-2'), 3.61 (t, ^{3}J = 6.7 Hz, 2H, H-3'), 4.32 (s, 2H, H-3''), 7.20 – 7.24 (m, 2H, H-3), 7.26 (t, ^{3}J = 5.1 Hz, 1H, H-1'), 7.52 (d, ^{3}J = 8.0 Hz, 1H, H-4''), 7.56 – 7.61 (m, 2H, H-2), 7.75 (d, ^{4}J = 1.7 Hz, 1H, H-7''), 8.10 (dd, ^{3}J = 8.0 Hz, ^{4}J = 1.7 Hz, 1H, H-5''), 11.06 (s, 1H, NH).

* signal overlaps with the residual solvent signal

^{13}C NMR (126 MHz, DMSO-*d*$_6$): δ [ppm] = 165.9 (C-1''), 149.1 (C-1'), 143.1 (C-4), 141.0 (C-3a''), 136.2 (C-1), 134.5 (C-7a''), 134.0 (C-5''), 129.5 (C-3), 127.0 (C-2), 125.7 (C-4''), 125.3 (C-7''), 121.0 (C-6''), 49.4 (C-3''), 38.8 (C-3'), 31.0 (C-2'), 21.1 (CH_3).

HRMS (ESI) *m/z*: $[M+H]^+$ Calcd. for $C_{18}H_{19}BrN_3O_3S$ 436.0320; Found 436.0325.

IR (ATR): $\tilde{\nu}$ [cm^{-1}] = 3067 (w, NH), 2881 (w), 2774 (w), 1666 (vs, C=O), 1599 (w, C=C), 1584 (w, C=C), 1464 (m), 1446 (m), 1422 (m), 1323 (s, RSO_2N), 1225 (m), 1163 (vs, RSO_2N), 1051 (s, C–Br), 929 (m), 813 (vs, sp^2 C–H), 769 (s, sp^2 C–H), 733 (s).

m.p. > 188 °C (decomposition).

*2.7.7. N'-(3-(5-Fluoro-1-oxoisoindolin-2-yl)propylidene)-4-methylbenzenesulfonohydrazide (**2g**)*

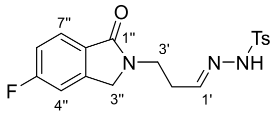

Following GP 7, compound **2g** was obtained as a colorless solid (210 mg, 559 µmol, 56%).

***R*f** (CH_2Cl_2/ac = 9/1): 0.14 [UV, $KMnO_4$].

^{1}H NMR (500 MHz, DMSO-*d*$_6$): δ [ppm] = 2.31 (s, 3H, CH_3), 2.47 (td, ^{3}J = 6.7 Hz, ^{3}J = 5.1 Hz,2H, H-2'), 3.59 (t, ^{3}J = 6.7 Hz, 2H, H-3'), 4.33 (s, 2H, H-3''), 7.23 – 7.28 (m, 3H, H-3, H-1'), 7.31 (ddd, $^{3}J_{H–F}$ = 10.6 Hz, ^{3}J = 8.4 Hz, ^{4}J = 2.3 Hz, 1H, H-6''), 7.41 (dd, $^{3}J_{H–F}$ = 8.8 Hz, ^{4}J = 2.3 Hz, 1H, H-4''), 7.58 – 7.62 (m, 2H, H-2), 7.66 (dd, ^{3}J = 8.4 Hz, $^{4}J_{H–F}$ = 5.1 Hz, 1H, H-7''), 11.06 (s, 1H, NH).

^{13}C NMR (126 MHz, DMSO-*d*$_6$): δ [ppm] = 166.4 (C-1''), 164.2 (d, $^{1}J_{C–F}$ = 247.2 Hz, C-5''), 149.2 (C-1'), 144.6 (d, $^{3}J_{C-F}$ = 10.5 Hz, C-3a''), 143.1 (C-4), 136.2 (C-1), 129.5 (C-3), 128.6 (d, $^{4}J_{C–F}$ = 2.0 Hz, C-7a''), 127.0 (C-2), 124.9 (d, $^{3}J_{C–F}$ = 9.9 Hz, C-7''), 115.5 (d, $^{2}J_{C–F}$ = 23.6 Hz, C-6''), 110.7 (d, $^{2}J_{C–F}$ = 24.3 Hz, C-4''), 49.31 (d, $^{4}J_{C–F}$ = 2.7 Hz, C-3''), 38.8 (C-3'), 31.1 (C-2'), 21.0 (CH_3).

^{19}F NMR (376 MHz, DMSO-*d*$_6$): δ [ppm] = −109.0 (td, ^{3}J = 9.2 Hz, ^{4}J = 5.3 Hz, 1F).

HRMS (ESI) *m/z*: $[M+H]^+$ Calcd. for $C_{18}H_{19}FN_3O_3S$ 376.1126; Found 376.1125.

IR (ATR): $\tilde{\nu}$ [cm^{-1}] = 3089 (m, NH), 2871 (w), 1687 (vs, C=O), 1670 (s, C=N), 1626 (m, C=C), 1598 (w, C=C), 1462 (s), 1321 (m, RSO_2N), 1251 (s), 1160 (vs, C–F), 1095 (m), 1059 (m), 905 (m), 859 (s, sp^2 C–H), 815 (m, sp^2 C–H), 715 (m), 662 (vs).

m.p. > 152 °C (decomposition).

*2.7.8. N'-(3-(5-Bromo-1-oxoisoindolin-2-yl)propylidene)-4-methylbenzenesulfonohydrazide (**2h**)*

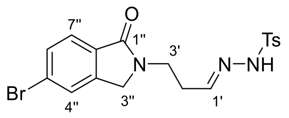

Following GP 7, compound **2h** was obtained as a colorless solid (253 mg, 579 µmol, 58%).

***R*f** (CH_2Cl_2/ac = 9/1): 0.12 [UV, $KMnO_4$].

^{1}H NMR (500 MHz, DMSO-*d*$_6$): δ [ppm] = 2.32 (s, 3H, CH_3), 2.46 (td, ^3J = 6.7 Hz, ^3J = 5.1 Hz,2H, H-2’), 3.60 (t, ^3J = 6.7 Hz, 2H, H-3’), 4.33 (s, 2H, H-3’’), 7.21 – 7.28 (m, 3H, H-3, H-1’), 7.56 (d, ^3J = 8.1 Hz, 1H, H-7’’), 7.57 – 7.62 (m, 2H, H-2), 7.67 (dd, ^3J = 8.1 Hz, ^4J = 1.7 Hz, 1H, H-6’’), 7.80 (d, ^4J = 1.7 Hz, 1H, H-4’’), 11.06 (s, 1H, NH).

^{13}C NMR (126 MHz, DMSO-*d*$_6$): δ [ppm] = 166.4 (C-1’’), 149.1 (C-1’), 144.1 (C-4), 143.1 (C-7a’’), 136.2 (C-1), 131.5 (C-3a’’), 131.0 (C-6’’), 129.5 (C-3), 127.0 (C-2), 126.6 (C-4’’), 125.0 (C-5’’), 124.6 (C-7’’),49.2 (C-3’’), 38.7 (C-3’), 31.0 (C-2’), 21.1 (CH_3).

HRMS (ESI) *m/z*: [M+H]$^+$ Calcd. for $C_{18}H_{19}BrN_3O_3S$ 436.0325; Found 436.0321.

IR (ATR): $\tilde{\nu}$ [cm^{-1}] = 3070 (w. NH), 2874 (w), 1663 (vs, C=O), 1611 (w, C=C), 1599 (w, C=C), 1456 (m), 1325 (m, RSO_2N), 1163 (vs, RSO_2N), 1051 (m, C–Br), 921 (m), 811 (s, sp^2 C–H), 766 (s, sp^2 C–H), (720 (m), 707 (m), 675 (m).

m.p. > 161 °C (decomposition).

*2.7.9. N'-(3-(5-Nitro-1-oxoisoindolin-2-yl)propylidene)-4-methylbenzenesulfonohydrazide (**2i**)*

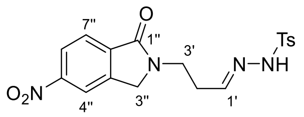

Following GP 7, compound **2i** was obtained as a colorless solid (326 mg, 811 µmol, 86%).

***R*f** (CH_2Cl_2/ac = 9/1): 0.10 [UV, $KMnO_4$].

^{1}H NMR (500 MHz, DMSO-*d*$_6$): δ [ppm] = 2.28 (s, 3H, CH_3), 2.50 – 2.53* (m, 2H, H-2’), 3.65 (t, ^3J = 6.8 Hz, 2H, H-3’), 4.47 (s, 2H, H-3’’), 7.22 – 7.26 (m, 2H, H-3), 7.27 (t, ^3J = 5.1 Hz, 1H, H-1’), 7.58 – 7.62 (m, 2H, H-2), 7.86 (d, ^3J = 8.3 Hz, 1H, H-7’’), 8.32 (dd, ^3J = 8.3 Hz, ^4J = 2.0 Hz, 1H, H-6’’), 8.44 (d, ^4J = 1.7 Hz, 1H, H-4’’), 11.08 (s, 1H, NH).

* signal overlaps with the residual solvent signal.

^{13}C NMR (126 MHz, DMSO-*d*$_6$): δ [ppm] = 165.4 (C-1’’), 149.5 (C-5’’), 149.0 (C-1’), 143.1 (2C, C-4, C-3a’’), 137.7 (C-7a’’), 136.2 (C-1), 129.5 (C-3), 127.0 (C-2), 124.0 (C-7’’), 123.6 (C-6’’), 119.1 (C-4’’), 49.7 (C-3’’), 39.1* (C-3’), 30.9 (C-2’), 20.9 (CH_3).

* signal overlaps with the residual solvent signal.

HRMS (ESI) *m/z*: [M+H]$^+$ Calcd. for $C_{18}H_{19}N_4O_5S$ 403.1071; Found 403.1066.

IR (ATR): $\tilde{\nu}$ [cm^{-1}] = 3098 (w, NH), 2870 (w), 1668 (vs, C=O), 1599 (w, C=C), 1528 (m, NO_2), 1449 (m), 1336 (vs, NO_2), 1323 (s, RSO_2N), 1160 (vs, RSO_2N), 1048 (m), 854 (m, sp^2 C–H), 815 (s, sp^2 C–H), 729 (vs), 707 (s), 666 (m).

m.p. > 207 °C (decomposition).

*2.7.10. N'-(3-(5-Cyano-1-oxoisoindolin-2-yl)propylidene)-4-methylbenzenesulfonohydrazide (**2j**)*

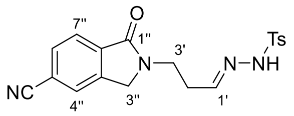

Following GP 7, compound **2j** was obtained as a colorless solid (177 mg, 464 µmol, 46%).

***R*f** (CH_2Cl_2/ac = 9/1): 0.13 [UV, $KMnO_4$].

^{1}H NMR (500 MHz, DMSO-*d*$_6$): δ [ppm] = 2.31 (s, 3H, CH_3), 2.47 – 2.50* (m, 2H, H-2’), 3.63 (t, ^3J = 6.7 Hz, 2H, H-3’), 4.41 (s, 2H, H-3’’), 7.21 – 7.25 (m, 2H, H-3), 7.26 (t, ^3J = 5.1 Hz, 1H, H-1’), 7.57 – 7.61 (m, 2H, H-2), 7.80 (d, ^3J = 7.8 Hz, 1H, H-7’’), 7.95 (dd, ^3J = 7.8 Hz, ^4J = 1.3 Hz, 1H, H-6’’), 8.07 (s, 1H, H-4’’), 11.07 (s, 1H, NH).

* signal overlaps with the residual solvent signal.

^{13}C NMR (126 MHz, DMSO-*d*$_6$): δ [ppm] = 165.8 (C-1’’), 149.1 (C-1’), 143.1 (C-4), 142.5 (C-3a’’), 136.2 (2C, C-1, C-7a’’), 132.1 (C-6’’), 129.5 (C-3), 127.7 (C-4’’), 127.0 (C-2), 123.7 (C-7’’), 118.6 (CN), 113.5 (C-5’’), 49.5 (C-3’’), 38.9 (C-3’), 30.9 (C-2’), 21.0 (CH_3).

HRMS (ESI) *m/z*: [M+H]$^+$ Calcd. for $C_{19}H_{19}N_4O_3S$ 383.1172; Found 383.1172.

IR (ATR): $\tilde{\nu}$ [cm^{-1}] = 3086 (w, NH), 2926 (w), 2876 (w), 2232 (w, C≡N), 1668 (vs, C=O), 1597 (w, C=C), 1459 (m), 1438 (m), 1422 (m), 1359 (m), 1335 (s, RSO_2N), 1316 (m), 1167 (vs, RSO_2N), 1067 (m), 880 (m), 850 (m, sp^2 C–H), 811 (s, sp^2 C–H), 681 (vs), 660 (vs).

m.p. > 185 °C (decomposition).

*2.7.11. Methyl 1-oxo-2-(3-(2-tosylhydrazineylidene)propyl)isoindoline-5-carboxylate (**2k**)*

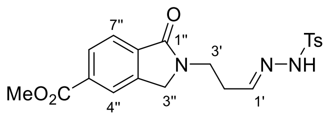

Following GP 7, compound **2k** was obtained as a colorless solid (272 mg, 654 µmol, 85%).

***R*f** (CH_2Cl_2/ac = 9/1): 0.09 [UV, $KMnO_4$].

^{1}H NMR (500 MHz, DMSO-*d*$_6$): δ [ppm] = 2.28 (s, 3H, CH_3), 2.47 – 2.50* (m, 2H, H-2’), 3.64 (t, ^3J = 6.7 Hz, 2H, H-3’), 3.91 (s, 3H, OMe), 4.40 (s, 2H, H-3), 7.20 – 7.24 (m, 2H, H-3’’), 7.27 (t, ^3J = 5.1 Hz, 1H, H-1’), 7.58 – 7.62 (m, 2H, H-2’’), 7.76 (d, ^3J = 7.9 Hz, 1H, H-7), 8.06 (dd, ^3J = 7.9 Hz, ^4J = 1.5 Hz, 1H, H-6), 8.10 (s, 1H, H-4’’), 11.07 (s, 1H, NH).

* signal overlaps with the residual solvent signal.

^{13}C NMR (126 MHz, DMSO-*d*$_6$): δ [ppm] = 166.3 (C-1), 165.9 (*C*OOMe), 149.1 (C-1’), 143.1 (C-4’’), 142.2 (C-3a), 136.3 (2C, C-7a, C-1’’), 132.0 (C-5), 129.5 (C-3’’), 128.9 (C-6), 127.0 (C-2’’), 124.3 (C-4), 123.1 (C-7), 52.6 (OMe), 49.6 (C-3), 38.9 (C-3’), 31.0 (C-2’), 21.0 (CH_3).

HRMS (ESI) *m/z*: $[M+H]^+$ Calcd. for $C_{20}H_{22}N_3O_5S$ 416.1275; Found 416.1270.

IR (ATR): $\tilde{\nu}$ [cm^{-1}] = 3101 (w, NH), 2955 (w), 2922 (w), 1713 (s, COO), 1658 (vs, CON), 1597 (w, C=C), 1441 (s), 1334 (m, RSO_2N), 1321 (m), 1287 (vs, $COOCH_3$), 1204 (s), 1164 (vs, RSO_2N), 1063 (m), 811 (s, sp^2 C–H), 744 (vs, sp^2 C–H), 673 (s).

m.p. > 197 °C (decomposition).

*2.7.12. N'-(3-(4-Methoxy-1-oxoisoindolin-2-yl)propylidene)-4-methylbenzenesulfonohydrazide (**2l**)*

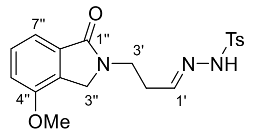

Following GP 7, compound **2l** was obtained as a colorless solid (189 mg, 488 µmol, 49%).

***R*f** (CH_2Cl_2/ac = 9/1): 0.11 [UV, $KMnO_4$].

^{1}H NMR (500 MHz, DMSO-*d*$_6$): δ [ppm] = 2.30 (s, 3H, CH_3), 2.47 (td, ^3J = 6.8 Hz, ^3J = 5.1 Hz, 2H, H-2'), 3.59 (t, ^3J = 6.8 Hz, 2H, H-3'), 3.89 (s, 3H, OMe), 4.28 (s, 2H, H-3''), 7.19 – 7.25 (m, 4H, H-3, H-5'', H-7''), 7.27 (t, ^3J = 5.1 Hz, 1H, H-1'), 7.46 (*virt*. t, $^3J \approx {}^3J$ = 7.8 Hz, 1H, H-6''), 7.57 – 7.61 (m, 2H, H-2), 11.04 (s, 1H, NH).

^{13}C NMR (126 MHz, DMSO-*d*$_6$): δ [ppm] = 167.3 (C-1''), 154.3 (C-4''), 149.3 (C-1'), 143.1 (C-4), 136.2 (C-1), 133.9 (C-7a''), 129.8 (C-6''), 129.5 (C-3), 129.3 (C-3a''), 126.9 (C-2), 114.8 (C-7''), 113.4 (C-5''), 55.6 (OMe), 47.5 (C-3''), 38.9 (C-3'), 31.1 (C-2'), 21.1 (CH_3).

HRMS (ESI) *m/z*: $[M+H]^+$ Calcd. for $C_{19}H_{23}N_3O_4S$ 388.1326; Found 388.1323.

IR (ATR): $\tilde{\nu}$ [cm^{-1}] = 3108 (w, NH), 2941 (w), 2876 (w), 1681 (vs, C=O), 1606 (m C=C), 1496 (m), 1462 (m), 1443 (m), 1330 (m, RSO_2N), 1277 (s, Ar–O–CH_3), 1159 (vs, RSO_2N), 1081 (s), 1058 (s), 902 (m), 819 (m, sp^2 C–H), 807 (m, sp^2 C–H), 748 (vs, sp^2 C–H), 659 (vs).

m.p. > 189 °C (decomposition).

*2.7.13. N'-(3-(4-Bromo-1-oxoisoindolin-2-yl)propylidene)-4-methylbenzenesulfonohydrazide (**2m**)*

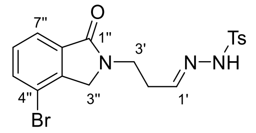

Following GP 7, compound **2m** was obtained as a colorless solid (1.36 g, 3.12 mmol, 77%).

***R*f** (CH_2Cl_2/ac = 9/1): 0.20 [UV, $KMnO_4$].

^{1}H NMR (500 MHz, DMSO-*d*6): δ [ppm] = 2.29 (s, 3H, CH_3), 2.48 – 2.53* (m, 2H, H-2'), 3.63 (t, ^3J = 6.7 Hz, 2H, H-3'), 4.31 (s, 2H, H-3''), 7.18 – 7.22 (m, 2H, H-3), 7.27 (t, ^3J = 5.1 Hz, 1H, H-1'), 7.47 (*virt*. t, $^3J \approx {}^3J$ = 7.7 Hz, 1H, H-6''), 7.56 – 7.60 (m, 2H, H-2), 7.67 (dd, ^3J = 7.7 Hz, ^4J = 0.9 Hz, 1H, H-7''), 7.83 (dd, ^3J = 7.7 Hz, ^4J = 0.9 Hz, 1H, H-5''), 11.06 (s, 1H, NH).

* signal overlaps with the residual solvent signal.

^{13}C NMR (126 MHz, DMSO-*d*6): δ [ppm] = 166.5 (C-1''), 149.2 (C-1'), 143.1 (C-4), 142.0 (C-3a''), 136.2 (C-1), 134.6 (C-7a''), 134.2 (C-5''), 130.3 (C-6''), 129.4 (C-3), 126.9 (C-2), 122.2 (C-7''), 117.2 (C-4''), 50.2 (C-3''), 39.0* (C-3'), 30.9 (C-2'), 21.1 (CH_3).

* signal overlaps with residual solvent signal.

HRMS (ESI) *m/z*: $[M+H]^+$ Calcd. for $C_{18}H_{19}BrN_3O_3S$ 436.0325; Found 436.0319.

IR (ATR): $\tilde{\nu}$ [cm^{-1}] = 3101 (m, NH), 2921 (w), 2873 (w), 1698 (vs, C=O), 1679 (s, C=N), 1614 (w, C=C), 1598 (w, C=C), 1457 (m), 1319 (m, RSO_2N), 1156 (vs, RSO_2N), 1061 (vs, C–Br), 908 (s), 803 (vs, sp^2 C–H), 749 (vs, sp^2 C–H), 664 (vs).

m.p. > 222 °C (decomposition).

*2.7.14. N'-(3-(1-Oxo-4-(trifluoromethyl)isoindolin-2-yl)propylidene)-4-methylbenzenesulfonohydrazide (**2n**)*

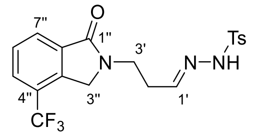

Following GP 7, compound **2n** was obtained as a colorless solid (224 mg, 526 µmol, 75%).

***R*f** (CH_2Cl_2/ac = 9/1): 0.15 [UV, $KMnO_4$].

^{1}H NMR (500 MHz, DMSO-*d*6): δ [ppm] = 2.28 (s, 3H, CH_3), 2.51 – 2.54* (m, 2H, H-2'), 3.64 (t, ^3J = 6.8 Hz, 2H, H-3'), 4.59 (s, 2H, H-3''), 7.17 – 7.21 (m, 2H, H-3), 7.28 (t, ^3J = 5.1 Hz, 1H, H-1'), 7.55 – 7.59 (m, 2H, H-2), 7.74 (*virt*. t, $^3J \approx {}^3J$ = 7.7 Hz, 1H, H-6''), 7.93 – 7.98 (m, 2H, H-5'', H-7''), 11.07 (s, 1H, NH).

* signal overlaps with the residual solvent signal.

^{13}C NMR (126 MHz, DMSO-*d*6): δ [ppm] = 165.8 (C-1''), 149.1 (C-1'), 143.1 (C-4), 138.9 (q, $^3J_{C-F}$ = 2.4 Hz, C-3a''), 136.2 (C 1), 134.0 (C-7a''), 129.4 (C-3), 129.2 (C-6''), 128.31 (q, $^3J_{C-F}$ = 4.1 Hz, C-5''), 127.1 (C-2), 126.9 (C-7''), 124.1 (q, $^2J_{C-F}$ = 32.7 Hz), 123.9 (q, $^1J_{C-F}$ = 273.0 Hz, CF_3), 48.8 (C-3''), 38.9 (C-3'), 30.9 (C-2'), 21.0 (CH_3).

^{19}F NMR (376 MHz, DMSO-*d*6): δ [ppm] = −60.3 (s).

HRMS (ESI) *m/z*: $[M+H]^+$ Calcd. for $C_{19}H_{19}F_3N_3O_3S$ 426.1094; Found 426.1087.

IR (ATR): $\tilde{\nu}$ [cm^{-1}] = 3106 (w, NH), 2948 (w), 2862 (w), 1667 (vs, C=O), 1615 (w, C=C), 1598 (w, C=C), 1466 (m), 1437 (s), 1356 (m), 1326 (vs, RSO_2N), 1158 (vs, RSO_2N), 1125 (vs, C–F), 1112 (vs, C–F), 1054 (s), 1040 (s), 813 (s, sp^2 C–H), 758 (vs, sp^2 C–H), 672 (vs).

m.p. > 203 °C (decomposition).

*2.7.15. N'-(3-(1-Methyl-3-oxoisoindolin-2-yl)propylidene)-4-methylbenzenesulfonohydrazide (**2o**)*

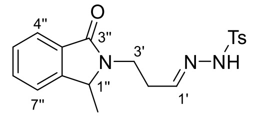

Following GP 7, compound **2o** was obtained as a colorless solid (49.8 mg, 134 µmol, 45%).

***R*f** (CH_2Cl_2/ac = 9/1): 0.34 [UV, $KMnO_4$].

^{1}H NMR (500 MHz, DMSO-*d*6): δ [ppm] = 1.31 (d, ^3J = 6.7 Hz, 3H, C-1''-CH_3), 2.32 (s, 3H, CH_3), 2.37 – 2.49 (m, 2H, H^a-2', H^b-2'), 3.32 – 3.38* (m, 1H, H^a-3'), 3.81 (dt, ^2J = 14.2 Hz, ^3J = 7.1 Hz, 1H, H^b-3'), 4.51 (q, ^3J = 6.7 Hz, 1H, H-1''), 7.22 – 7.25 (m, 2H, H-3), 7.26 (t, ^3J = 5.2 Hz, 1H, H-1'), 7.48 (*virt*. td $^3J \approx {}^3J$ = 7.4 Hz, ^4J = 1.1 Hz, 1H, H-5''), 7.54 – 7.64 (m, 5H, H-2, H-4'', H-6'', H-7''), 11.06 (bs, 1H, NH).

* signal overlaps with residual water signal of the solvent

^{13}C NMR (126 MHz, DMSO-*d*6): δ [ppm] = 166.8 (C-3''), 149.3 (C-1'), 147.3 (C-4), 143.1 (C-7a''), 136.3 (C-1), 131.5 (C-6''), 131.2 (C-3a''), 129.5 (C-3), 128.0 (C-5''), 127.0 (C-2), 122.6 (2C, C-4'', C-7''), 55.2 (C-1''), 36.4 (C-3'), 31.4 (C-2'), 21.1 (CH_3), 18.0 (C-1''-*C*H_3).

HRMS (ESI) *m/z*: $[M+H]^+$ Calcd. for $C_{19}H_{23}N_3O_3S$ 372.1376; Found 372.1371.

IR (ATR): $\tilde{\nu}$ [cm^{-1}] = 3050 (w, NH), 2988 (w), 2878 (w), 2775 (w), 1664 (vs, C=O), 1618 (m, C=C), 1596 (m, C=C), 1470 (m), 1446 (m), 1415 (m), 1361 (m), 1336 (s, RSO_2N), 1182 (m), 1164 (vs, RSO_2N), 1069 (m), 941 (m), 811 (s, sp^2 C–H), 764 (s), 731 (vs, sp^2 C–H), 697 (vs), 662 (vs).

m.p. > 171 °C (decomposition).

*2.7.16. N'-(3-(5-Oxo-5,7-dihydro-6H-pyrrolo[3,4-b]pyridin-6-yl)-4-methyl-propylidene)-4-methylbenzenesulfonohydrazide (**2p**)*

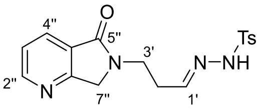

Following GP 7, compound **2p** was obtained as a colorless solid (69.7 mg, 194 µmol, 7%).

***R*f** (CH_2Cl_2/ac = 9/1): 0.30 [UV, $KMnO_4$].

^{1}H NMR (500 MHz, DMSO-d_6): δ [ppm] = 2.31 (s, 3H, CH_3), 2.47 – 2.52* (m, 2H, H-2'), 3.64 (t, ^3J = 6.8 Hz, 2H, H-3'), 4.40 (s, 2H, H-7''), 7.21 – 7.25 (m, 2H, H-3), 7.28 (t, ^3J = 5.1 Hz, 1H, H-1'), 7.51 (dd, ^3J = 7.7 Hz, ^3J = 4.9 Hz, 1H, H-3''), 7.58 – 7.62 (m, 2H, H-3), 8.04 (dd, ^3J = 7.7 Hz, ^4J = 1.6 Hz, 1H, H-4''), 8.76 (dd, ^3J = 4.9 Hz, ^4J = 1.6 Hz, 1H, H-2''), 11.05 (bs, 1H, NH).

* signal overlaps with residual solvent signal

^{13}C NMR (126 MHz, DMSO-d_6): δ [ppm] = 165.8 (C-5''), 162.4 (C-7a''), 152.4 (C-2''), 149.2 (C-1'), 143.2 (C-4), 136.2 (C-1), 131.2 (C-4''), 129.5 (C-3), 127.0 (C-2), 125.7 (C-4a''), 123.3 (C-3''), 51.1 (C-7''), 38.6 (C-3'), 31.0 (C-2'), 21.1 (CH_3).

HRMS (ESI) *m/z*: $[M+H]^+$ Calcd. for $C_{17}H_{19}N_4O_3S$ 359.1172; Found 359.1167.

IR (ATR): $\tilde{\nu}$ [cm^{-1}] = 3043 (w, NH), 2897 (w), 2786 (w), 1657(vs, C=O), 1607 (m, C=C), 1599 (m, C=C), 1581 (w, C=N), 1460 (m), 1441 (m), 1419 (m), 1363 (m), 1325 (s, RSO_2N), 1181 (m), 1163 (vs, RSO_2N), 1052 (m), 958 (m), 920 (m), 876 (m), 807 (s, sp^2 C–H), 758 (vs, sp^2 C–H), 658 (vs).

m.p. > 198 °C (decomposition).

*2.7.17. N'-(4-(1-Oxoisoindolin-2-yl)butylidene)-4-methylbenzenesulfonohydrazide (**2q**)*

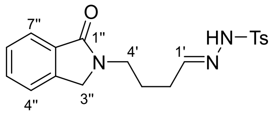

Following GP 7, compound **2q** was obtained as a colorless solid (729 mg, 1.96 mmol, 84%).

***R*f** (CH_2Cl_2/ac = 9/1): 0.12 [UV, $KMnO_4$].

^{1}H NMR (500 MHz, DMSO-d_6): δ [ppm] = 1.68 (*virt.* quint, $^3J \approx {}^3J$ = 7.3 Hz, 2H, H-3'), 2.10 (td, ^3J = 7.3 Hz, ^3J = 5.0 Hz, 2H, H-2'), 2.34 (s, 3H, CH_3), 3.40 (t, ^3J = 7.3 Hz, 2H, H-4'), 4.39 (s, 2H, H-3''), 7.27 (t, ^3J = 5.0 Hz, 1H, H-1'), 7.36 – 7.41 (m, 2H, H-3), 7.48 (ddd, ^3J = 7.8 Hz, ^3J = 6.6 Hz, ^4J = 1.9 Hz, 1H, H-6''), 7.55 – 7.62 (m, 2H, H-4'', H-5''), 7.66 (*virt.* dt, ^3J = 7.8 Hz, $^4J \approx {}^5J$ = 1.0 Hz, 1H, H-7''), 7.67 – 7.71 (m, 2H, H-2), 10.93 (bs, 1H, NH).

^{13}C NMR (126 MHz, DMSO-d_6): δ [ppm] = 167.3 (C-1''), 151.1 (C-1'), 143.3 (C-4), 141.9 (C-3a''), 136.2 (C-1), 132.4 (C-7a''), 131.3 (C-5''), 129.6 (C-3), 127.8 (C-6''), 127.2 (C-2), 123.4 (C-4''), 122.7 (C-7), 49.5 (C-3''), 41.1 (C-4'), 29.3 (C-2'), 24.5 (C-3'), 21.0 (CH_3).

HRMS (ESI) *m/z*: $[M+H]^+$ Calcd. for $C_{19}H_{22}N_3O_3S$ 372.1376; Found 372.1384.

IR (ATR): $\tilde{\nu}$ [cm^{-1}] = 3029 (w, NH), 2911 (w), 2866 (w), 2771 (w), 1660 (vs, C=O), 1618 (w, C=C), 1596 (w, C=C), 1478 (m), 1459 (m), 1426 (m), 1360 (m), 1331 (s, RSO_2N), 1260 (m), 1133 (vs, RSO_2N), 1063 (m), 806 (s, sp^2 C–H), 771 (s), 736 (vs, sp^2 C–H), 663 (vs).

m.p. > 173 °C (decomposition).

*2.7.18. N'-(2-((1-Oxoisoindolin-2-yl)methyl)benzylidene)-4-methylbenzenesulfonohydrazide (**2r**)*

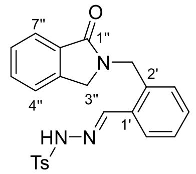

Following GP 7, compound **2r** was obtained as a colorless solid (230 mg, 548 µmol, 69%).

***R*f** (CH_2Cl_2/ac = 9/1): 0.41 [UV, $KMnO_4$].

^{1}H NMR (500 MHz, DMSO-*d6*): δ [ppm] = 2.29 (s, 3H, CH_3), 4.26 (s, 2H, C-3''), 4.77 (s, 2H, CH_2), 7.19 (dd, ^3J = 7.2 Hz, ^4J = 1.8 Hz, 1H, H-3'), 7.30 – 7.39 (m, 4H, H-3, H-4', H-5'), 7.49 – 7.55 (m, 2H, H-4'', H-6''), 7.58 – 7.63 (m, 2H, H-6', H-5''), 7.72 – 7.82 (m, 3H, H-2, C-7''), 8.24 (s, 1H, CHN), 11.57 (s, 1H, NH).

^{13}C NMR (126 MHz, DMSO-*d6*): δ [ppm] = 167.3 (C-1''), 146.1 (CHN), 143.6 (C-4), 141.9 (C-3a''), 136.1 (C-1), 135.7 (C-2'), 131.9 (C-7a''), 131.6 (C-5''), 131.5 (C-1'), 130.1 (C-4'), 129.7 (C-3), 128.9 (C-3'), 128.0 (C-6''), 127.8 (2C, C-5', C-6'), 127.4 (C-2), 123.6 (C-4''), 123.0 (C-7''), 49.4 (C-3''), 43.2 (CH_2), 21.0 (CH_3).

HRMS (ESI) *m/z*: $[M+H]^+$ Calcd. for $C_{23}H_{22}N_3O_3S$ 420.1376; Found 420.1375.

IR (ATR): $\tilde{\nu}$ [cm^{-1}] = 3606(w), 3454 (w), 3057 (w, NH), 2982 (w), 2883 (w), 2776 (w), 1659 (s, C=O), 1619 (w, C=C), 1597 (w, C=C), 1472 (m), 1455 (m), 1417 (m), 1330 (s, RSO_2N), 1305 (m), 1265 (m), 1134 (vs, RSO_2N), 1056 (m), 948 (m), 814 (m, sp^2 C–H), 731 (vs, sp^2 C–H), 702 (s), 664 (vs).

m.p. > 192 °C (decomposition).

*2.7.19. N'-(2-(2-(1-Oxoisoindolin-2-yl)ethyl)benzylidene)-4-methylbenzenesulfonohydrazide (**2s**)*

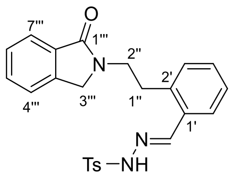

Following GP 7, compound **2s** was obtained as a colorless solid (500 mg, 1.15 mmol, 93%).

***R*f** (CH_2Cl_2/ac = 9/1): 0.36 [UV, $KMnO_4$].

^{1}H NMR (500 MHz, DMSO-*d6*): δ [ppm] = 2.33 (s, 3H, CH_3), 3.02 (t, ^3J = 7.6 Hz, 2H, H-1''), 3.62 (t, ^3J = 7.6 Hz, 2H, H-2''), 4.34 (s, 2H, C-3''), 7.20 (dd, ^3J = 7.3 Hz, ^4J = 1.9 Hz, 1H, H-3'), 7.24 (*virt*. td, $^3J \approx {}^3J$ = 7.3 Hz, ^4J = 1.9 Hz, 1H, H-5') 7.27 (*virt*. td, $^3J \approx {}^3J$ = 7.3 Hz, ^4J = 1.7 Hz, 1H, H-4'), 7.37 – 7.41 (m, 2H, H-3), 7.48 (*virt*. td, $^3J \approx {}^3J$ = 7.4 Hz, ^4J = 1.3 Hz, 1H, H-6'''), 7.55 (d, ^3J = 7.4 Hz, 1H, H-4'''), 7.57 – 7.61 (m, 2H, H-6', H-5'''), 7.65 (*virt*. dt, ^3J = 7.4 Hz, $^4J \approx {}^5J$ = 1.0 Hz, 1H, H-7'''), 7.74 – 7.78 (m, 2H, H-2), 8.25 (s, 1H, CHN), 11.49 (s, 1H, NH).

^{13}C NMR (126 MHz, DMSO-*d6*): δ [ppm] = 167.2 (C-1'''), 146.0 (CHN), 143.5 (C-4), 141.8 (C-3a''), 137.8 (C-2'), 136.2 (C-1), 132.3 (C-7a'''), 131.6 (C-1'), 131.3 (C-5'''), 130.6 (C-3'), 129.9 (C-4'), 129.8 (C-3), 127.9 (C-6'''), 127.3 (C-2), 127.1 (C-6'), 127.0 (C-5'), 123.4 (C-4'''), 122.7 (C-7'''), 49.8 (C-3'''), 42.9 (C-2''), 31.2 (C-1''), 21.0 (CH_3).

HRMS (ESI) *m/z*: $[M+H]^+$ Calcd. for $C_{24}H_{24}N_3O_3S$ 434.1533; Found 434.1529.

IR (ATR): $\tilde{\nu}$ [cm^{-1}] = 3548 (w), 3377 (w), 3026 (w), 2884 (w), 1667 (s, C=O), 1623 (w, C=C), 1596 (w, C=C), 1474 (m), 1455 (m), 1419 (m), 1328 (s, RSO_2N), 1160 (vs, RSO_2N), 1093 (m), 1045 (m), 963 (m), 817 (s, sp^2 C–H), 727 (vs, sp^2 C–H), 664(vs).

m.p. > 192 °C (decomposition).

*2.7.20. N'-(3-(1-Oxoisoindolin-2-yl)butylidene)-4-methylbenzenesulfonohydrazide (**2u**)*

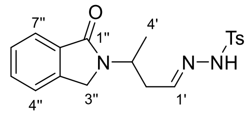

Following GP 7, compound **2u** was obtained as a colorless solid (331 mg, 891 µmol, 48%).

***R*f** (CH_2Cl_2/ac = 9/1): 0.13 [UV, $KMnO_4$].

^{1}H NMR (500 MHz, DMSO-*d*6): δ [ppm] = 1.13 (t, ^3J = 6.6 Hz, 3H, H-4'), 2.30 (s, 3H, CH_3), 2.45 – 2.49 (m, 2H, H-2'), 4.22 (d, ^2J = 17.6 Hz, 1H, H^a-3''), 4.36 (d, ^2J = 17.6 Hz, 1H, H^b-3''), 4.44 (tq, ^3J = 8.5 Hz, ^3J = 6.6 Hz, 1H, H-3'), 7.16 – 7.20 (m, 3H, H-3, H-1'), 7.48 (*virt*. td, $^3J \approx {}^3J$ = 7.4 Hz, ^4J = 1.1 Hz, 1H, H-6''), 7.52 – 7.57 (m, 3H, H-2, H-4''), 7.85 – 7.65 (m, 2H, H-5'', H-7''), 10.98 (s, 1H, NH).

^{13}C NMR (126 MHz, DMSO-*d*6): δ [ppm] = 167.0 (C-1''), 148.9 (C-1'), 143.1 (C-4), 141.9 (C-3a''), 136.2 (C-1), 132.4 (C-7a''), 131.2 (C-5''), 129.5 (C-3), 127.8 (C-6''), 126.9 (C-2), 123.5 (C-4''), 122.7 (C-7''), 45.3 (C-3''), 44.6 (C-3'), 37.2 (C-2'), 21.1 (CH_3), 18.6 (C-4').

HRMS (ESI) *m/z*: $[M+H]^+$ Calcd. for $C_{19}H_{22}N_3O_3S$ 372.1376; Found 372.1371.

IR (ATR): $\tilde{\nu}$ [cm^{-1}] = 3059 (m, NH), 2974 (w), 2876 (w), 2771 (w), 1655 (vs, C=O), 1597 (w, C=C), 1471 (m), 1451 (s), 1412 (m), 1342 (m), 1323 (s, RSO_2N), 1161 (vs, RSO_2N), 1064 (m), 916 (m), 814 (m), 736 (vs, sp^2 C–H), 663 (vs).

m.p. > 100 °C (decomposition).

*2.7.21. N'-(3-((tert-Butyldimethylsilyl)oxy)-4-(1-oxoisoindolin-2-yl)butylidene)-4-methylbenzenesulfonohydrazide (**2v**)*

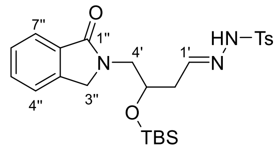

Following GP 7, compound **2v** was obtained after flash column chromatography (CH_2Cl_2/ac = 9/1) as a colorless solid (77.6 mg, 155 µmol, 21%).

***R*f** (CH_2Cl_2/ac = 9/1): 0.37 [UV, $KMnO_4$].

^{1}H NMR (500 MHz, DMSO-*d*6): δ [ppm] = −0.15 (s, 3H, $SiCH_3^a$), −0.08 (s, 3H, $SiCH_3^b$), 0.76 [s, 9H, $(CH_3)_3$], 2.17 (*virt*. dt, ^2J = 14.4 Hz, $^3J \approx {}^3J$ = 6.0 Hz, 1H, H^a-2'), 2.29 (*virt*. dt, ^2J = 14.4 Hz, $^3J \approx {}^3J$ = 6.0 Hz, 1H, H^b-2'), 2.33 (s, 3H, CH_3), 3.31 (dd, ^2J = 13.9 Hz, ^3J = 4.9 Hz, 1H, H^a-4'), 3.49 (dd, ^2J = 13.9 Hz, ^3J = 6.0 Hz, 1H, H^b-4'), 4.13 (*virt* qd, $^3J \approx {}^3J \approx {}^3J$ = 6.0 Hz, ^3J = 4.9 Hz, 1H, H-3'), 4.39 (d, ^2J = 17.6 Hz, 1H, H^a-3''), 4.49 (d, ^2J = 17.6 Hz, 1H, H^b-3''), 7.27 (t, ^3J = 6.0 Hz, 1H, H-1'), 7.35 – 7.40 (m, 2H, H-3), 7.48 (ddd, ^3J = 7.9 Hz, ^3J = 5.8 Hz, ^4J = 2.6 Hz, 1H, H-6''), 7.56 – 7.62 (m, 2H, H-4'', H-5''), 7.64 – 7.71 (m, 3H, H-2, H-7''), 11.09 (s, 1H, NH).

^{13}C NMR (126 MHz, DMSO-*d*6): δ [ppm] = 167.7 (C-1''), 148.6 (C-1'), 143.2 (C-4), 141.9 (C-3a''), 136.3 (C-1), 132.2 (C-7a''), 131.4 (C-5''), 129.6 (C-3), 127.9 (C-6''), 127.2 (C-2), 123.3 (C-4''), 122.7 (C-7''), 68.6 (C-3'), 51.2 (C-3''), 47.9 (C-4'), 37.9 (C-2'), 25.7 ([C(*C*H3)3], 21.0 (CH_3), 17.5 [*C*[CH3]3], −5.01 ($SiCH_3^b$), −5.03 ($SiCH_3^a$).

HRMS (ESI) *m/z*: $[M+H]^+$ Calcd. for $C_{25}H_{36}N_3O_4SSi$ 502.2190; Found 502.2185.

IR (ATR): $\tilde{\nu}$ [cm^{-1}] = 3066 (w, NH), 2954 (w), 2928 (w), 2885 (w), 2857 (w), 1664 (vs, C=O), 1621 (w, C=C), 1597 (w, C=C), 1472 (m), 1456 (m), 1415 (m), 1325 (m, RSO_2N), 1305 (m), 1253 (m), 1163 (vs, RSO_2N), 1088 (m), 865 (m), 834 (vs, sp^2 C–H), 813 (s), 773 (s), 734 (vs, sp^2 C–H), 664 (vs).

m.p. > 123 °C (decomposition).

*2.7.22. N'-(3-((4-Methoxybenzyl)oxy)-4-(1-oxoisoindolin-2-yl)butylidene)-4-methylbenzenesulfonohydrazide (**2w**)*

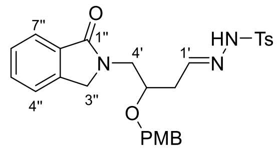

Following GP 7, compound **2w** was obtained as a parchment white solid (296 mg, 582 µmol, 64%).

***R*f** (CH_2Cl_2/ac = 9/1): 0.14 [UV, $KMnO_4$].

^{1}H NMR (500 MHz, DMSO-d_6): δ [ppm] = 2.25 – 2.33 (m, 4H, CH_3, H^a-2'), 2.40 (*virt*. dt, ^2J = 14.9 Hz, ^3J ≈ ^3J = 4.9 Hz, 1H, H^b-2'), 3.44 (dd, ^2J = 14.3 Hz, ^3J = 4.9 Hz, 1H, H^a-4'), 3.51 (dd, ^2J = 14.3 Hz, ^3J = 6.7 Hz, 1H, H^b-4'), 3.69 (s, 3H, OMe), 3.81 (*virt* tt, ^3J ≈ ^3J = 6.7 Hz, ^3J ≈ ^3J = 4.9 Hz, 1H, H-3'), 4.28 (d, ^2J = 11.4 Hz, 1H, OC*H*H), 4.33 (d, ^2J = 11.4 Hz, 1H, OCH*H*), 4.34 (d, ^2J = 17.8 Hz, 1H, H^a-3''), 4.38 (d, ^2J = 17.8 Hz, 1H, H^b-3''), 6.71 – 6.76 (m, 2H, H-3'''), 7.03 – 7.07 (m, 2H, H-2'''), 7.30 – 7.36 (m, 3H, H-3, H-1'), 7.48 (*virt*. td, ^3J ≈ ^3J = 7.5 Hz, ^4J = 1.1 Hz, 1H, H-6''), 7.53 (d, ^3J = 7.5 Hz, 1H, H-4''), 7.59 (*virt*. td, ^3J ≈ ^3J = 7.5 Hz, ^4J = 1.1 Hz, 1H, H-5''), 7.66 (*virt*. dt, ^3J = 7.5Hz, ^4J ≈ ^5J = 1.1 Hz, 1H, H-7''), 7.67 – 7.71 (m, 2H, H-2), 11.06 (s, 1H, NH).

^{13}C NMR (126 MHz, DMSO-d_6): δ [ppm] = 168.0 (C-1''), 159.1 (C-4'''), 149.3 (C-1'), 143.7 (C-4), 142.5 (C-3a''), 136.7 (C-1), 132.5 (C-7a''), 131.8 (C-5''), 130.5 (C-1'''), 130.1 (C-3), 129.7 (C-2'''), 128.2 (C-6''), 127.7 (C-2), 123.7 (C-4''), 123.2 (C-7''), 113.9 (C-3'''), 74.9 (C-3'), 70.4 (OCH_2), 55.4 (OMe), 51.3 (C-3''), 45.3 (C-4'), 35.4 (C-2'), 21.4 (CH_3).

HRMS (ESI) *m/z*: [M+Na]$^+$ Calcd. for $C_{27}H_{29}N_3O_5SNa$ 530.1720; Found 530.1716.

IR (ATR): $\tilde{\nu}$ [cm^{-1}] = 3022 (w), 2868 (w), 2836 (w), 2768 (w), 1661 (vs, C=O), 1612 (w, C=C)m 1595 (w, C=C), 1513 (m), 1474 (m), 1456 (m), 1360 (m), 1338 (m, RSO_2N), 1256 (m), 1167 (vs, RSO_2N), 1065 (vs, COMe), 839 (m, sp^2 C–H), 804 (s, sp^2 C–H), 738 (vs, sp^2 C–H), 662 (vs).

m.p. > 182 °C (decomposition).

*2.7.23. N'-(2-Methyl-4-(1-oxoisoindolin-2-yl)butylidene)-4-methylbenzenesulfonohydrazide (**2x**)*

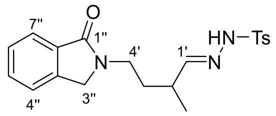

Following GP 7, compound **2x** was obtained as a colorless solid (227 mg, 589 µmol, 71%).

***R*f** (CH_2Cl_2/ac = 9/1): 0.35 [UV, $KMnO_4$].

¹H NMR (500 MHz, DMSO-*d*6): δ [ppm] = 0.95 (d, ^3J = 6.9 Hz, 3H, C-2'-CH_3), 1.53 (ddt, ^2J = 13.6 Hz, ^3J = 8.4 Hz, ^3J = 6.3 Hz, 1H, H^a-3'), 1.68 (ddt, ^2J = 13.6 Hz, ^3J = 8.4 Hz, ^3J = 6.8 Hz, 1H, H^b-3'), 2.20 – 2.27 (m, 1H, H-2'), 2.31 (s, 3H, CH_3), 3.25 – 3.21* (m, 2H, H-4'), 4.31 (d, ^3J = 17.7 Hz, 1H, H^a-3''), 4.35 (d, ^3J = 17.7 Hz, 1H, H^b-3''), 7.19 (d, ^3J = 5.5 Hz, 1H, H-1'), 7.36 – 7.40 (m, 2H, H-3), 7.47 (*virt*. td, $^3J \approx {}^3J$ = 7.6 Hz, ^4J = 1.5 Hz, 1H, H-6''), 7.56 (ddd, ^3J = 6.9 Hz, ^4J = 1.5 Hz, ^5J =1.1 Hz, 1H, H-4''), 7.59 (ddd, ^3J = 7.6 Hz, ^3J = 6.9 Hz, ^4J = 1.1 Hz, 1H, H-5''), 7.65 (*virt*. dt, ^3J = 7.6 Hz, $^4J \approx {}^5J$ = 1.1 Hz, 1H, H-7''), 7.67 – 7.71 (m, 2H, H-2), 10.88 (bs, 1H, NH).

* signal overlaps with residual water signal of the solvent

¹³C NMR (126 MHz, DMSO-*d*6): δ [ppm] = 167.2 (C-1''), 155.0 (C-1'), 143.3 (C-4), 141.9 (C-3a''), 136.1 (C-1), 132.4 (C-7a''), 131.3 (C-5''), 129.6 (C-3), 127.9 (C-6''), 127.3 (C-2), 123.4 (C-4''), 122.7 (C-7''), 49.4 (C-3''), 39.6* (C-4'), 33.9 (C2'), 31.9 (C-3'), 21.0 (CH_3), 17.3 (C-2'-*C*H_3).

* signal overlaps with residual solvent signal

HRMS (ESI) *m/z*: $[M+H]^+$ Calcd. for $C_{20}H_{24}N_3O_3S$ 386.1533; Found 386.1536.

IR (ATR): $\tilde{\nu}$ [cm^{-1}] = 3035 (w, NH), 2967 (w), 2872 (w), 2776 (w), 1659 (vs, C=O), 1622 (m, C=C), 1596 (m, C=C), 1477 (m), 1457 (m), 1423 (m), 1361 (m), 1333 (s, RSO_2N), 1181 (m), 1167 (vs, RSO_2N), 1042 (m), 811 (s, sp^2 C–H), 772 (s), 734 (vs, sp^2 C–H), 662 (vs).

m.p. > 138 °C (decomposition).

2.8. Procedures for individual substrates

*2.8.1. 2-Hydroxy-4-methoxy-4-oxobutan-1-aminium chloride (**SI-5**)*

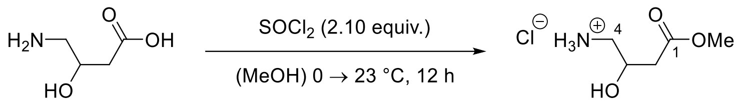

Based on a modified procedure by *Ulgheri et al.,*[9] $SOCl_2$ (4.57 mL, 7.49 g, 63.0 mmol, 2.10 equiv.) was added over 40 min dropwise to a suspension of 4-amino-3-hydroxybutanoic acid (3.57 g, 30.0 mmol, 1.00 equiv.) in MeOH (39 ml, 775 mM) at 0 °C. After the addition, the cooling bath was removed and the reaction mixture was stirred at r.t. for 12 h. The solvent was removed under reduced pressure to yield the entitled ester **SI-5** (5.10 g, 30.0 mmol, *quant.*) as a colorless oil.

^{1}H NMR (500 MHz, MeOD): δ [ppm] = 2.56 (dd, ^2J = 16.0 Hz, ^3J = 7.2 Hz, 1H, H^a-2), 2.60 (dd, ^2J = 16.0 Hz, ^3J = 5.6 Hz, 1H, H^b-2), 2.90 (dd, ^2J = 12.8 Hz, ^3J = 9.2 Hz, 1H, H^a-4), 3.12 (dd, ^2J = 12.8 Hz, ^3J = 3.2 Hz, 1H, H^b-4), 3.70 (s, 3H, OMe), 4.22 (dddd, ^3J = 9.2 Hz, ^3J = 7.2 Hz, ^3J = 5.6 Hz, ^3J = 3.2 Hz, 1H, H-3).

^{13}C NMR (126 MHz, MeOD): δ [ppm] = 172.6 (C-1), 65.6 (C-3), 52.3 (OMe), 45.3 (C-4), 40.5 (C-2).

Spectral data matched those reported in the literature.[9]

*2.8.2. Methyl 3-hydroxy-4-(1-oxoisoindolin-2-yl)butanoate (**SI-6**)*

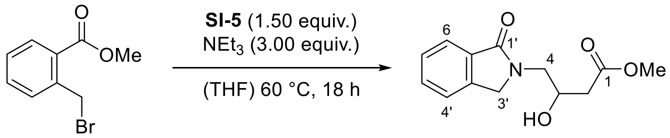

Following GP 4 using the amine **SI-5** as substrate, compound **SI-6** was obtained as a white solid (1.96 g, 7.85 mmol, 79%).

***R*f** (Hex/EtOAc = 1/4): 0.10 [UV, $KMnO_4$].

^{1}H NMR (500 MHz, $CDCl_3$): δ [ppm] = 2.55 (dd, ^2J = 16.7 Hz, ^3J = 8.5 Hz, 1H, H^a-2), 2.63 (dd, ^2J = 16.7 Hz, ^3J = 4.2 Hz, 1H, H^b-2), 3.70 (s, 3H, OMe), 3.71 (dd, ^2J = 14.4 Hz, ^3J = 6.4 Hz, 1H, H^a-4), 3.77 (dd, ^2J = 14.4 Hz, ^3J = 3.4 Hz, 1H, H^b-4), 4.35 (dddd, ^3J = 8.5 Hz, ^3J = 6.4 Hz, ^3J = 4.2 Hz, ^3J = 3.4 Hz, 1H, H-3), 4.59 (s, 2H, H-3’), 7.43 – 7.49 (m, 2H, H-4’, H-6’), 7.55 (*virt*. td, $^3J \approx ^3J$ = 7.5 Hz, ^4J = 1.1 Hz, 1H, H-5’), 7.85 (dd, ^3J = 7.7 Hz, ^4J = 1.1 Hz, 1H, H-7’).

^{13}C NMR (126 MHz, $CDCl_3$): δ [ppm] = 173.1 (C-1), 169.8 (C-1’), 141.9 (C-7a’), 132.3 (C-3a’), 131.7 (C-5’), 128.2 (C-6’), 123.9 (C-7’), 122.8 (C-4’), 68.2 (C-3), 52.5 (C-3’), 52.1 (OMe), 48.3 (C-4), 38.7 (C-2).

HRMS (ESI) *m/z*: $[M+H]^+$ Calcd. for $C_{13}H_{16}NO_4$ 250.1074; Found 250.1073.

IR (ATR): $\tilde{\nu}$ [cm^{-1}] = 3341 (m, OH), 2957 (w), 2922 (w), 2848 (w), 1729 (s, COO), 1663 (vs, CON), 1620 (m (C=C), 1594 (w, C=C), 1474 (m), 1440 (m), 1345 (s), 1270 (s), 1141 (s), 996 (m), 860 (m), 771 (m), 739 (vs, sp^2 C–H), 681 (m).

m.p. = 121-124 °C.

*2.8.3. Methyl 3-((tert-butyldimethylsilyl)oxy)-4-(1-oxoisoindolin-2-yl)butanoate (**SI-7a**)*

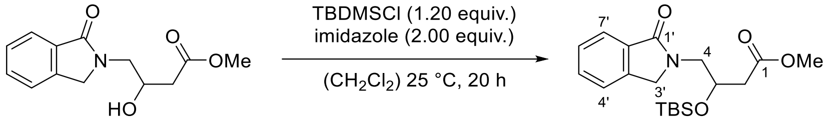

Based on a modified procedure by *Kumar et al.*,[10] TBSCl (181 mg, 1.20 mmol, 1.20 equiv.) was added to a solution of alcohol **SI-6** (249 mg, 1.00 mmol, 1.00 equiv.) and imidazole (136 mg, 2.00 mmol, 2.00 equiv.) in CH_2Cl_2 (2.0 mL, 500 mM) and the mixture was stirred at 25 °C for 20 h. The reaction mixture was diluted with CH_2Cl_2 (20 mL) and quenched by addition of H_2O (20 mL). The organic layer was separated and the aqueous layer was extracted with CH_2Cl_2 (2 × 20 mL). The combined organic layers were washed with brine (50 mL), dried over Na_2SO_4 and filtered. After removal of the solvent under reduced pressure, the crude product was purified by automated flash column chromatography (Hex/EtOAc) yielding the silyl ether **SI-7a** (355 mg, 976 μmol, 98%) as a colorless oil.

***R*f** (Hex/EtOAc = 3/2): 0.40 [UV, $KMnO_4$].

^{1}H NMR (500 MHz, $CDCl_3$): δ [ppm] = 0.06 (s, 3H, $SiCH_3^a$), 0.07 (s, 3H, $SiCH_3^b$), 0.88 [s, 9H, $SiC(CH_3)_3$], 2.45 (dd, ^2J = 15.3 Hz, ^3J = 7.2 Hz, 1H, H^a-2), 2.57 (dd, ^2J = 15.5 Hz, ^3J = 4.7 Hz, 1H, H^b-2), 3.52 (dd, ^2J = 14.1 Hz, ^3J = 4.7 Hz, 1H, H^a-4), 3.63 (s, 3H, OMe), 3.93 (dd, ^2J = 14.1 Hz, ^3J = 4.7 Hz, 1H, H^b-4), 4.42 (d, ^2J = 17.1 Hz, 1H, H^a-3'), 4.51 (*virt.* dq, ^3J = 7.2 Hz, $^3J \approx {}^3J \approx {}^3J$ = 4.7 Hz, 1H, H-2), 4.64 (d, ^2J = 17.1 Hz, 1H, H^b-3'), 7.42 – 7.48 (m, 2H, H-4, H-6), 7.54 (*virt.* td, $^3J \approx {}^3J$ = 7.5 Hz, ^4J = 1.1 Hz, 1H, H-5), 7.84 (*virt.* dt, ^3J = 7.4 Hz, $^4J \approx {}^5J$ = 1.1 Hz, 1H, H-7).

^{13}C NMR (126 MHz, $CDCl_3$): δ [ppm] = 171.8 (C-1), 169.1 (C-1'), 141.7 (C-7a'), 132.5 (C-3a'), 131.5 (C-5'), 128.1 (C-6'), 123.9 (C-7'), 122.8 (C-4'), 68.9 (C-3), 52.3 (C-3'), 51.8 (OMe), 48.6 (C-4), 40.3 (C-2), 25.8 [SiC(*C*H$_3$)$_3$], 18.0 [Si*C*(CH$_3$)$_3$], −4.50 ($CiCH_3^b$), −4.99 ($SiCH_3^a$).

HRMS (ESI) *m/z*: [M+H]$^+$ Calcd. for $C_{19}H_{30}NO_4Si$ 364.1939; Found 364.1934.

IR (ATR): $\tilde{\nu}$ [cm^{-1}] = 2953 (w), 2930 (w), 2895 (w), 2857 (w), 1737 (s, COO), 1691 (vs, CON), 1621 (w, C=C), 1471 (m), 1409 (m), 1254 (s), 1082 (vs, CO), 1005 (m), 828 (vs, sp^2 C–H), 810 (s, sp^2 C–H), 777 (vs, sp^2 C–H), 734 (vs, sp^2 C–H).

*2.8.4. Methyl 3-((4-methoxybenzyl)oxy)-4-(1-oxoisoindolin-2-yl)butanoate (**SI-7b**)*

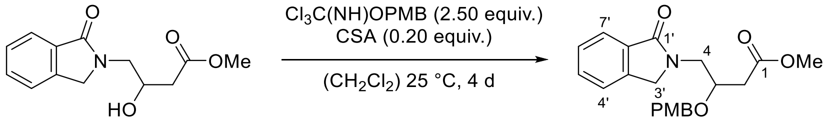

Based on a modified procedure by *Wu et al.*,[11] 4-methoxybenzyl-2,2,2-trichloroacetimidate (0.48 mL, 651 mg, 2.30 mmol, 2.50 equiv.) was added to a solution of alcohol **SI-6** (230 mg, 922 µmol, 1.00 equiv.) and CSA (42.8 mg, 184 µmol, 0.20 equiv.) in CH_2Cl_2 (2.6 mL, 350 mM) and the mixture was stirred at 25 °C for 4 d. The reaction mixture was quenched by addition of sat. $NaHCO_3$ solution (20 mL) and diluted with CH_2Cl_2 (20 mL). The organic layer was separated and the aqueous layer was extracted with CH_2Cl_2 (2 × 20 mL). The combined organic layers were washed with brine (50 mL), dried over Na_2SO_4 and filtered. After removal of the solvent under reduced pressure, the crude product was purified by automated flash column chromatography (Hex/EtOAc) yielding the PMB ether **SI-7b** (320 mg, 867 µmol, 94%) as a colorless oil.

***R*f** (Hex/EtOAc = 2/3): 0.27 [UV, $KMnO_4$].

^{1}H NMR (500 MHz, $CDCl_3$): δ [ppm] = 2.59 (dd, ^2J = 15.8 Hz, ^3J = 7.1 Hz, 1H, H^a-2), 2.64 (dd, ^2J = 15.8 Hz, ^3J = 5.6 Hz, 1H, H^b-2), 3.67 (s, 3H, COOMe), 3.69 (dd, ^2J = 14.4 Hz, ^3J = 3.9 Hz, 1H, H^a-4), 3.76 (s, 3H, OMe), 3.81 (dd, ^2J = 14.4 Hz, ^3J = 5.6 Hz, 1H, H^b-4), 4.24 (*virt*. dtd, ^3J = 7.1 Hz, ^3J ≈ ^3J = 5.4 Hz, ^3J = 3.9 Hz, 1H, H-2), 4.39 (d, ^2J = 17.1 Hz, 1H, H^a-3'), 4.46 (d, ^2J = 17.1 Hz, 1H, H^b-3'), 4.46 (d, ^2J = 11.2 Hz, 1H, OC*H*H), 4.57 (d, ^2J = 11.2 Hz, 1H, OCH*H*), 6.76 – 6.80 (m, 2H, H-3''), 7.15 – 7.20 (m, 2H, H-2''), 7.39 (*virt*. dt, ^3J = 7.5 Hz, ^4J ≈ ^5J = 1.0 Hz, 1H, H-4'), 7.45 (*virt*. td, ^3J ≈ ^3J = 7.5 Hz, ^4J = 1.0 Hz, 1H, H-6), 7.52 (*virt*. td, ^3J ≈ ^3J = 7.5 Hz, ^4J = 1.0 Hz, 1H, H-5), 7.84 (*virt*. dt, ^3J = 7.5 Hz, ^4J ≈ ^5J = 1.0 Hz, 1H, H-7).

^{13}C NMR (126 MHz, $CDCl_3$): δ [ppm] = 171.8 (C-1), 169.1 (C-1'), 159.4 (C-4''), 141.8 (C-7a'), 132.5 (C-3a'), 131.5 (C-5'), 130.1 (C-1''), 129.6 (C-2''), 128.0 (C-6), 123.8 (C-7), 122.7 (C-4), 113.9 (C-3''), 74.9 (C-3), 72.0 (OCH_2), 55.4 (OMe), 52.0 (C-3'), 51.9 (COOMe), 45.8 (C-4), 37.8 (C-2).

HRMS (ESI) *m/z*: $[M+H]^+$ Calcd. for $C_{21}H_{24}NO_5$ 370.1649; Found 370.1645.

IR (ATR): $\tilde{\nu}$ [cm^{-1}] = 2951 (w), 2838 (w), 1735(s, COO), 1683 (vs, CON), 1612 (, C=C), 1586 (w, C=C), 1513 (s), 1455 (m), 1412 (m), 1302 (m), 1246 (vs, ArOMe), 1173 (s), 1073 (s, CO), 1029 (vs, ArOMe), 821 (m, sp^2 C–H), 735 (vs, sp^2 C–H), 684 (m).

*2.8.5. 3-((tert-Butyldimethylsilyl)oxy)-4-(1-oxoisoindolin-2-yl)butanal (**SI-8a**)*

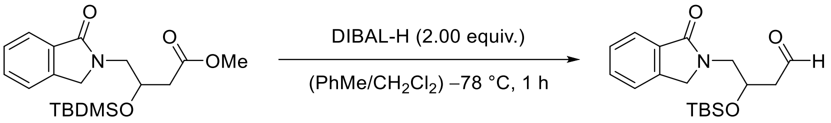

Based on a modified procedure by *Jiang et al.*,[12] DIBAL-H (1 M in PhMe, 1.00 mL, 1.00 mmol, 2.00 equiv.) was added dropwise to a solution of ester **SI-7a** (175 mg, 481 µmol, 1.00 equiv.) in CH_2Cl_2 (1.2 mL, 0.4 M) at −78 °C. The reaction mixture was kept at that temperature for 1 h and afterwards quenched with MeOH (1.0 mL) at −78 °C. After warming to room temperature, the suspension was treated with sat. *Rochelle salt* solution (15 mL) and stirred vigorously for 30 min. The aqueous layer was extracted with EtOAc (3 × 20 mL). The combined organic layers were washed with H_2O (30 mL) and brine (30 mL) and dried over Na_2SO_4. The solution was filtered over a short silica plug (↕ 5 cm, ⌀ 4 cm) and eluted with EtOAc (200 mL). The filtrate was concentrated under reduced pressure to obtain the entitled aldehyde **SI-8a** (99.4 mg, 298 µmol, 62%), which was used for the formation of its hydrazone without further purification and analysis.

*2.8.6. 3-((4-Methoxybenzyl)oxy)-4-(1-oxoisoindolin-2-yl)butanal (**SI-8b**)*

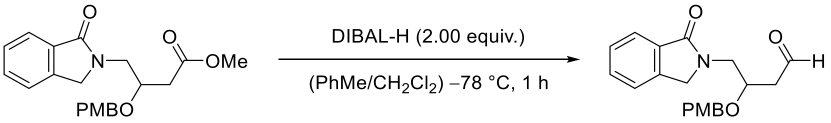

Based on a modified procedure by *Jiang et al.*,[12] DIBAL-H (1 M in PhMe, 3.14 mL, 3.14 mmol, 2.00 equiv.) was added dropwise to a solution of ester **SI-7b** (580 mg, 1.57 mmol, 1.00 equiv.) in CH_2Cl_2 (3.9 mL, 0.4 M) at −78 °C. The reaction mixture was kept at that temperature for 1 h and afterwards quenched with MeOH (1.0 mL) at −78 °C. After warming to room temperature, the suspension was treated with sat. *Rochelle salt* solution (15 mL) and stirred vigorously for 30 min. The aqueous layer was extracted with EtOAc (3 × 20 mL). The combined organic layers were washed with H_2O (30 mL) and brine (30 mL) and dried over Na_2SO_4. The solution was filtered over a short silica plug (↕ 5 cm, ⌀ 4 cm) and eluted with EtOAc (200 mL). The filtrate was concentrated under reduced pressure to obtain the entitled aldehyde **SI-8b** (324 mg, 954 µmol, 61%), which was used for the formation of its hydrazone without further purification and analysis.

*2.8.7. Dimethyl 2-(chloromethyl)terephthalate (**SI-9**)*

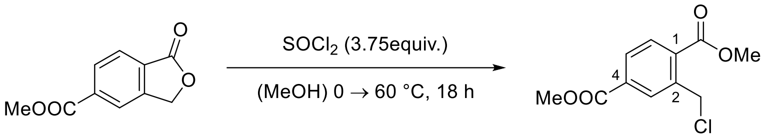

Based on a modified procedure by *Yan et al.*,[13] $SOCl_2$ (1.36 mL, 2.23 g, 18.8 mmol, 3.75 equiv.) was added dropwise to a solution of phthalide (961 mg, 5.00 mmol, 1.00 equiv.) in MeOH (4.0 mL, 1.25 M) at 0 °C. After 10 min, the cooling bath was removed and the reaction mixture was stirred at 60 °C for 18 h. After cooling to r.t., the mixture was poured onto ice and the aqueous layer was extracted with CH_2Cl_2 (3 × 40 mL). The combined organic layers were dried over Na_2SO_4, filtered and the solvent was removed under reduced pressure. The crude product was purified by automated flash column chromatography (Hex/EtOAc) to yield the entitled terephthalate **SI-9** (254 mg, 1.04 mmol, 21%) as white solid, alongside with recovered phthalide (634 mg, 3.30 mmol, 66% recovered).

***R*f** (Hex/EtOAc = 4/1): 0.55 [UV, $KMnO_4$].

^{1}H NMR (500 MHz, $CDCl_3$): δ [ppm] = 3.95 (s, 3H, COOMe), 3.96 (s, 3H, COOMe), 5.05 (s, 2H, CH_2Cl), 8.02 (d, ^3J = 8.1 Hz, 1H, H-6), 8.04 (dd, ^3J = 8.1 Hz, ^4J = 1.6 Hz, 1H, H-5), 8.20 (d, ^4J = 1.6 Hz, 1H, H-3).

^{13}C NMR (126 MHz, $CDCl_3$): δ [ppm] = 166.6 (*C*OOMe), 165.9 (*C*OOMe), 139.1 (C-2), 133.6 (C-1), 133.0 (C-4), 132.0 (C-3), 131.3 (C-6), 129.5 (C-5), 52.8 (OMe), 52.7 (OMe), 44.0 (CH_2Cl).

HRMS (ESI) *m/z*: $[M+H]^+$ Calcd. for $C_{11}H_{12}ClO_4$ 243.0419; Found 243.0411.

IR (ATR): $\tilde{\nu}$ [cm^{-1}] = 3036 (w), 3012 (w), 2960 (w), 2844 (w), 1714 (vs, C=O), 1575 (w, C=C), 1436 (m), 1424 (m), 1298 (m), 1282 (s), 1265 (s), 1239 (vs, OMe), 1191 (s), 1109 (vs), 1071 (s), 956 (m), 821 (m, sp^2 C–H), 792 (m), 765 (s), 742 (vs).

m.p. = 56-57°C.

*2.8.8. 2-(2-Bromoethyl)benzaldehyde (**SI-10**)*

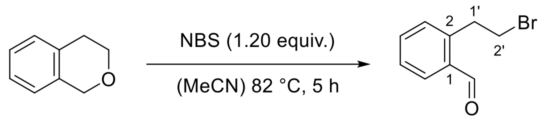

Based on a modified procedure by *D'Acry et al.*,[14] *N*-bromosuccinimide (4.06 g, 22.8 mmol, 1.20 equiv.) was added to a solution of isochromane (2.38 mL, 2.55 g. 19.0 mmol, 1.00 equiv.) in MeCN (19 mL, 1.0 M) and the solution was gradually heated to reflux (82 °C). The reaction mixture was kept at this temperature for 5 h. Afterwards, the solvent was removed under reduced pressure and the residue was suspended with Et_2O (100 mL) and filtered. The filtrate was washed with aq. NaOH (0.1 M, 2 × 100 mL) and brine (100 mL). The organic layer was dried over Na_2SO_4, filtered and the solvent was removed under reduced pressure. The crude product was purified by automated flash column chromatography (Hex/EtOAc) to yield the entitled benzaldehyde **SI-10** (2.98 g, 14.0 mmol, 74%) as yellow oil.

$\boldsymbol{R}_f$ (Hex/EtOAc = 9/1): 0.50 [UV, $KMnO_4$].

^{1}H NMR (500 MHz, $CDCl_3$): δ [ppm] = 3.56 – 3.60 (m, 2H, H-1''), 3.60 – 3.65 (m, 2H, H-2''), 7.34 (dd, ^3J = 7.5 Hz, ^4J = 1.3 Hz, 1H, H-3), 7.49 (*virt*. td, $^3J \approx {}^3J$ = 7.5 Hz, ^4J = 1.3 Hz, 1H, H-5), 7.57 (*virt*. td, $^3J \approx {}^3J$ = 7.5 Hz, ^4J = 1.5 Hz, 1H, H-4), 7.83 (dd, ^3J = 7.5 Hz, ^4J = 1.5 Hz, 1H, H-6), 10.16 (s, 1H, CHO).

^{13}C NMR (126 MHz, $CDCl_3$): δ [ppm] = 193.1 (CHO), 140.7 (C-1), 134.7 (C-6), 134.0 (C-2), 133.9 (C-4), 132.3 (C-3), 127.8 (C-5), 36.5 (C-1'), 32.9 (C-2').

Spectral data matched those reported in the literature.[14]

*2.8.9. 2-(2-(2-Bromoethyl)phenyl)-1,3-dioxolane (**SI-11**)*

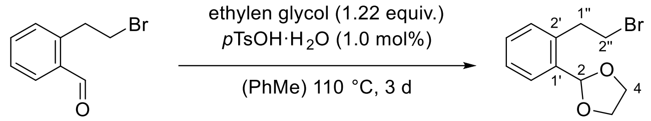

*p*TsOH·H_2O (26.6 mg, 140 µmol, 1.0 mol%) was added to a solution of benzaldehyde **SI-10** (2.97 g, 14.0 mmol, 1.00 equiv.) and ethylene glycol (0.95 mL, 1.06 g, 17.0 mmol. 1.22 equiv.) in PhMe (31 mL, 450 mM) and the solution was heated at 110 °C with a *Dean-Stark*-apparatus for 3 d. The reaction mixture was cooled and the organic layer was washed with sat. $NaHCO_3$ solution (2 × 20 mL) and brine (20 mL). The organic layer was dried over Na_2SO_4, filtered and the solvent was removed under reduced pressure. The product **SI-11** thus obtained (3.30 g, 12.8 mmol, 92%) as a yellow oil was of sufficient purity for the next step.

***R*f** (Hex/EtOAc = 4/1): 0.60 [UV, $KMnO_4$].

^{1}H NMR (500 MHz, $CDCl_3$): δ [ppm] = 3.27 – 3.32 (m, 2H), 3.57 – 3.62 (m, 2H), 4.04 – 4.10 (m, 2H, H^a-4, H^a-5), 4.13 – 4.19 (m, 2H, H^b-4, H^b-5), 5.94 (s, 1H, H-2), 7.23 (dd, ^3J = 7.4 Hz, ^4J = 1.6 Hz, 1H, H-3), 7.29 (*virt*. td, $^3J \approx {}^3J$ = 7.4 Hz, ^4J = 1.3 Hz, 1H, H-5), 7.33 (*virt*. td, $^3J \approx {}^3J$ = 7.4 Hz, ^4J = 1.6 Hz, 1H, H-4), 7.56 (dd, ^3J = 7.4 Hz, ^4J = 1.6 Hz, 1H, H-6).

^{13}C NMR (126 MHz, $CDCl_3$): δ [ppm] = 137.8 (C-1'), 135.4 (C-2'), 130.6 (C-6'), 129.5 (C-5'), 127.3 (C-4'), 127.2 (C-3'), 102.4 (C-2), 65.5 (C-4, C-5), 36.4 (C-1''), 32.8 (C-2'').

HRMS (ESI) *m/z*: $[M+H]^+$ Calcd. for $C_{11}H_{14}BrO_2$ 257.0172; Found 257.0168.

IR (ATR): $\tilde{\nu}$ [cm^{-1}] = 2950 (w), 2885 (w), 1717 (m), 1689 (m), 1601 (w, C=C), 1576 (w, C=C), 1451 (w), 1293 (m), 1264 (m), 1249 (m), 1213 (m), 1068 (s), 943 (m), 751 (vs, sp^2 C–H), 711 (m).

2.8.10. *2-(2-(1,3-dioxolan-2-yl)phenyl)ethan-1-amine (**SI-12**)*

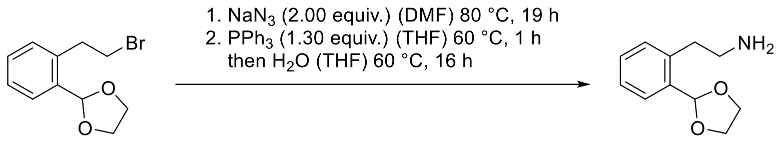

Sodium azide (650 mg, 10.0 mmol, 2.00 equiv.) was added to a solution of bromide **SI-11** (1.29 g, 5.00 mmol, 1.00 equiv.) in DMF (12.5 mL, 400 mM) and the mixture was stirred at 80 °C for 19 h. After cooling to room temperature, the mixture was diluted with EtOAc (100 mL). The organic layer was washed with brine (100 mL), H_2O (2 × 100 mL) and brine (80 mL) again, dried over Na_2SO_4 and filtered. The solvent was removed under reduced pressure und the resulting yellow oil was dried *in vacuo*.

The crude product was dissolved in THF (25 mL, 200 mM) and PPh_3 (1.70 g, 6.50 mmol, 1.30 equiv.) was added in one portion. The mixture was heated slowly to 60 °C and stirred at this temperature, until no further gas evolution was observed (ca. 1 h). Then H_2O (5.0 mL) was added and the reaction mixture was stirred at 60 °C for 16 h. The mixture was diluted with Et_2O (50 mL) and the organic layer was extracted with sat. NH_4Cl solution (2 × 50 mL). The combined aq. layers were basified with aq. NaOH (8 M, 15 mL, pH = 8-9) and extracted with CH_2Cl_2 (2 × 150 mL). The combined organic layers were dried over Na_2SO_4 and filtered. After removal of the solvent under reduced pressure, amine **SI-12** (960 mg, 4.97 mmol, 99%) was obtained as a crude product which was used without further purification in the cyclization reaction.

2.8.11. *2-(2-(1,3-Dioxolan-2-yl)phenethyl)isoindolin-1-one (**SI-2s**)*

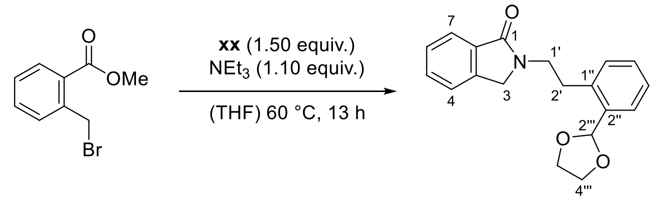

Following GP 4 using amine **SI-12** as substrate, compound **SI-2s** was obtained as a yellow oil (504 mg, 1.63 mmol, 81%).

***R*f** (Hex/EtOAc = 2/3): 0.52 [UV, $KMnO_4$].

^{1}H NMR (500 MHz, DMSO-*d*6): δ [ppm] = 3.01 – 3.06 (m, 2H, H-2’), 3.72 – 3.77 (m, 2H, H-1’), 3.93 – 4.00 (m, 2H, H^a-4’’’, H^a-5’’’), 4.05 – 4.12 (m, 2H, H^b-4’’’, H^b-5’’’), 4.40 (s, 2H, H-3), 5.96 (s, 2H, H-1’’’), 7.23 – 7.27 (m, 2H, H-4’’’, H-6’’), 7.31 (*virt*. td, $^3J \approx {}^3J$ = 7.4 Hz, ^4J = 1.5 Hz, 1H, H-5’’’), 7.45 – 7.50 (m, 2H, H-6, H-3’’), 7.54 – 7.61 (m, 2H, H-4, H-5), 7.67 (*virt*. dt, ^3J = 7.5 Hz, $^4J \approx {}^5J$ = , 1H, H-7).

^{13}C NMR (126 MHz, DMSO-*d*6): δ [ppm] = 167.2 (C-1), 141.9 (C-7a), 137.8 (C-1’’), 135.6 (C-2’’), 132.5 (C-3a), 131.2 (C-5), 130.1 (C-6’’), 129.2 (C-5’’’), 127.8 (C-6), 126.9 (C-3’’), 126.4 (C-4’’’), 123.4 (C-4), 122.7 (C-7), 101.3 (C-2’’’), 64.8 (C-4’’’, C-5’’’), 49.8 (C-3), 43.4 (C-1’), 30.6 (C-2’).

HRMS (ESI) *m/z*: $[M+H]^+$ Calcd. for $C_{19}H_{20}NO_3$ 310.1438; Found 310.1432.

IR (ATR): $\tilde{\nu}$ [cm^{-1}] = 3040 (w), 2930 (w), 2888 (m), 1679 (vs, C=O), 1618 (m, C=C), 1593 (w, C=C), 1470 (m), 1455 (m), 1414 (m), 1383 (m), 1326 (m, O–C–O), 1303 (m), 1144 (w), 1066 (s), 972 (s), 943 (s), 875 (w), 781 (m), 739 (vs, sp^2 C–H), 722 (s), 685 (m).

m.p. = 82-83 °C

*2.8.12. 2-(2-(Bromomethyl)benzyl)isoindolin-1-one (**SI-13**)*

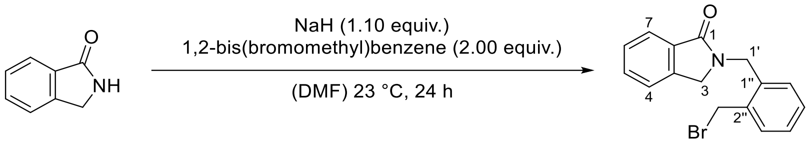

Following GP 3 using 1,2-bis(bromomethyl)benzene as alkylation reagent, compound **SI-13** was obtained as a colorless solid (667 mg, 2.11 mmol, 42%).

***R*f** (Hex/EtOAc = 1/1): 0.70 (UV, $KMnO_4$].

^{1}H NMR (500 MHz, $CDCl_3$): δ [ppm] = 4.26 (s, 2H, H-3), 4.65 (s, 2H, CH_2Br), 4.95 (s, 2H, H-1'), 7.26 – 7.32 (m, 3H, H-4'', H-5'', H-6''), 7.37 – 7.41 (m, 2H, H-4, H-3''), 7.47 (*virt*. td, $^3J \approx ^3J$ = 7.4 Hz, ^4J = 1.1 Hz, 1H, H-6), 7.52 (*virt*. td, $^3J \approx ^3J$ = 7.4 Hz, ^4J = 1.2 Hz, 1H, H-5), 7.90 (*virt*. dt, ^3J = 7.4 Hz, $^4J \approx ^5J$ = 1.2 Hz, 1H, H-7).

^{13}C NMR (126 MHz, $CDCl_3$): δ [ppm] = 168.5 (C-1), 141.4 (C-7a), 136.6 (C-2''), 135.5 (C-1''), 132.6 (C-3a), 131.6 (C-5), 131.4 (C-3''), 130.0 (C-6''), 129.3 (C-5''), 128.7 (C-4''), 128.2 (C-6), 124.0 (C-7), 122.9 (C-4), 49.8 (C-3), 43.7 (C-1'), 31.2 (CH_2Br).

HRMS (ESI) *m/z*: $[M+H]^+$ Calcd. for $C_{16}H_{15}BrNO$ 316.0332; Found 316.0332.

IR (ATR): $\tilde{\nu}$ [cm^{-1}] = 3022 (w), 2905 (w), 1682 (vs, C=O), 1619 (m, C=C), 1472 (m), 1452 (s), 1408 (s), 1326 (m), 1303 (m), 1227 (m), 1210 (m), 992 (m), 774 (m), 735 (vs, sp^2 C–H), 719 (s, sp^2 C–H), 687 (m), 678 (s, C–Br).

m.p. = 102-105 °C.

*2.8.13. 2-((1-Oxoisoindolin-2-yl)methyl)benzaldehyde (**SI-4r**)*

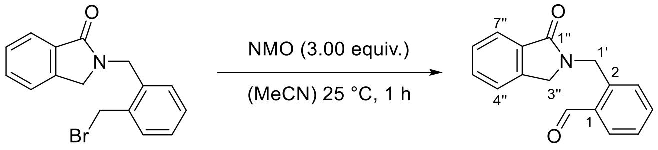

NMO (351 mg, 3.00 mmol, 3.00 equiv.) was added in one portion to a solution of bromide **SI-13** (316 mg, 1.00 mmol, 1.00 equiv.) in MeCN (2.0 mL, 500 mM) and the reaction mixture was stirred at 25 °C for 1 h. The solvent was removed under reduced pressure and the residue was dissolved in CH_2Cl_2 (20 mL). The organic layer was washed with sat. $Na_2S_2O_3$ solution (20 mL) and the aqueous layer was extracted with CH_2Cl_2 (2 × 20 mL). The combined organic layers were dried over Na_2SO_4, filtered and the solvent was removed under reduced pressure. The crude product was subjected to automated flash column chromatography (Hex/EtOAc) to yield the entitled benzaldehyde **SI-4r** (249 mg, 989 µmol, 99%) as a colorless solid.

***R*f** (CH_2Cl_2/ac = 9/1): 0.63 [UV, $KMnO_4$].

^{1}H NMR (500 MHz, $CDCl_3$): δ [ppm] = 4.36 (s, 2H, H-3''), 5.30 (s, 2H, H-1'), 7.39 – 7.43 (m, 2H, H-3, H-4''), 7.46 – 7.52 (m, 2H, H-4, H-5''), 7.52 – 7.58 (m, 2H, H-5, H-6''), 7.87 (dd, ^3J = 7.6 Hz, ^4J = 1.6 Hz, 1H, H-6), 7.90 (*virt*. dt, ^3J = 7.5 Hz, ^4J ≈ ^5J = 1.1 Hz, 1H, H-7''), 10.26 (s, 1H, CHO).

^{13}C NMR (126 MHz, $CDCl_3$): δ [ppm] = 193.6 (CHO), 168.8 (C-1''), 141.5 (C-3a''), 139.1 (C-1), 134.4 (C-4), 134.3 (C-6), 133.9 (C-2), 132.5 (C-7a''), 131.7 (C-5''), 129.7 (C-3), 128.3 (C-5/C-6''), 128.2 (C-5/C-6''), 124.1 (C-7''), 123.0 (C-4''), 50.3 (C-3''), 43.3 (C-1').

HRMS (ESI) *m/z*: $[M+H]^+$ Calcd. for $C_{16}H_{14}NO_2$ 252.1019; Found 252.1019.

IR (ATR): $\tilde{\nu}$ [cm^{-1}] = 3059 (w), 2981 (w), 2913 (w), 2741 (w, CHO), 1682 (vs, C=O), 1599 (m, C=C), 1470 (m), 1454 (m), 1408 (m), 1303 (m), 1210 (m), 757 (m), 735 (vs, sp^2 C–H).

m.p. = 108-110 °C.

*2.8.14. 2-(3,3-Diethoxypropyl)-3-methylisoindolin-1-one (**SI-3o**)*

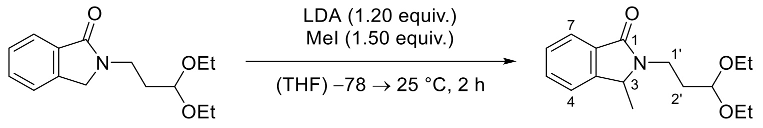

Based on a modified procedure by *Deniau et al.*,[15] a freshly prepared solution of LDA (1 M in THF, 2.40 mL, 2.40 mmol, 1.20 equiv.) was added to a solution of **SI-3a** (527 mg, 2.00 mmol, 1.00 equiv.) in THF (7.6 mL, 0.2 M) at −78 °C to become a deep yellow solution. After 1 h, MeI (187 mL, 426 mg, 3.00 mmol, 1.50 equiv.) was added dropwise. The reaction was kept at −78 °C for further 15 min and then warmed to 25 °C. After 1 h, the solvent was removed under reduced pressure and the residue was dissolved in EtOAc (50 mL). The organic layer was washed with sat. NH_4Cl solution (2 × 40 mL) and brine (40 mL), dried over Na_2SO_4, filtered and the solvent was removed under reduced pressure. The crude product was subjected to automated flash column chromatography (Hex/EtOAc) to yield the entitled isoindolinone **SI-3o** (418 mg, 1.51 mmol, 75%) as a colorless oil.

***R*f** (Hex/EtOAc = 1/1): 0.52 [UV, $KMnO_4$].

^{1}H NMR (500 MHz, $CDCl_3$): δ [ppm] = 1.13 (t, ^3J = 7.1 Hz, 3H, CH_3^a), 1.21 (t, ^3J = 7.1 Hz, 3H, CH_3^b), 1.47 (d, ^3J = 6.8 Hz, 3H, C-3-CH_3), 1.93 (dddd, ^2J = 13.9 Hz, ^3J = 8.3 Hz, ^3J = 6.9 Hz, ^3J = 5.4 Hz, 1H, H^a-2’), 2.01 (*virt*. ddt, ^2J = 13.9 Hz, ^3J = 8.5 Hz, $^3J \approx {}^3J$ = 5.7 Hz. 1H, H^b-2’), 3.33 (ddd, ^2J = 14.2 Hz, ^3J = 8.3 Hz, ^3J = 5.7 Hz, 1H, H^a-1’), 3.47 (dq, ^2J = 9.3 Hz, ^3J = 7.1 Hz, 1H, OC*H*H^a), 3.54 (dq, ^2J = 9.3 Hz, ^3J = 7.1 Hz, 1H, OC*H*H^b), 3.61 – 3.72 (m, 2H, OCHH^a, OCHH^b), 3.99 (ddd, ^2J = 14.2 Hz, ^3J = 8.5 Hz, ^3J = 6.9 Hz, 1H, H^b-1’), 4.54 – 4.60 (m, 2H, H-3, H-3’), 7.41 (*virt*. dq, ^3J = 7.5 Hz, $^4J \approx {}^4J \approx {}^5J$ = 0.9 Hz, 1H, H-4), 7.63 (*virt*. td, $^3J \approx {}^3J$ = 7.5 Hz, ^4J = 0.9 Hz, 1H, H-6), 7.53 (*virt*. td, $^3J \approx {}^3J$ = 7.5 Hz, ^4J = 0.9 Hz, 1H, H-5), 7.82 (*virt*. dt, ^3J = 7.5 Hz, $^4J \approx {}^5J$ = 0.9 Hz, 1H, H-7).

^{13}C NMR (126 MHz, $CDCl_3$): δ [ppm] = 168.2 (C-1), 147.1 (C-3a), 132.1 (C-7a), 131.5 (C-5), 128.2 (C-6), 123.6 (C-7), 122.0 (C-4), 101.5 (C-3’’), 62.2 (OCH_2^b), 61.6 (OCH_2^a), 55.8 (C-3), 36.2 (C-1’), 32.8 (C-2’), 18.3 (C-3-*C*H_3), 15.5 (CH_3^a), 15.4 (CH_3^b).

HRMS (ESI) *m/z*: $[M+Na]^+$ Calcd. for $C_{16}H_{23}NO_3Na$ 300.1570; Found 300.1565.

IR (ATR): $\tilde{\nu}$ [cm^{-1}] = 2975 (w), 2931 (w), 2877 (w), 1676 (vs, C=O), 1619 (w, C=C), 1596 (w, C=C), 1470 (m), 1447 (m), 1409 (m), 1374 (m), 1123 (s, O–C–O), 1056 (vs, O–C–O), 866 (w), 799 (w), 760 (s), 727 (s, sp^2 C–H), 695 (s).

*2.8.15. Methyl 2-(chloromethyl)nicotinate (**SI-14**)*

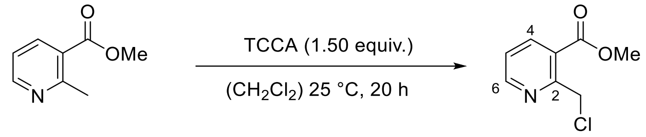

Based on a modified procedure by *Spitale et al.*,[16] trichloroisocyanuric acid (5.07 g, 21.8 mmol, 1.50 equiv.) was added to a solution of methyl 2-methylnicotinate (2.00 mL, 2.20 g, 14.6 mmol, 1.00 equiv.) in CH_2Cl_2 (14.6 mL, 1.0 M) and the resulting suspension was stirred at 25 °C for 20 h. The reaction was diluted with CH_2Cl_2 (20 mL) and quenched by addition of sat. $NaHCO_3$ solution (40 mL). The aqueous layer was washed with CH_2Cl_2 (2 × 40 mL). The combined organic layers were dried over Na_2SO_4, filtered and the solvent was removed under reduced pressure to yield the entitled nicotinate **SI-14** (2.53 g, 13.6 mmol, 94%) as a pale-yellow oil.

***R*f** (Hex/EtOAc = 9/1): 0.20 [UV, $KMnO_4$].

^{1}H NMR (500 MHz, $CDCl_3$): δ [ppm] = 3.96 (s, 3H, OMe), 5.12 (s, 2H, CH_2Cl), 7.36 (dd, ^3J = 7.9 Hz, ^3J = 4.9 Hz, 1H, H-5), 8.28 (dd, ^3J = 7.9 Hz, ^4J = 1.8 Hz, 1H, H-4), 8.72 (dd, ^3J = 4.9 Hz, ^4J = 1.5 Hz, 1H, H-6).

^{13}C NMR (126 MHz, $CDCl_3$): δ [ppm] = 166.0 (*C*OOMe), 157.4 (C-2), 152.4 (C-6), 139.3 (C-4), 125.5 (C-3), 123.4 (C-5), 52.9 (OMe), 45.6 (CH_2Cl).

Spectral data matched those reported in the literature.[16]

3. Optimization of the reaction conditions with hydrazone

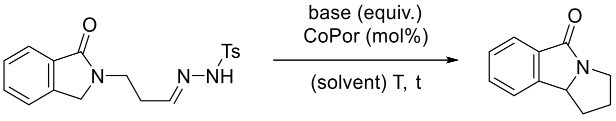

The reaction was performed under the indicated conditions and were initiated by addition of the solvent or the base when the latter is liquid to the substrate (100 µmol) and the indicated catalyst. The reaction was terminated after the indicated time by evaporation of the solvent. All yields refer to isolated material.

#	R	mol%	base	equiv	T [°C]	t [h]	solvent	c [mM]	**1a** [%]
1	Ph	5	Cs_2CO_3	1.50	60	24	TFT	200	14
2	Ph	5	Cs_2CO_3	1.50	60	24	DCE	200	-
3	Ph	5	Cs_2CO_3	1.50	60	24	TFT	50	37
4	Ph	5	Cs_2CO_3	1.50	60	24	TFT	20	39
5	Ph	5	Cs_2CO_3	1.50	23	24	CH_2Cl_2	50	-
6	Ph	5	Cs_2CO_3	1.50	60	7	TFT	20	25
7	Ph	5	Cs_2CO_3	1.50	60	7	TFT	10	23
8	Ph	5	Cs_2CO_3	1.50	40	24	TFT	20	-
9	Ph	5	Cs_2CO_3	1.50	40	24	PhMe	20	-
10	Ph	5	Cs_2CO_3	1.50	60	24	PhH	20	53
11	Ph	5	Cs_2CO_3	1.50	60	24	PhMe	20	41
12	Ph	5	Cs_2CO_3	1.50	60	24	PhCl	20	59
13	Ph	5	Cs_2CO_3	1.50	60	24	*o*DCB	20	65
14	Ph	5	Cs_2CO_3	1.50	60	24	py	20	60
15	Ph	5	-	-	60	24	py	20	-
16	Ph	5	Cs_2CO_3	1.50	60	24	diglyme	20	74
17	Ph	5	NEt_3	1.50	60	24	*o*DCB	20	-
18	Ph	5	DIPEA	1.50	60	24	*o*DCB	20	-
19	Ph	5	DBU	1.50	60	24	*o*DCB	20	59
20	Ph	5	DBN	1.50	60	24	*o*DCB	20	38
21	Ph	5	DABCO	1.50	60	24	*o*DCB	20	-
22	Ph	5	TMG	1.50	60	24	*o*DCB	20	35
23	Ph	5	TBD	1.50	60	24	*o*DCB	20	62
24	Ph	5	NaH	1.50	60	24	*o*DCB	20	-
25	Ph	5	Cs_2CO_3	1.00	60	24	*o*DCB	20	59
26	Ph	5	Cs_2CO_3	2.00	60	24	*o*DCB	20	71
27	Ph	5	Cs_2CO_3	2.50	60	24	*o*DCB	20	73

28	Ph	5	Cs_2CO_3	3.00	60	24	*o*DCB	20	71
29	Ph	5	Cs_2CO_3	5.00	60	24	*o*DCB	20	71
30	Ph	5	Cs_2CO_3	1.50	60	24	$CHCl_3$	20	21
31	C_6F_5	5	Cs_2CO_3	2.50	60	24	*o*DCB	20	67
32	Mes	5	Cs_2CO_3	2.50	60	24	*o*DCB	20	75
33	PMP	5	Cs_2CO_3	2.50	60	24	*o*DCB	20	74
34	Ph	2.5	Cs_2CO_3	2.50	60	24	*o*DCB	20	58
35	Ph	1	Cs_2CO_3	2.50	60	24	*o*DCB	20	78
36	Ph	7.5	Cs_2CO_3	2.50	60	24	*o*DCB	20	73
37	-	-	Cs_2CO_3	2.50	60	24	*o*DCB	20	-
38	Ph	0.5	Cs_2CO_3	2.50	60	24	*o*DCB	20	mixture
39	Ph, Ns	1	Cs_2CO_3	2.50	60	24	*o*DCB	20	-
40	Ph, Ns	1	DBU	2.50	60	24	*o*DCB	20	mixture
**41**	**Ph**	**1**	**DBU**	**2.50**	**60**	**24**	***o*DCB**	**20**	**83**
42	Ph	1	Cs_2CO_3[1]	2.50	60	24	*o*DCB	20	79
43	Ph	1	Cs_2CO_3	2.50	60	48	*o*DCB	20	83

4. Catalysis

4.1. General procedure 8 (GP 8): Co-catalyzed Cyclization

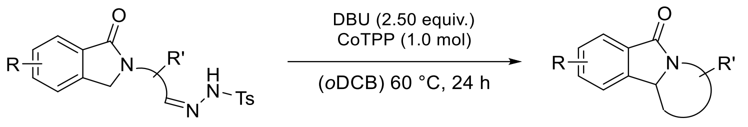

The corresponding hydrazone (100 µmol, 1.00 equiv.) and Co(TPP) (672 µg, 1.00 µmol, 1.0 mol%) was added to an oven-dried *Schlenk* tube and the atmosphere was exchanged (3×). The solids were dissolved in *o*DCB (5.0 mL, 20 mM) to yield a bright red solution. Then, DBU (37.3 µL, 38.1 mg, 250 µmol, 2.50 equiv.) was added dropwise (about seven drops), upon which the solution turned from red to copper. The tube was sealed and the reaction mixture was heated to 60 °C for 24 h. After cooling to room temperature, the solvent was removed under reduced pressure and the crude product was subjected to automated flash column chromatography (Hex/EtOAc) to yield the entitled cyclization products.

*4.1.1. 1,2,3,9b-Tetrahydro-5H-pyrrolo[2,1-a]isoindol-5-one (**1a**)*

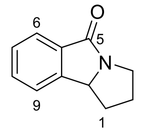

Following GP 8, compound **1a** was obtained as a colorless solid (14.3 mg, 82.6 µmol, 83%).

One-pot experiment: Based on the procedure by *Lankelma et al*,[17] Aldehyde **SI-4a** (18.9 mg, 100 µmol, 1.00 equiv.), *p*-toluenesulfonyl hydrazide (22.4 mg, 120 µmol, 1.20 equiv.) and Co(TPP) (672 µg, 1.00 µmol, 1.0 mol%) were added to an oven-dried *Schlenk* tube and the atmosphere was exchanged. The solids were dissolved in *o*DCB (5.0 mL, 20 mM) and DBU (37.3 µL, 38.1 mg, 250 µmol, 2.50 equiv.) was added dropwise. The tube was sealed and the reaction mixture was stirred at 60 °C for 24 h. After cooling to room temperature, the solvent was removed under reduced pressure and the crude product was subjected to automated flash column chromatography (Hex/EtOAc) to yield **1a** (14.0 mg, 80.8 µmol, 81%) as a colorless solid.

$\boldsymbol{R_f}$ (Hex/EtOAc = 1/1): 0.22 [UV, $KMnO_4$].

^{1}H NMR (500 MHz, $CDCl_3$): δ [ppm] = 1.19 – 1.30 (m, 1H, H^a-1), 2.26 – 2.42 (m, 3H, H^b-1, H-2), 3.43 (ddd, ^2J = 11.6 Hz, ^3J = 8.8 Hz, ^3J = 3.2 Hz, 1H, H^a-3), 3.73 (*virt*. dt, ^2J = 11.4 Hz, $^3J \approx {}^3J$ = 8.1 Hz, 1H, H^b-3), 4.68 (dd, ^3J = 10.6 Hz, ^3J = 5.6 Hz, 1H, H-9b), 7.41 – 7.47 (m, 2H, H-7, H-9), 7.52 (*virt*. td, ^3J = 7.5 Hz, ^4J = 1.1 Hz, 1H, H-8), 7.79 (*virt*. dt, ^3J = 7.5 Hz, $^4J \approx {}^5J$ = 1.1 Hz, 1H, H-6).

^{13}C NMR (126 MHz, $CDCl_3$): δ [ppm] = 171.8 (C-5), 146.6 (C-9a), 133.8 (C-5a), 131.7 (C-8), 128.5 (C-7), 124.1 (C-6), 122.8 (C-9), 64.84 (C-9b), 42.1 (C-3), 29.9 (C-1), 29.4 (C-2).

Spectral data matched those reported in the literature.[18]

*4.1.2. 2,2-Dimethyl-1,2,3,9b-tetrahydro-5H-pyrrolo[2,1-a]isoindol-5-one (**1b**)*

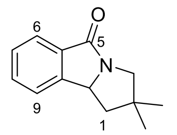

Following GP 8, compound **1b** was obtained as a colorless solid (16.8 mg, 83.5 μmol, 83%).

***R*f** (Hex/EtOAc = 1/1): 0.51 [UV, $KMnO_4$].

^{1}H NMR (500 MHz, $CDCl_3$): δ [ppm] = 1.13 (s, 3H, CH_3^a), 1.26 (dd, ^2J = 12.0 Hz, ^3J =10.4 Hz, 1H, H^a-1), 1.32 (s, 3H, CH_3^b), 2.06 (dd, ^2J = 12.0 Hz, ^3J =6.4 Hz, 1H, H^b-1), 3.17 (d, ^2J = 11.4 Hz, 1H, H^a-3), 3.55 (d, ^2J = 11.4 Hz, 1H, H^b-3), 4.90 (dd, ^3J = 10.4 Hz, ^3J = 6.4 Hz, 1H, H-9b), 7.39 (*virt*. dq, ^3J = 7.4 Hz, $^4J \approx {}^4J$ = 0.9 Hz, 1H, H-9), 7.44 (*virt*. td, $^3J \approx {}^3J$ = 7.4 Hz, ^4J = 0.9 Hz, 1H, H-7), 7.51 (*virt*. td, $^3J \approx {}^3J$ = 7.4 Hz, ^4J = 1.1 Hz, 1H, H-8), 7.79 (*virt*. dt, ^3J = 7.5 Hz, $^4J \approx {}^5J$ = 1.1 Hz, 1H, H-6).

^{13}C NMR (126 MHz, $CDCl_3$): δ [ppm] = 172.0 (C-5), 147.2 (C-9a), 133.6 (C-5a), 131.8 (C-8), 128.4 (C-7), 124.2 (C-6), 122.7 (C-9), 63.9 (C-9b), 56.2 (C-3), 44.7 (C-2), 44.3 (C-1), 28.8 (CH_3^a), 28.3 (CH_3^b).

HRMS (ESI) *m/z*: $[M+H]^+$ Calcd. for $C_{13}H_{16}NO$ 202.1226; Found 202.1229.

IR (ATR): $\tilde{\nu}$ [cm^{-1}] = 3064 (w), 3049 (w), 2957 (m), 2935 (m), 2878 (m), 1674 (vs, C=O), 1614 (m, C=C), 1467 (m), 1385 (s), 1336 (m), 1234 (m), 1095 (m), 845 (m), 742 (vs, sp^2 C–H), 690 (vs), 674 (s).

m.p. = 138-143 °C.

*4.1.3. 6-Bromo-1,2,3,9b-tetrahydro-5H-pyrrolo[2,1-a]isoindol-5-one (**1c**)*

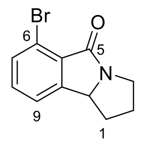

Following GP 8, compound **1c** was obtained as a colorless solid (21.9 mg, 86.9 μmol, 87%).

***R*f** (Hex/EtOAc = 1/1): 0.36 [UV, $KMnO_4$].

^{1}H NMR (500 MHz, $CDCl_3$): δ [ppm] = 1.20 – 1.30 (m, 1H, H^a-1), 2.26 – 2.41 (m, 3H, H^b-1, H-2), 3.41 (ddd, ^2J = 11.8 Hz, ^3J = 9.1 Hz, ^3J = 3.1 Hz, 1H, H^a-3), 3.76 (ddd, ^2J = 11.8 Hz, ^3J = 8.8 Hz, ^3J = 8.0 Hz, 1H, H^b-3), 4.60 (dd, ^3J = 10.5 Hz, ^3J = 5.4 Hz, 1H, H-9b), 7.33 – 7.38 (m, 2H, H-8, H-9), 7.58 (dd, ^3J = 7.3 Hz, ^4J = 1.5 Hz, 1H, H-7).

^{13}C NMR (126 MHz, $CDCl_3$): δ [ppm] = 169.7 (C-5), 149.1 (C-9a), 133.4 (C-7), 132.7 (C-8), 131.2 (C-5a), 122.0 (C-9), 119.1 (C-6), 63.4 (C-9b), 42.5 (C-3), 30.1 (C-1), 29.0 (C-2).

HRMS (ESI) *m/z*: $[M+H]^+$ Calcd. for $C_{11}H_{11}ClNO$ 252.0019; Found 252.0017.

IR (ATR): $\tilde{\nu}$ [cm^{-1}] = 3068 (w), 2966 (w), 2952 (w), 2862 (w), 2875 (w), 1693 (vs, C=O), 1601 (s, C=C), 1572 (s, C=C), 1454 (s), 1377 (vs), 1327 (s), 1217 (s), 1096 (m, C–Br), 874 (s), 792 (s, sp^2 C–H), 779 (vs, sp^2 C–H), 700 (vs).

m.p. = 71-72 °C.

*4.1.4. 6-Methyl-1,2,3,9b-tetrahydro-5H-pyrrolo[2,1-a]isoindol-5-one (**1d**)*

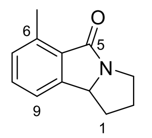

Following GP 8, compound **1d** was obtained as a colorless solid (16.7 mg, 89.2 μmol, 89%).

***R*f** (Hex/EtOAc = 1/1): 0.46 [UV, $KMnO_4$].

^{1}H NMR (500 MHz, $CDCl_3$): δ [ppm] = 1.17 – 1.28 (m, 1H, H^a-1), 2.23 – 2.39(m, 3H, H^b-1, H-2), 2.69 (s, 3H, CH_3), 3.39 (ddd, ^2J = 11.7 Hz, ^3J = 9.0 Hz, ^3J = 3.4 Hz, 1H, H^a-3), 3.72 (*virt*. dt, ^2J = 11.7 Hz, ^3J ≈ ^3J = 8.6 Hz, 1H, H^b-3), 4.60 (dd, ^3J = 10.6 Hz, ^3J = 5.5 Hz, 1H, H-9b), 7.18 (dq, ^3J = 7.6 Hz, ^4J = 0.9 Hz, 1H, H-7), 7.23 (dd, ^3J = 7.6 Hz, ^4J = 1.0 Hz, 1H, H-9), 7.87 (*virt*. t, ^3J ≈ ^3J = 7.6 Hz, 1H, H-8).

^{13}C NMR (126 MHz, $CDCl_3$): δ [ppm] = 172.9 (C-5), 147.2 (C-9a), 138.2 (C-6), 131.3 (C-8), 130.8 (C-5a), 130.4 (C-7), 120.2 (C-9), 64.1 (C-9b), 42.2 (C-3), 30.2 (C-1), 29.1 (C-2), 17.4 (CH_3).

HRMS (ESI) *m/z*: $[M+H]^+$ Calcd. for $C_{12}H_{14}NO$ 188.1070; Found 188.1068.

IR (ATR): $\tilde{\nu}$ [cm^{-1}] = 2970 (w), 2891 (w), 1687 (vs, C=O), 1605 (w, C=C), 1482 (w), 1377 (s), 1307 (m), 1216 (m), 1085 (w, C–Br), 784 (m), 726 (w), 698 (w).

m.p. = 62-73 °C.

*4.1.5. 7-Chloro-1,2,3,9b-tetrahydro-5H-pyrrolo[2,1-a]isoindol-5-one (**1e**)*

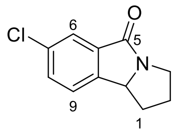

Following GP 8, compound **1e** was obtained as a colorless solid (19.1 mg, 92.0 μmol, 92%).

***R*f** (Hex/EtOAc = 1/1): 0.35 [UV, $KMnO_4$].

^{1}H NMR (500 MHz, $CDCl_3$): δ [ppm] = 1.18 – 1.29 (m, 1H, H^a-1), 2.27 – 2.44 (m, 3H, H^b-1, H-2), 3.43 (ddd, ^2J = 11.5 Hz, ^3J = 8.7 Hz, ^3J = 2.9 Hz, 1H, H^a-3), 3.76 (*virt*. dt, ^2J = 11.5 Hz, ^3J ≈ ^3J = 8.3 Hz, 1H, H^b-3), 4.65 (dd, ^3J = 10.7 Hz, ^3J = 5.4 Hz, 1H, H-9b), 7.37 (*virt*. dt, ^3J = 8.0 Hz, ^4J ≈ ^5J = 0.7 Hz, 1H, H-9), 7.49 (dd, ^3J = 8.0 Hz, ^4J = 1.8 Hz, 1H, H-8), 7.76 (d, ^4J = 1.8 Hz, 1H, H-6).

^{13}C NMR (126 MHz, $CDCl_3$): δ [ppm] = 170.3 (C-5), 144.6 (C-9a), 135.7 (, 134.8 (, 131.9 (C-8), 124.3 (C-6), 124.1 (C-9), 64.5 (C-9b), 42.2 (C-3), 29.9 (C-1), 29.3 (C-2).

HRMS (ESI) *m/z*: $[M+H]^+$ Calcd. for $C_{11}H_{11}ClNO$ 208.0524; Found 208.0524.

IR (ATR): $\tilde{\nu}$ [cm^{-1}] = 3082 (w), 2981 (m, sp^3 C–H), 2892 (w, sp^3 C–H), 1693 (vs, C=O), 1468 (m), 1425 (s), 1374 (s), 1174 (w), 1063 (w), 825 (w, sp^2 C–H), 714 (w), 688 (w).

m.p. = 92-93 °C.

*4.1.6. 7-Bromo-1,2,3,9b-tetrahydro-5H-pyrrolo[2,1-a]isoindol-5-one (**1f**)*

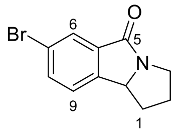

Following GP 8, compound **1f** was obtained as a colorless solid (23.9 mg, 94.8 μmol, 95%).

***R*f** (Hex/EtOAc = 1/1): 0.26 [UV, $KMnO_4$].

^{1}H NMR (500 MHz, $CDCl_3$): δ [ppm] = 1.18 – 1.29 (m, 1H, H^a-1), 2.26 – 2.43 (m, 3H, H^b-1, H-2), 3.42 (ddd, ^2J = 11.5 Hz, ^3J = 8.8 Hz, ^3J = 2.9 Hz, 1H, H^a-3), 3.72 (*virt.* dt, ^2J = 11.5 Hz, $^3J \approx {}^3J$ = 8.3 Hz, 1H, H^b-3), 4.63 (dd, ^3J = 10.7 Hz, ^3J = 5.4 Hz, 1H, H-9b), 7.31 (*virt*. dt, ^3J = 8.0 Hz, $^4J \approx {}^5J$ = 0.7 Hz, 1H, H-9), 7.64 (dd, ^3J = 8.0 Hz, ^4J = 2.0 Hz, 1H, H-8), 7.92 (d, ^4J = 2.0 Hz, 1H, H-6).

^{13}C NMR (126 MHz, $CDCl_3$): δ [ppm] = 170.1 (C-5), 145.1 (C-9a), 135.9 (C-5a), 134.6 (C-8), 127.3 (C-6), 124.4 (C-9), 122.5 (C-7), 64.6 (C-9b), 42.1 (C-3), 29.8 (C-1), 29.3 (C-2).

HRMS (ESI) *m/z*: $[M+H]^+$ Calcd. for $C_{11}H_{11}BrNO$ 252.0019; Found 252.0016.

IR (ATR): $\tilde{\nu}$ [cm^{-1}] = 3036 (w), 2972 (w), 2940 (w), 2888 (w), 2873 (w), 1688 (vs, C=O), 1606 (w, C=C), 1582 (w, C=C), 1664 (m), 1420 (s), 1382 (s), 1327 (s), 1226 (m), 1170 (m), 1141 (m), 1118 (m), 1053 (m, C–Br), 894 (m), 823 (s), 754 (s, sp^2 C–H), 701 (s), 680 (vs).

m.p. = 94-95 °C.

*4.1.7. 8-Fluoro-1,2,3,9b-tetrahydro-5H-pyrrolo[2,1-a]isoindol-5-one (**1g**)*

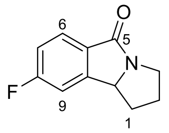

Following GP 8, compound **1g** was obtained as a colorless solid (17.0 mg, 88.9 μmol, 89%).

***R*f** (Hex/EtOAc = 1/1): 0.24 [UV, $KMnO_4$].

^{1}H NMR (500 MHz, $CDCl_3$): δ [ppm] = 1.21 – 1.33 (m, 1H, H^a-1), 2.26 – 2.44 (m, 3H, H^b-1, H-2), 3.41 (ddd, ^2J = 11.6 Hz, ^3J = 8.9 Hz, ^3J = 3.2 Hz, 1H, H^a-3), 3.72 (*virt* dt, ^2J = 11.6 Hz, $^3J \approx {}^3J$ = 8.3 Hz, 1H, H^b-3), 4.65 (dd, ^3J = 10.6 Hz, ^3J = 5.7 Hz, 1H, H-9b), 7.10 – 7.17 (m, 2H, H-7, H-9), 7.76 (dd, $^3J_{H–H}$ = 8.4 Hz, $^4J_{H–F}$ = 5.0 Hz, 1H, H-6).

^{13}C NMR (126 MHz, $CDCl_3$): δ [ppm] = 170.9 (C-5), 165.3 (d, $^1J_{C–F}$ = 251.6 Hz, C-8), 148.9 (d, $^3J_{C–F}$ = 9.6 Hz, C-9a), 129.8 (d, $^4J_{C–F}$ = 2.3 Hz, C-5a), 126.2 (d, $^3J_{C–F}$ = 9.7 Hz, C-6), 116.2 (d,^{2} $J_{C–F}$ = 23.6 Hz, C-7), 110.3 (d, $^2J_{C–F}$ = 23.9 Hz, C-9), 64.4 (d, $^4J_{C–F}$ = 2.6 Hz, C-9b), 42.3 (C-3), 29.9 (C-1), 29.2 (C-2).

^{19}F NMR (376 MHz, $CDCl_3$): δ [ppm] = −107.5 (*virt* td, $^3J \approx {}^3J$ = 8.5 Hz, ^4J = 5.0 Hz).

HRMS (ESI) *m/z*: $[M+H]^+$ Calcd. for $C_{11}H_{11}FNO$ 192.0819; Found 192.0818.

IR (ATR): $\tilde{\nu}$ [cm^{-1}] = 3053 (w), 2973 (w), 2900 (w), 2873 (w), 1682 (vs, C=O), 1621 (s, C=C), 1476 (m), 1391 (s), 1246 (vs, C–F), 1141 (s), 1088 (s), 935 (s), 901 (s), 883 (s), 829 (s, sp^2 C–H), 757 (vs), 690 (vs).

m.p. = 67-68 °C.

*4.1.8. 8-Bromo-1,2,3,9b-tetrahydro-5H-pyrrolo[2,1-a]isoindol-5-one (**1h**)*

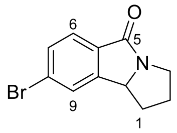

Following GP 8, compound **1h** was obtained as a colorless solid (23.0 mg, 91.2 μmol, 91%).

***R*f** (Hex/EtOAc = 1/1): 0.29 [UV, $KMnO_4$].

^{1}H NMR (500 MHz, $CDCl_3$): δ [ppm] = 1.20 – 1.31 (m, 1H, H^a-1), 2.26 – 2.43 (m, 3H, H^b-1, H-2), 3.41 (ddd, ^2J = 11.4 Hz, ^3J = 8.8 Hz, ^3J = 2.9 Hz, 1H, H^a-3), 3.72 (*virt* dt, ^2J = 11.4 Hz, $^3J \approx {}^3J$ = 8.1 Hz, 1H, H^b-3), 4.65 (dd, ^3J = 10.6 Hz, ^3J = 5.4 Hz, 1H, H-9b), 7.58 – 7.61 (m, 2H, H-7, H-9), 7.65 (d, ^3J = 8.7 Hz, 1H, H-6).

^{13}C NMR (126 MHz, $CDCl_3$): δ [ppm] = 170.8 (C-5), 148.2 (C-9a), 132.8 (C-5a), 132.0 (C-7), 126.5 (C-8), 126.3 (C-9), 125.6 (C-7), 64.4 (C-9b), 42.1 (C-3), 29.8 (C-1), 29.2 (C-2).

HRMS (ESI) *m/z*: $[M+H]^+$ Calcd. for $C_{11}H_{11}BrNO$ 252.0019; Found 252.0015.

IR (ATR): $\tilde{\nu}$ [cm^{-1}] = 2975 (w), 2949 (w), 2928 (w), 2884 (w), 1677 (vs, C=O), 1605 (s, C=C), 1418 (m), 1380 (vs), 1224 (m), 1143 (m), 1054 (s, C–Br), 874 (s, sp^2 C–H), 843 (vs, sp^2 C–H), 786 (s), 750 (s), 684 (vs).

m.p. = 155-157 °C.

*4.1.9. 8-Nitro-1,2,3,9b-tetrahydro-5H-pyrrolo[2,1-a]isoindol-5-one (**1i**)*

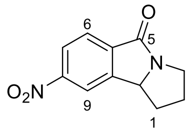

Following GP 8, compound **1i** was obtained as a colorless solid (9.3 mg, 42.6 μmol, 43%).

***R*f** (Hex/EtOAc = 1/1): 0.17 [UV, $KMnO_4$].

^{1}H NMR (500 MHz, $CDCl_3$): δ [ppm] = 1.27 – 1.37 (m, 1H, H^a-1), 2.35 – 2.49 (m, 3H, H^b-1, H-2), 3.49 (ddd, ^2J = 12.0 Hz, ^3J = 8.6 Hz, ^3J = 2.8 Hz, 1H, H^a-3), 3.78 (*virt* dt, ^2J = 12.0 Hz, $^3J \approx {}^3J$ = 8.2 Hz, 1H, H^b-3), 4.78 (dd, ^3J = 10.7 Hz, ^3J = 5.3 Hz, 1H, H-9b), 7.94 (d, ^3J = 8.3 Hz, 1H, H-6), 8.31 (d, ^4J = 2.0 Hz, 1H, H-9), 8.35 (dd, ^3J = 8.3 Hz, ^4J = 2.0 Hz, 1H, H-7).

^{13}C NMR (126 MHz, $CDCl_3$): δ [ppm] = 168.9 (C-5), 150.2 (C-8), 147.1 (C-9a), 139.4 (C-5a), 125.2 (C-6), 124.4 (C-7), 118.5 (C-9), 64.6 (C-9b), 42.4 (C-3), 29.8 (C-1), 29.2 (C-2).

HRMS (ESI) *m/z*: $[M+H]^+$ Calcd. for $C_{11}H_{11}N_2O_3$ 219.0764; Found 219.0765.

IR (ATR): $\tilde{\nu}$ [cm^{-1}] = 3107 (w), 3037 (w), 2954 (m), 2924 (m), 2854 (m), 1683 (vs, C=O), 1324 (w, C=C), 1297 (w, C=C), 1526 (vs, NO_2), 1458 (m), 1391 (s), 1338 (vs, NO_2), 1225 (m), 1168 (m), 855 (m, sp^2 C–H), 816 (s, sp^2 C–H), 732 (vs), 682 (vs).

m.p. = 131-135 °C.

*4.1.10. 5-Oxo-2,3,5,9b-tetrahydro-1H-pyrrolo[2,1-a]isoindole-8-carbonitrile (**1j**)*

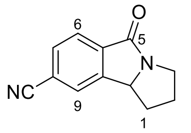

Following GP 8, compound **1j** was obtained as a colorless solid (16.1 mg, 81.2 μmol, 81%).

***R*f** (Hex/EtOAc = 1/1): 0.21 [UV, $KMnO_4$].

^{1}H NMR (500 MHz, $CDCl_3$): δ [ppm] = 1.23 – 1.32 (m, 1H, H^a-1), 2.32 – 2.47 (m, 3H, H^b-1, H-2), 3.46 (ddd, ^2J = 12.0 Hz, ^3J = 9.1 Hz, ^3J = 3.3 Hz, 1H, H^a-3), 3.76 (*virt* dt, ^2J = 12.0 Hz, $^3J \approx {}^3J$ = 8.3 Hz, 1H, H^b-3), 4.73 (dd, ^3J = 10.7 Hz, ^3J = 5.4 Hz, 1H, H-9b), 7.73 – 7.78 (m, 2H, H-7, H-9), 7.89 (dd, ^3J = 7.7 Hz, ^5J = 0.9 Hz, 1H, H-6).

^{13}C NMR (126 MHz, $CDCl_3$): δ [ppm] = 169.3 (C-5), 146.6 (C-9a), 137.9 (C-5a), 132.6 (C-7), 126.8 (C-9), 125.0 (C-6), 118.4 (CN), 115.1 (C-8), 64.5 (C-9b), 42.2 (C-3), 29.7 (C-1), 29.2 (C-2).

HRMS (ESI) *m/z*: $[M+H]^+$ Calcd. for $C_{12}H_{11}N_2O$ 199.0866; Found 199.0866.

IR (ATR): $\tilde{\nu}$ [cm^{-1}] = 3047 (w), 2954 (w), 2927 (w), 2892 (w), 2227 (m, C≡N), 1687 (vs, C=O), 1621 (w, C=C), 1582 (w, C=C), 1424 (m), 1395 (s), 1314 (m), 1143 (m), 904 (m), 844 (s, sp^2 C–H), 792 (m), 756 (m), 689 (vs), 676 (s).

m.p. = 140-142 °C.

*4.1.11. Methyl 5-oxo-2,3,5,9b-tetrahydro-1H-pyrrolo[2,1-a]isoindole-8-carboxylate (**1k**)*

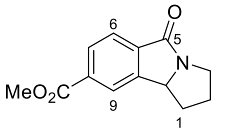

Following GP 8, compound **1k** was obtained as a colorless solid (19.1 mg, 82.6 μmol, 83%).

***R*f** (Hex/EtOAc = 1/1): 0.27 [UV, $KMnO_4$].

^{1}H NMR (500 MHz, $CDCl_3$): δ [ppm] = 1.20 – 1.32 (m, 1H, H^a-1), 2.31 – 2.45 (m, 3H, H^b-1, H-2), 3.45 (ddd, ^2J = 11.8 Hz, ^3J = 9.0 Hz, ^3J = 3.5 Hz, 1H, H^a-3), 3.75 (*virt* dt, ^2J = 11.8 Hz, $^3J \approx {}^3J$ = 8.4 Hz, 1H, H^b-3), 3.95 (s, 2H, OMe), 4.72 (dd, ^3J = 10.8 Hz, ^3J = 5.3 Hz, 1H, H-9b), 7.84 (d, ^3J = 7.9 Hz, 1H, H-6), 8.12 (d, ^4J = 1.5 Hz, 1H, H-9), 8.14 (dd, ^3J = 7.9 Hz, ^4J = 1.5 Hz, 1H, H-7).

^{13}C NMR (126 MHz, $CDCl_3$): δ [ppm] = 170.4 (C-5), 166.6 (*C*OOMe), 146.3 (C-9a), 137.9 (C-5a), 133.1 (C-8), 130.0 (C-7), 124.2 (C-9), 124.1 (C-6), 64.8 (C-9b), 52.7 (OMe), 42.2 (C-3), 29.8 (C-1), 29.3 (C-2).

HRMS (ESI) *m/z*: $[M+H]^+$ Calcd. for $C_{13}H_{14}NO_3$ 232.0968; Found 232.0966.

IR (ATR): $\tilde{\nu}$ [cm^{-1}] = 2981 (w), 2891 (w), 1722 (s, COO), 1697 (CON), 1591 (w, C=C), 1439 (m), 1385 (m), 1291 (s), 1267 (s), 1209 (m), 1085 (m), 746 (m), 689 (w).

m.p. = 127-129 °C.

*4.1.12. 9-Methoxy-1,2,3,9b-tetrahydro-5H-pyrrolo[2,1-a]isoindol-5-one (**1l**)*

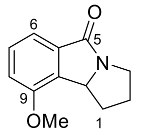

Following GP 8, compound **1l** was obtained as a colorless solid (17.2 mg, 84.6 µmol, 85%).

***R*f** (Hex/EtOAc = 1/1): 0.29 [UV, $KMnO_4$].

^{1}H NMR (500 MHz, $CDCl_3$): δ [ppm] = 1.14 – 1.24 (m, 1H, H^a-1), 2.25 – 2.42 (m, 3H, H^b-1, H-2), 3.40 (ddd, ^2J = 11.7 Hz, ^3J = 8.8 Hz, ^3J = 2.8 Hz, 1H, H^a-3), 3.68 (*virt* dt, ^2J = 11.7 Hz, $^3J \approx {}^3J$ = 8.7 Hz, 1H, H^b-3), 3.90 (s, 3H, OMe), 4.66 (dd, ^3J = 10.8 Hz, ^3J = 5.6 Hz, 1H, H-9b), 6.99 (dd, ^3J = 7.5 Hz, ^4J = 1.3 Hz, 1H, H-8), 7.38 (dd, ^3J = 7.5 Hz, ^4J = 1.3 Hz, 1H, H-6), 7.41 (*virt*. t, $^3J \approx {}^3J$ = 7.5 Hz, 1H, H-7).

^{13}C NMR (126 MHz, $CDCl_3$): δ [ppm] = 171.9 (C-5), 154.1 (C-9), 135.5 (C-5a), 134.6 (C-9a), 130.2 (C-7), 116.1 (C-6), 113.0 (C-8), 63.2 (C-9b), 55.6 (OMe), 41.2 (C-3), 29.4 (C-2), 29.3 (C-1).

HRMS (ESI) *m/z*: $[M+H]^+$ Calcd. for $C_{14}H_{14}NO_2$ 204.1019; Found 204.1019.

IR (ATR): $\tilde{\nu}$ [cm^{-1}] = 2972 (w, sp^3 C–H), 2890 (w, sp^3 C–H), 2840 (w), 1685 (vs, C=O), 1598 (s, C=C), 1490 (s, C=C), 1442 (m), 1379 (m), 1340 (m), 1267 (vs, ArOMe), 1071 (s), 753 (s, sp^2 C–H).

m.p. = 111-113 °C.

*4.1.13. 9-Bromo-1,2,3,9b-tetrahydro-5H-pyrrolo[2,1-a]isoindol-5-one (**1m**)*

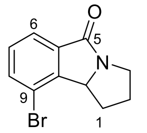

Following GP 8, compound **1m** was obtained as a colorless solid (20.9 mg, 82.8 µmol, 83%).

Scale up experiment: DBU (373 µL, 381 mg, 2.50 mmol, 2.50 equiv.) was added to a solution of hydrazone **2m** (463 mg, 1.00 mmol, 1.00 equiv.) and Co(TPP) (6.72 mg, 10.0 µmol, 1.0 mol%) in *o*DCB (50 mL) and the flask was sealed and stirred at 60 °C. After a reaction time of 48 h, the solvent was removed under reduced pressure and the crude product was purified by automated flash column chromatography. The entitled isoindolinone **1m** (205 mg, 814 µmol. 81%) was obtained as a colorless solid.

***R*f** (Hex/EtOAc = 1/1): 0.38 [UV, $KMnO_4$].

^{1}H NMR (500 MHz, $CDCl_3$): δ [ppm] = 1.20 – 1.30 (m, 1H, H^a-1), 2.29 – 2.44 (m, 2H, H-2), 2.58 (*virt*. dtd, ^2J = 12.2 Hz, $^3J \approx {}^3J$ = 6.0 Hz, ^3J = 1.9 Hz, 1H, H^b-1), 3.44 (ddd, ^2J = 11.7 Hz, ^3J = 8.9 Hz, ^3J = 3.2 Hz, 1H, H^a-3), 3.75 (*virt* dt, ^2J = 11.7 Hz, $^3J \approx {}^3J$ = 8.5 Hz, 1H, H^b-3), 4.65 (dd, ^3J = 10.7 Hz, ^3J = 6.0 Hz, 1H, H-9b), 7.35 (*virt*. t, $^3J \approx {}^3J$ = 7.7 Hz, 1H, H-7), 7.65 (dd, ^3J = 7.7 Hz, ^4J = 0.9 Hz, 1H, H-8), 7.74 (dd, ^3J = 7.7 Hz, ^4J = 0.9 Hz, 1H, H-6).

^{13}C NMR (126 MHz, $CDCl_3$): δ [ppm] = 170.6 (C-5), 146.5 (C-9a), 135.9 (C-5a), 134.8 (C-8), 130.4 (C-7), 123.1 (C-6), 117.8 (C-4), 65.5 (C-9b), 42.1 (C-3), 29.1 (C-2), 28.6 (C-1).

HRMS (ESI) *m/z*: $[M+H]^+$ Calcd. for $C_{11}H_{11}BrNO$ 252.0019; Found 252.0019.

IR (ATR): $\tilde{\nu}$ [cm^{-1}] = 2974 (w), 2891 (w), 1697 (vs, C=O), 1607 (w, C=C), 1575 (w, C=C), 1458 (m), 1380 (s), 1329 (w), 1208 (w), 1012 (m, C–Br), 784 (w), 755 (s, sp^2 C–H).

m.p. = 126-128 °C.

*4.1.14. 9-Trifluormethyl-1,2,3,9b-tetrahydro-5H-pyrrolo[2,1-a]isoindol-5-one (**1n**)*

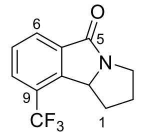

Following GP 8, compound **1n** was obtained as a colorless solid (22.4 mg, 92.9 μmol, 93%).

***R*f** (Hex/EtOAc = 1/1): 0.36 [UV, $KMnO_4$].

^{1}H NMR (500 MHz, $CDCl_3$): δ [ppm] = 1.27 – 1.37 (m, 1H, H^a-1), 2.31 – 2.48 (m, 3H, H^b-1, H-2), 3.45 (ddd, ^2J = 11.7 Hz, ^3J = 8.9 Hz, ^3J = 3.2 Hz, 1H, H^a-3), 3.77 (*virt* dt, ^2J = 11.7 Hz, $^3J \approx {}^3J$ = 8.5 Hz, 1H, H^b-3), 4.88 (dd, ^3J = 10.9 Hz, ^3J = 5.8 Hz, 1H, H-9b), 7.60 (*virt*. t, $^3J \approx {}^3J$ = 7.7 Hz, 1H, H-7), 7.77 (d, ^3J = 7.7 Hz, 1H, H-8), 7.99 (d, ^3J = 7.7 Hz, 1H, H-6).

^{13}C NMR (126 MHz, $CDCl_3$): δ [ppm] = 169.7 (C-5), 143.2 (q, $^3J_{C-F}$ = 2.4 Hz, C-9a), 135.5 (C-5a), 129.1 (C-7), 128.5 (q, $^3J_{C-F}$ = 4.5 Hz, C-8), 127.7 (C-6), 125.8 (q, $^2J_{C-F}$ = 33.6 Hz, C-9), 123.8 (q, $^1J_{C-F}$ = 273.0 Hz, CF_3), 64.3 (C-9b), 41.8 (C-3), 29.9 (C-1), 28.9 (C-2).

^{19}F NMR (376 MHz, $CDCl_3$): δ [ppm] = −61.0 (s, CF_3).

HRMS (ESI) *m/z*: $[M+H]^+$ Calcd. for $C_{12}H_{11}F_3NO$ 242.0787; Found 242.0787.

IR (ATR): $\tilde{\nu}$ [cm^{-1}] = 3073 (w), 3050 (w), 2987 (w), 2901 (w), 1688 (vs, C=O), 1602 (m, C=C), 1386 (s), 1328 (vs), 1214 (m), 1107 (vs, C–F), 1014 (m), 829 (m), 773 (s, sp^2 C–H), 746 (s), 700 (m), 659 (m).

m.p. = 97-98 °C.

*4.1.15. 9b-Methyl-1,2,3,9b-tetrahydro-5H-pyrrolo[2,1-a]isoindol-5-one (**1o**)*

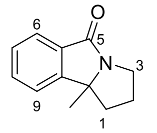

Following GP 8, compound **1o** was obtained as a colorless oil (16.5 mg, 88.1 μmol, 88%).

***R*f** (Hex/EtOAc = 1/1): 0.42 [UV, $KMnO_4$].

^{1}H NMR (500 MHz, $CDCl_3$): δ [ppm] = 1.44 (*virt*. td, $^2J \approx {}^3J$ = 11.9 Hz, ^3J = 8.9 Hz, 1H, H^a-1), 1.51 (s, 3H, CH_3), 2.02 (ddd, ^2J = 11.9 Hz, ^3J = 7.4 Hz, ^3J = 1.9 Hz, 1H, H^b-1), 2.29 – 2.37 (m, 1H, H^a-2), 2.38 – 2.46 (m, 1H, H^b-2), 3.39 (ddd, ^2J = 11.9 Hz, ^3J = 9.4 Hz, ^3J = 3.8 Hz, 1H, H^a-3), 3.84 (ddd, ^2J = 11.9 Hz, ^3J = 9.1 Hz, ^3J = 7.5 Hz, 1H, H^b-3), 7.40 (*virt*. dt, ^3J = 7.5 Hz, $^4J \approx {}^5J$ = 1.0 Hz, 1H, H-9), 7.43 (*virt* td, $^3J \approx {}^3J$ = 7.5 Hz, ^4J = 1.0 Hz, 1H, H-7), 7.53 (*virt*. td, $^3J \approx {}^3J$ = 7.5 Hz, ^4J = 1.0 Hz, 1H, H-8), 7.76 (*virt*. dt, ^3J = 7.5 Hz, $^4J \approx {}^5J$ = 1.0 Hz, 1H, H-6).

^{13}C NMR (126 MHz, $CDCl_3$): δ [ppm] = 171.3 (C-5), 151.9 (C-9a), 132.1 (C-5a), 132.0 (C-8), 128.4 (C-7), 124.2 (C-9), 121.7 (C-6), 70.3 (C-9b), 40.1 (C-3), 35.0 (C-1), 28.5 (C-2), 23.6 (CH_3).

HRMS (ESI) *m/z*: $[M+H]^+$ Calcd. for $C_{12}H_{14}NO$ 188.1070; Found 188.1067.

IR (ATR): $\tilde{\nu}$ [cm^{-1}] = 3049 (w), 2969 (w), 2892 (w), 1683 (s, C=O), 1614 (m, C=C), 1466 (m), 1356 (vs), 1330 (s), 1306 (m), 1223 (w), 1208 (w), 1112 (w), 1087 (w), 921 (w), 874 (w), 759 (vs, sp^2 C–H), 695 (vs), 676 (s).

*4.1.16. 7,8,9,9a-Tetrahydro-5H-pyrido[2,3-a]pyrrolizin-5-one (**1p**)*

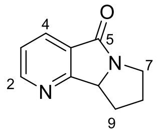

Following GP 8, compound **1p** was obtained as a colorless oil (15.8 mg, 90.7 μmol, 91%).

***R*f** (CH_2Cl_2/ac = 2/3): 0.60 [UV, $KMnO_4$].

^{1}H NMR (500 MHz, $CDCl_3$): δ [ppm] = 1.37 (*virt*. dtd, ^2J = 12.0 Hz, $^3J \approx {}^3J$ = 10.7 Hz, ^3J = 8.2 Hz, 1H, H^a-9), 2.30 – 2.42 (m, 2H, H-8), 2.45 (*virt*. dtd, ^2J = 12.0 Hz, $^3J \approx {}^3J$ = 6.2 Hz, ^3J = 1.9 Hz, 1H, H^b-1), 3.46 (ddd, ^2J = 11.8 Hz, ^3J = 8.9 Hz, ^3J = 3.4 Hz, 1H, H^a-7), 3.79 (*virt*. dt, ^2J = 11.8 Hz, $^3J \approx {}^3J$ = 8.4 Hz, 1H, H^b-7), 4.73 (dd, ^3J = 10.7 Hz, ^3J = 6.2 Hz, 1H, H-9a), 7.39 (dd, ^3J = 7.7 Hz, ^3J = 4.9 Hz, 1H, H-3), 8.07 (dd, ^3J = 7.7 Hz, ^4J = 1.6 Hz, 1H, H-4), 8.70 (dd, ^3J = 4.9 Hz, ^4J = 1.6 Hz, 1H, H-2).

^{13}C NMR (126 MHz, $CDCl_3$): δ [ppm] = 169.8 (C-5), 166.5 (C-9b), 152.6 (C-2), 132.4 (C-4), 127.6 (C-4a), 123.6 (C-3), 66.1 (C-9a), 42.4 (C-7), 29.2 (C-9), 28.6 (C-8).

HRMS (ESI) *m/z*: $[M+H]^+$ Calcd. for $C_{10}H_{11}N_2O$ 175.0866; Found 175.0863.

IR (ATR): $\tilde{\nu}$ [cm^{-1}] = 3087 (w), 2974 (w), 2893 (w), 1678 (s, C=O), 1604 (m, C=C), 1582 (m, C=N), 1474 (w), 1380 (s), 1328 (m), 1216 (m), 1100 (m), 938 (w), 827 (w), 783 (s, sp^2 C–H), 747 (vs, sp^2 C–H), 7221 (m), 684 (m).

*4.1.17. 1,3,4,10b-Tetrahydropyrido[2,1-a]isoindol-6(2H)-one (**1q**)*

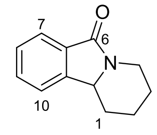

Following GP 8, compound **1q** was obtained as a colorless solid (12.4 mg, 66.2 μmol, 66%) alongside with side-product **4q** (1.9 mg, 10.3 μmol, 10%).

Spectral data of **1q**:

***R*f** (Hex/EtOAc = 1/1): 0.26 [UV, $KMnO_4$].

^{1}H NMR (500 MHz, $CDCl_3$): δ [ppm] = 1.11 (*virt.* tdd, $^2J \approx {}^3J$ = 13.2 Hz, ^3J = 11.8 Hz, ^3J = 3.5 Hz, 1H, H^a-1), 1.38 (*virt*. qdd, $^2J \approx {}^3J \approx {}^3J$ = 13.2 Hz, ^3J = 5.1 Hz, ^3J = 3.5 Hz, 1H, H^a-3), 1.65 (*virt*. qt, 1H, $^2J \approx {}^3J \approx {}^3J$ = 13.2 Hz, $^3J \approx {}^3J$ = 3.5 Hz H^a-2), 1.81 (*virt*. dqd, 1H, ^2J = 13.2 Hz, $^3J \approx {}^3J \approx {}^3J$ = 3.5 Hz, ^3J = 1.7 Hz H^b-3), 1.99 (*virt*. dqd, ^2J = 13.2 Hz, 1H, $^3J \approx {}^3J \approx {}^3J$ = 3.5 Hz, ^3J = 1.4 Hz, H^b-2), 2.34 (dddd, 1H, ^2J = 13.2 Hz, ^3J = 3.9 Hz, ^3J = 3.5 Hz, ^3J = 1.4 Hz H^b-1), 2.97 (*virt*. td, 1H, $^2J \approx {}^3J$ = 13.2 Hz, ^3J = 3.5 Hz, H^a-4), 4.25 (dd, ^3J = 11.8 Hz, ^3J = 3.9 Hz, 1H, H-10b), 4.48 (*virt*. ddt, 1H, ^2J = 13.2 Hz, ^3J = 5.1 Hz, ^3J = 1.7 Hz, H^b-4), 7.40 (*virt*. dq, ^3J = 7.4 Hz, $^4J \approx {}^4J \approx {}^5J$ = 1.0 Hz, 1H, H-10), 7.44 (*virt*. td, $^3J \approx {}^3J$ = 7.4 Hz, ^4J = 1.0 Hz, 1H, H-8), 7.51 (*virt*. td, $^3J \approx {}^3J$ = 7.4 Hz, ^4J = 1.0 Hz, 1H, H-9), 7.84 (*virt* dt, ^3J = 7.4 Hz, $^4J \approx {}^5J$ = 1.0 Hz, 1H, H-7).

^{13}C NMR (126 MHz, $CDCl_3$): δ [ppm] = 166.1 (C-6), 145.7 (C-10a), 132.5 (C-6a), 131.1 (C-9), 128.1 (C-8), 123.7 (C-7), 121.7 (C-10), 59.0 (C-10b), 39.7 (C-4), 31.8 (C-1), 25.3 (C-3), 23.7 (C-2).

Spectral data matched those reported in the literature.[19]

Spectral data of side product **4q**:

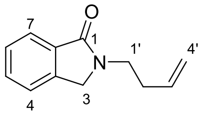

^{1}H NMR (500 MHz, $CDCl_3$): δ [ppm] = 2.45 (*virt*. qt, $^3J \approx {}^3J$ = 6.9 Hz, $^4J \approx {}^4J$ = 1.4 Hz, 2H, H-2'), 3.71 (t, ^3J = 6.9 Hz, 2H, H-1'), 4.40 (s, 2H, H-3), 5.05 (*virt*. dq, ^3J = 10.2 Hz, $^2J \approx {}^4J$ = 1.4 Hz, 1H, H_Z-4'), 5.11 (*virt*. dq, ^3J = 17.1 Hz, $^2J \approx {}^4J$ = 1.4 Hz, 1H, H_E-4'), 5.84 (ddt, ^3J = 17.1 Hz, ^3J = 10.2 Hz, ^3J = 6.9 Hz, 1H, H-3'), 7.40 – 7.48 (m, 2H, H-4, H-6), 7.52 (*virt*. td, $^3J \approx {}^3J$ = 7.5 Hz, ^4J = 1.2 Hz, 1H, H-5), 7.84 (d, ^3J = 7.5 Hz, 1H, H-7).

Spectral data matched those reported in the literature.[20]

*4.1.18. 11b,12-Dihydroisoindolo[2,1-b]isoquinolin-7(5H)-one (**1r**)*

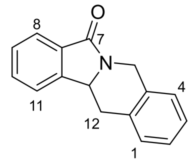

Following GP 8, compound **1r** was obtained as a colorless solid (23.5 mg, 99.8 μmol, 99%).

$\boldsymbol{R_f}$ (Hex/EtOAc = 1/1): 0.39 [UV, $KMnO_4$].

^{1}**H NMR** (500 MHz, $CDCl_3$): δ [ppm] = 2.65 (dd, ^2J = 15.4 Hz, ^3J = 11.9 Hz, 1H, H^a-12), 3.40 (dd, ^2J = 15.4 Hz, ^3J = 4.2 Hz, 1H, H^b-12), 4.58 (d, ^2J = 17.5 Hz, 1H, H^a-5), 4.63 (dd, ^3J = 11.9 Hz, ^3J = 4.2 Hz, 1H, H-11b), 5.33 (d, ^2J = 17.5 Hz, 1H, H^b-5), 7.20 – 7.25 (m, 2H, H-1, H-4), 7.27 – 7.31 (m, 2H, H-2, H-3), 7.51 (*virt*. td, $^3J \approx {}^3J$ = 7.5 Hz, ^4J = 1.1 Hz, 1H, H-9), 7.56 (dd, ^3J = 7.5 Hz, ^4J = 1.1 Hz, 1H, H-11), 7.60 (*virt*. td, $^3J \approx {}^3J$ = 7.5 Hz,, ^4J = 1.1 Hz, 1H, H-10), 7.92 (*virt*. dt, ^3J = 7.5 Hz, $^4J \approx {}^5J$ = 1.1 Hz, 1H, H-8).

^{13}**C NMR** (126 MHz, $CDCl_3$): δ [ppm] = 167.4 (C-7), 145.7 (C-11a), 132.7 (C-7a), 132.1 (C-4a), 131.9 (C-12a), 131.6 (C-10), 129.3 (C-1), 128.6 (C-9), 127.3 (C-2/C-3), 127.1 (C-2/C-3), 127.0 (C-4), 123.9 (C-8), 122.2 (C-11), 55.9 (C-11b), 42.1 (C-5), 35.0 (C-12).

Spectral data matched those reported in the literature.[21]

*4.1.19. 7,8,13,13a-tetrahydro-5H-benzo[4,5]azepino[2,1-a]isoindol-5-one (**1s**)*

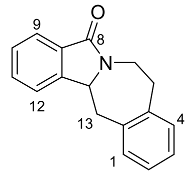

Following GP 8, compound **1s** was obtained as a colorless solid (22.9 mg, 91.6 μmol, 92%).

$\boldsymbol{R_f}$ (Hex/EtOAc = 1/1): 0.64 [UV, $KMnO_4$].

^{1}**H NMR** (500 MHz, $CDCl_3$): δ [ppm] = 2.89 – 2.98 (m, 2H, H^a-8, H^a-13), 2.99 – 3.07 (m, 2H, H^a-7, H^b-8), 3.30 (dd, ^2J = 14.6 Hz, ^3J = 1.8 Hz, 1H, H^b-13), 4.45 (dd, ^3J = 11.0 Hz, ^3J = 1.8 Hz, 1H, H-13a), 4.79 – 4.83 (m, 1H, H^b-7), 7.21 – 7.27 (m, 3H, H-9*,H-10, H-11), 7.29 – 7.34 (m, 1H, H-12*), 7.50 (*virt*. td, $^3J \approx {}^3J$ = 7.4 Hz, ^4J = 1.3 Hz, 1H, H-3), 7.56 (d, ^3J = 7.4 Hz, 1H, H-1), 7.60 (*virt*. td, $^3J \approx {}^3J$ = 7.4 Hz,, ^4J = 1.1 Hz, 1H, H-2), 7.89 (d, ^3J = 7.4 Hz, 1H, H-4).

* Signals are interchangeable.

^{13}**C NMR** (126 MHz, $CDCl_3$): δ [ppm] = 167.2 (C-5), 145.0 (C-13b), 141.5 (C-8a), 137.9 (C-12a), 132.1 (C-4a), 131.7 (C-2), 130.0 (2C, C-9, C-12), 128.6 (C-3), 127.6 (C-10*), 127.2 (C-11*), 123.9 (C-4), 122.2 (C-1), 61.3 (C-13a), 42.7 (C-13), 41.3 (C-7), 36.4 (C-8).

* Signals are interchangeable.

Spectral data matched those reported in the literature.[21]

*4.1.20. 3-Methyl-1,2,3,9b-tetrahydro-5H-pyrrolo[2,1-a]isoindol-5-one (**1u**)*

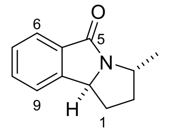

Following GP 8, compound **1u** was obtained as a colorless solid (15.5 mg, 82.8 μmol, 83%).

d.r. = 95/5.

***R*f** (Hex/EtOAc = 1/1): 0.46 [UV, $KMnO_4$].

^{1}H NMR (500 MHz, $CDCl_3$): δ [ppm] = 1.22 – 1.31 (m, 1H, H^a-1), 1.43 (d, ^3J = 6.4 Hz, 3H, CH_3), 1.92 (*virt*. tdd, $^2J \approx {^3J}$ = 12.9 Hz, ^3J = 8.4 Hz, ^3J = 6.4 Hz, 1H, H^a-2), 2.25 – 2.32 (m, 1H, H^b-1), 2.56 (*virt.* dtd, ^2J = 12.9 Hz, $^3J \approx {^3J}$ = 7.3 Hz, ^3J = 1.2 Hz, 1H, H^b-2), 4.03 – 4.11 (m, 1H, H-3), 4.76 (dd, ^3J = 10.9 Hz, ^3J =5.7 Hz, 1H, H-9b), 7.41 (*virt*. dq, ^3J = 7.5 Hz, $^4J \approx {^4J}$ = 1.0 Hz, 1H, H-9), 7.44 (*virt*. td, $^3J \approx {^3J}$ = 7.5 Hz, ^4J = 1.0 Hz, 1H, H-7), 7.51 (*virt*. td, $^3J \approx {^3J}$ = 7.5 Hz, ^4J = 1.1 Hz, 1H, H-8), 7.79 (*virt*. dt, ^3J = 7.5 Hz, $^4J \approx {^5J}$ = 1.1 Hz, 1H, H-6).

^{13}C NMR (126 MHz, $CDCl_3$): δ [ppm] = 171.7 (C-5), 146.4 (C-9a), 134.0 (C-5a), 131.6 (C-8), 128.4 (C-7), 124.1 (C-6), 122.7 (C-9), 64.1 (C-9b), 50.7 (C-3), 38.3 (C-2), 30.5 (C-1), 22.5 (CH_3).

HRMS (ESI) *m/z*: $[M+H]^+$ Calcd. for $C_{12}H_{14}NO$ 188.1070; Found 188.1068.

IR (ATR): $\tilde{\nu}$ [cm^{-1}] = 29665 (w), 2945 (w), 2927 (w), 2945 (w), 1681 (vs, C=O), 1614 (w, C=C), 1474 (m), 1370 (vs), 1323 (s), 1222 (m), 1169 (m), 1154 (m), 1106 (m), 962 (m), 841 (m), 768 (vs, sp^2 C–H), 743 (s), 698 (vs), 679 (m).

m.p. : 57-58 °C

Significant NOE contacts:

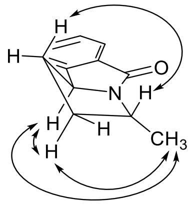

*4.1.21. 3-((tert-Butyldimethylsilyl)oxy)-1,3,4,10b-tetrahydropyrido[2,1-a]isoindol-6(2H)-one (**1v**)*

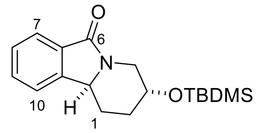

Following GP 8, compound **1v** was obtained as a colorless solid (22.5 mg, 70.9 μmol, 71%).

d.r. = 97/3.

***R*f** (Hex/EtOAc = 3/2): 0.48 [UV, $KMnO_4$].

^{1}H NMR (500 MHz, $CDCl_3$): δ [ppm] = 0.10 (s, 3H, $SiCH_3^a$), 0.11 (s, 3H, $SiCH_3^b$), 0.91 [s, 9H, $C(CH_3)_3$], 1.19 (*virt*. tdd, $^2J \approx {}^3J$ = 13.3 Hz, ^3J = 11.8 Hz, ^3J = 3.6 Hz, 1H, H^a-1), 1.68 (*virt*. tdd, $^2J \approx {}^3J$ = 13.3 Hz, ^3J = 10.8 Hz, ^3J = 3.6 Hz, 1H, H^a-2), 2.08 (*virt*. dqd, ^2J = 13.3 Hz, $^3J \approx {}^3J \approx {}^3J$ = 3.6 Hz, ^4J = 1.8 Hz, 1H, H^b-2), 2.34 (*virt*. dq, ^2J = 13.3 Hz, $^3J \approx {}^3J \approx {}^3J$ = 3.6 Hz, 1H, H^b-1), 2.81 (dd, ^2J = 12.8 Hz, ^3J = 10.1 Hz, 1H, H^a-4), 3.75 – 3.65 (m, 1H, H-3), 4.25 (dd, ^3J = 11.8 Hz, ^3J = 3.6 Hz, 1H, H-10b), 4.51 (ddd, ^2J = 12.8 Hz, ^3J = 5.5 Hz, ^4J = 1.8 Hz, 1H, H^b-4), 7.40 (*virt*. dt, ^3J = 7.4 Hz, $^4J \approx {}^5J$ = 1.0 Hz 1H, H-10), 7.45 (*virt*. td, $^3J \approx {}^3J$ = 7.4 Hz, ^4J = 1.0 Hz, 1H, H-8), 7.55 (*virt*. td, $^3J \approx {}^3J$ = 7.4 Hz, ^4J = 1.0 Hz, 1H, H-9), 7.83 (*virt*. dt, ^3J = 7.4 Hz, $^4J \approx {}^5J$ = 1.0 Hz, 1H, H-7).

^{13}C NMR (126 MHz, $CDCl_3$): δ [ppm] = 166.4 (C-6), 145.2 (C-10a), 132.5 (C-6a), 131.4 (C-9), 128.3 (C-8), 123.9 (C 7), 121.9 (C-10), 68.0 (C-3), 58.2 (C-10b), 46.4 (C-4), 33.5 (C-2), 29.5 (C-1), 25.9 [C(CH$_3$)$_3$], 18.2 [C(CH$_3$)$_3$], −4.5 ($SiCH_3^a$), −4.6 ($SiCH_3^b$).

HRMS (ESI) *m/z*: $[M+H]^+$ Calcd. for $C_{18}H_{28}NO_2Si$ 318.1884; Found 318.1878.

IR (ATR): $\tilde{\nu}$ [cm^{-1}] = 3048 (w), 2953 (m), 2928 (m), 2886 (w), 2856 (m), 1688 (vs, C=O), 1618 (C=C), 1470 (m), 1426 (m), 1251 (m), 1089 (s, C–O), 978 (m), 860 (s), 834 (vs, sp^2 C–H), 775 (vs, sp^2 C–H), 728 (s), 686 (s).

m.p. = 82-83 °C.

Significant NOE contacts:

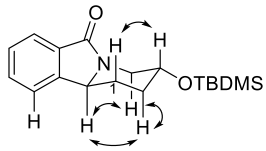

*4.1.22. 3-((4-Methoxybenzyl)oxy)-1,3,4,10b-tetrahydropyrido[2,1-a]isoindol-6(2H)-one (**1w**)*

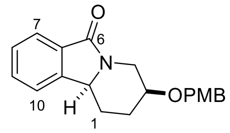

Following GP 8, compound **1w** was obtained as a colorless oil (25.3 mg, 78.2 μmol, 78%).

d.r. = 90/10.

***R*f** (Hex/EtOAc = 3/2): 0.36 [UV, $KMnO_4$].

^{1}H NMR (500 MHz, $CDCl_3$): δ [ppm] = 1.77 – 1.91 (m, 2H, H^a-1, H^a-2), 2.15 (*virt*. dtd, ^{2}J = 10.8 Hz, $^{3}J \approx {}^{3}J$ = 7.3 Hz, ^{3}J = 6.0 Hz, 1H, H^b-2), 2.23 – 2.30 (m, 1H, H^b-1), 3.60 (dd, ^{2}J = 14.2 Hz, ^{3}J = 7.3 Hz, 1H, H^a-4), 3.80 (s, 3H, OMe), 4.08 (dd, ^{2}J = 14.2 Hz, ^{3}J = 3.4 Hz, 1H, H^b-4), 4.29 (*virt*. dq, $^{3}J \approx {}^{3}J \approx {}^{3}J$ = 7.3 Hz, ^{3}J = 3.4 Hz, 1H, H-3), 4.50 (d, ^{2}J = 17.4 Hz, 1H, OC*H*H), 4.67 (d, ^{2}J = 17.4 Hz, 1H, OCH*H*), 4.83 (*virt*. t, ^{3}J = 7.2 Hz, 1H, H-10b), 6.85 – 6.89 (m, 2H, H-3'), 7.25 – 7.28 (m, 2H, H-2'), 7.41 (*virt*. dt, ^{3}J = 7.5 Hz, $^{4}J \approx {}^{5}J$ = 1.0 Hz 1H, H-10), 7.45 (*virt*. td, $^{3}J \approx {}^{3}J$ = 7.5 Hz, ^{4}J = 1.0 Hz, 1H, H-8), 7.52 (*virt*. td, $^{3}J \approx {}^{3}J$ = 7.5 Hz, ^{4}J = 1.0 Hz, 1H, H-9), 7.86 (*virt*. dt, ^{3}J = 7.5 Hz, $^{4}J \approx {}^{5}J$ = 1.0 Hz, 1H, H-7).

^{13}C NMR (126 MHz, $CDCl_3$): δ [ppm] = 169.0 (C-6), 159.1 (C-4'), 142.0 (C-10a), 134.6 (C-1'), 132.7 (C-6a), 131.4 (C-9), 128.0 (C-8), 127.4 (C-2'), 123.8 (C-7), 122.8 (C-10), 113.9 (C-3'), 81.5 (C-10b), 79.1 (C-3), 55.4 (OMe), 52.1 (OCH_2), 47.0 (C-4), 33.9 (C-1), 29.3 (C-2).

HRMS (ESI) *m/z*: $[M+H]^+$ Calcd. for $C_{20}H_{22}NO_3$ 324.1594; Found 324.1588.

IR (ATR): $\tilde{\nu}$ [cm^{-1}] = 3057 (w), 2929 (w), 2839 (w), 1671 (s, C=O), 1602 (m, C=C), 1513 (s), 1395 (m), 1246 (vs, ArOMe), 1170 (s), 1030 (s, ArOMe), 830 (s, sp^2 C–H), 732 (vs, sp^2 C–H).

Significant NOE contacts:

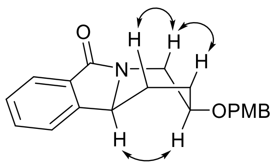

*4.1.23. 2-Methyl-1,3,4,10b-tetrahydropyrido[2,1-a]isoindol-6(2H)-one (**1x**)*

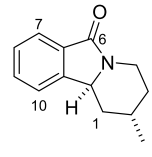

Following GP 8, compound **1x** was obtained as a colorless solid (18.9 mg, 93.9 µmol, 94%).

d.r. = 94/6.

$\boldsymbol{R_f}$ (Hex/EtOAc = 3/2): 0.26 [UV, $KMnO_4$].

^{1}**H NMR** (500 MHz, $CDCl_3$): δ [ppm] = 1.28 (d, ^{3}J = 7.3 Hz, 3H, CH_3), 1.35 (*virt*. td, $^{2}J \approx {}^{3}J$ = 12.8 Hz, ^{3}J = 4.4 Hz, 1H, H^a-1), 1.60 (*virt*. ddt, ^{2}J = 13.4 Hz, ^{3}J = 4.0 Hz, $^{3}J \approx {}^{3}J$ = 2.0 Hz, 1H, H^a-3), 1.68 (*virt*. tt, $^{2}J \approx {}^{3}J$ = 13.4 Hz, $^{3}J \approx {}^{3}J$ = 5.2 Hz, 1H, H^b-3), 2.13 (*virt*. ddt, ^{2}J = 12.8 Hz, ^{3}J = 4.1 Hz, $^{3}J \approx {}^{4}J$ = 1.9 Hz, 1H, H^b-1), 2.23 – 2.33 (m, 1H, H-2), 3.20 (*virt*. td, $^{2}J \approx {}^{3}J$ = 13.4 Hz, ^{3}J = 4.0 Hz 1H, H^a-4), 4.34 (ddd, ^{2}J = 13.4 Hz, ^{3}J = 5.2 Hz, ^{3}J = 2.0 Hz, 1H, H^b-4), 4.51 (dd, ^{3}J = 12.8 Hz, ^{3}J = 4.0 Hz, 1H, H-10b), 7.39 (*virt*. dq, ^{3}J = 7.5 Hz, $^{4}J \approx {}^{4}J \approx {}^{5}J$ = 1.0 Hz 1H, H-10), 7.44 (*virt*. td, $^{3}J \approx {}^{3}J$ = 7.5 Hz, ^{4}J = 1.0 Hz, 1H, H-8), 7.51 (*virt*. td, $^{3}J \approx {}^{3}J$ = 7.5 Hz, ^{4}J = 1.0 Hz, 1H, H-9), 7.86 (*virt*. dt, ^{3}J = 7.5 Hz, $^{4}J \approx {}^{5}J$ = 1.0 Hz, 1H, H-7).

^{13}**C NMR** (126 MHz, $CDCl_3$): δ [ppm] = 166.4 (C-6), 146.5 (C-10a), 132.6 (C-6a), 131.1 (C-9), 128.1 (C-8), 123.9 (C-7), 121.7 (C-10), 53.9 (C-10b), 37.4 (C-1), 34.7 (C-4), 30.7 (C-3), 26.0 (C-2), 16.3 (CH_3).

HRMS (ESI) *m/z*: $[M+H]^+$ Calcd. for $C_{13}H_{16}NO$ 202.1226; Found 202.1229.

IR (ATR): $\tilde{\nu}$ [cm^{-1}] = 3049 (w), 2957 (w), 2324 (w), 2876 (w), 1675 (vs, C=O), 1617 (m, C=C), 1593 (w), 1457 (m), 1417 (s), 1287 (s), 1104 (m), 1007 (m), 756 (s), 734 (vs, sp^2 C–H), 689 (vs).

m.p. = 106-107 °C.

Significant NOE contacts:

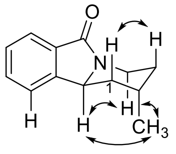

4.1.24. Attempted Catalytic Cyclization Reactions

In the course of our research, we have attempted to cyclize a number of substrates, but have been unsuccessful. Under optimized reaction conditions, the formation of a four-membered ring was not observed.

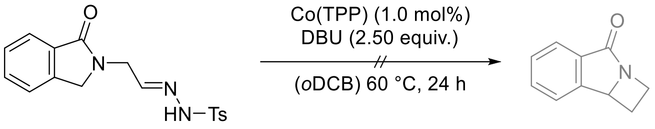

On the other hand, an extended linker length forming a seven-membered ring gave the hydrazone in insufficient yield. Attempted cyclization with the crude hydrazone or crude aldehyde did not yield any product.

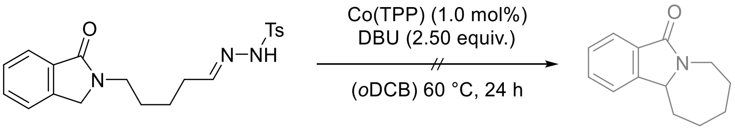

In a related context, the cyclization reaction of the methyl ketone hydrazone under the optimized conditions resulted in no product formation. Instead, the hydrazone was isolated in small amounts (<10%) after column chromatography. Although the hydrazone appeared to be more stable under the reaction conditions, no product was detected.

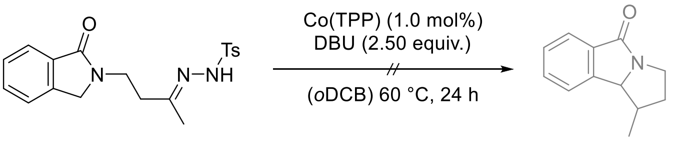

During this study, we tried some substitution patterns in the linker forming the five-membered ring. The hydrazone with a cyclopropyl moiety gave only a sluggish reaction with no evidence of cyclization or ring-opened products.

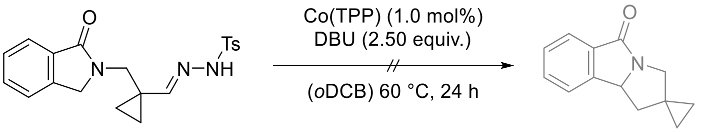

In addition, the substrate with a CF_2 group in the linker failed in due course. This became evident when the reaction mixture turned dark brown instead of copper after the addition of the base. We attribute this to the strong electron-withdrawing properties of the CF_2 group, which destabilizes the important intermediates.

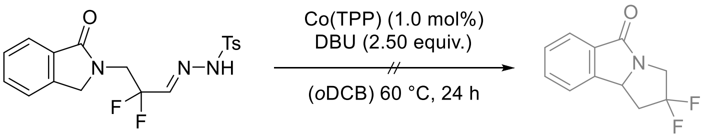

5. Deuteration Experiments

5.1. Substrate Synthesis

*5.1.1. 2-(2-(1,3-Dioxolan-2-yl)ethyl)isoindoline-1,3-dione (**SI-15**)*

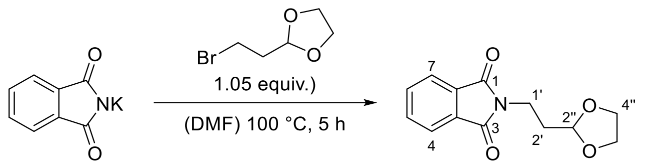

2-(2-Bromoethyl)-1,3-dioxolane (1.28 mL, 1.90 g, 10.5 mmol, 1.05 equiv) was added to a solution of potassium phthalimide (1.85 g, 10.0 g, 1.00 equiv.) in DMF (18.2 mL, 0,55 M) and the reaction mixture was heated to 100 °C for 5 h. After cooling to room temperature, the solvent was removed under reduced pressure. The residue was dissolved in CH_2Cl_2 (50 mL) and H_2O (50 mL) and the layers were separated. The aqueous layer was extracted with CH_2Cl_2 (2 × 50 mL). The combined organic layers were dried over Na_2SO_4, filtered and the solvent was removed under reduced pressure to obtain the alkylated phthalimide **SI-15** (2.47 g, 10.0 mmol, *quant.*) as an off-white solid.

***R*f** (Hex/EtOAc = 1/4): 0.73 [UV, $KMnO_4$].

^{1}H NMR (500 MHz, $CDCl_3$): δ [ppm] = 2.08 (td, ^3J = 6.9 Hz, ^3J = 4.4 Hz, 2H, H-2'), 3.78 – 3.83 (m, 2H, H^a-4'', H^a-5''), 3.85 (t, ^3J = 6.9 Hz, 2H, H-1'), 3.91 – 3.96 (m, 2H, H^b-4'', H^b-5''), 4.96 (t, ^3J = 4.4 Hz, 1H, H-2''), 7.68 – 7-72 (m, 2H, H-5, H-6), 7.82 – 7.86 (m, 2H, H-4, H-7).

^{13}C NMR (126 MHz, $CDCl_3$): δ [ppm] = 168.5 (C-1, C-3), 133.8 (C-5, C-6), 132.3 (C-3a, C-7a), 123.3 (C-4, C-7), 102.7 (C-1''), 65.1 (C-4'', C-5''), 33.3 (C-1'), 32.3 (C-2').

HRMS (ESI) *m/z*: $[M+H]^+$ Calcd. for $C_{13}H_{14}NO_4$ 248.0917; Found 248.0914.

IR (ATR): $\tilde{\nu}$ [cm^{-1}] = 2949 (w), 2886 (w), 1699 (vs, C=O), 1615 (w, C=C), 1394 (s), 1371 (s, O–C–O), 1123 (s), 1062 (m), 945 (m), 890 (s), 794 (w), 727 (s, sp^2 C–H), 713 (vs).

m.p. = 118-120 °C.

*5.1.2. 2-(2-(1,3-dioxolan-2-yl)ethyl)isoindoline-1,3-dione (**SI-2a**-d_2)*

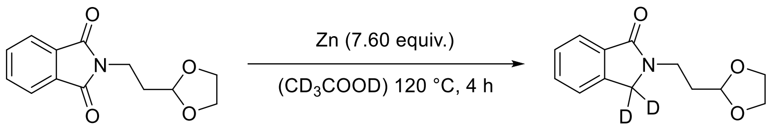

Zinc powder (1.12 g, 17.2 mmol, 7.60 equiv.) was added to a solution of phthalimide **SI-14** (560 mg, 2.26 mmol, 1.00 equiv.) in CD_3COOD (22.6 mL, 0.1 M) and the reaction mixture was stirred at 120 °C for 4 h. After cooling to room temperature, the mixture was quenched with sat. $NaHCO_3$ solution (50 mL) and diluted with CH_2Cl_2 (80 mL). The layers were separated and the organic layer was washed with sat. $NaHCO_3$ solution and brine (50 mL). The combined aqueous layers were extracted with CH_2Cl_2 (3 × 80 mL). The combined organic layers were dried over Na_2SO_4, filtered and the solvent was removed under reduced pressure. The crude product was purified by automated flash column chromatography (Hex/EtOAc) to yield the entitled Isoindolinone **SI-2a**-d_2 (170 mg), which already contained some deprotected aldehyde. Thus, the mixture was directly subjected to the deprotection method without further purification and analyzation.

*5.1.3. N'-(3-(1-Oxoisoindolin-2-yl-3,3-d_2)propylidene)-4-methylbenzenesulfonohydrazide-d_2 (**2a**-d_2)*

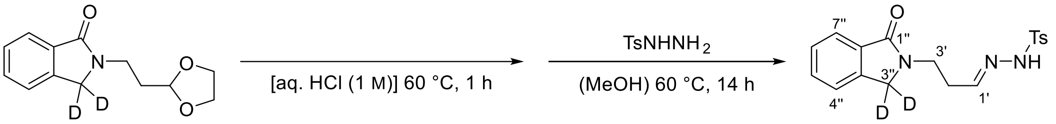

Following GP 6, the crude product **SI-2a**-d_2 (170 mg) was used to obtain the aldehyde (140 mg). The crude aldehyde was subjected to GP 7 to obtain the desired hydrazone **2a**-d_2 (163 mg, 453 µmol, 20% over three steps, 98% d_2) as a white solid.

***R*f** (CH_2Cl_2/ac = 9/1): 0.21 [UV, $KMnO_4$].

^{1}H NMR (500 MHz, DMSO-d_6): δ [ppm] = 2.31 (s, 3H, CH_3), 2.47 (td, ^3J = 6.7 Hz, ^3J = 5.1 Hz, 2H, H-2'), 3.61 (t, ^3J = 6.7 Hz, 2H, H-3'), 7.22 – 7.25 (m, 2H, H-3), 7.27 (t, ^3J = 5.1 Hz, 1H, H-1'), 7.48 (*virt.*td, $^3J \approx {}^3J$ = 7.6 Hz, ^4J = 1.0 Hz, 1H, H-6''), 7.53 (dd, ^3J = 7.6 Hz, ^4J = 1.0 Hz, 1H, H-4''), 7.58 – 7.62 (m, 3H, H-2, H-5''), 7.64 (*virt*. dt, ^3J = 7.5 Hz, $^4J \approx {}^5J$ = 1.0 Hz, 1H, H-7'') 11.05 (s, 1H, NH).

^{13}C NMR (126 MHz, DMSO-d_6): δ [ppm] = 167.8 (C-1''), 149.7 (C-1'), 143.6 (C-4), 142.2 (C-3a''), 136.7 (C-1), 132.8 (C-7a''), 131.7 (C-5''), 129.9 (C-3), 128.3 (C-6''), 127.4 (C-2), 123.8 (C-4''), 123.1 (C-7''), 49.4* (C-3''), 39.1 (C-3'), 31.6 (C-2'), 21.5 (CH_3).

*Signal of C-3'' was too weak to detect any C–D coupling.

HRMS (ESI) *m/z*: $[M+H]^+$ Calcd. for $C_{18}H_{18}D_2N_3O_3S$ 360.1345; Found 360.1342.

*5.1.4. 2-(3-Hydroxypropyl)isoindolin-1-one-3-d_1 (**SI-1a**-d_1)*

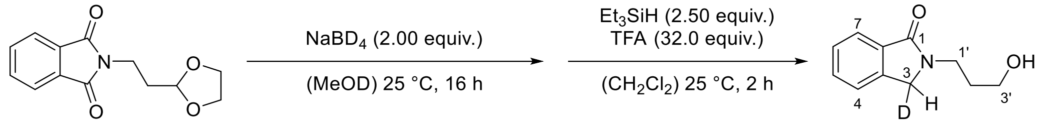

$NaBD_4$ (419 mg, 10.0 mmol, 2.00 equiv.) was added in portions to a solution of phthalimide **SI-14** (1.24 g, 5.00 mmol, 1.00 equiv.) in MeOD (10 mL, 0.5 M) and the reaction mixture was stirred at room temperature for 16 h. After removal of the solvent, the residue was dissolved in CH_2Cl_2 (50 mL) and H_2O (50 mL) and the layers were separated. The aqueous layer was extracted with CH_2Cl_2 (2 × 50 mL) and the combined organic layers were dried over Na_2SO_4, filtered and the solvent was removed under reduced pressure.

The crude product was redissolved in CH_2Cl_2 (25 mL, 0.2 M) and Et_3SiH (2.00 mL, 1.45 g, 12.5 mmol, 2.50 equiv.) and TFA (12.2 mL, 18.2 g, 160 mmol, 32.0 equiv.) were added sequentially. The reaction mixture was stirred at room temperature for 2 h. Afterwards, the reaction was quenched by addition of aq. NaOH (1 M, 50 mL) and the layers were separated. The aqueous layer was extracted with CH_2Cl_2 (2 × 50 mL). The combined organic layers were dried over Na_2SO_4, filtered and the solvent was removed under reduced pressure. The crude product was purified by automated flash column chromatography (Hex/EtOAc) to yield the entitled alcohol **SI-1a**-d_1 (263 mg, 1.37 mmol, 37%) as colorless oil.

***R*f** (EtOAc/MeOH = 9/1): 0.32 [UV, $KMnO_4$].

^{1}H NMR (500 MHz, $CDCl_3$): δ [ppm] = 1.84 (tt, ^3J = 6.8 Hz, ^3J = 5.6 Hz, 2H, H-2'), 3.32 (bs, 1H, OH), 3.58 (t, ^3J = 5.6 Hz, 2H, H-3'), 3.79 (t, ^3J = 6.8 Hz, 2H, H-1'), 4.39 (t, $^2J_{H–D}$ = 2.4 Hz, 1H, H-3), 7.44 – 7.50 (m, 2H, H-4, H-6), 7.55 (*virt*. td, $^3J \approx {}^3J$ = 7.5 Hz, ^4J = 1.2 Hz, 1H, H-5), 7.85 (*virt*. dt, ^3J = 7.6 Hz, $^4J \approx {}^5J$ = 1.2 Hz. 1H, H-7).

^{13}C NMR (126 MHz, $CDCl_3$): δ [ppm] = 170.0 (C-1), 141.2 (C-3a), 132.4 (C-7a), 131.7 (C-5), 128.4 (C-6), 123.9 (C-7), 122.9 (C-4), 58.3 (C-3'), 50.3 (t, $^1J_{C–D}$ = 21.6 Hz), 38.8 (C-1'), 30.9 (C-2').

HRMS (ESI) *m/z*: $[M+H]^+$ Calcd. for $C_{11}H_{13}DNO_2$ 193.1082; Found 193.1080.

*5.1.5. N'-(3-(1-oxoisoindolin-2-yl-3-d_1)propylidene)-4-methylbenzenesulfonohydrazide (**2a**-d_1)*

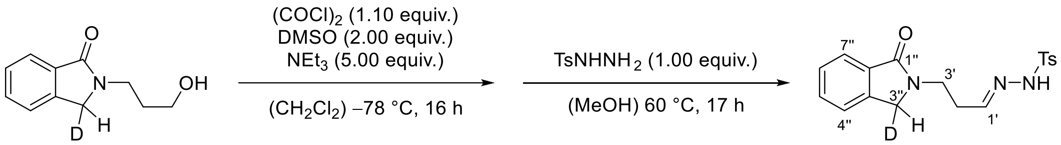

Following GP 5, the corresponding aldehyde (200 mg, 1.05 mmol, *quant*.) was obtained as a colorless oil.

Following GP 7, the hydrazone **2a**-d_1 (179 mg, 500 µmol, 50%,) was obtained as a colorless solid.

***R*f** (CH_2Cl_2/ac = 4/1): 0.21 [UV, $KMnO_4$].

^{1}H NMR (500 MHz, DMSO-d_6): δ [ppm] = 2.31 (s, 3H, CH_3), 2.47 (td, ^3J = 6.7 Hz, ^3J = 5.1 Hz, 2H, H-2'), 3.61 (t, ^3J = 6.7 Hz, 2H, H-3'), 4.32 (bs, 1H, H-3''), 7.22 – 7.25 (m, 2H, H-3), 7.27 (t, ^3J = 5.1 Hz, 1H, H-1'), 7.48 (*virt*.t, $^3J \approx {}^3J$ = 7.5 Hz, 1H, H-6''), 7.53 (dd, ^3J = 7.6 Hz, ^4J = 1.0 Hz, 1H, H-4''), 7.58 – 7.62 (m, 3H, H-2, H-5''), 7.64 (*virt*. dt, ^3J = 7.5 Hz, $^4J \approx {}^5J$ = 1.0 Hz, 1H, H-7''), 11.05 (s, 1H, NH).

^{13}C NMR (126 MHz, DMSO-d_6): δ [ppm] = 167.3 (C-1''), 149.3 (C-1'), 143.1 (C-4), 141.8 (C-3a''), 136.2 (C-1), 132.3 (C-7a''), 131.3 (C-5), 129.5 (C-3), 127.8 (C-6''), 127.0 (C-2), 123.4 (C-4''), 122.7 (C-7''), 49.3 (t, $^1J_{C\text{–}D}$ = 21.3 Hz, C-3''), 38.7 (C-3'), 31.1 (C-2'), 21.1 (CH_3).

HRMS (ESI) *m/z*: $[M+H]^+$ Calcd. for $C_{18}H_{19}DN_3O_3S$ 359.1283; Found 359.1276.

*5.1.6. 2-Methylisoindolin-1-one-3,3-d_2 (**3**-d_2)*

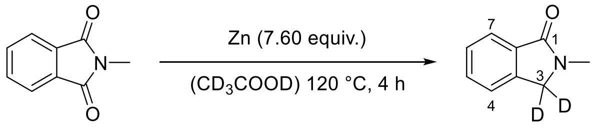

Zinc powder (1.24 g, 19.0 mmol, 7.60 equiv.) was added to a solution of *N*-methylphthalimide (403 mg, 2.50 mmol, 1.00 equiv.) in CD_3COOD (5.0 mL, 0.5 M) and the reaction mixture was stirred at 120 °C for 3 h. After cooling to room temperature, the mixture was quenched with aq. NaOH (1 M, 30 mL) and diluted with CH_2Cl_2 (50 mL). The layers were separated and aqueous layers was extracted with CH_2Cl_2 (2 × 50 mL). The combined organic layers were dried over Na_2SO_4, filtered and the solvent was removed under reduced pressure. The entitled isoindolinone **3**-d_2 (352 mg, 2.36 mmol, 94%, 97% d_2) was thus obtained as a white solid.

***R*f** (Hex/EtOAc = 2/3): 0.21 [UV, $KMnO_4$].

^{1}H NMR (500 MHz, $CDCl_3$): δ [ppm] = 3.20 (s, 3H, CH_3), 7.41 – 7.47 (m, 2H, H-4, H-6), 7.52 (*virt*. td, $^3J \approx {}^3J$ = 7.5 Hz, ^4J = 1.2 Hz, 1H, H-5), 7.84 (*virt*. dt, ^3J = 7.5 Hz, $^4J \approx {}^5J$ = 1.2 Hz, 1H, H-7).

^{13}C NMR (126 MHz, $CDCl_3$): δ [ppm] = 168.8 (C-1), 141.0 (C-3a), 133.1 (C-7a), 131.2 (C-5), 128.1 (C-6), 123.7 (C-7), 122.7 (C-4), 51.50 (pent, $^1J_{C-D}$ = 21.6 Hz, C-3), 30.0 (CH_3).

HRMS (ESI) *m/z*: $[M+H]^+$ Calcd. for $C_9H_8D_2NO$ 150.0882; Found 150.0880.

5.2. Deuteration Studies

*5.2.1. 1,2,3,9b-tetrahydro-5H-pyrrolo[2,1-a]isoindol-5-one-1,9b-d_2 (**1a**-d_2)*

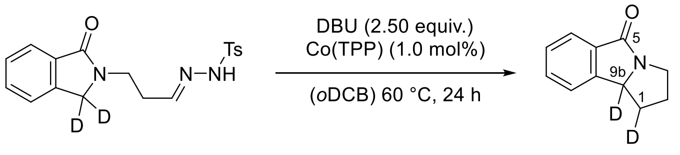

Following GP 8, compound **1a**-d_2 (12.5 mg, 71.3 µmmol, 71%) was isolated as colorless solid.

***R*f** (Hex/EtOAc = 1/1): 0.22 [UV, $KMnO_4$].

The product was isolated as a mixture of two diastereomers. For clarity, only one of the diastereomers is reported.

^{1}H NMR (500 MHz, $CDCl_3$): δ [ppm] = 1.19 – 1.28 (m, 0.7H, H^a-1), 2.25 – 2.41 (m, 2.4H, H^b-1, H-2), 3.38 – 3.45 (m, 1H, H^a-3), 3.68 – 3.76 (m, 1H, H^b-3), 7.40 – 7.47 (m, 2H, H-7, H-9), 7.51 (*virt*. t, $^3J \approx {}^3J$ = 7.4 Hz, 1H, H-8), 7.78 (d, ^3J = 7.4 Hz, 1H, H-6).

^{13}C NMR (126 MHz, $CDCl_3$): δ [ppm] = 171.8 (C-5), 146.5 (C-9a), 133.8 (C-5a), 131.7 (C-8), 128.5 (C-7), 124.1 (C-6), 122.8 (C-4), 64.4 (t, $^1J_{C–D}$ = 22.0 Hz, C-9b), 42.1 (C-3), 29.6 (t, $^1J_{C–D}$ = 20.5 Hz, C-1), 29.2 (C-2).

HRMS (ESI) *m/z*: $[M+H]^+$ Calcd. for $C_{11}H_{10}D_2NO$ 176.1039; Found 176.1037.

*5.2.2. 1,2,3,9b-tetrahydro-5H-pyrrolo[2,1-a]isoindol-5-one-9b-d_1 (**1a**-d_1)*

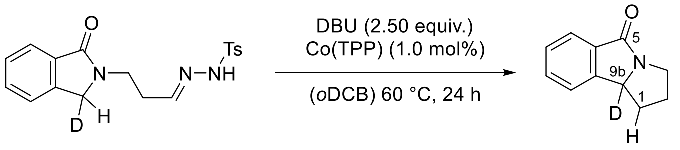

Following GP 8, compound **1a**-d_1 (14.1 mg, 80.9 µmmol, 81%, 82% *d*) was isolated as colorless solid.

***R*f** (Hex/EtOAc = 1/1): 0.22 [UV, $KMnO_4$].

^{1}H NMR (500 MHz, $CDCl_3$): δ [ppm] = 1.19 – 1.28 (m, 1H, H^a-1), 2.25 – 2.41 (m, 3H, H^b-1, H-2), 3.38 – 3.45 (m, 1H, H^a-3), 3.68 – 3.76 (m, 1H, H^b-3), 7.40 – 7.47 (m, 2H, H-7, H-9), 7.51 (*virt*. t, $^3J \approx {}^3J$ = 7.4 Hz, 1H, H-8), 7.78 (d, ^3J = 7.4 Hz, 1H, H-6).

^{13}C NMR (126 MHz, $CDCl_3$): δ [ppm] = 171.8 (C-5), 146.5 (C-9a), 133.8 (C-5a), 131.7 (C-8), 128.5 (C-7), 124.1 (C-6), 122.8 (C-4), 64.4 (t, $^1J_{C–D}$ = 22.0 Hz, C-9b), 42.0 (C-3), 29.6 (t, $^1J_{C–D}$ = 20.5 Hz, C-1), 29.2 (C-2).

*5.2.3. Deuteration Experiment of 2-Methylisoindolin-1-one-3,3-d_2 (**3**-d_2)*

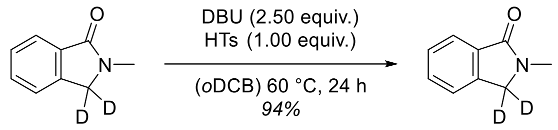

3-d_2 (97.2%), **3**-d_1 (2.8%) **3**-d_2 (91.1%), **3**-d_1 (8.4%), **3** (0.5%)

DBU (37.3 µL, 38.1 mg, 250 µmol, 2.50 equiv.) was added dropwise to a stirred solution of **3**-d_2 (14.9 mg, 100 µmol, 1.00 equiv., 97% d_2) and HTs (15.6 mg, 100 µmol, 1.00 equiv.) in *o*DCB (5.0 mL, 20 mM). The tube was sealed and stirred at 60 °C for 24 h. After cooling to room temperature, the solvent was removed under reduced pressure and the crude product was subjected to automated flash column chromatography to yield isoindolinone (14.0 mg, 93.8 µmol, 94% recovered) as white solid. NMR analysis showed a deterioration of deuterium (91.1% **3**-d_2, 8.4% **3**-d_1, 0.5% **3**).

***R*$_f$** (Hex/EtOAc = 2/3): 0.21 [UV, $KMnO_4$].

^{1}H NMR (500 MHz, $CDCl_3$): δ [ppm] = 1.19 – 1.28 (m, 0.7H, H^a-1), 2.25 – 2.41 (m, 2.4H, H^b-1, H-2), 3.38 – 3.45 (m, 1H, H^a-3), 3.68 – 3.76 (m, 1H, H^b-3), 7.40 – 7.47 (m, 2H, H-7, H-9), 7.51 (*virt*. t, $^3J \approx {}^3J$ = 7.4 Hz, 1H, H-8), 7.78 (d, ^3J = 7.4 Hz, 1H, H-6).

^{13}C NMR (126 MHz, $CDCl_3$): δ [ppm] = 171.8 (C-5), 146.5 (C-9a), 133.8 (C-5a), 131.7 (C-8), 128.5 (C-7), 124.1 (C-6), 122.8 (C-4), 64.4 (t, $^1J_{C-D}$ = 22.0 Hz, C-9b), 42.1 (C-3), 29.6 (t, $^1J_{C-D}$ = 20.5 Hz, C-1), 29.2 (C-2).

6. Application: Synthesis of Lennoxamine

*6.1.1. Ethyl 6-(chloromethyl)-2,3-dimethoxybenzoate (**SI-16**)*

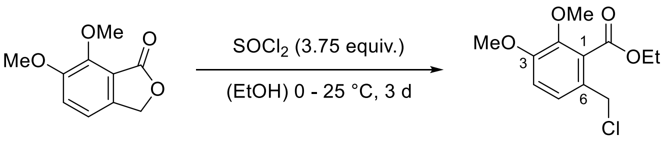

Based on a modified procedure by *Yan et al.*,[13] $SOCl_2$ (1.44 mL, 2.36 g, 19.88 mmol, 3.75 equiv.) was added dropwise to a suspension of meconin (1.03 g, 5.30 mmol, 1.00 equiv.) in EtOH (4.24 mL, 1.25 M) at 0 °C. After the addition, the cooling bath was removed and the resulting solution as stirred at 25 °C for 3 d. The reaction was quenched by addition of H_2O (10 mL) and sat. $NaHCO_3$ solution (20 mL). The aqueous layer was extracted with EtOAc (3 × 40 mL). The combined organic layers were washed with brine (30 mL), dried over Na_2SO_4, filtered and the solvent was removed under reduced pressure. The crude product was subjected to automated flash column chromatography (Hex/EtOAc) to yield the entitled benzoate **SI-16** (796 mg, 3.25 mmol, 61%) as a colorless oil alongside with recovered starting material (393 mg, 2.02 mmol, 38% recovered).

***R*f** (Hex/EtOAc = 4/1): 0.40 [UV, $KMnO_4$].

^{1}H NMR (500 MHz, $CDCl_3$): δ [ppm] = 1.41 (t, ^3J = 7.1 Hz, 3H, CH_3), 3.88 (s, 3H, OMe), 3.88 (s, 3H, OMe), 4.44 (q, ^3J = 7.1 Hz, 2H, OCH_2), 4.61 (s, 2H, CH_2Cl), 6.92 (d, ^3J = 8.5 Hz, 1H, H-), 7.11 (d, ^3J = 8.5 Hz, 1H, H-).

^{13}C NMR (126 MHz, $CDCl_3$): δ [ppm] = 166.9 (CO), 153.2 (C-3), 146.8 (C-2), 129.2 (C-6), 127.5 (C-1), 126.0 (C-5), 113.4 (C-4), 61.8 (OCH_2), 43.8 (CH_2Cl), 14.4 (CH_3).

Spectral data matched those reported in the literature.[13]

*6.1.2. 2-(Benzo[d][1,3]dioxol-5-yl)ethan-1-ammonium chloride (**SI-17**)*

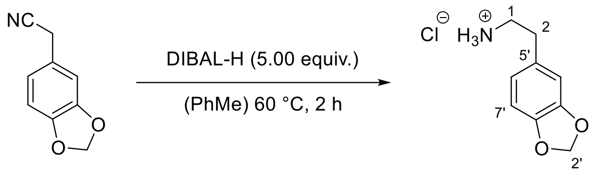

Based on a modified procedure by *Vinogradova et al.*,[22] a solution of 2-(benzo[d][1,3]dioxol-5-yl)acetonitrile (645 mg, 4.00 mmol, 1.00 equiv.) in THF (4 mL, 1 M) was added dropwise to a solution of DIBAL-H (1 M in PhMe, 20 mL, 20 mmol, 5.00 equiv.). The reaction mixture was then heated to 60 °C for 2 h. After cooling to r.t., the reaction was quenched by addition of EtOH (10 mL) and aq. KOH (20 wt%, 50 mL). The aqueous layer was extracted with EtOAc (3 × 50 mL). the combined organic layers were washed with brine (50 mL), dried over Na_2SO_4, filtered and the solvent was removed under reduced pressure. The residue was dissolved in acetone (50 mL) and filtered and the filtrate was treated with aq. HCl (37%, pH = 2). The resulting precipitate was collected and dried *in vacuo* to yield the entitled ammonium chloride **SI-17** (668 mg, 3.31 mmol, 83%) as a white solid.

^{1}H NMR (500 MHz, DMSO-d_6): δ [ppm] = 2.78 – 2.85 (m, 2H, H-2), 2.96 (t, ^3J = 7.7 Hz, 2H, H-1), 5.98 (s, 2H, H-2’), 6.71 (dd, ^3J = 8.0 Hz, ^4J = 1.7 Hz, 1H, H-6’), 6.83 – 6.88 (m, 2H, H-4, H-7’), 7.95 – 8.35 (bs, 3H, NH_3).

^{13}C NMR (126 MHz, $CDCl_3$): δ [ppm] = 147.4 (C-3a’), 146.0 (C-7a’), 131.1 (C-5’), 121.8 (C-6’), 109.1 (C-4’), 108.4 (C-7’), 100.9 (C-2’), 40.0 (C-1), 32.6 (C-2).

Spectral data matched those reported in the literature.[23]

*6.1.3. 2-(2-(Benzo[d][1,3]dioxol-5-yl)ethyl)-6,7-dimethoxyisoindolin-1-one (**SI-18**)*

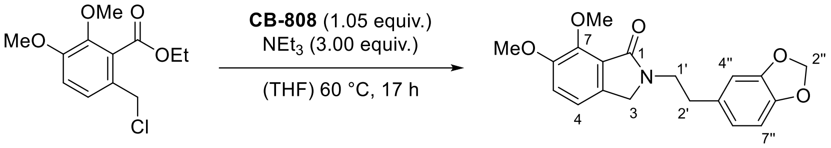

Based on a modified procedure by *Sahakitpichan et al.*,[24] ammonium chloride **SI-17** (562 mg, 2.79 mmol, 1.05 equiv.) and NEt_3 (1.11 mL, 807 mg, 7.97 mmol, 3.00 equiv.) were added sequentially to a solution of benzoate **SI-16** (650 mg, 2.66 mmol, 1.00 equiv.) in THF (5.3 ml, 500 mM) and the reaction mixture was stirred at 60 °C for 17 h. The reaction was quenched by addition of H_2O (30 mL) and the aqueous layer was extracted with CH_2Cl_2 (3 × 30 mL). The combined organic layers were dried over Na_2SO_4, filtered and the solvent was removed under reduced pressure. The crude product was subjected to automated flash column chromatography to yield the entitled isoindolinone **SI-18** (432 mg, 1.26 mmol, 48%) as a white solid.

***R*f** (Hex/EtOAc = 1/1): 0.29 [UV, $KMnO_4$].

^{1}H NMR (500 MHz, $CDCl_3$): δ [ppm] = 2.89 (t, ^3J = 7.3 Hz, 2H, H-2'), 3.76 (t, ^3J = 7.3 Hz, 2H, H-1'), 3.89 (s, 3H, C-6-OMe), 4.09 (s, 3H, C-7-OMe), 4.13 (d, ^4J = 0.9 Hz, 2H, H-3), 5.92 (s, 2H, H-2''), 6.67 (dd, ^3J = 7.9 Hz, ^4J = 1.7 Hz, 1H, H-6''), 6.72 (d, ^3J = 7.9 Hz, 1H, H-7''), 6.73 (d, ^4J = 1.7 Hz, 1H, H-4''), 7.01 (dt, ^3J = 8.1 Hz, ^4J = 0.9 Hz, 1H, H-4), 7.06 (d, ^3J = 8.1 Hz, 1H, H-5).

^{13}C NMR (126 MHz, $CDCl_3$): δ [ppm] = 166.8 (C-1), 152.4 (C-6), 147.9 (C-3a''), 147.3 (C-7a''), 146.3 (C-7), 134.6 (C-3a), 132.8 (C-5''), 125.3 (C-7a), 121.8 (C-6''), 117.8 (C-5), 116.3 (C-4), 109.2 (C-4''), 108.5 (C-7''), 101.0 (C-2''), 62.7 (C-6''-O*Me*), 56.9 (C-7''-O*Me*), 49.8 (C-3), 44.5 (C-1'), 34.7C (C-2').

Spectral data matched those reported in the literature.[24]

*6.1.4. 6-(2-(6,7-dimethoxy-1-oxoisoindolin-2-yl)ethyl)benzo[d][1,3]dioxole-5-carbaldehyde (**SI-4t**)*

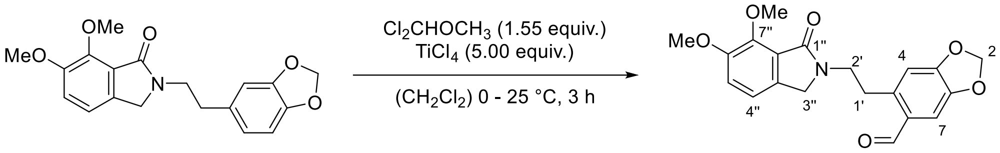

Based on a modified procedure by *Sahakitpichan et al.*,[24] $TiCl_4$ (0.33 mL, 569 mg, 3.00 mmol, 5.00 equiv.) was added to a solution of isoindolinone **SI-18** (204 mg, 1.00 mmol, 1.00 equiv.) and Cl_2CHOCH_3 (0.08 ml, 107 mg, 930 µmol, 1.55 equiv.) in CH_2Cl_2 (6.9 ml, 87 mM) at 0 °C. The reaction mixture was kept at this temperature for further 15 min and then stirred at 25 °C for 3 h. The reaction was quenched by pouring onto ice and the aqueous layer was extracted with CH_2Cl_2 (3 × 50 mL). The combined organic layers were dried over Na_2SO_4, filtered and the solvent was removed under reduced pressure. The crude product was subjected to automated flash column chromatography to yield the entitled isoindolinone **SI-4t** (187 mg, 506 µmol, 84%) as a white solid.

***R*f** (Hex/EtOAc = 1/1): 0.18 [UV, $KMnO_4$].

^{1}H NMR (500 MHz, $CDCl_3$): δ [ppm] = 3.30 – 3.35 (m, 2H, H-1'), 3.72 – 3.76 (m, 2H, H-2'), 3.89 (s, 3H, C-7''-OMe), 4.09 (s, 3H, C-6''-OMe), 4.28 (s, 2H, H-3''), 6.04 (s, 2H, H-2), 6.82 (s, 1H, H-4), 7.05 (d, ^3J = 8.1 Hz, 1H, H-5''), 7.08 (d, ^3J = 8.1 Hz, 1H, H-4''), 7.27 (s, 1H, H-7), 10.08 (s, 1H, CHO).

^{13}C NMR (126 MHz, $CDCl_3$): δ [ppm] = 190.2 (CHO), 166.8 (C-1''), 152.4 (C-7''), 152.3 (C-3a), 147.2 (C-6''), 147.1 (C-7a), 138.6 (C-5), 134.5 (C-3a''), 128.5 (C-6), 125.0 (C-7a''), 117.8 (C-5''), 116.4 (C-4''), 111.4 (C-4), 111.3 (C-7), 102.1 (C-2), 62.6 (C-6''-O*Me*), 56.8 (C-7''-O*Me*), 49.7 (C-3''), 44.4 (C-2'), 31.5 (C-1').

Spectral data matched those reported in the literature.[24]

*6.1.5. N'-((6-(2-(6,7-dimethoxy-1-oxoisoindolin-2-yl)ethyl)benzo[d][1,3]dioxol-5-yl)methylene)-4-methylbenzenesulfonohydrazide (**2t**)*

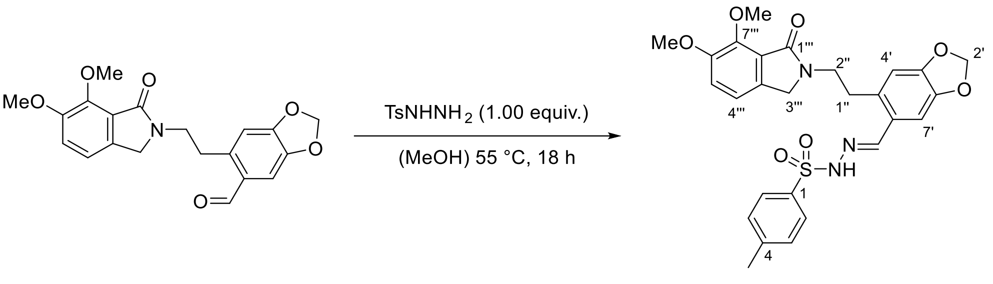

Following GP7, hydrazide **2t** was obtained as a colorless solid (148 mg, 275 µmol, 60%).

***R*f** (CH_2Cl_2/ac = 9/1): 0.26 [UV, $KMnO_4$].

^{1}H NMR (500 MHz, DMSO-*d*6): δ [ppm] = 2.35 (s, 3H, CH_3), 2.88 (t, ^3J = 7.4 Hz, 2H, H-1''), 3.51 (t, ^3J = 7.4 Hz, 2H, H-2''), 3.81 (s, 3H, C-6'''-OMe), 3.84 (s, 3H, C-7'''-OMe), 4.25 (s, 2H, H-3'''), 6.00 (s, 2H, H-2'), 6.81 (s, 1H, H-4'), 7.06 (s, 1H, H-7'), 7.18 (d, ^3J = 8.2 Hz, 1H, H-5'''), 7.25 (d, ^3J = 8.2 Hz, 1H, H-4'''), 7.36 – 7.41 (m, 2H, H-3), 7.73 – 7.77 (m, 2H, H-2), 8.15 (s, 1H, CHN), 11.31 (s, 1H, NH).

^{13}C NMR (126 MHz, $CDCl_3$): δ [ppm] = 165.5 (C-1'''), 151.7 (C-7'''), 148.9 (C-3a'), 146.4 (C-6'''), 146.0 (CHN), 145.2 (C-7a'), 143.4 (C-4), 136.1 (C-1), 134.8 (C-3a'''), 133.2 (C-5'), 129.7 (C-3), 127.3 (C-2), 125.3 (C-6'), 124.4 (C-7a'''), 118.4 (C-4'''), 116.7 (C-5'''), 110.1 (C-4'), 104.7 (C-7'), 101.5 (C-2'), 61.7 (C-7'''-O*Me*), 56.4 (C-6'''-O*Me*), 48.8 (C-3'''), 43.4 (C-2''), 30.5 (C-1''), 21.0 (CH_3).

HRMS (ESI) *m/z*: $[M+H]^+$ Calcd. for $C_{27}H_{38}N_3O_7S$ 538.1642; Found 538.16353.

IR (ATR): $\tilde{\nu}$ [cm^{-1}] = 2901 (w), 1660 (s, C=O), 1596 (w, C=C), 1493 (s), 1483 (s), 1463 (s), 1355 (m), 1326 (m), 1265 (vs, ArOMe), 1162 (vs), 1037 (vs, ArOMe), 934 (s), 872 (m, sp^2 C–H), 813 (s, sp^2 C–H), 755 (s), 662 (vs).

m.p. = > 136 °C (decomposition).

*6.1.6. 9,10-Dimethoxy-5,6,12b,13-tetrahydro-8H-[1,3]dioxolo[4'',5'':4',5']benzo[1',2':4,5]azepino[2,1-a]isoindol-8-one (Lennoxamine, **1t**)*

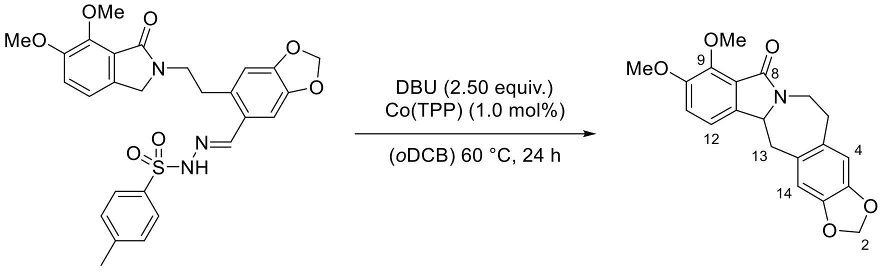

Following GP 8, Lennoxamine (**1t**) was obtained as a colorless solid (33.5 mg, 94.8 µmol, 95%).

***R*f** (Hex/EtOAc = 1/1): 0.24 [UV, $KMnO_4$].

^{1}H NMR (500 MHz, $CDCl_3$): δ [ppm] = 2.78 – 2.86 (m, 2H, H^a-5, H^a-13), 2.88 – 2.96 (m, 2H, H^b-5, H^a-6), 3.10 (dd, ^2J = 14.7 Hz, ^3J = 1.7 Hz, 1H, H^b-13) 3.91 (s, 3H, C-10-OMe), 4.10 (s, 3H, C-9-OMe), 4.29 (dd, ^3J = 10.8 Hz, ^3J = 1.7 Hz, H-12b), 4.70 – 4.79 (m, 1H, H^b-6), 5.95 (d, ^2J = 1.5 Hz, 1H, H^a-2), 5.96 (d, ^2J = 1.5 Hz, 1H, H^b-2), 6.70 (s, 1H, H-4), 6.77 (s, 1H, H-14), 7.12 (d, ^3J = 8.2 Hz, 1H, H-11), 7.16 (dd, ^3J = 8.2 Hz, ^4J = 0.8 Hz, 1H, H-12).

^{13}C NMR (126 MHz, $CDCl_3$): δ [ppm] = 165.3 (C-8), 152.8 (C-10), 147.3 (C-9), 146.5 (C-3a*), 146.2 (C-14a*), 138.3 (C-12a), 135.0 (C-4a), 131.1 (C-13a), 124.3 (C-8a), 117.3 (C-12), 116.3 (C-11), 110.5 (2C, C-4, C-14), 101.2 (C-2), 62.7 (C-9-OMe), 60.3 (C-12b), 56.9 (C-10-OMe), 42.9 (C-13), 41.3 (C-6), 36.1 (C-5).

*Signals are interchangeable.

Spectral data matched those reported in the literature.[25]

7. Crystallographic Data

SC-XRD structure report for compound 1x.

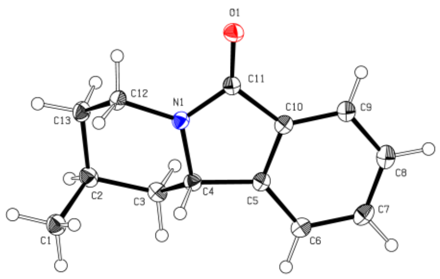

Figure SI-1 *Molecular structure of **1x** in the solid-state. ADPs are given at the 50% probability level.*

A yellow, block shaped crystal of $C_{13}H_{15}NO$ coated with perfluorinated ether and fixed on top of a Kapton micro sampler was used for X-ray crystallographic analysis. The X-ray intensity data were collected at 100(2) K on a Bruker D8 VENTURE Duo three-angle diffractometer with an IMS microsource with MoK_α radiation (λ=0.71073 Å) using APEX4.[26] The diffractometer was equipped with a Helios optic monochromator, a Bruker PHOTON II detector, and an Oxford Cryostreamlow temperature device.
A matrix scan was used to determine the initial lattice parameters. All data were integrated with the Bruker SAINT V8.40B software package using a narrow-frame algorithm and the reflections were corrected for Lorentz and polarisation effects, scan speed, and background.[27] The integration of the data using a monoclinic unit cell yielded a total of 45744 reflections within a 2θ range [°] of 5.08 to 50.70 (0.83 Å), of which 1923 were independent. Data were corrected for absorption effects including odd and even ordered spherical harmonics by the multi-scan method (SADABS 2016/2).[28] Space group assignment was based upon systematic absences, E statistics, and successful refinement of the structure.
The structure was solved by direct methods using SHELXT and refined by full-matrix least-squares methods against F^2 by minimizing $\Sigma w(F_o^2 - F_c^2)^2$ using SHELXL in conjunction with SHELXLE.[29-31] All non-hydrogen atoms were refined with anisotropic displacement parameters. Hydrogen atoms were refined isotropically on calculated positions using a riding model with their U_{iso} values constrained to 1.5 times the U_{eq} of their pivot atoms for terminal sp^3 carbon atoms and a C–H distance of 0.98 Å. Non-methyl hydrogen atoms were refined using a riding model with methylene, aromatic, and other C–H distances of 0.99 Å, 0.95 Å, and 1.00 Å, respectively, and U_{iso} values constrained to 1.2 times the U_{eq} of their pivot atoms.
Neutral atom scattering factors for all atoms and anomalous dispersion corrections for the non-hydrogen atoms were taken from International Tables for Crystallography.[32] Crystallographic data for the structures reported in this paper have been deposited with the Cambridge Crystallographic Data Centre.[33] Supplementary crystallographic data reported in this paper have been deposited with the Cambridge Crystallographic Data Centre (CCDC 2363807) and can be obtained free of charge from The Cambridge Crystallographic Data Centre via www.ccdc.cam.ac.uk/structures.[33] This report and the CIF file were generated using FinalCif.[34]

Table 1. Crystal data and structure refinement for compound 1x.

CCDC number	2363807
Empirical formula	$C_{13}H_{15}NO$
Formula weight	201.26
Temperature [K]	100(2)
Crystal system	monoclinic
Space group (number)	$P2_1/n$ (14)
a [Å]	8.7949(4)
b [Å]	10.0299(4)
c [Å]	12.3982(5)
α [°]	90
β [°]	106.019(2)
γ [°]	90
Volume [$Å^3$]	1051.20(8)
Z	4
ρ_{calc} [gcm^{-3}]	1.272
μ [mm^{-1}]	0.080
F(000)	432
Crystal size [mm^3]	0.112×0.174×0.382
Crystal colour	yellow
Crystal shape	block
Radiation	MoK_α (λ=0.71073 Å)
2θ range [°]	5.08 to 50.70 (0.83 Å)
Index ranges	$-10 \le h \le 10$<br>$-12 \le k \le 12$<br>$-14 \le l \le 14$
Reflections collected	45744
Independent reflections	1923<br>R_{int} = 0.0463<br>R_{sigma} = 0.0147
Completeness to θ = 25.242°	100.0
Data / Restraints / Parameters	1923 / 0 / 137
Goodness-of-fit on F^2	1.173
Final *R* indexes [$I \ge 2\sigma(I)$]	R_1 = 0.0527<br>wR_2 = 0.1053
Final *R* indexes [all data]	R_1 = 0.0637<br>wR_2 = 0.1159
Largest peak/hole [$eÅ^{-3}$]	0.23/−0.27

8. NMR Spectra of New Compounds

8.1. Isoindolinone derivatives

*8.1.1. 2-(3-Hydroxy-2,2-dimethylpropyl)isoindolin-1-one (**SI-1b**)*

^{1}H NMR (500 MHz, $CDCl_3$):

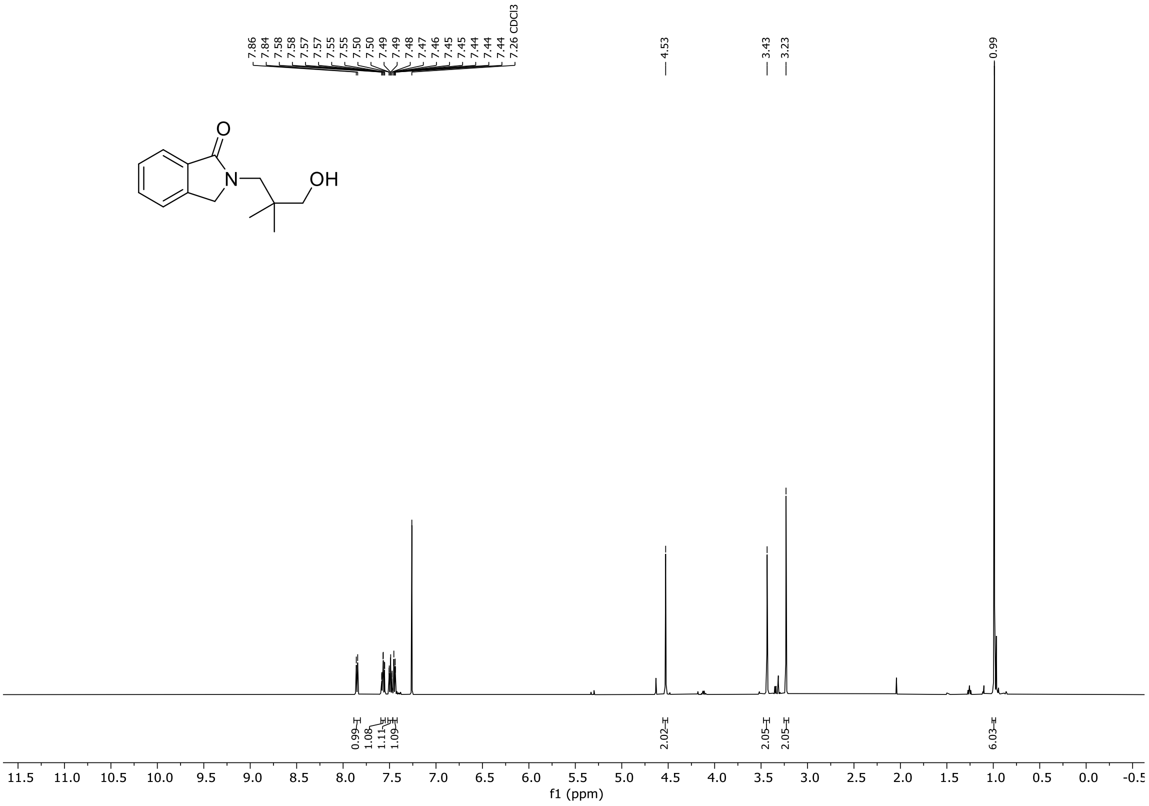

^{13}C NMR (126 MHz, $CDCl_3$):

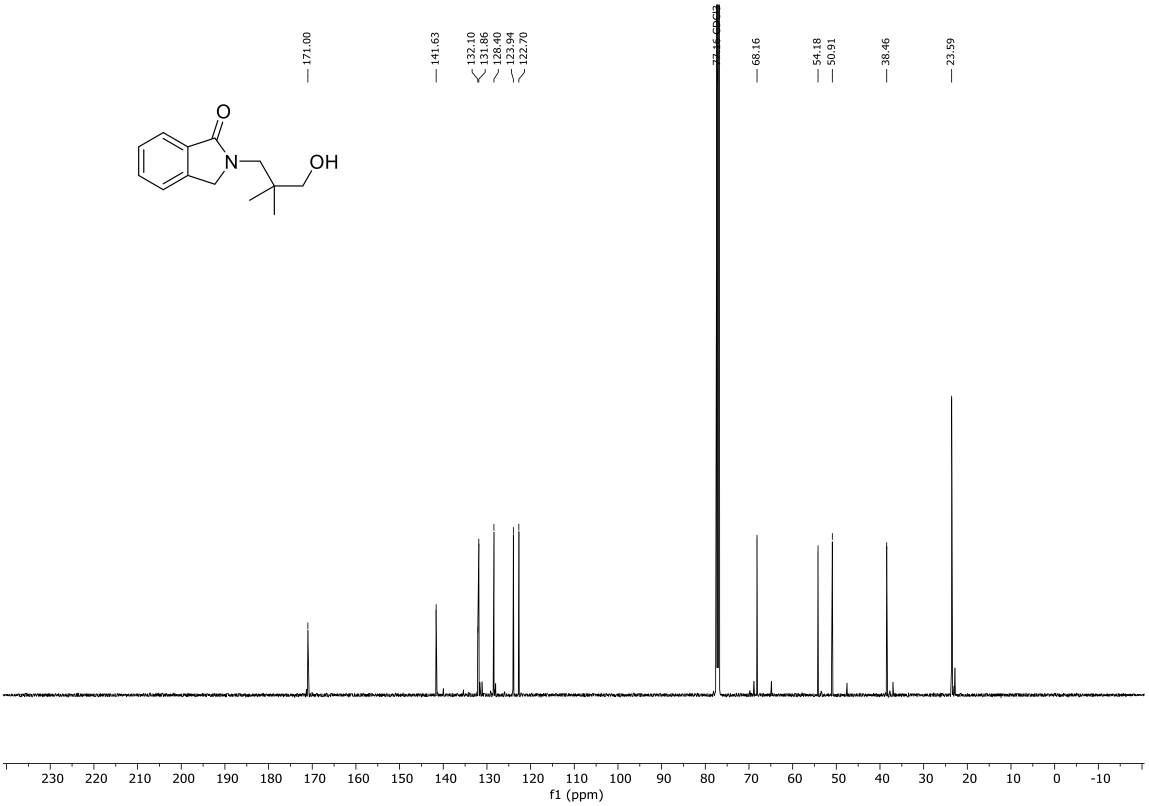

*8.1.2. 6-Chloro-2-(3-hydroxypropyl)isoindolin-1-one (**SI-1e**)*

^{1}H NMR (500 MHz, $CDCl_3$):

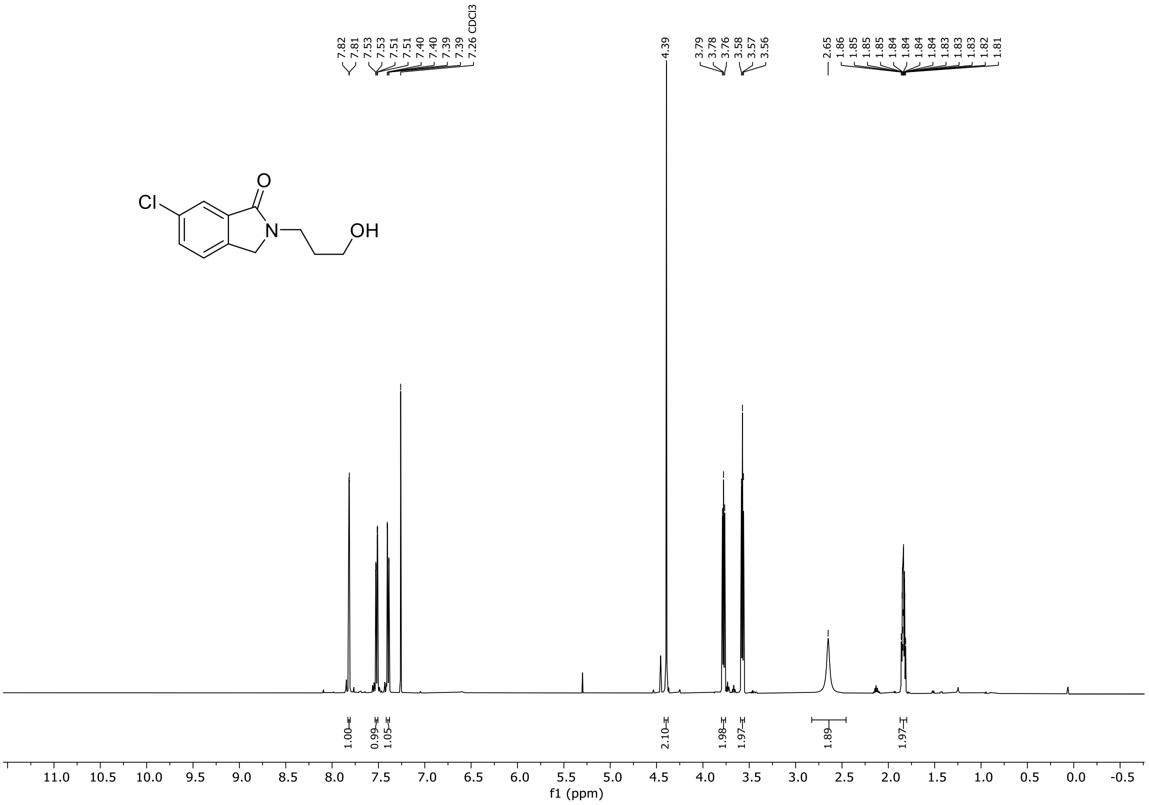

^{13}C NMR (126 MHz, $CDCl_3$):

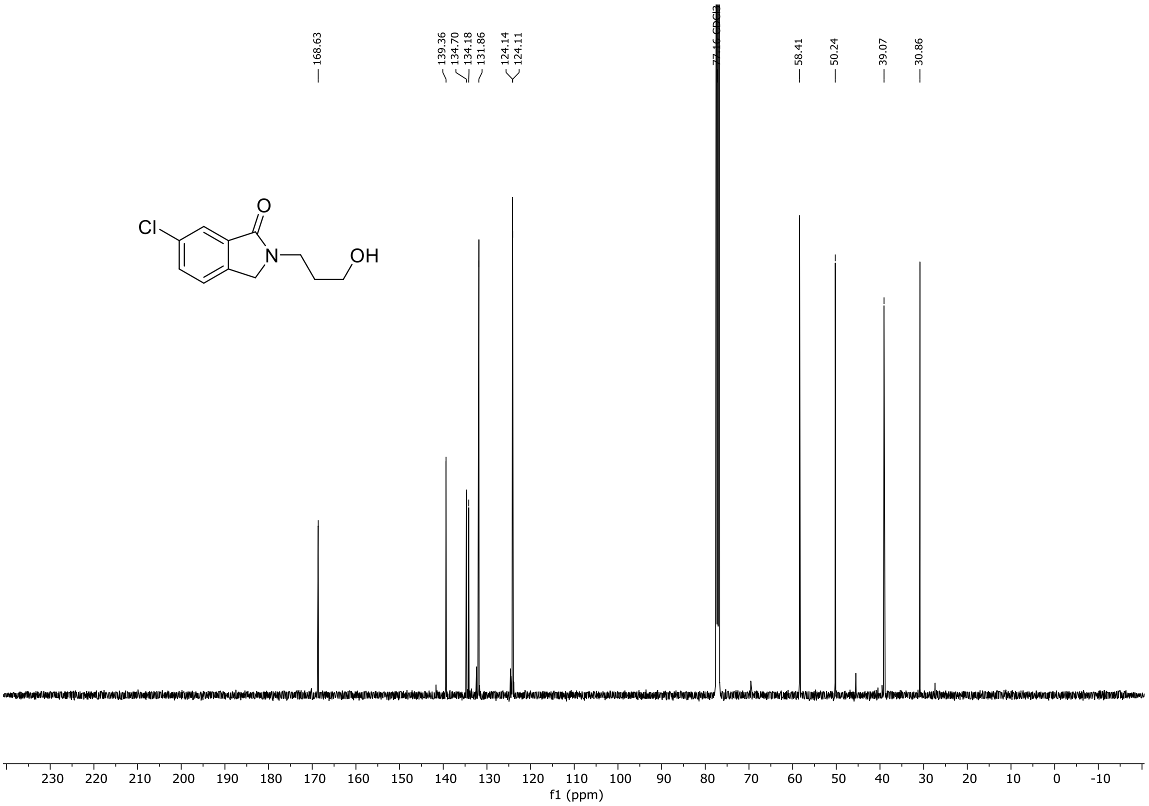

*8.1.3. 6-Bromo-2-(3-hydroxypropyl)isoindolin-1-one (**SI-1f**)*

^{1}H NMR (500 MHz, $CDCl_3$):

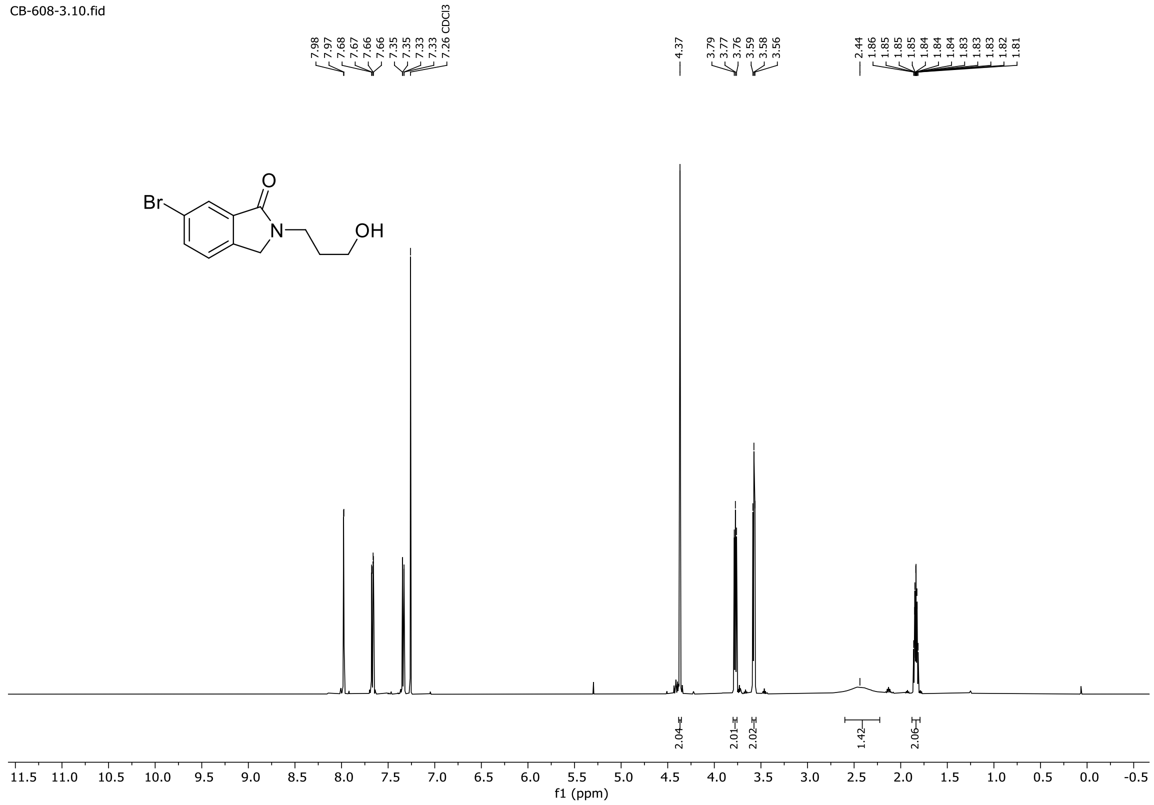

^{13}C NMR (126 MHz, $CDCl_3$):

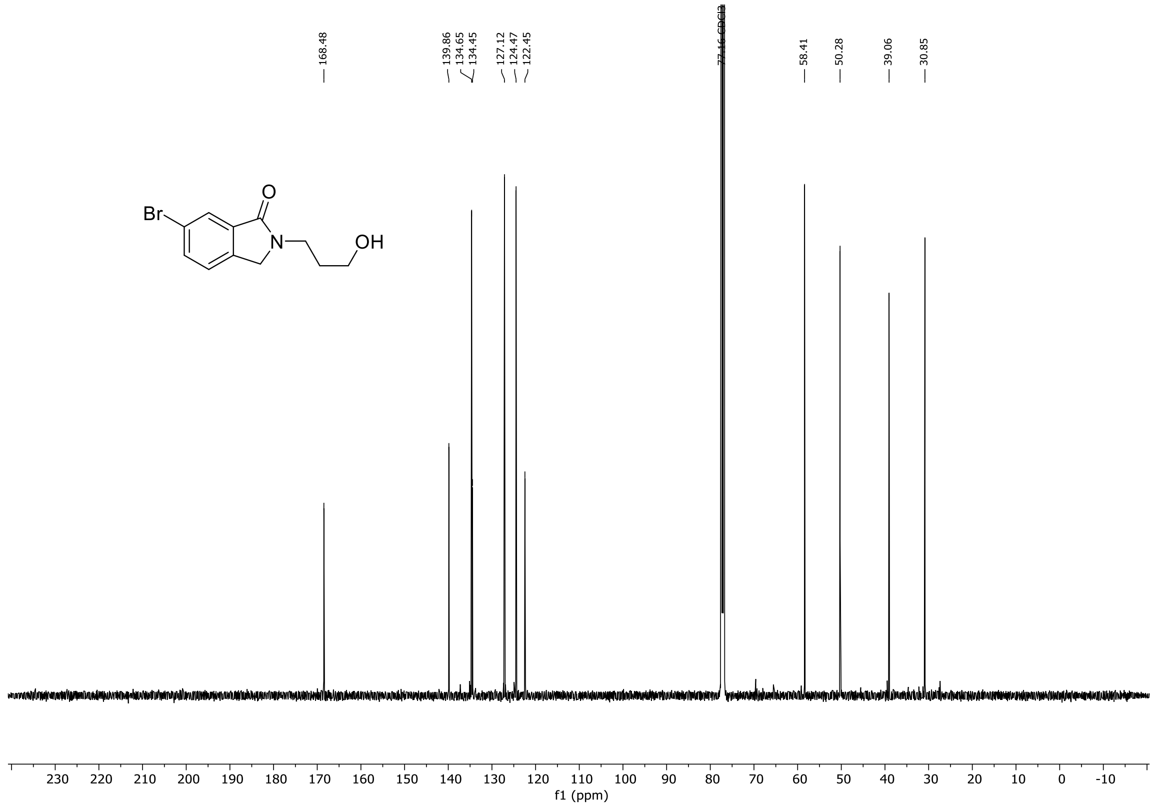

*8.1.4. 4-Methoxy-2-(3-hydroxypropyl)isoindolin-1-one (**SI-1l**)*

^{1}H NMR (500 MHz, $CDCl_3$):

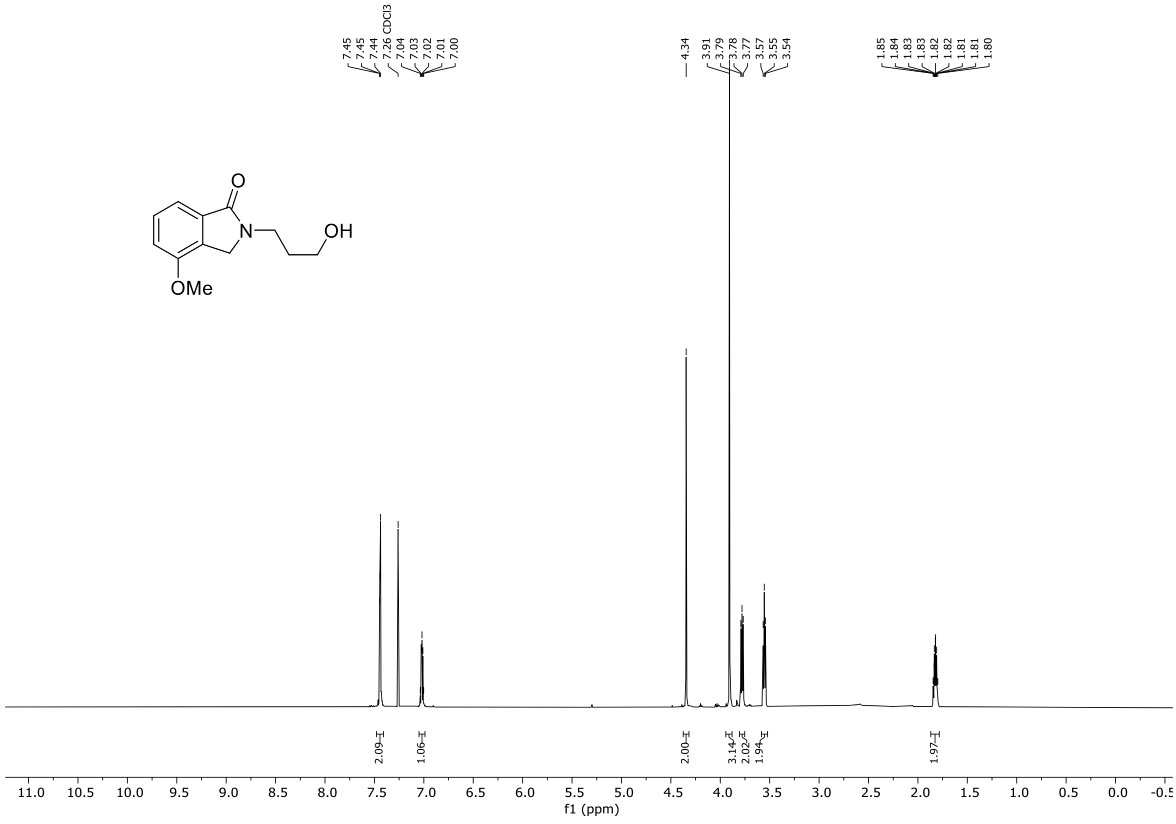

^{13}C NMR (126 MHz, $CDCl_3$):

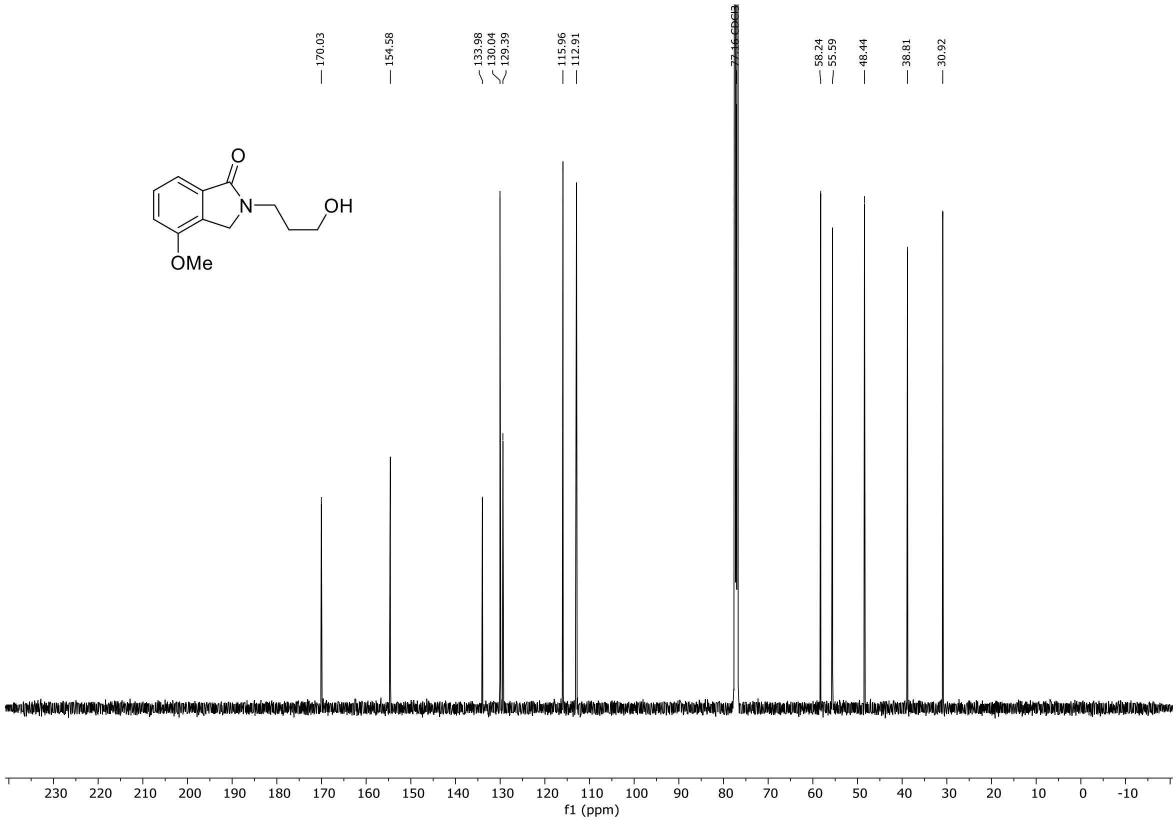

*8.1.5. 2-(4-Hydroxybutyl)isoindolin-1-one (**SI-1q**)*

^{1}H NMR (500 MHz, $CDCl_3$):

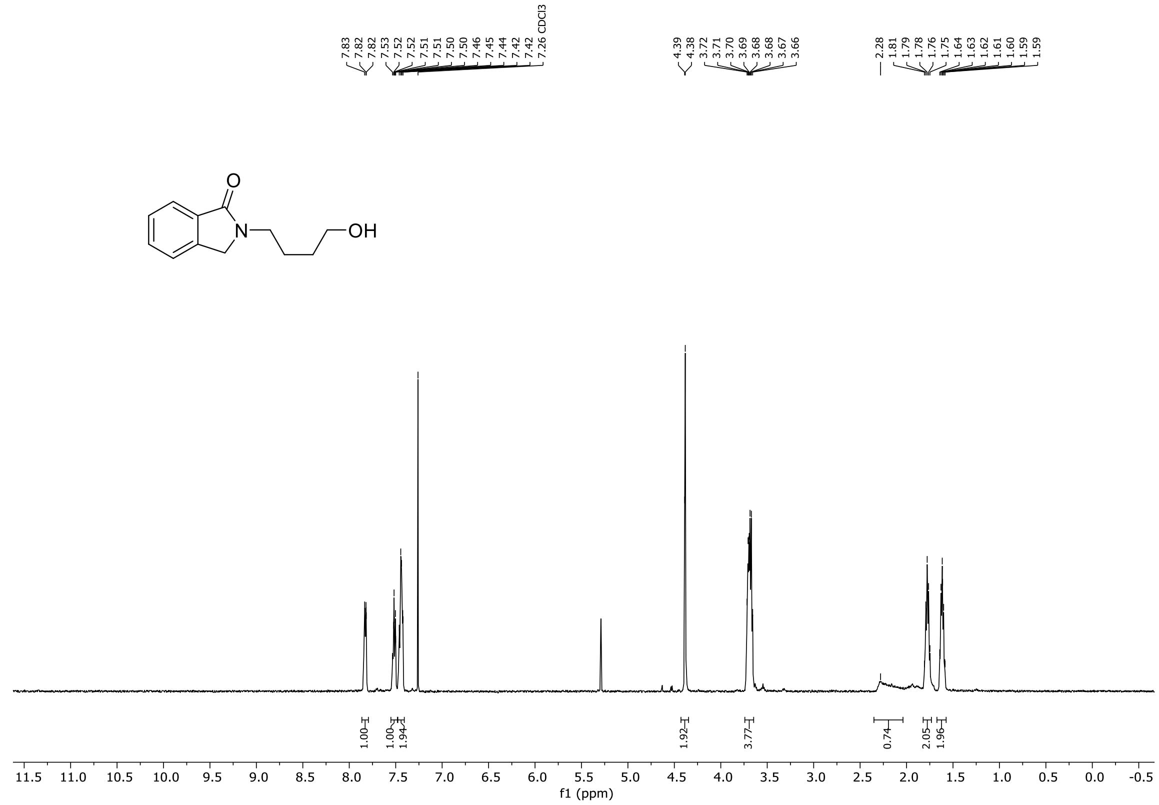

^{13}C NMR (126 MHz, $CDCl_3$):

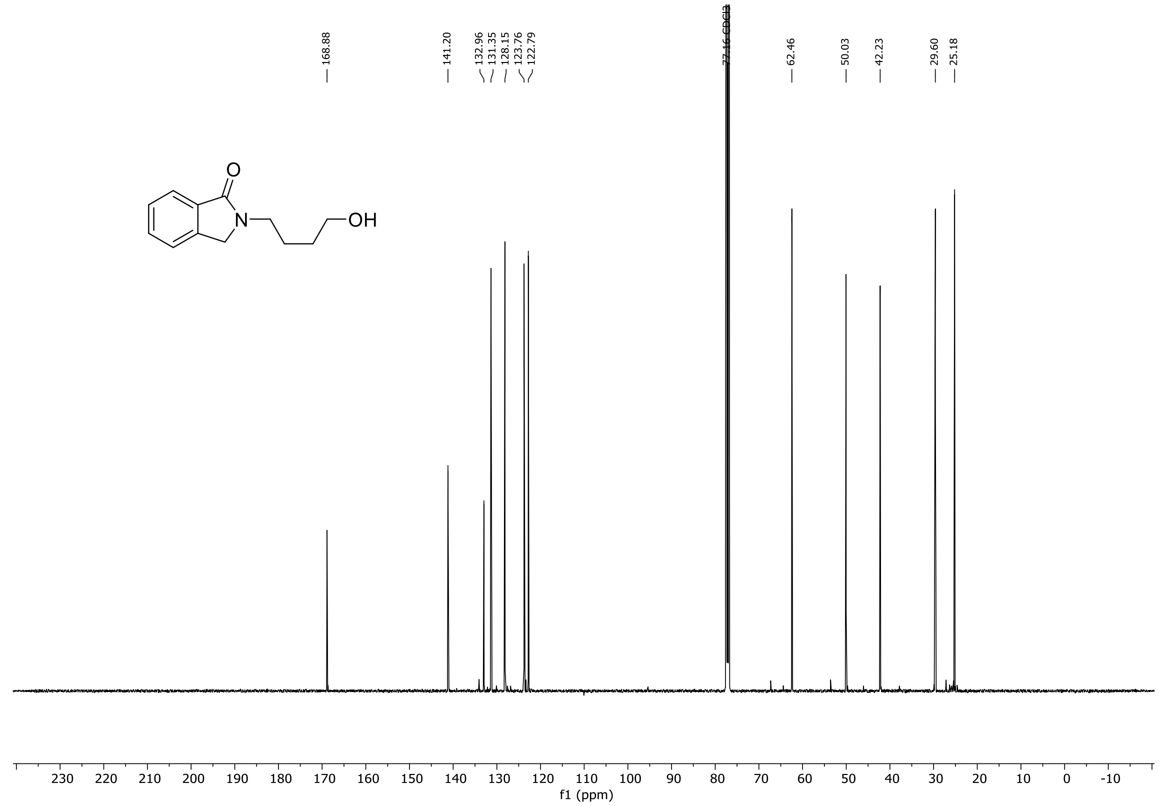

*8.1.6. 2-(4-Hydroxybutan-2-yl)isoindolin-1-one (**SI-1u**)*

^{1}H NMR (500 MHz, $CDCl_3$):

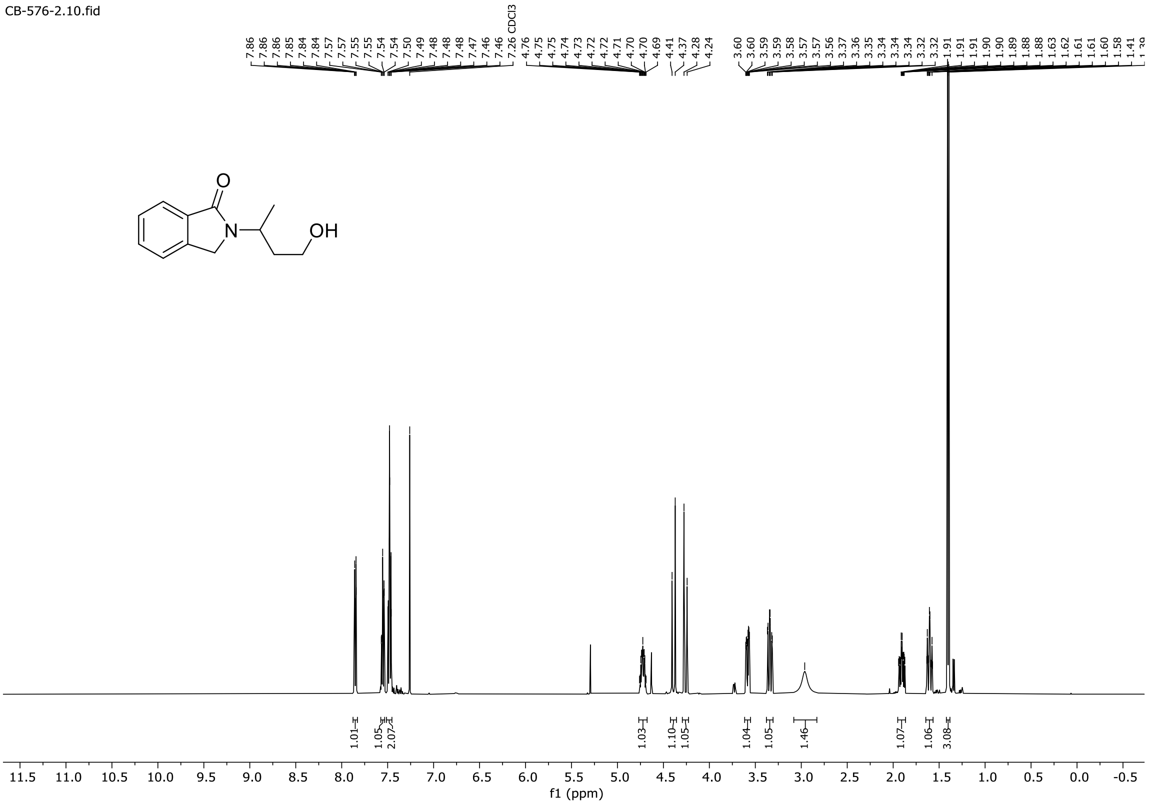

^{13}C NMR (126 MHz, $CDCl_3$):

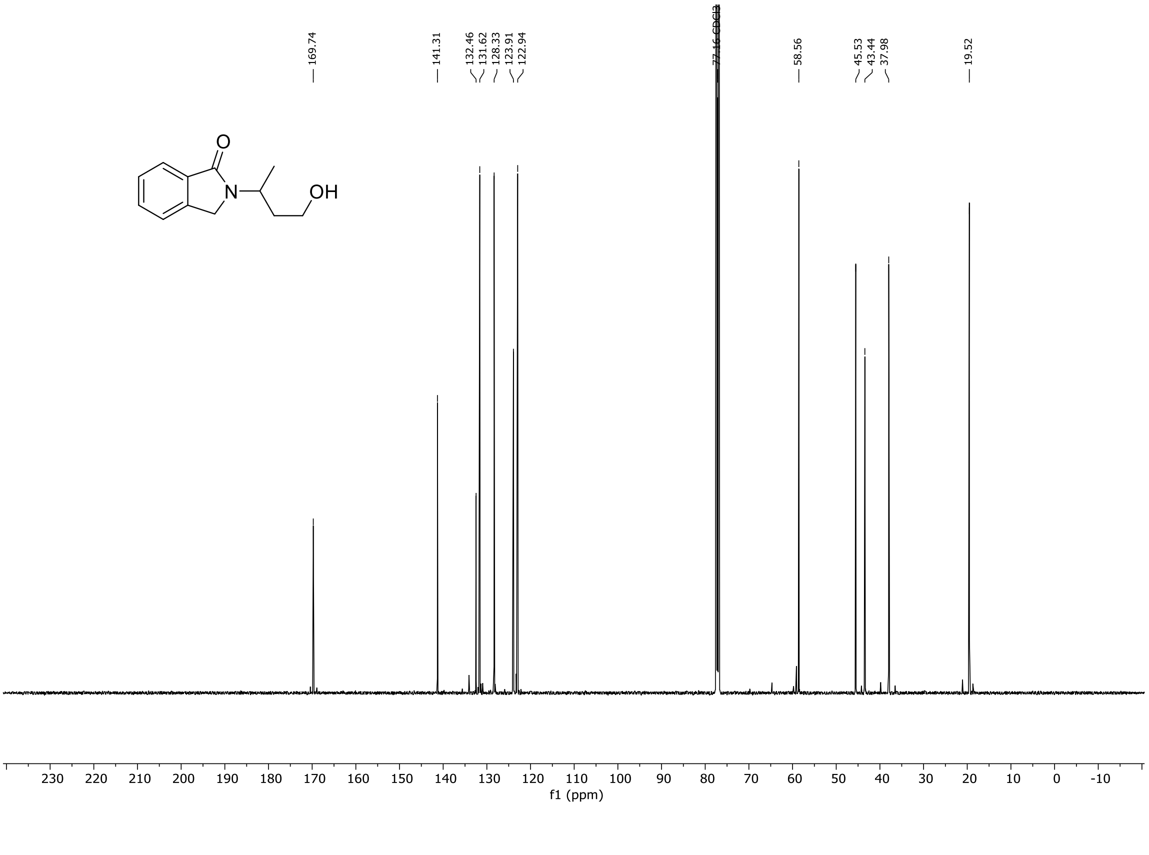

*8.1.7. 2-(4-Hydroxy-3-methylbutyl)isoindolin-1-one (**SI-1x**)*

^{1}H NMR (500 MHz, CDCl$_3$):

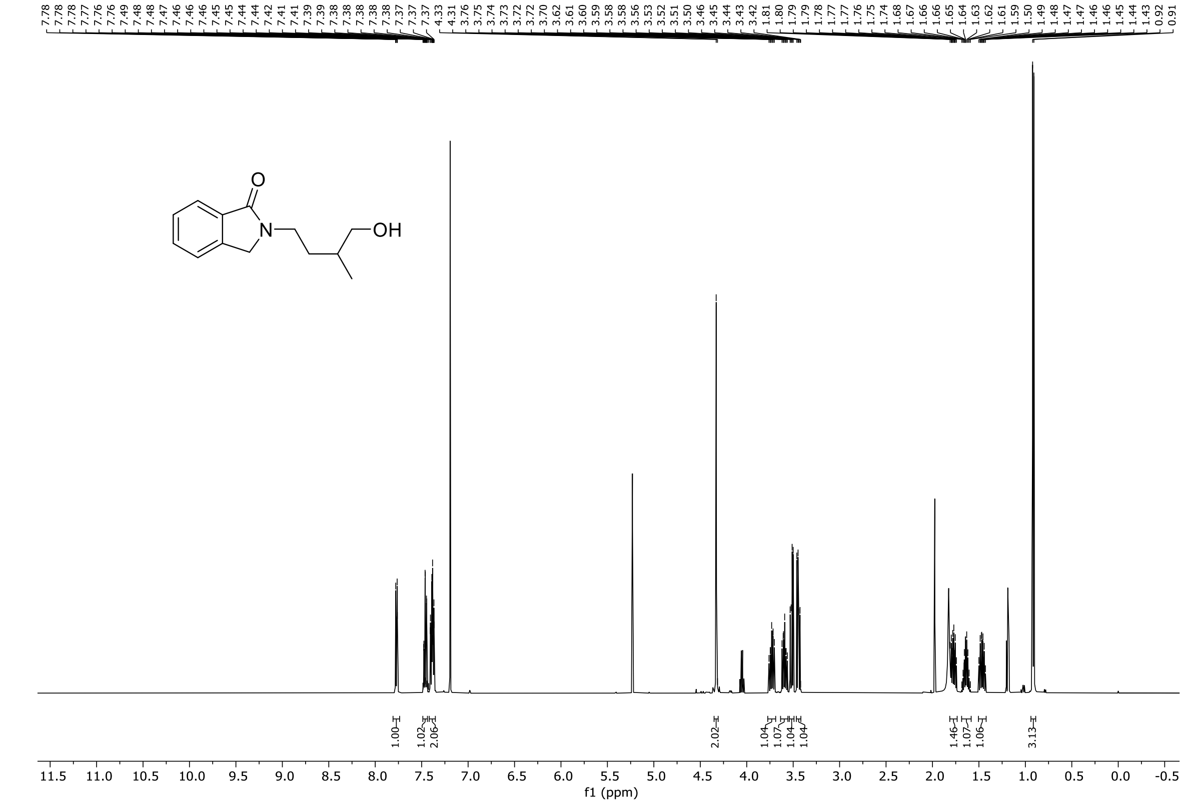

^{13}C NMR (126 MHz, CDCl$_3$):

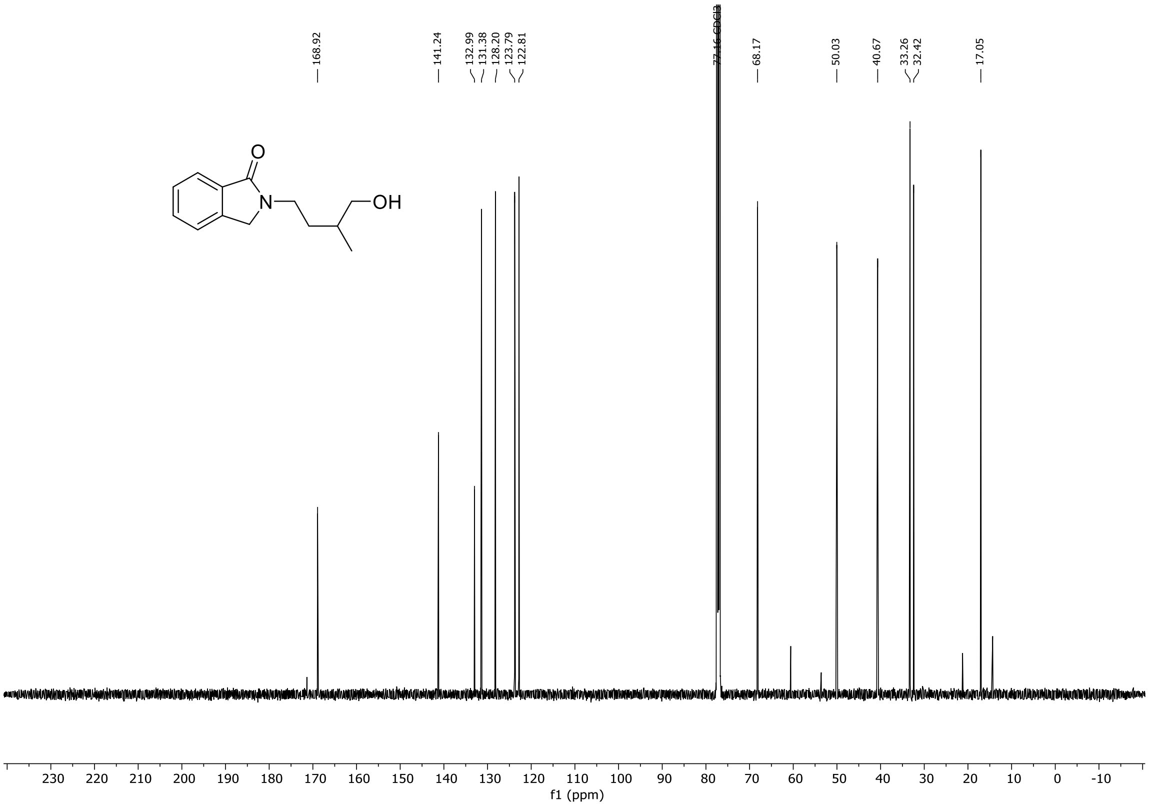

*8.1.8. 2-(2-(1,3-dioxolan-2-yl)ethyl)-7-bromoisoindolin-1-one (**SI-2c**)*

^{1}H NMR (500 MHz, $CDCl_3$):

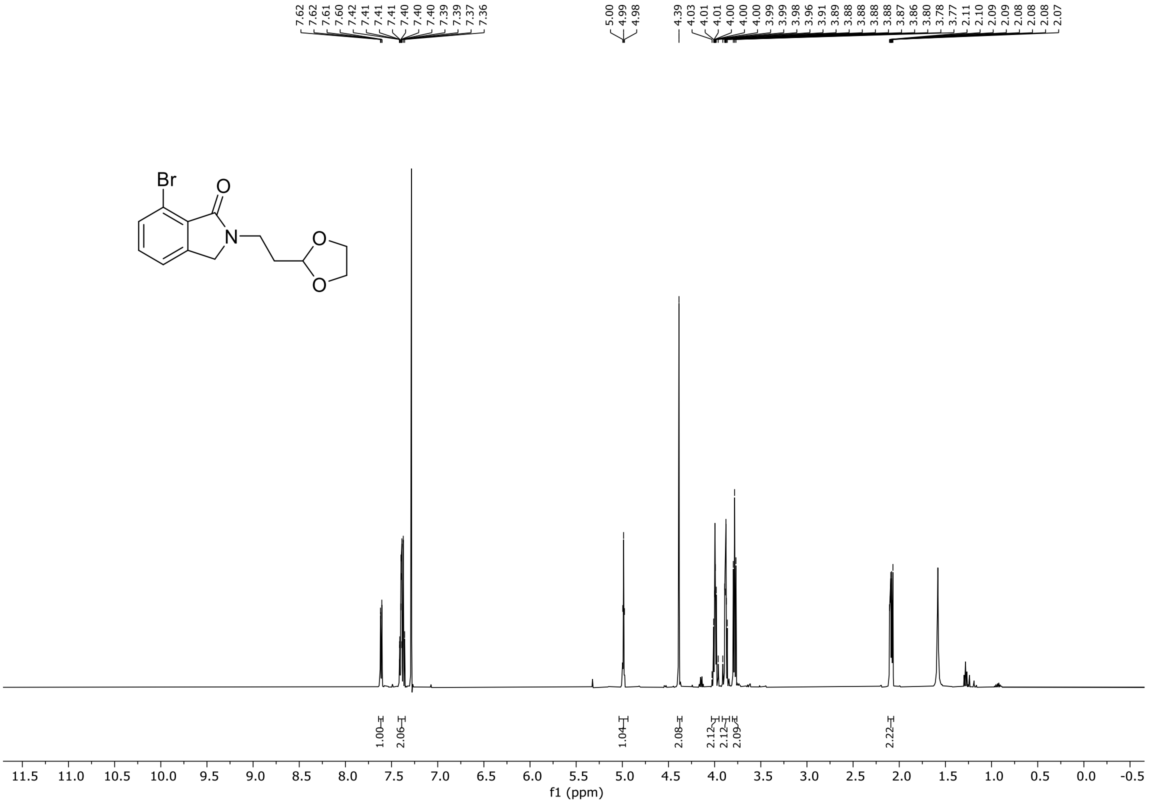

^{13}C NMR (126 MHz, $CDCl_3$):

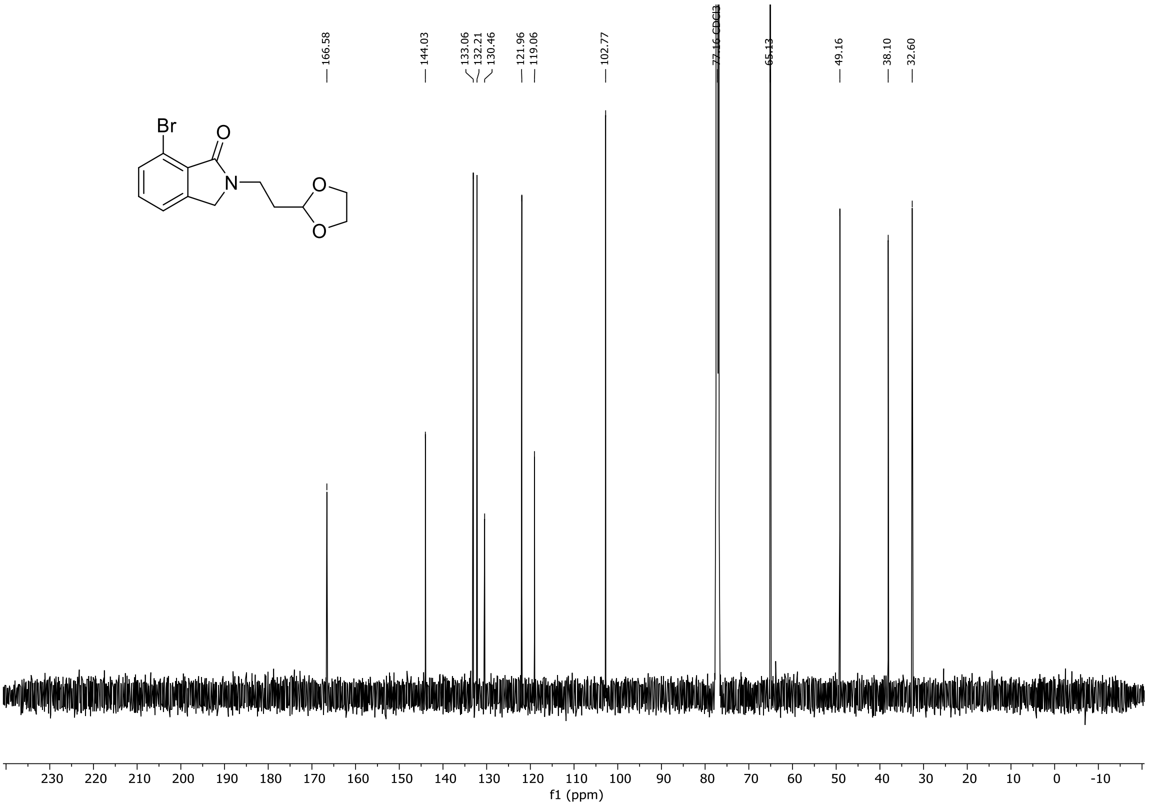

*8.1.9. 2-(2-(1,3-dioxolan-2-yl)ethyl)-7-methylisoindolin-1-one (**SI-2d**)*

^{1}H NMR (500 MHz, $CDCl_3$):

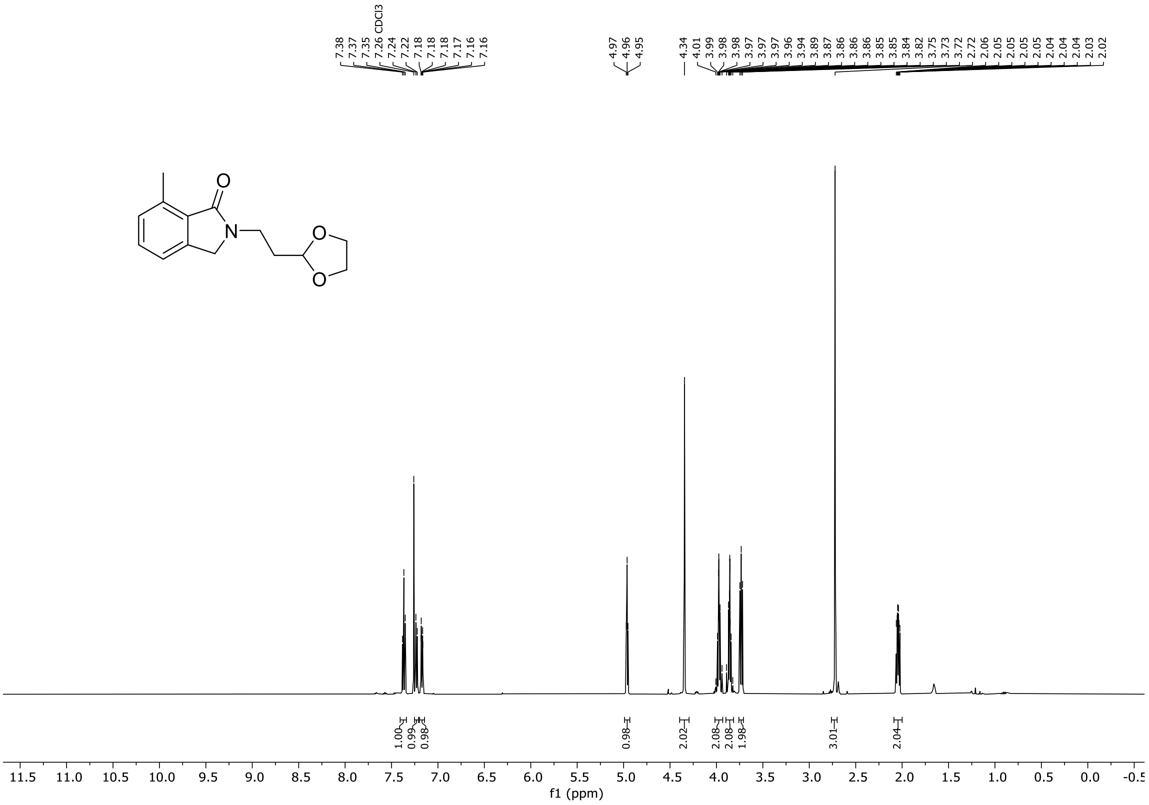

^{13}C NMR (126 MHz, $CDCl_3$):

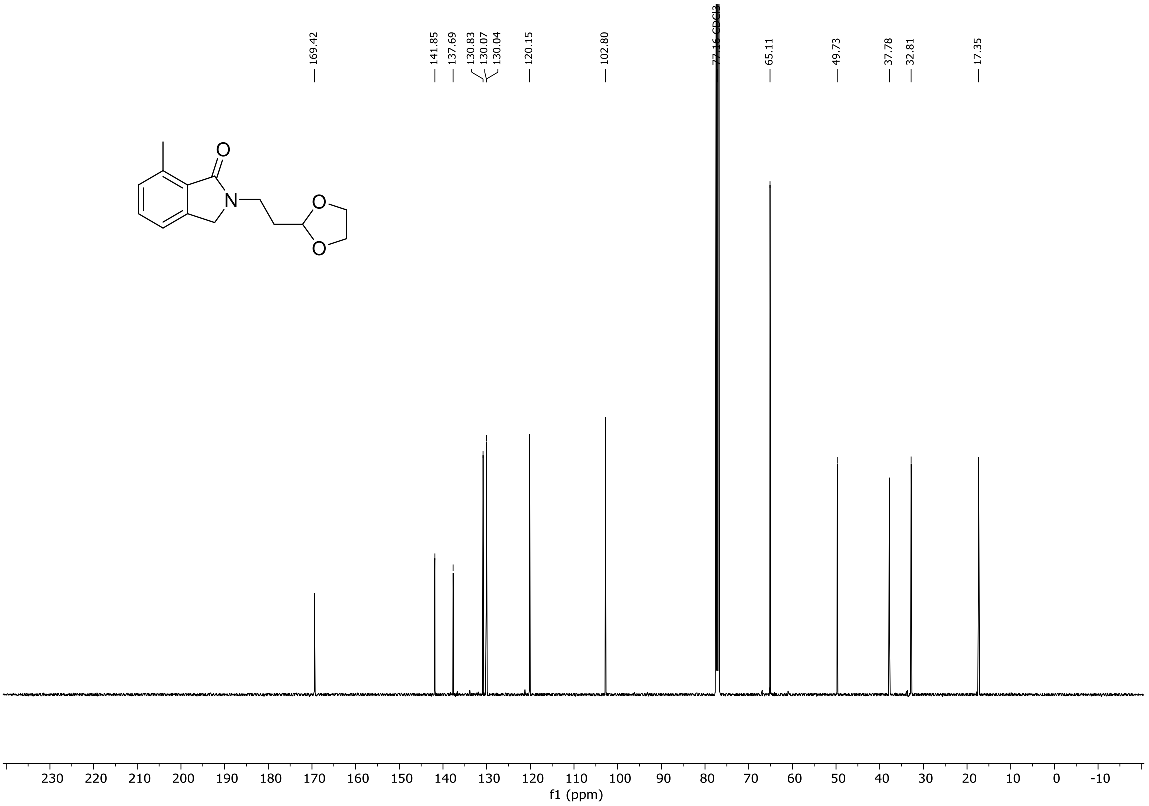

*8.1.10. 2-(2-(1,3-dioxolan-2-yl)ethyl)-5-fluoroisoindolin-1-one (**SI-2g**)*

^{1}H NMR (500 MHz, $CDCl_3$):

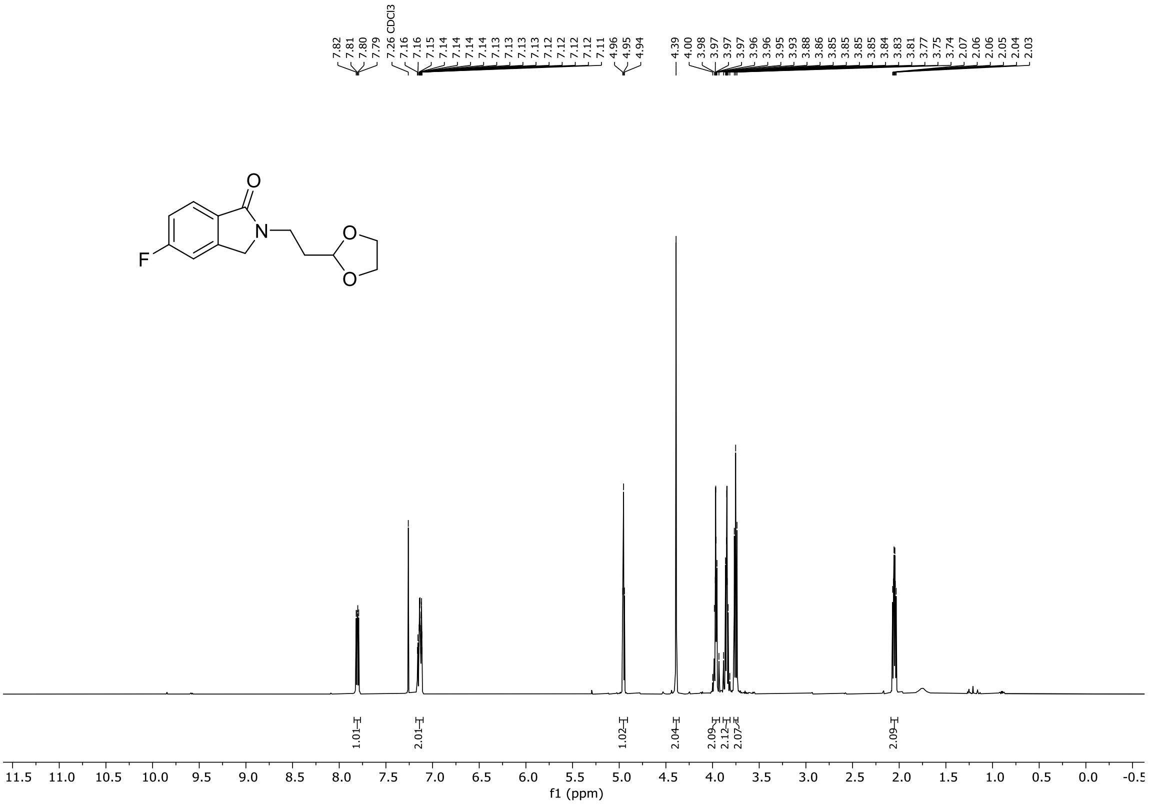

^{13}C NMR (126 MHz, $CDCl_3$):

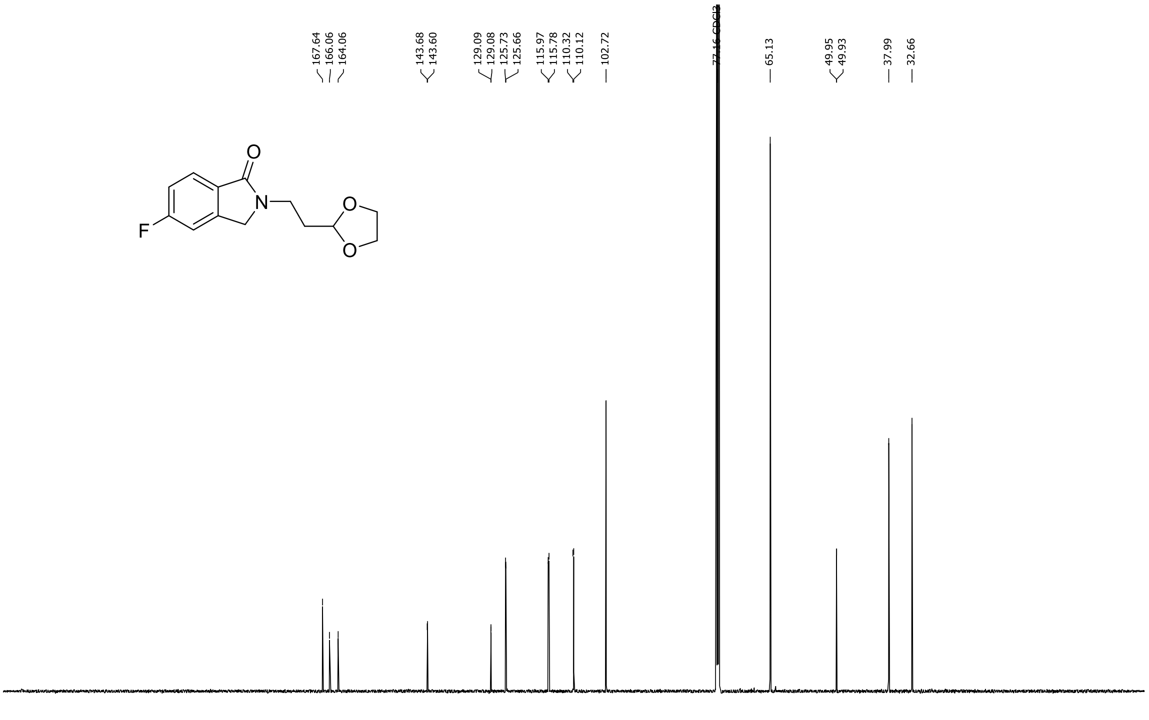

^{19}F NMR (376 MHz, $CDCl_3$):

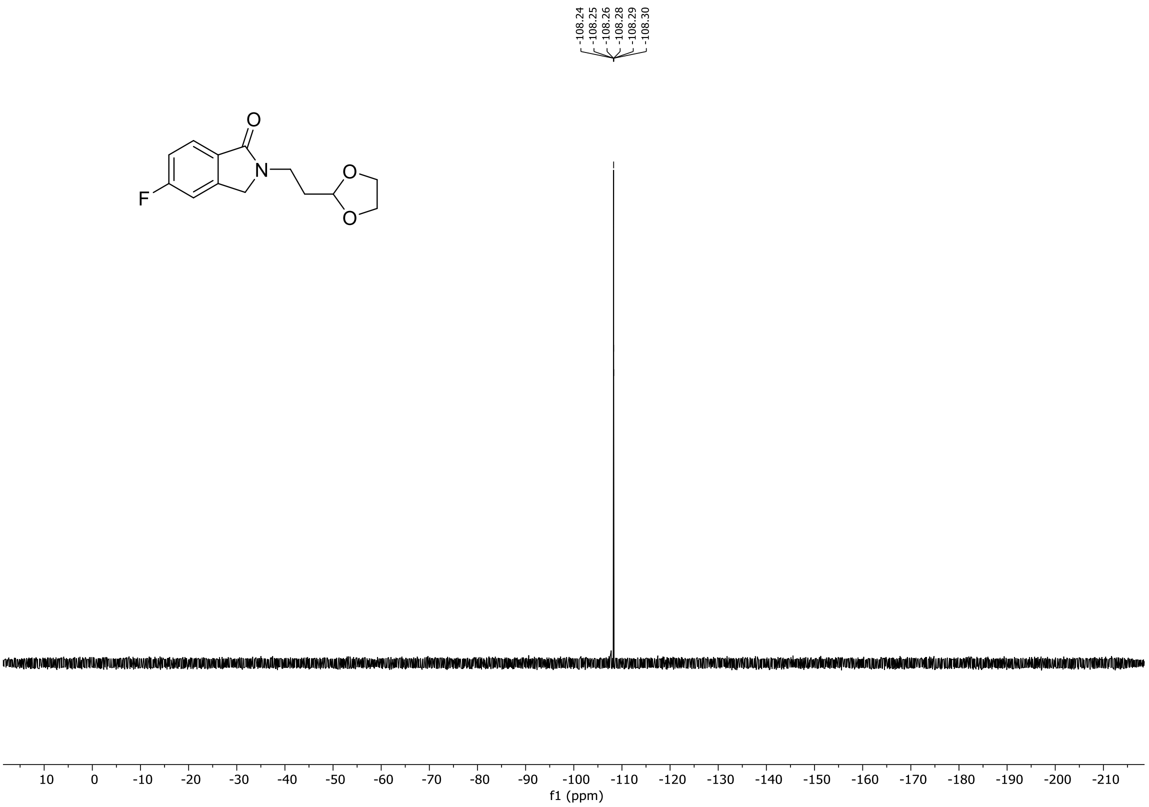

8.1.11. *2-(2-(1,3-dioxolan-2-yl)ethyl)-5-bromoisoindolin-1-one (**SI-2h**)*

^{1}H NMR (500 MHz, $CDCl_3$):

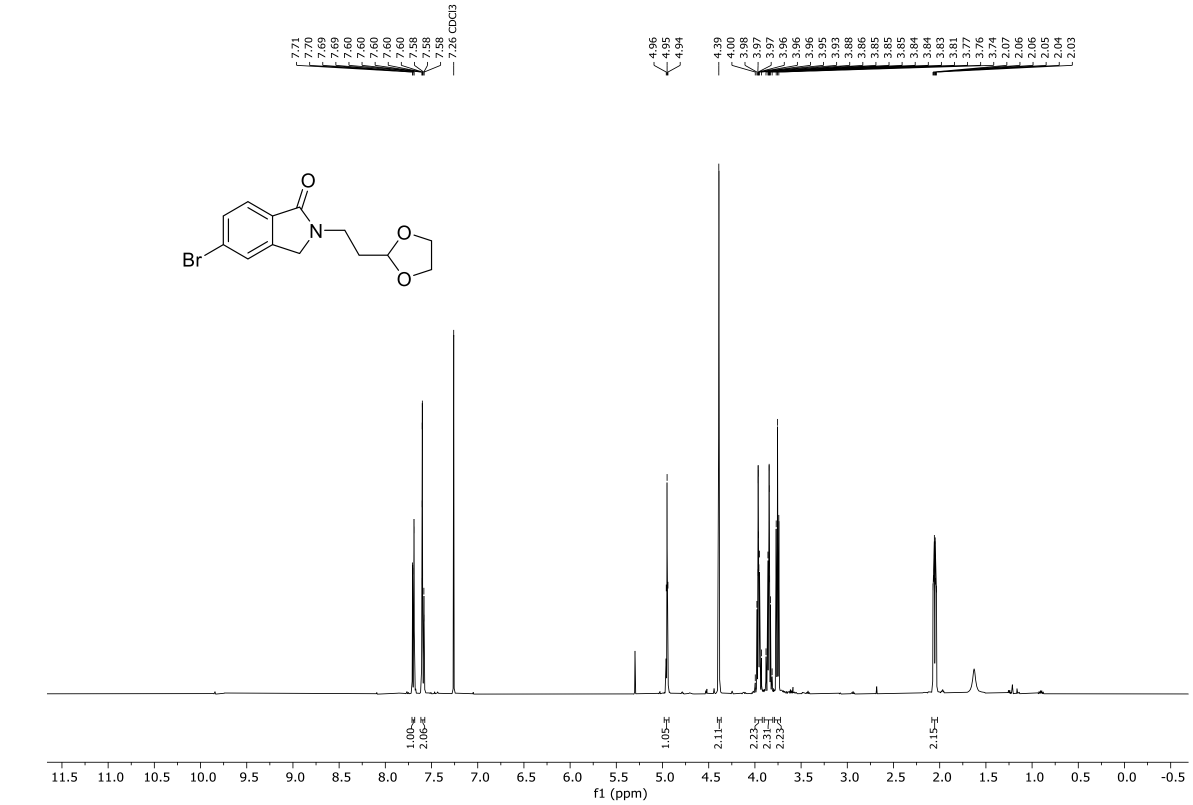

^{13}C NMR (126 MHz, $CDCl_3$):

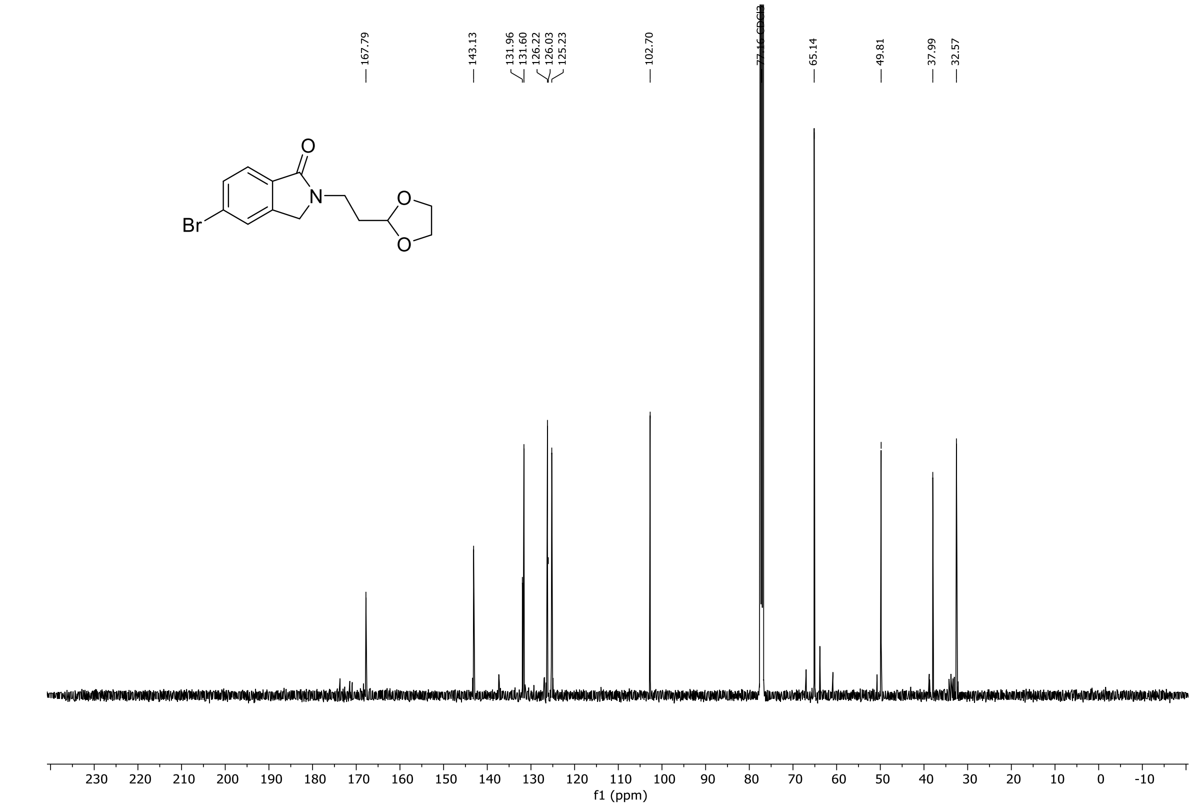

*8.1.12. 2-(2-(1,3-dioxolan-2-yl)ethyl)-1-oxoisoindoline-5-carbonitrile (**SI-2j**)*

^{1}H NMR (500 MHz, $CDCl_3$):

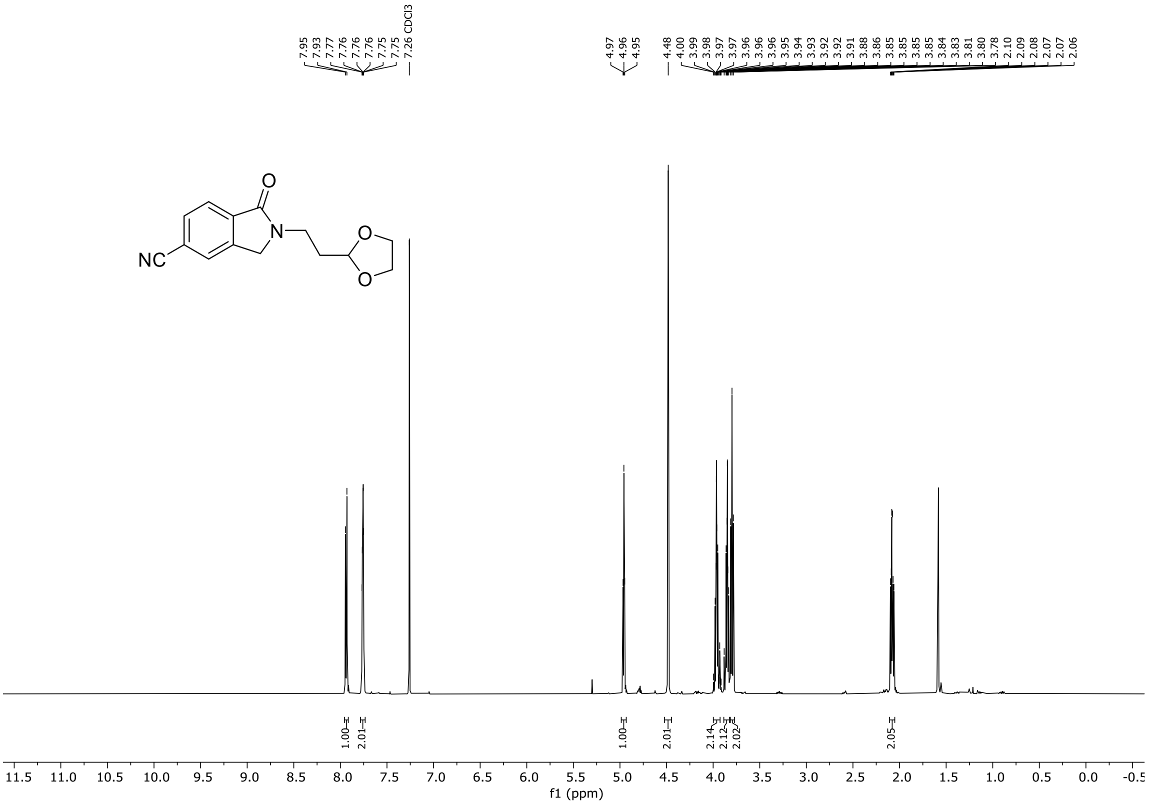

^{13}C NMR (126 MHz, $CDCl_3$):

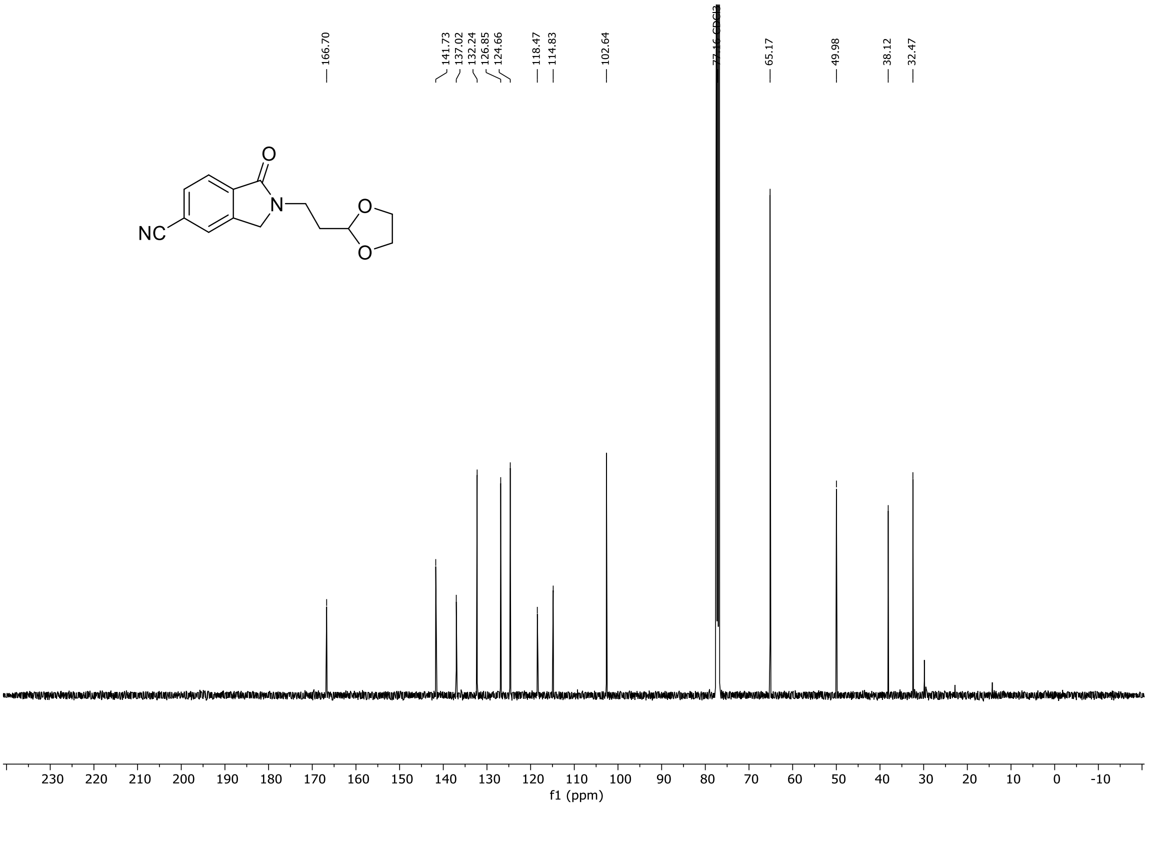

*8.1.13. 2-(2-(1,3-Dioxolan-2-yl)ethyl)-4-bromoisoindolin-1-one (**SI-2m**)*

^{1}H NMR (500 MHz, $CDCl_3$):

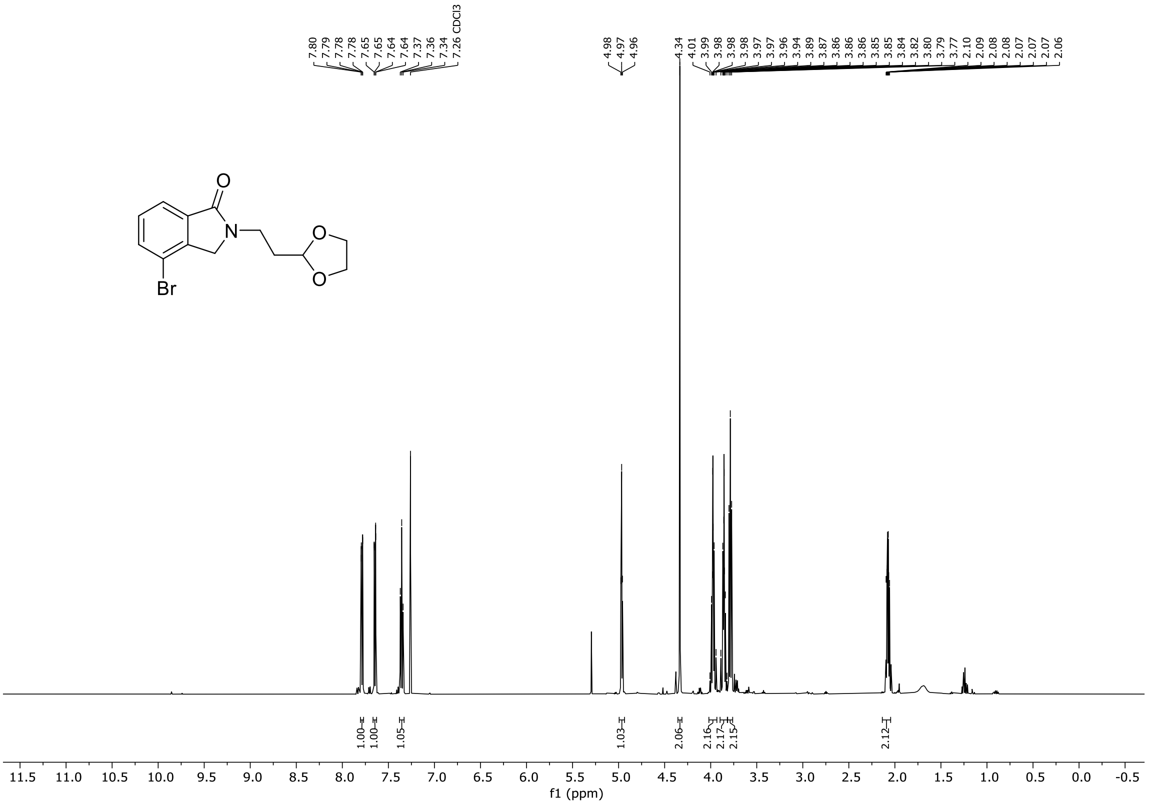

^{13}C NMR (126 MHz, $CDCl_3$):

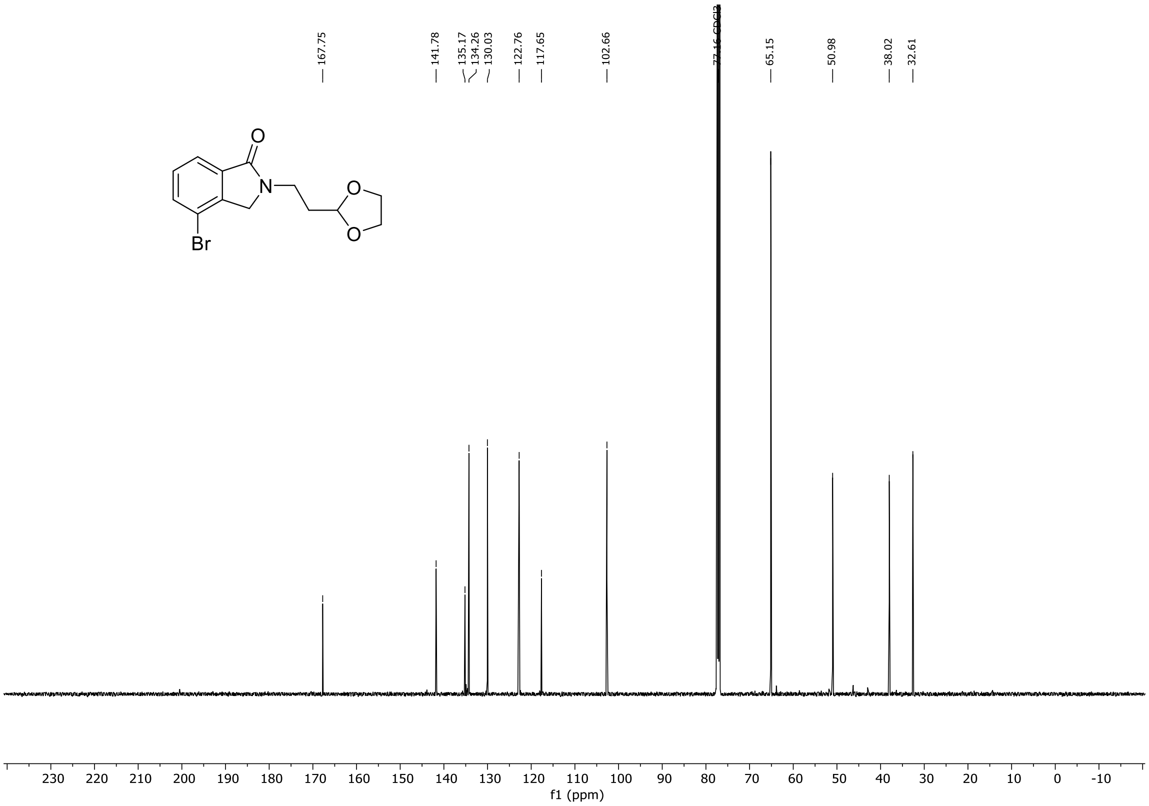

*8.1.14. 2-(3,3-Diethoxypropyl) isoindolin-1-one (**SI-3a**)*

^{1}H NMR (500 MHz, $CDCl_3$):

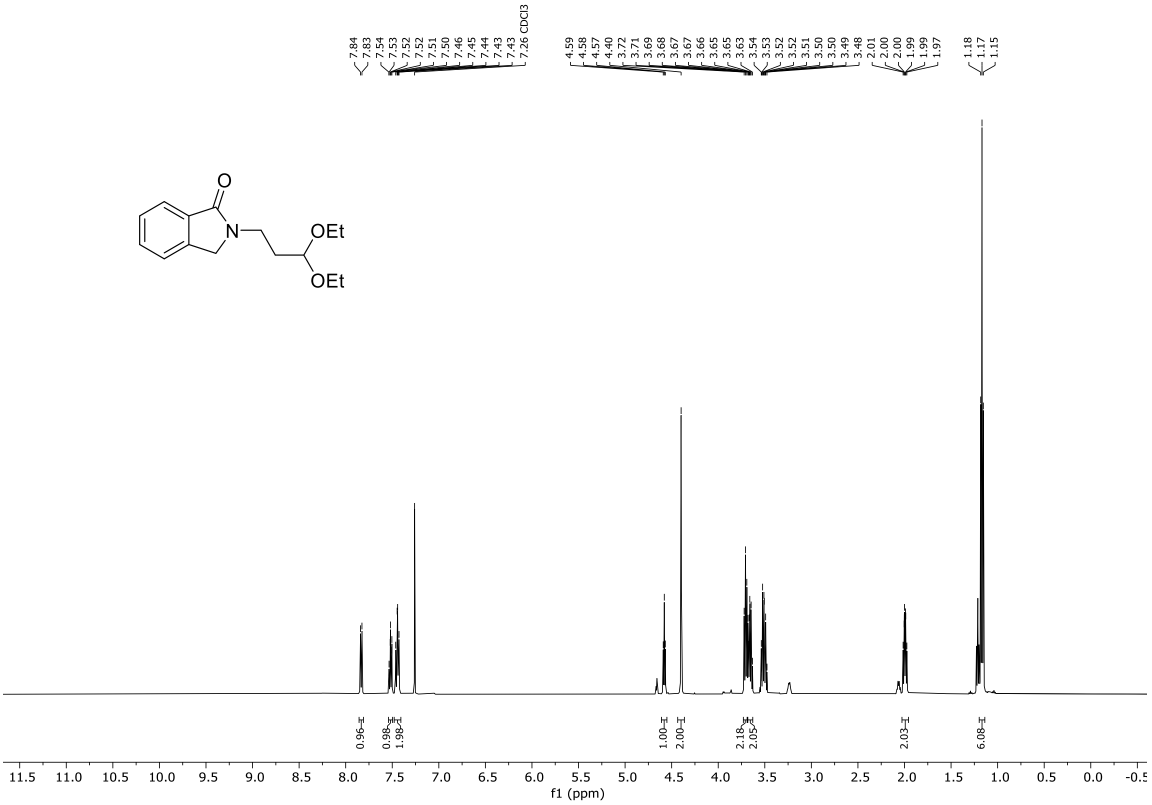

^{13}C NMR (126 MHz, $CDCl_3$):

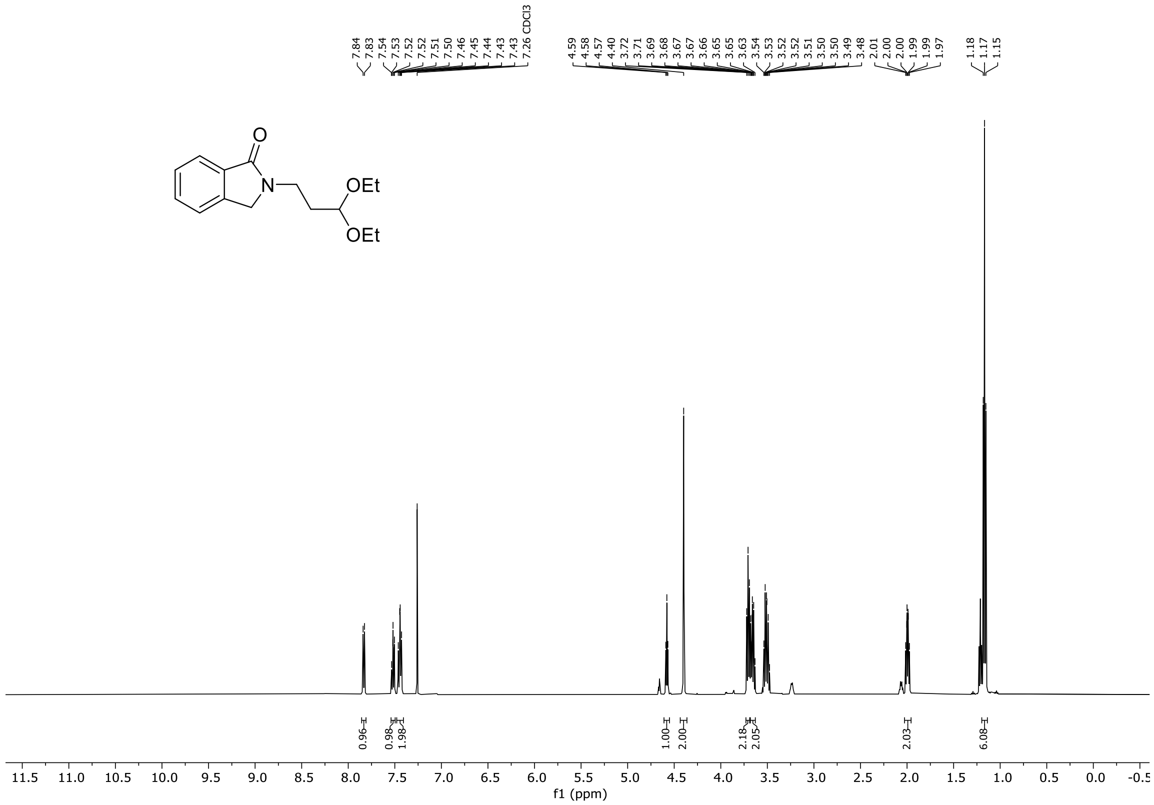

*8.1.15. 2-(3,3-Diethoxypropyl)-5-nitroisoindolin-1-one (**SI-3i**)*

^{1}H NMR (500 MHz, $CDCl_3$):

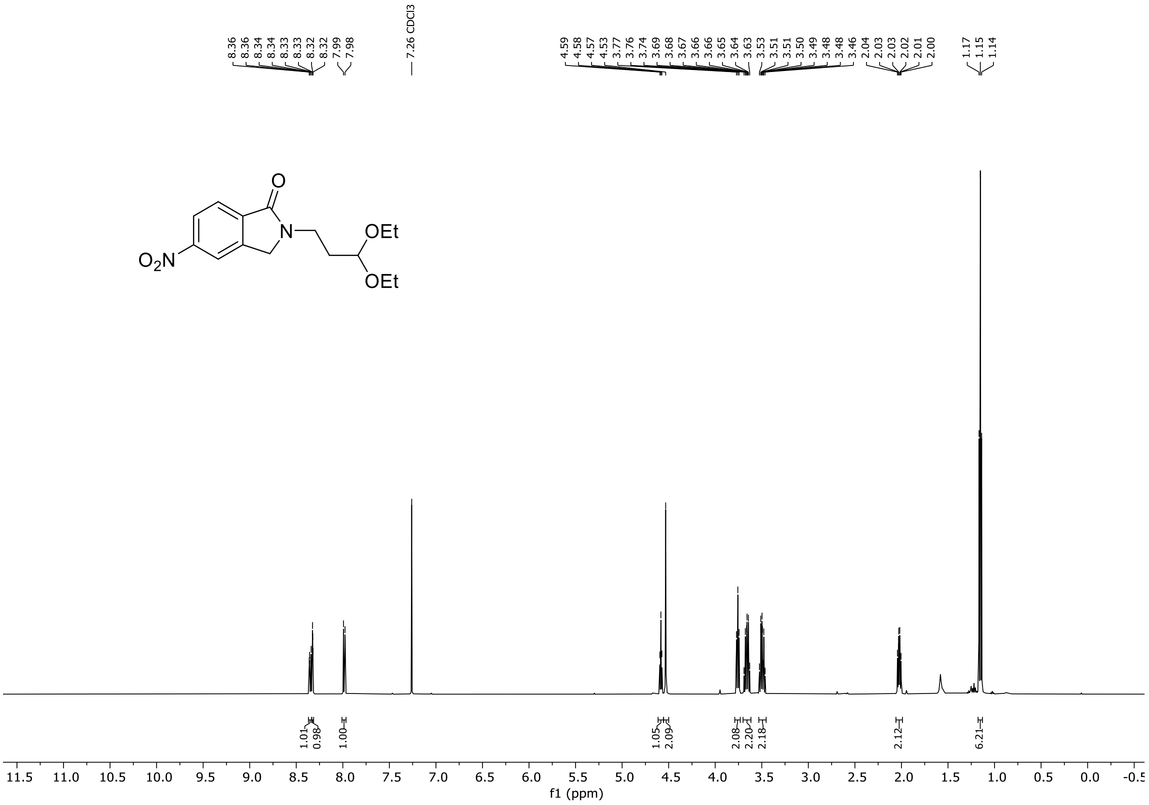

^{13}C NMR (126 MHz, $CDCl_3$):

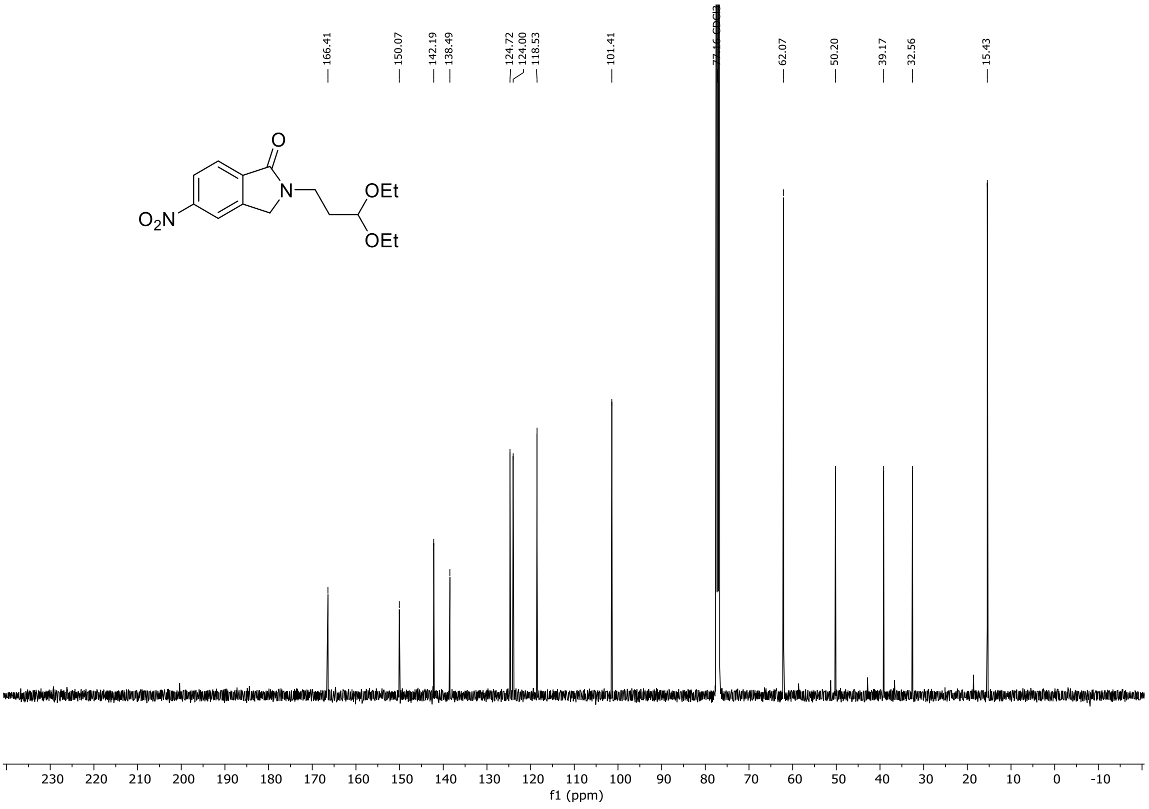

*8.1.16. Methyl 2-(3,3-diethoxypropyl)-1-oxoisoindoline-5-carboxylate (**SI-3k**)*

^{1}H NMR (500 MHz, DMSO-d_6):

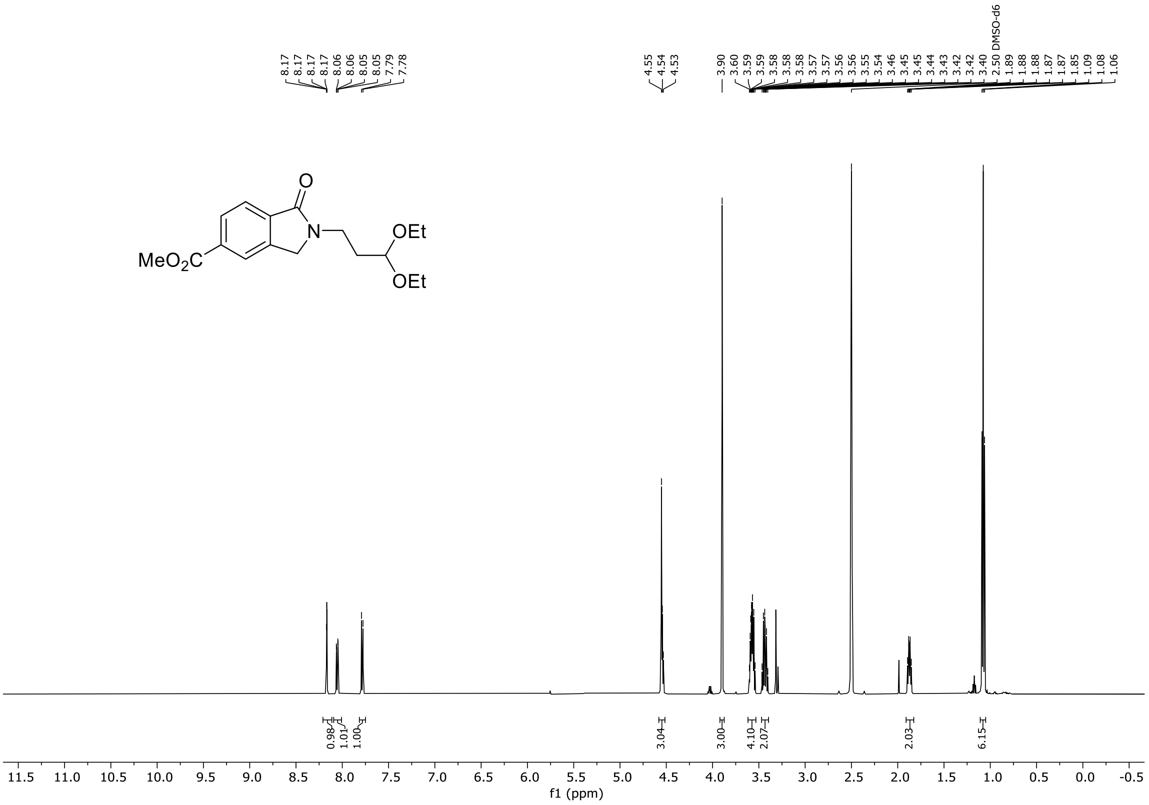

^{13}C NMR (126 MHz, DMSO-d_6):

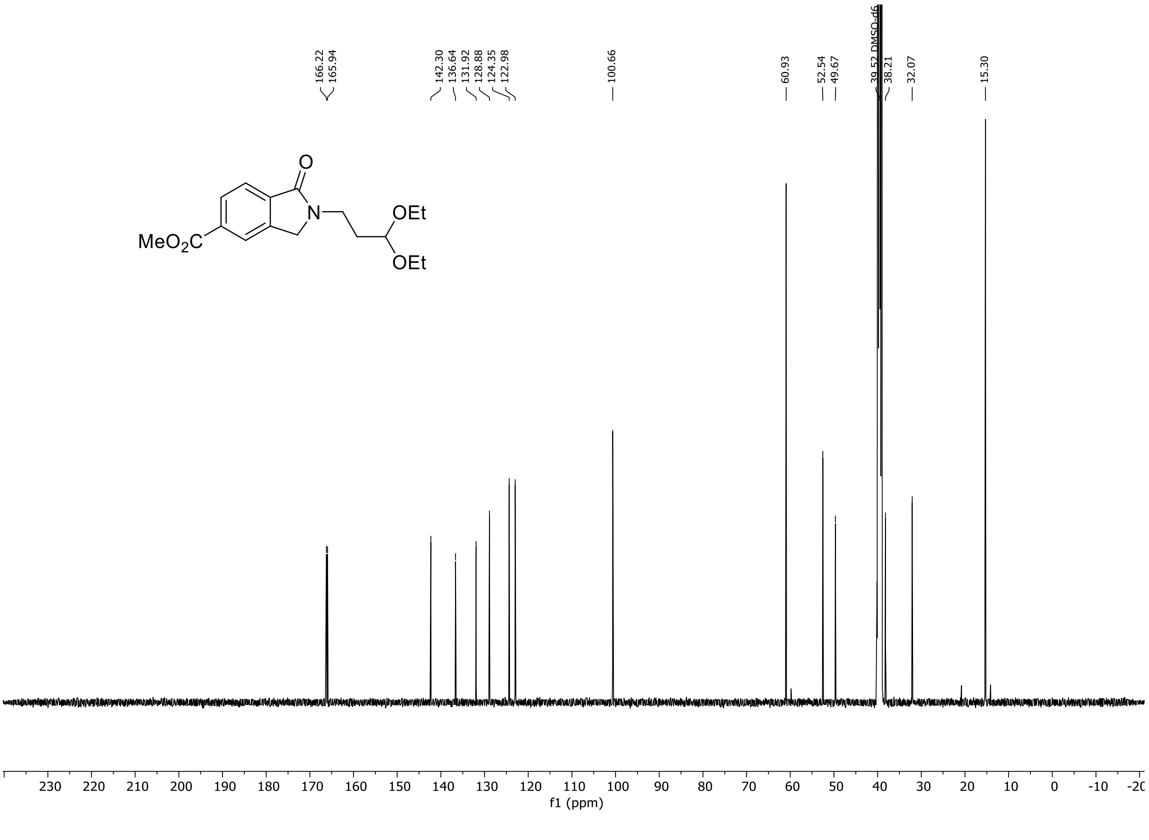

8.1.17. 2-(3,3-Diethoxypropyl)-4-(trifluoromethyl)isoindolin-1-one (**SI-3n**)

^{1}H NMR (500 MHz, DMSO-d_6):

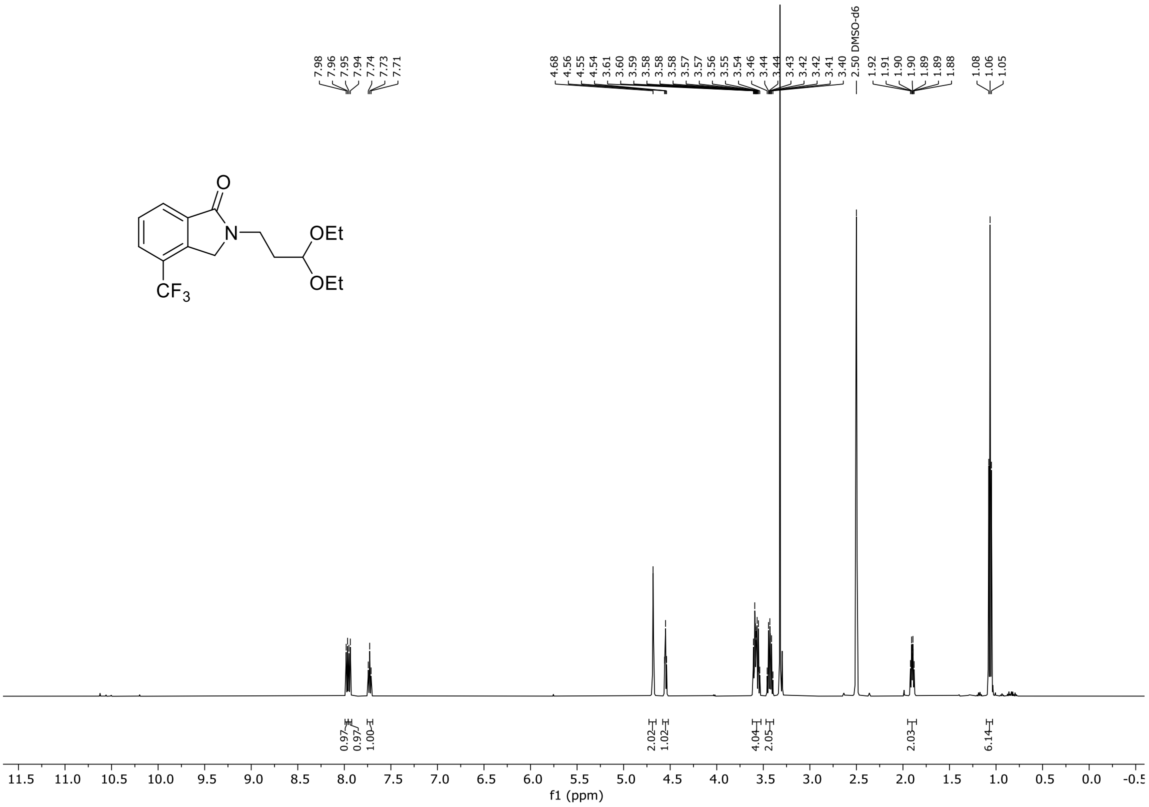

^{13}C NMR (126 MHz, DMSO-d_6):

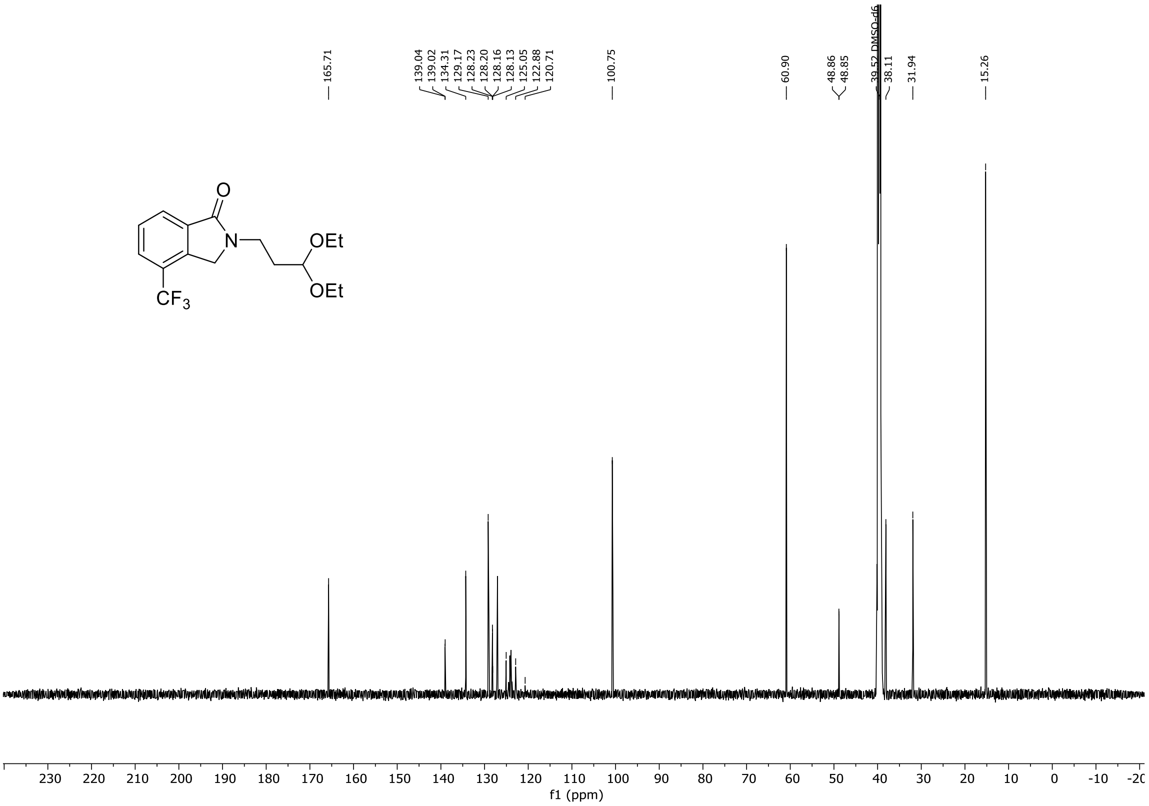

^{19}F NMR (376 MHz, DMSO-d_6):

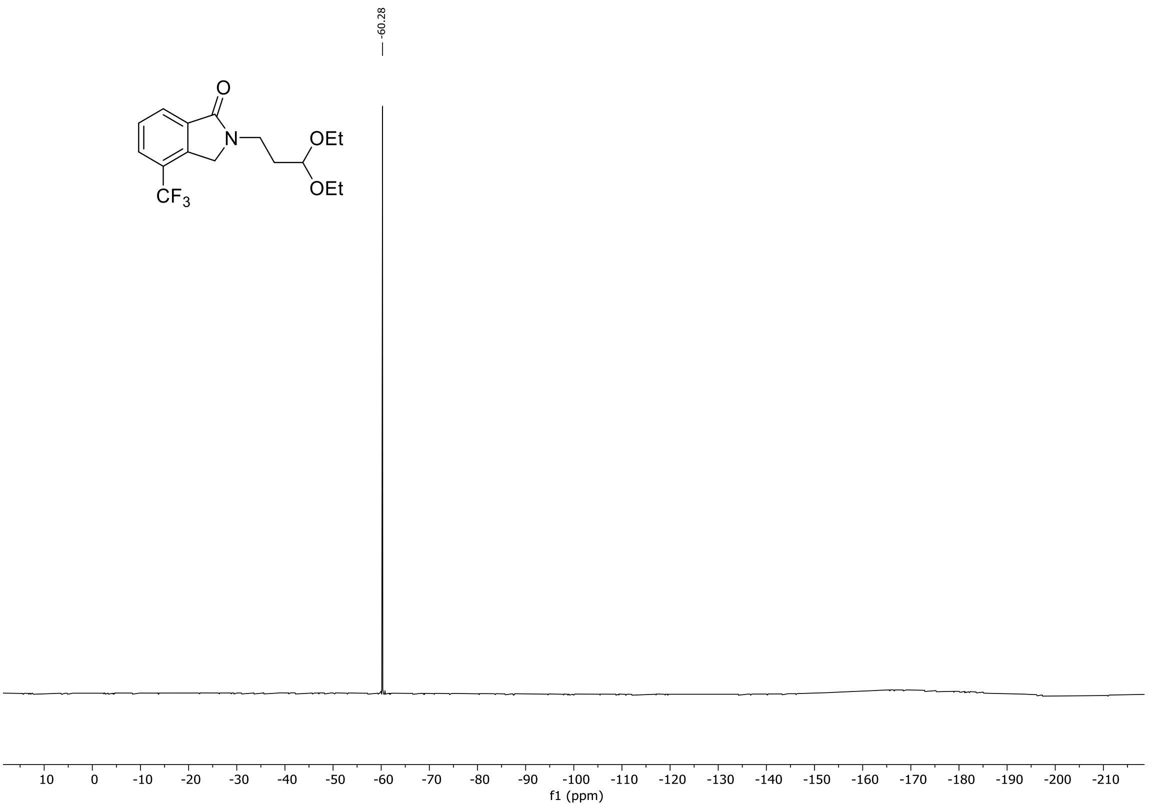

*8.1.18. 6-(3,3-Diethoxypropyl)-6,7-dihydro-5H-pyrrolo[3,4-b]pyridin-5-one (**SI-3p**)*

^{1}H NMR (500 MHz, $CDCl_3$):

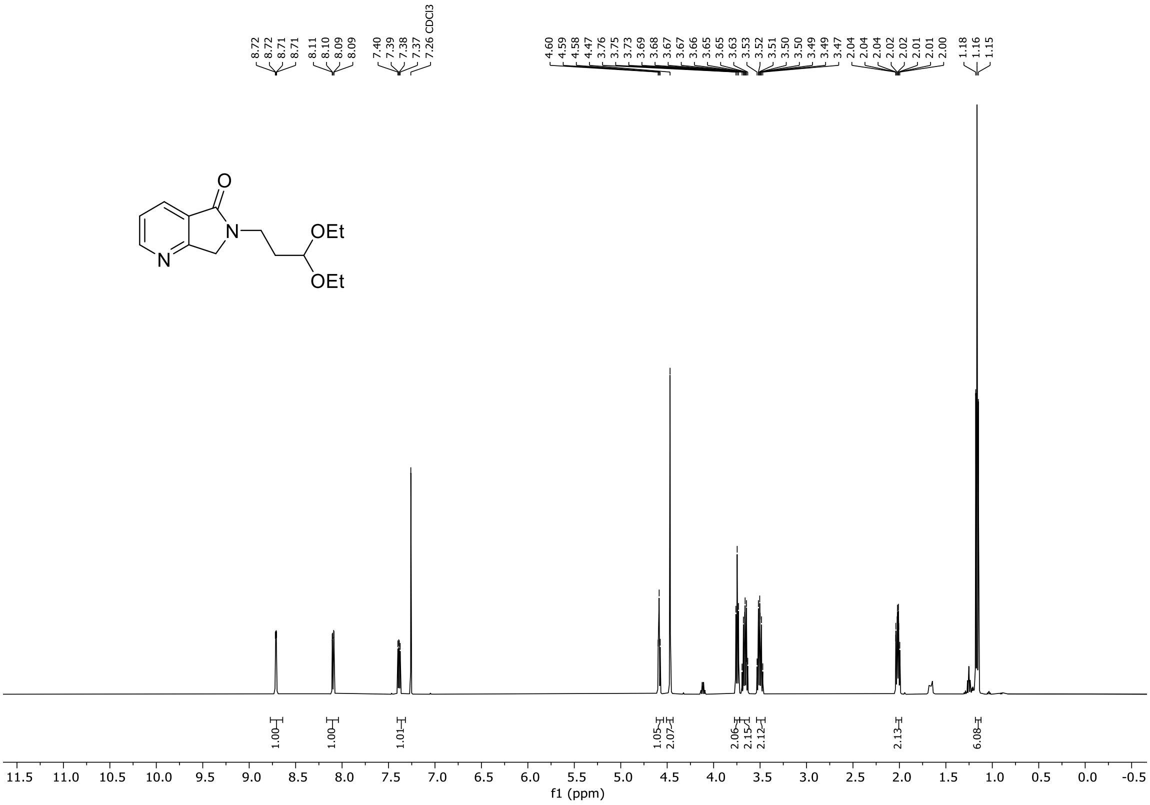

^{13}C NMR (126 MHz, DMSO-d_6):

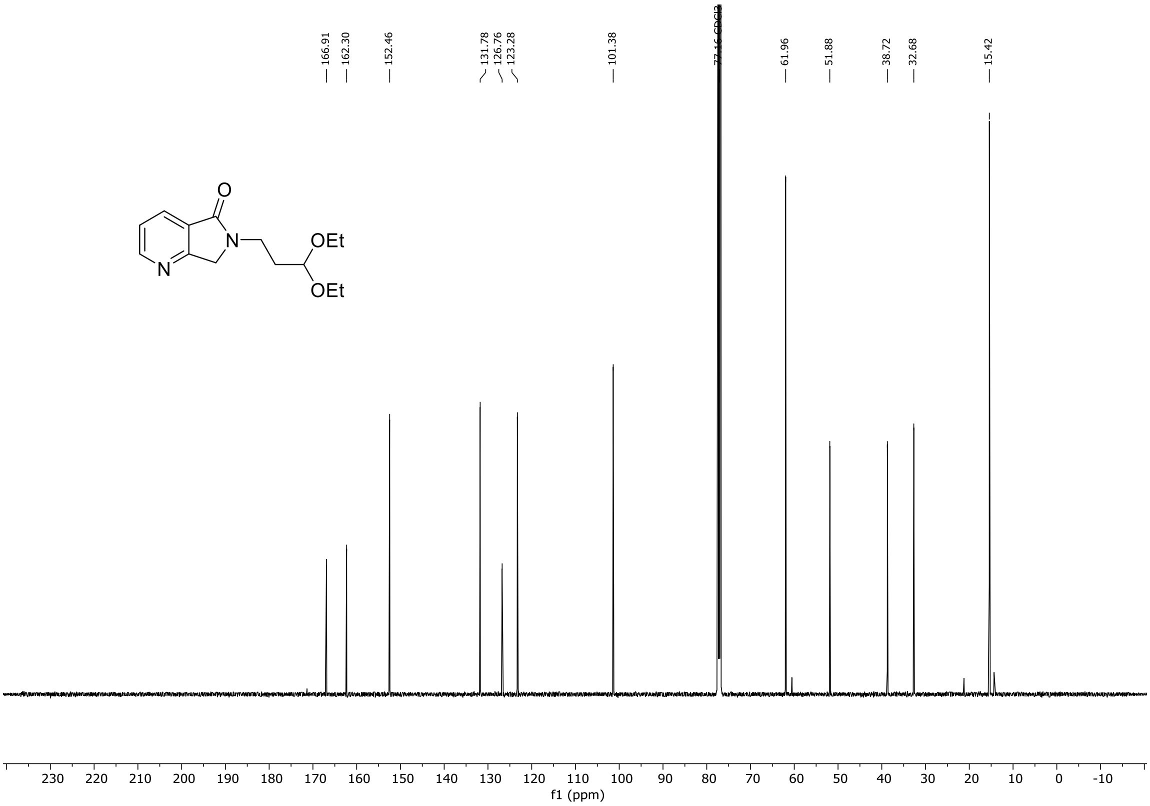

8.2. Hydrazones

*8.2.1. N'-(3-(1-Oxoisoindolin-2-yl)propylidene)-4-methylbenzenesulfonohydrazide (**2a**)*

^{1}H NMR (500 MHz, DMSO-d_6):

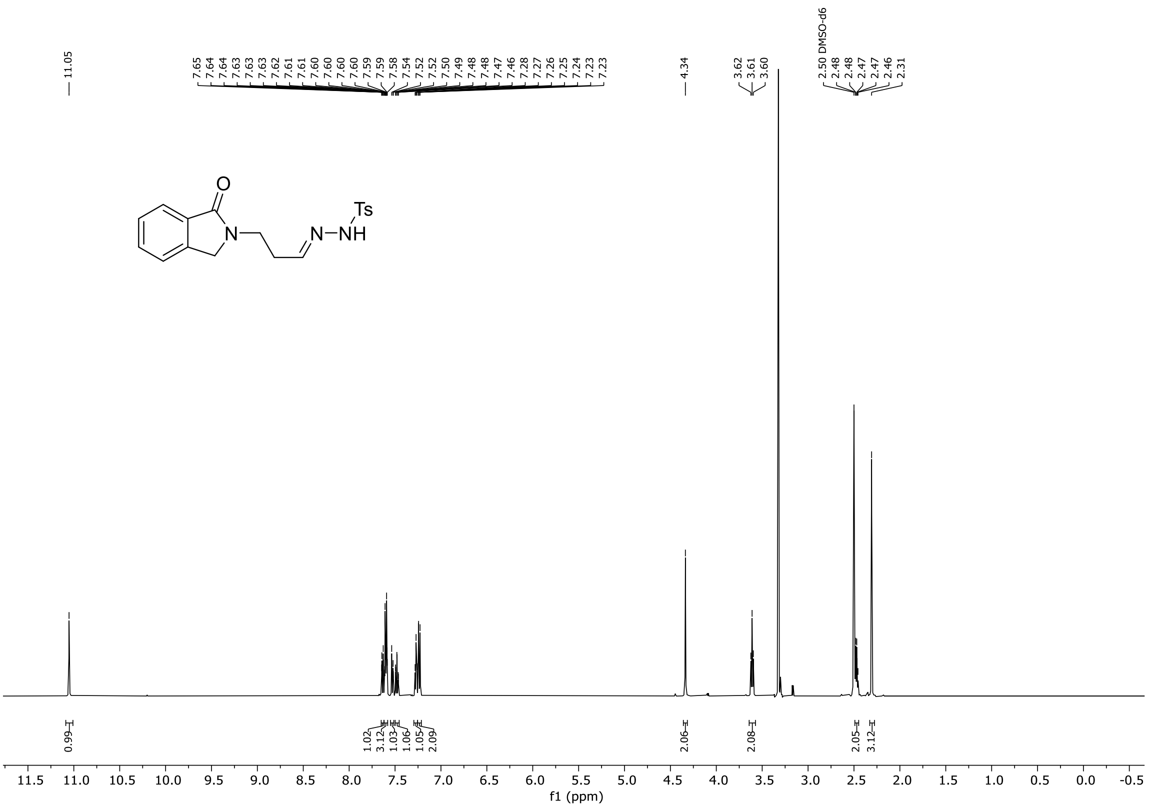

^{13}C NMR (126 MHz, DMSO-d_6):

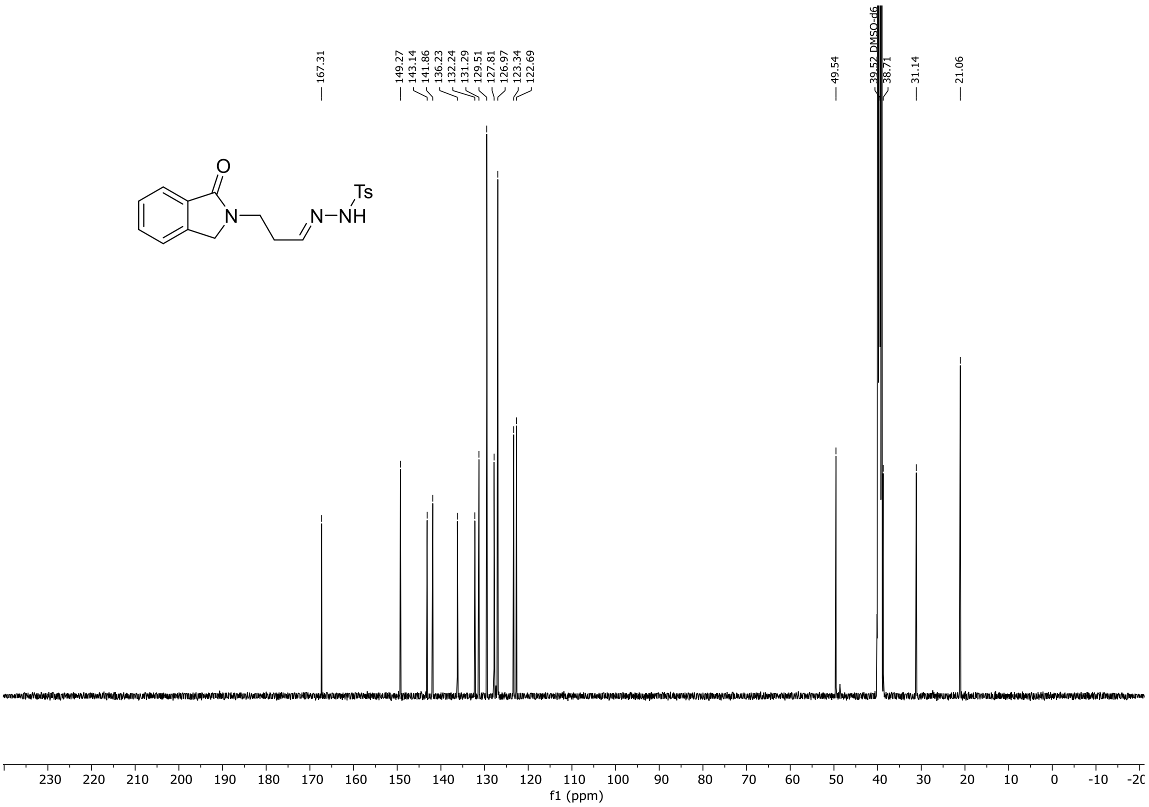

*8.2.2. N'-(2,2-Dimethyl-3-(1-oxoisoindolin-2-yl)propylidene)-4-methylbenzenesulfonohydrazide (**2b**)*

^{1}H NMR (500 MHz, DMSO-d_6):

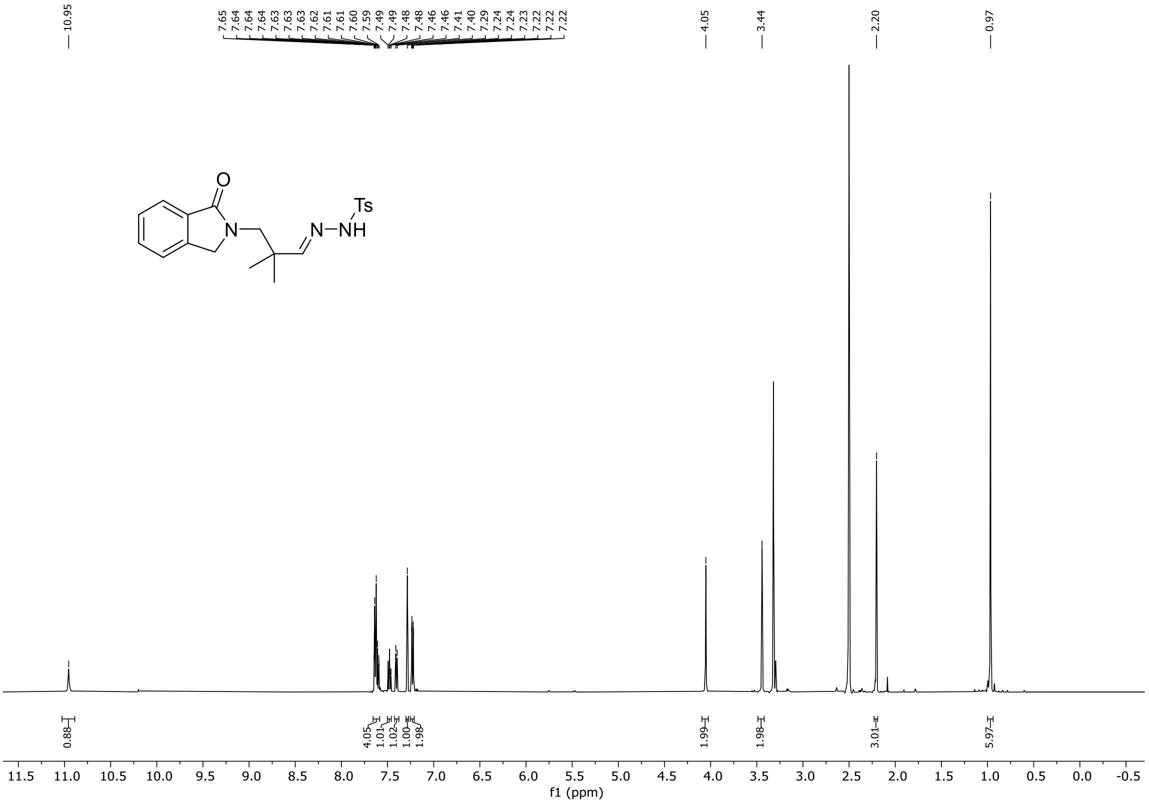

^{13}C NMR (126 MHz, DMSO-d_6):

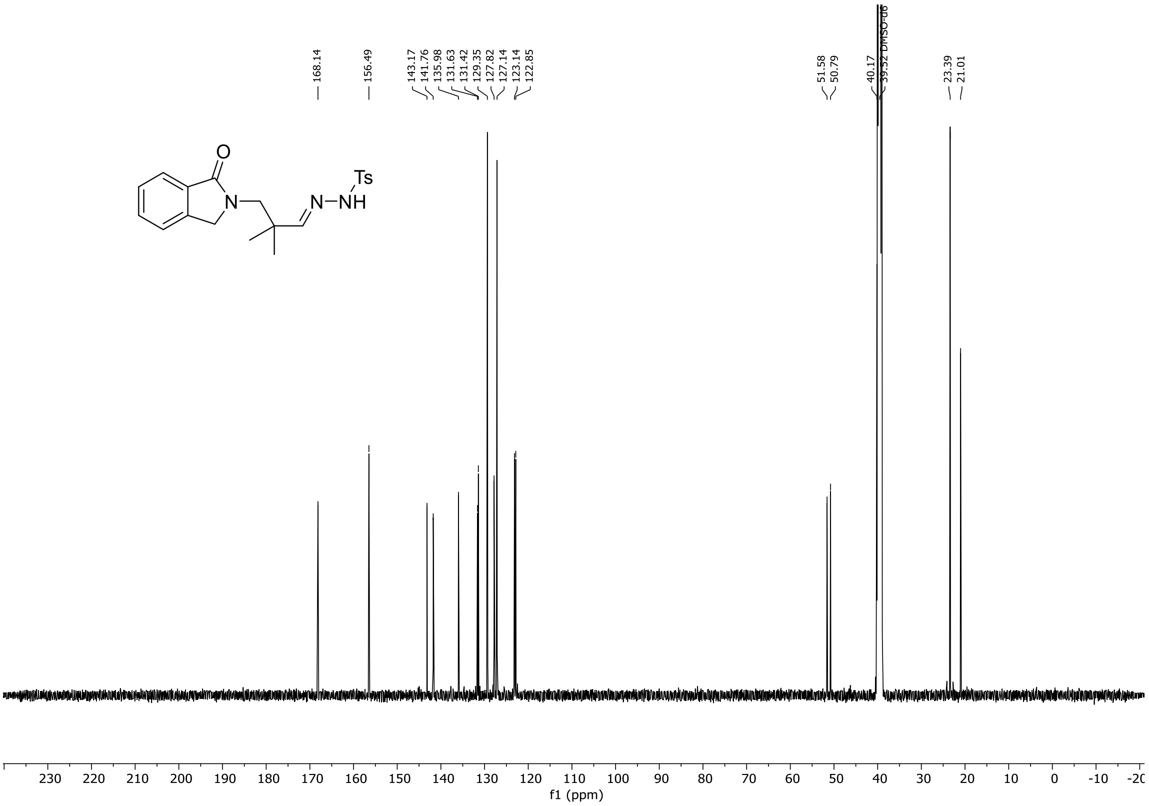

*8.2.3. N'-(3-(7-Bromo-1-oxoisoindolin-2-yl)propylidene)-4-methylbenzenesulfonohydrazide (**2c**)*

^{1}H NMR (500 MHz, DMSO-d_6):

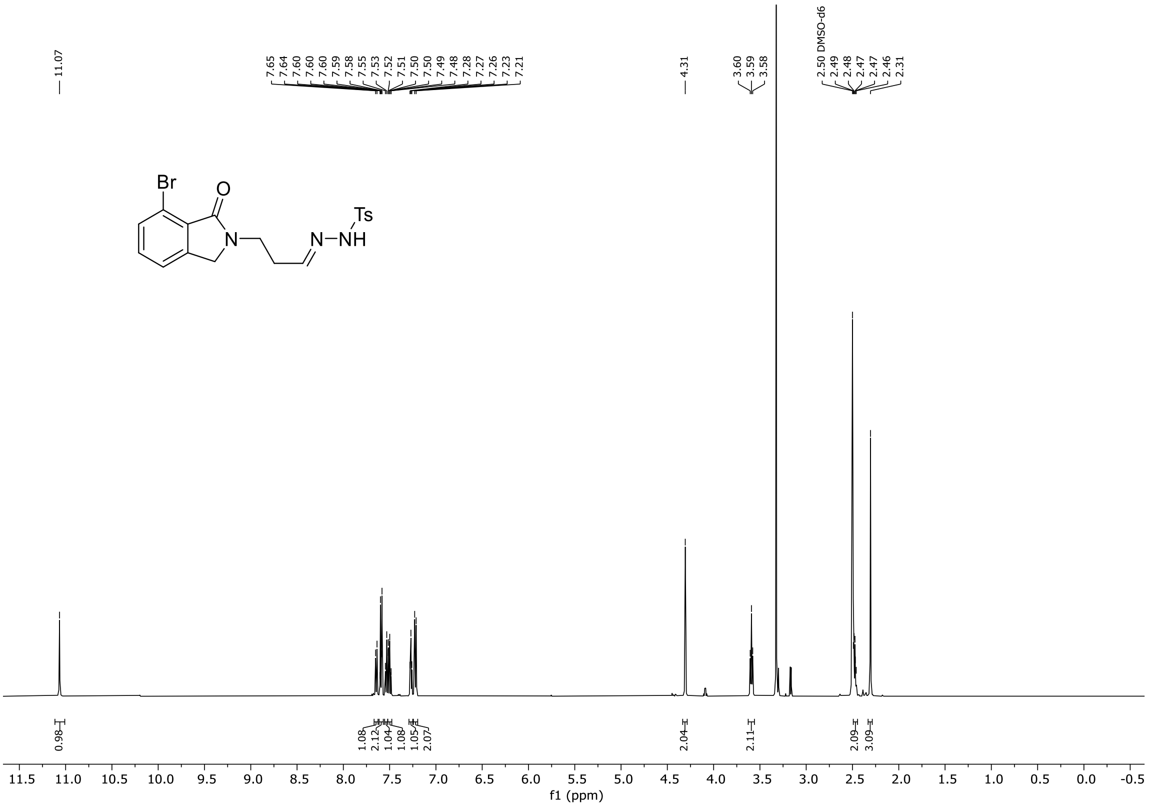

^{13}C NMR (126 MHz, DMSO-d_6):

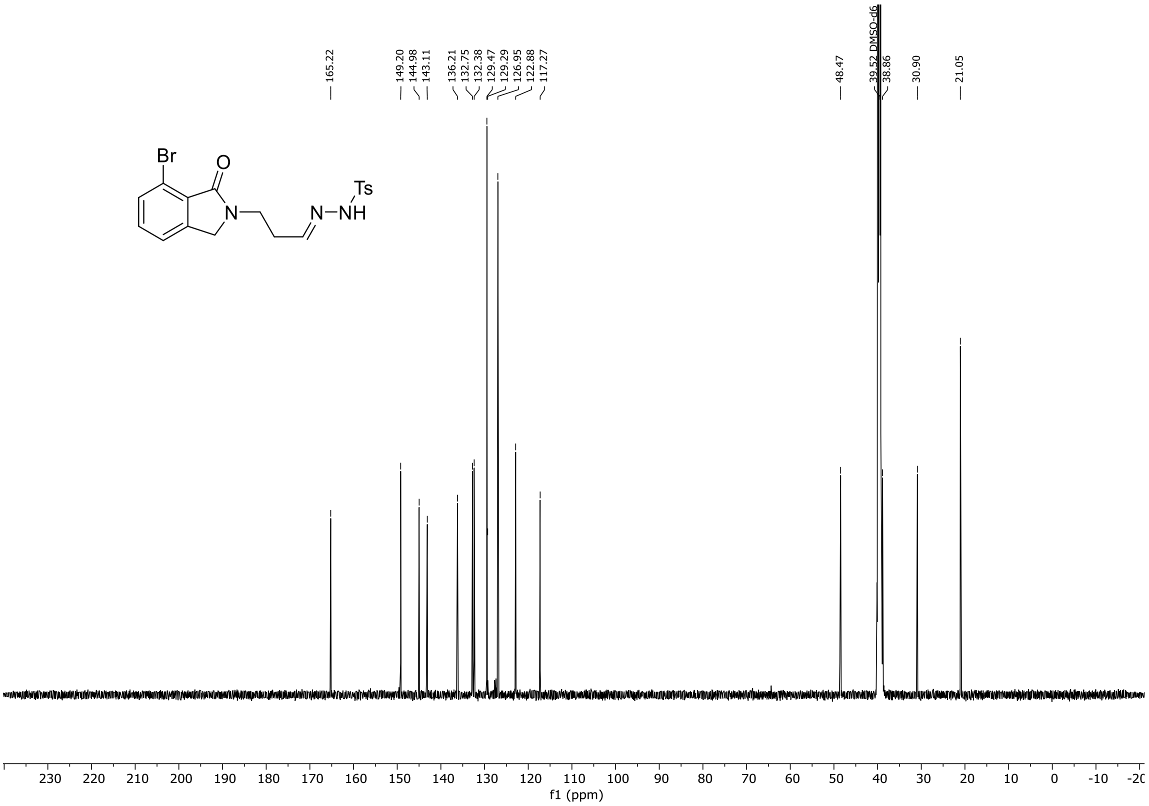

*8.2.4. N'-(3-(7-Methyl-1-oxoisoindolin-2-yl)propylidene)-4-methylbenzenesulfonohydrazide (**2d**)*

^{1}H NMR (500 MHz, DMSO-d_6):

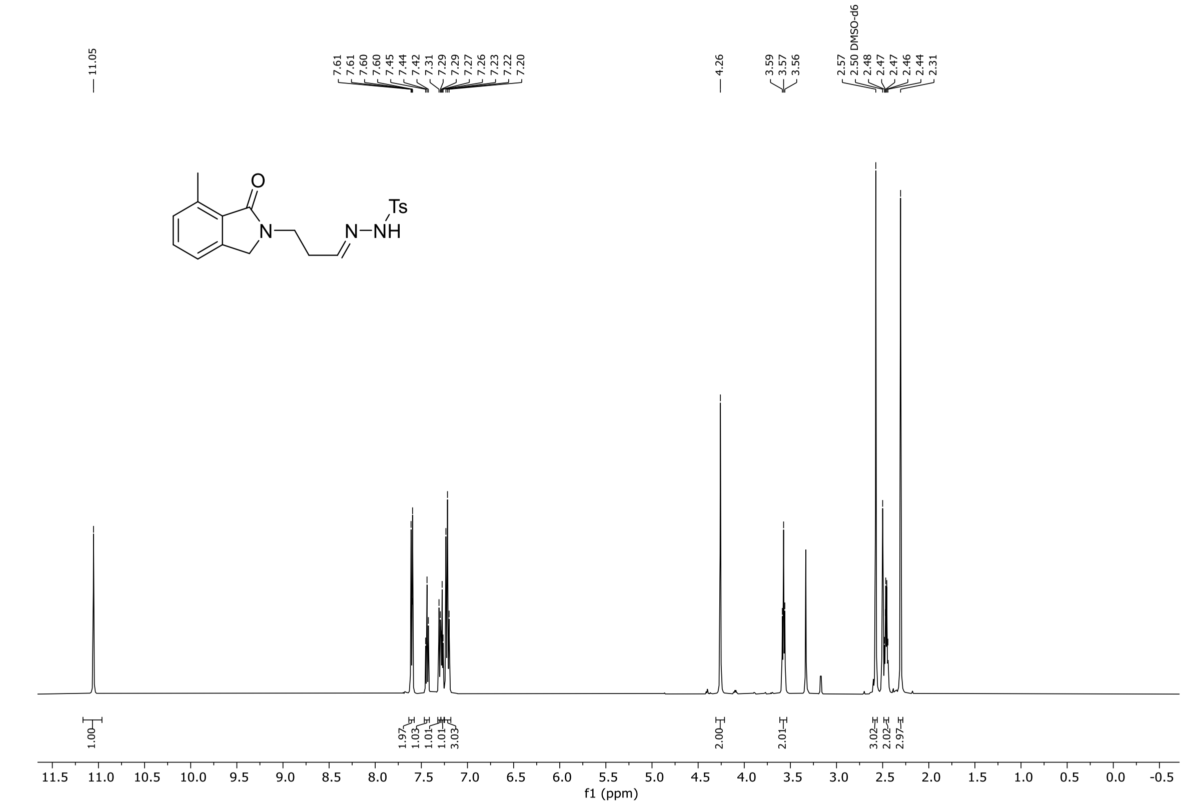

^{13}C NMR (126 MHz, DMSO-d_6):

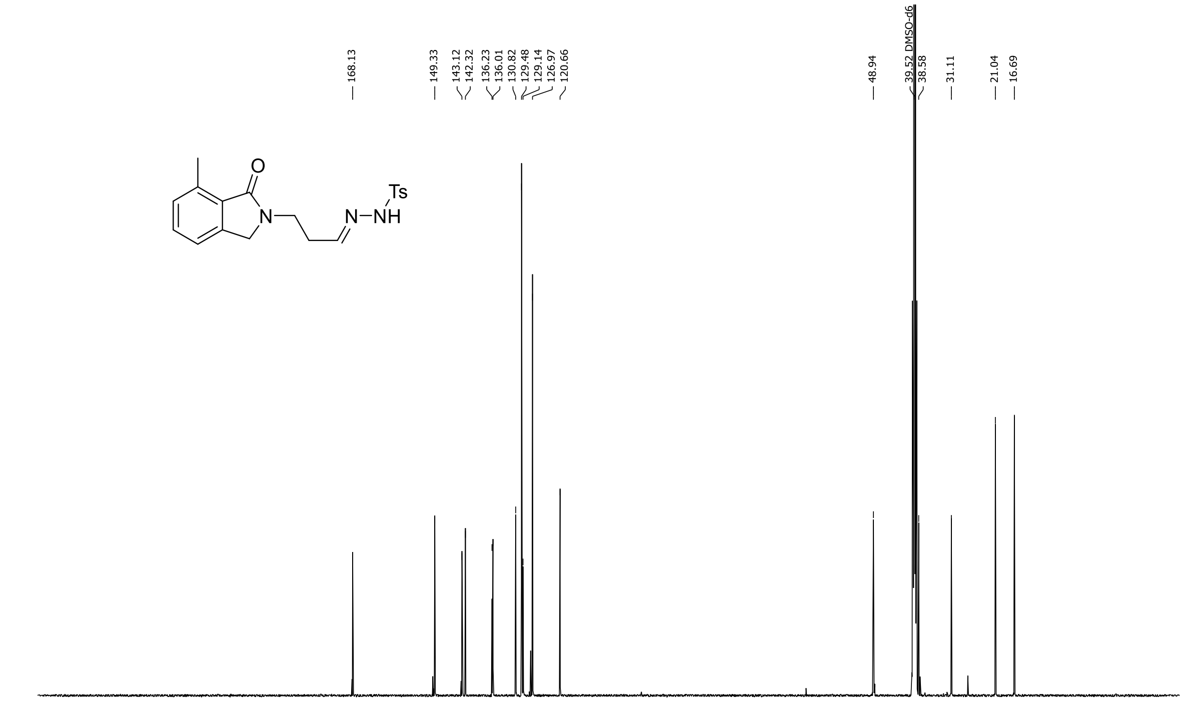

*8.2.5. N'-(3-(6-Chloro-1-oxoisoindolin-2-yl)propylidene)-4-methylbenzenesulfonohydrazide (**2e**)*

^{1}H NMR (500 MHz, DMSO-d_6):

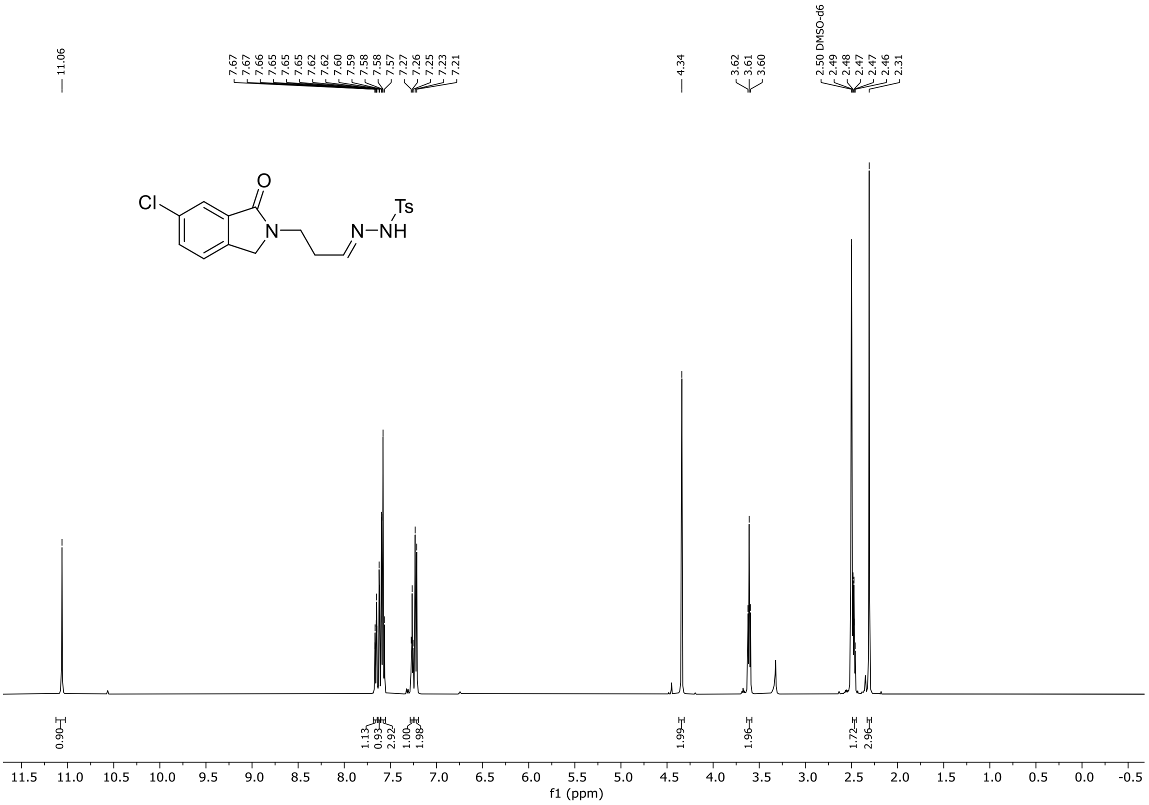

^{13}C NMR (126 MHz, DMSO-d_6):

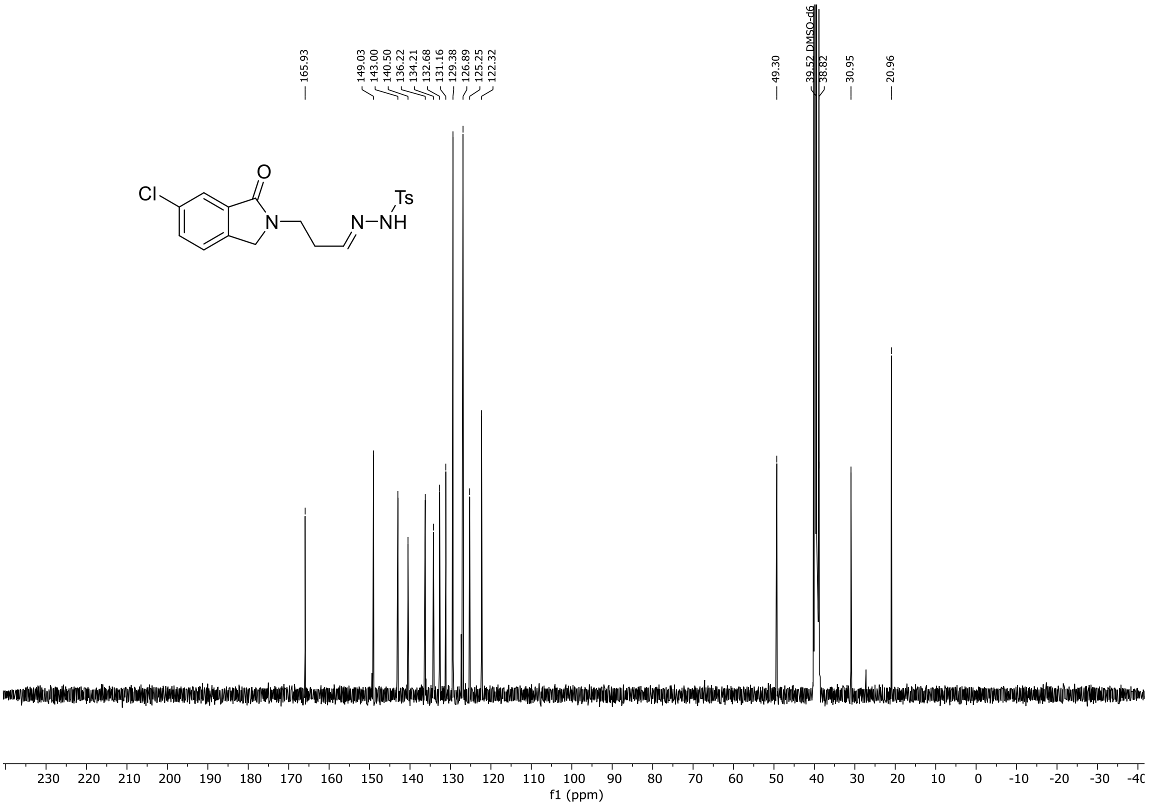

*8.2.6. N'-(3-(6-Bromo-1-oxoisoindolin-2-yl)propylidene)-4-methylbenzenesulfonohydrazide (**2f**)*

^{1}H NMR (500 MHz, DMSO-d_6):

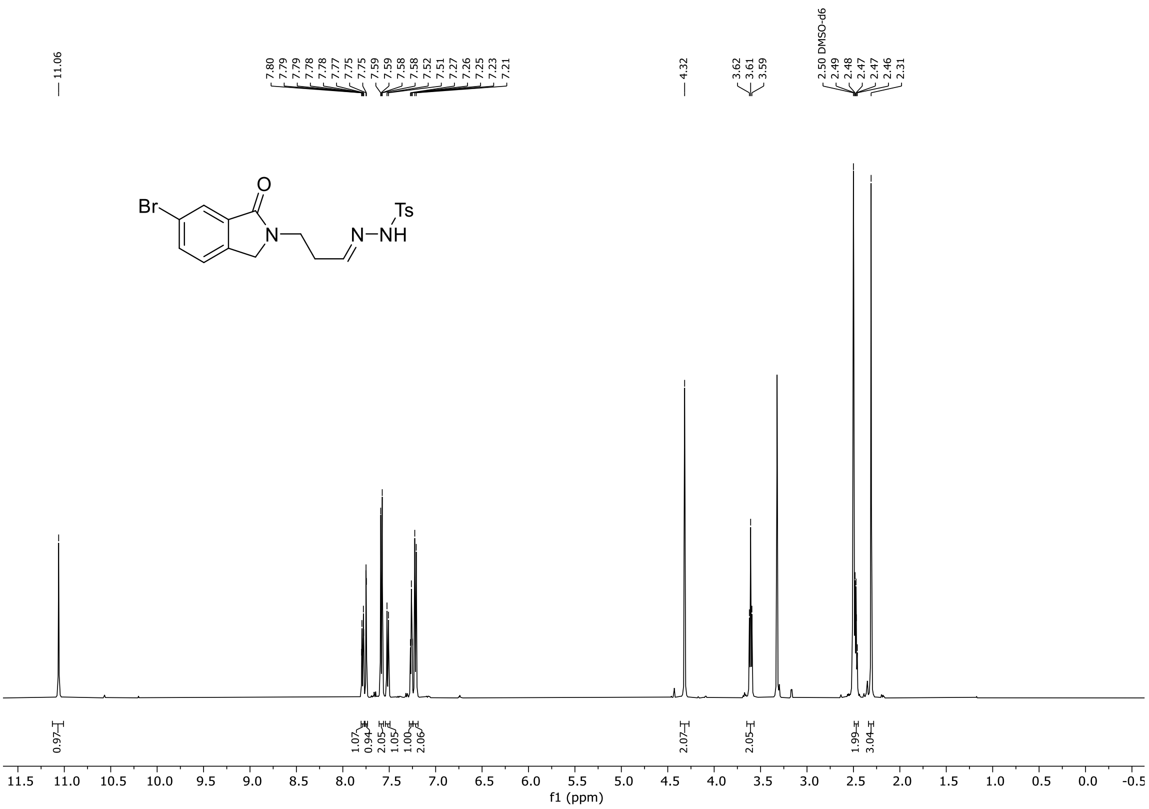

^{13}C NMR (126 MHz, DMSO-d_6):

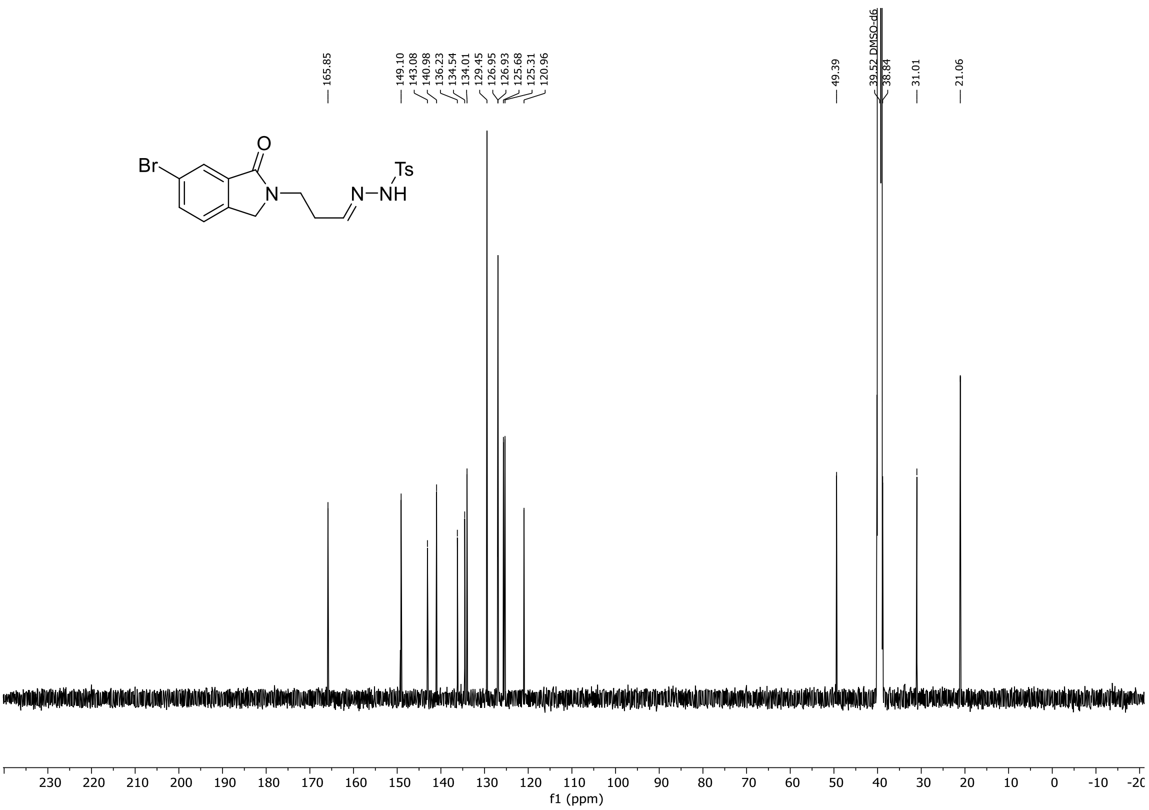

*8.2.7. N'-(3-(5-Fluoro-1-oxoisoindolin-2-yl)propylidene)-4-methylbenzenesulfonohydrazide (**2g**)*

^{1}H NMR (500 MHz, DMSO-d_6):

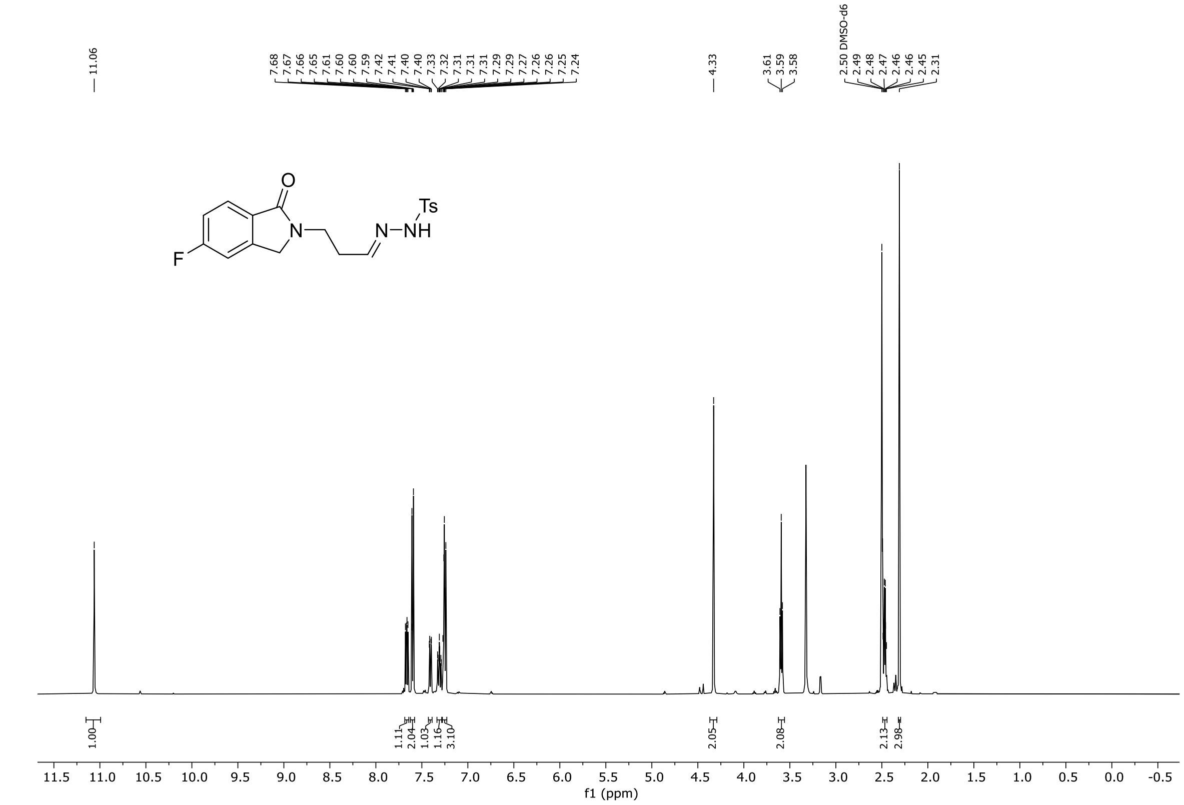

^{13}C NMR (126 MHz, DMSO-d_6):

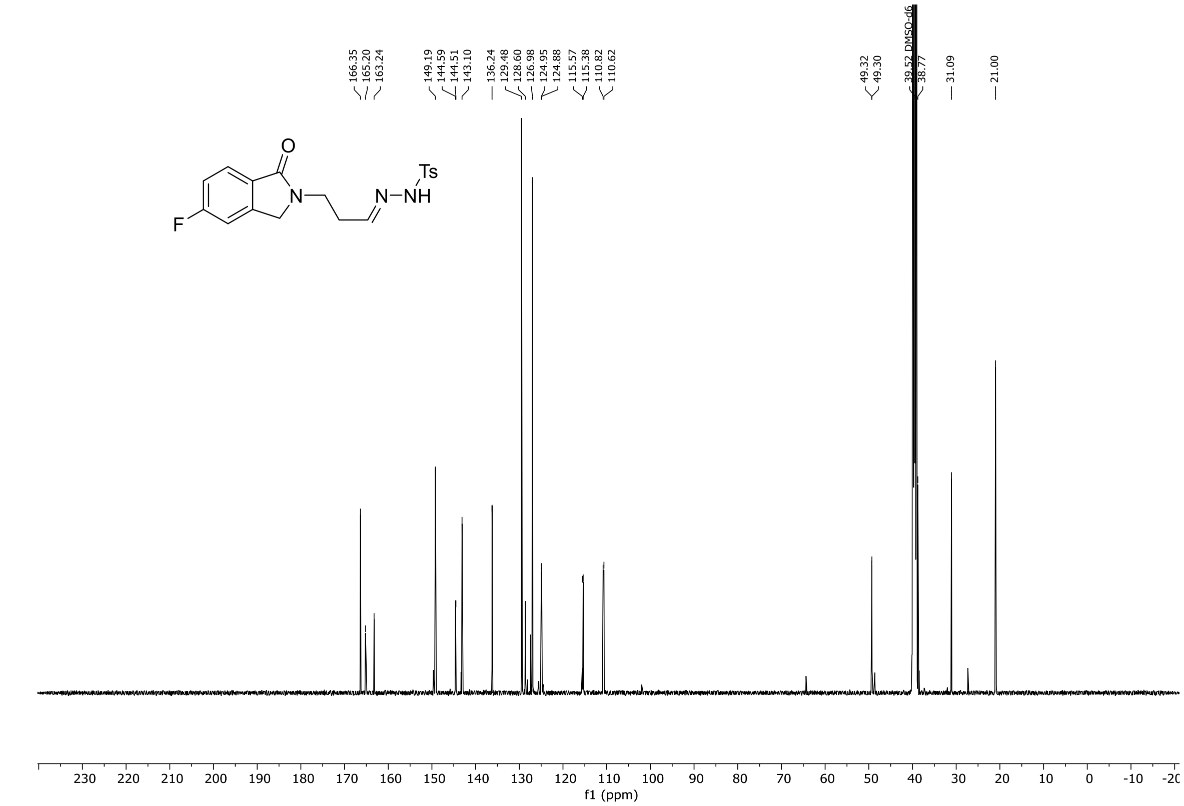

^{19}F NMR (376 MHz, DMSO-d_6):

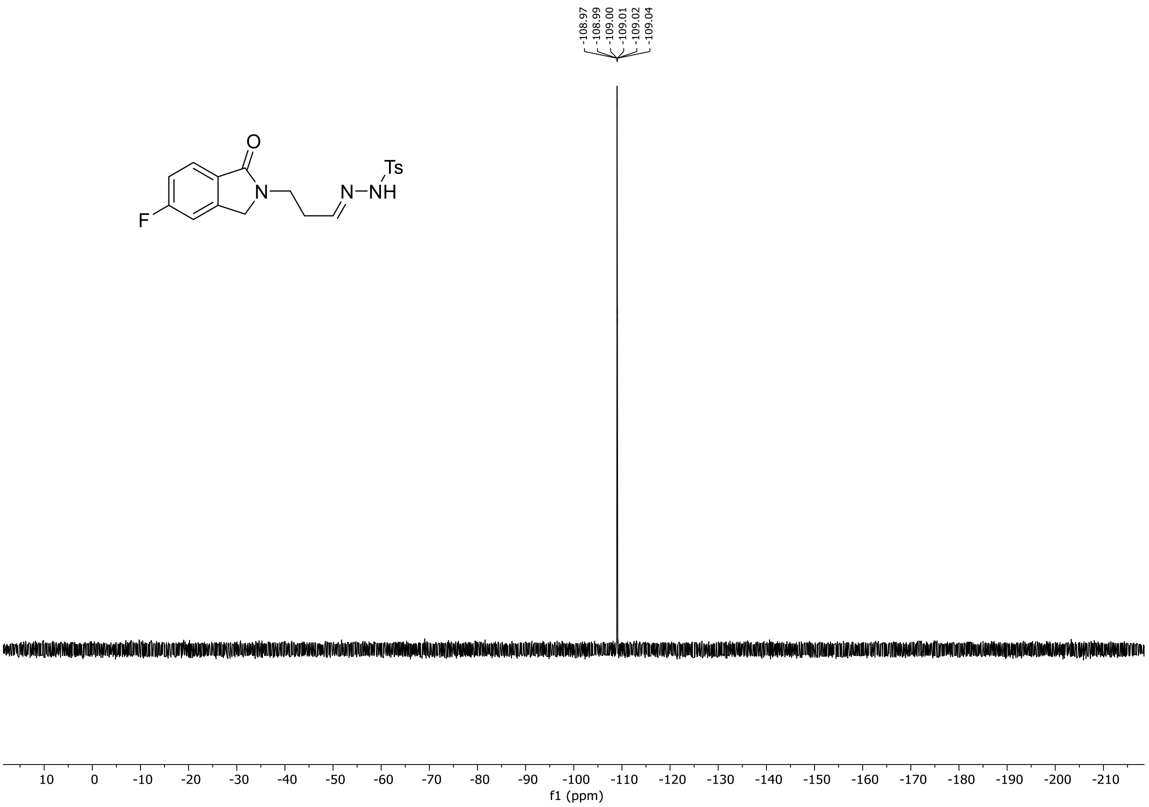

*8.2.8. N'-(3-(5-Bromo-1-oxoisoindolin-2-yl)propylidene)-4-methylbenzenesulfonohydrazide (**2h**)*

^{1}H NMR (500 MHz, DMSO-d_6):

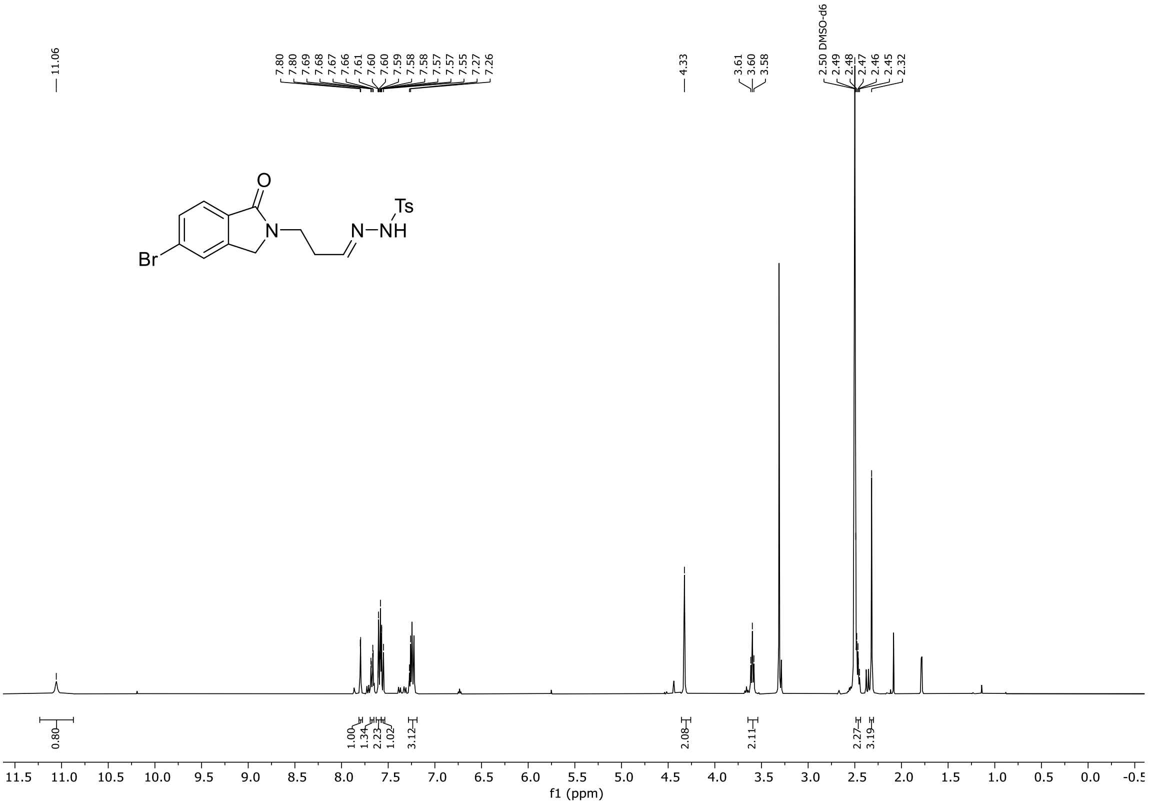

^{13}C NMR (126 MHz, DMSO-d_6):

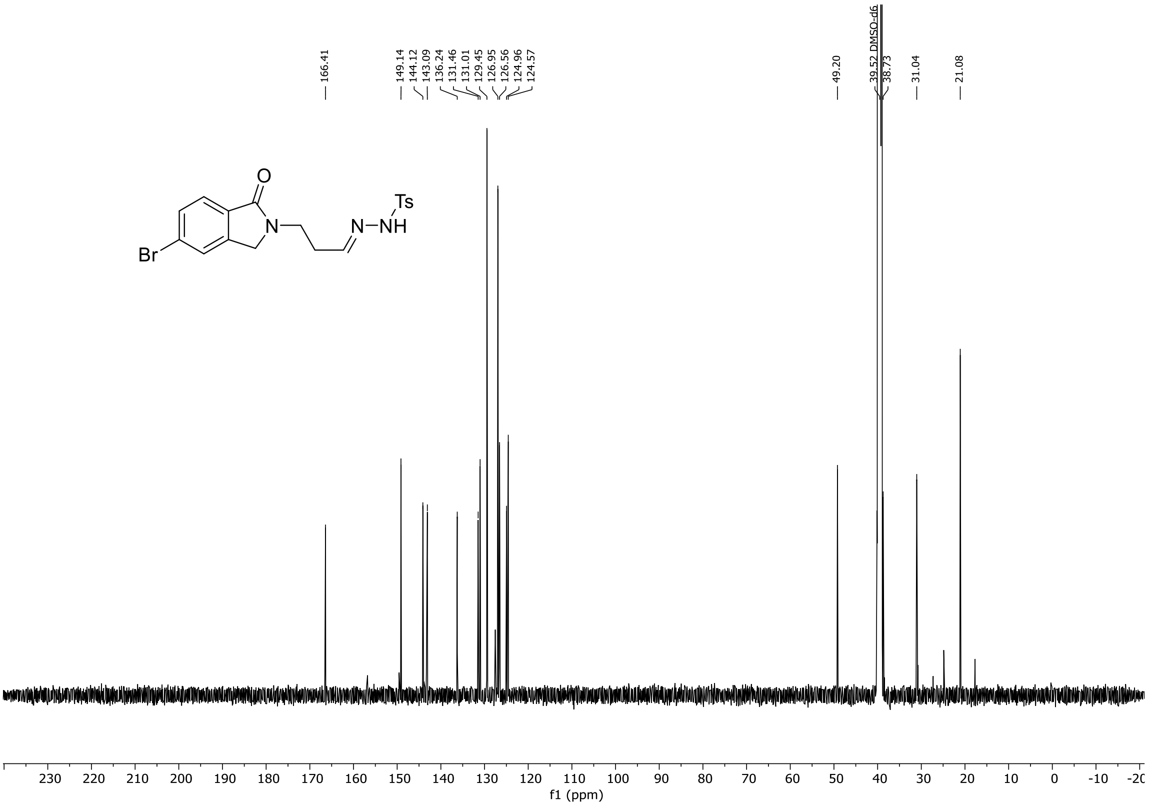

*8.2.9. N'-(3-(5-Nitro-1-oxoisoindolin-2-yl)propylidene)-4-methylbenzenesulfonohydrazide (**2i**)*

^{1}H NMR (500 MHz, DMSO-d_6):

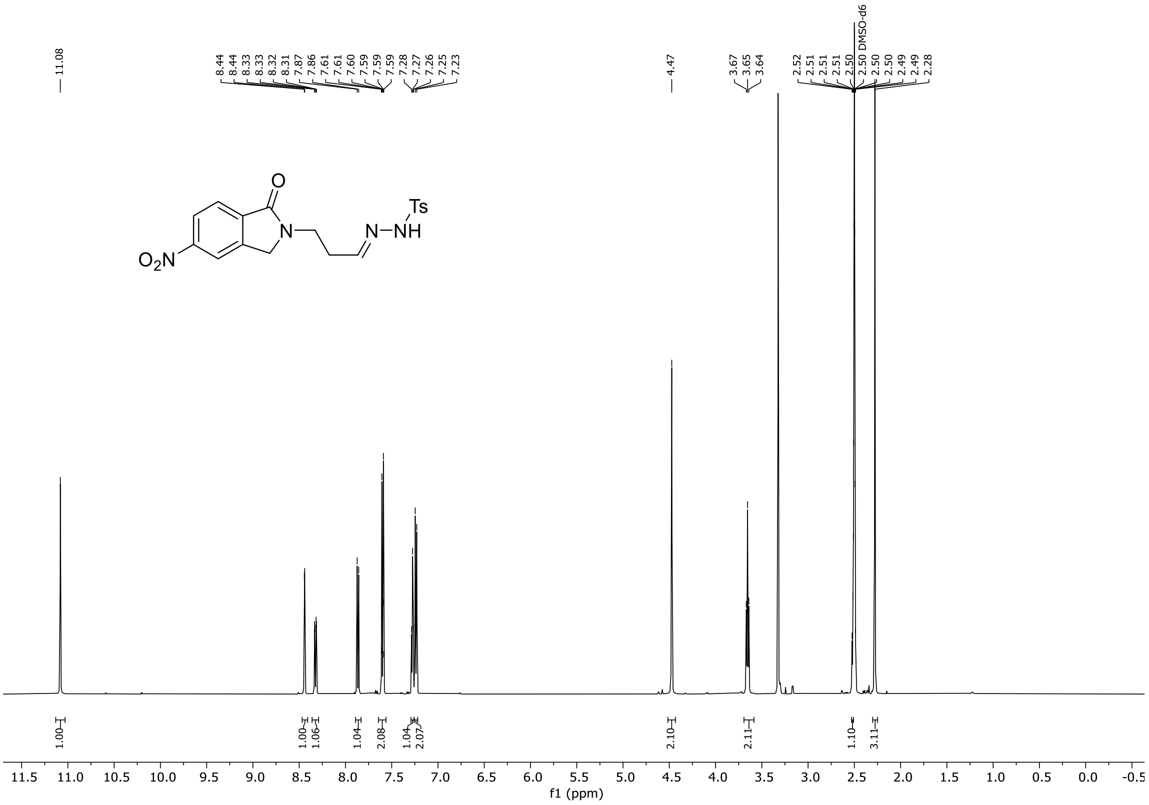

^{13}C NMR (126 MHz, DMSO-d_6):

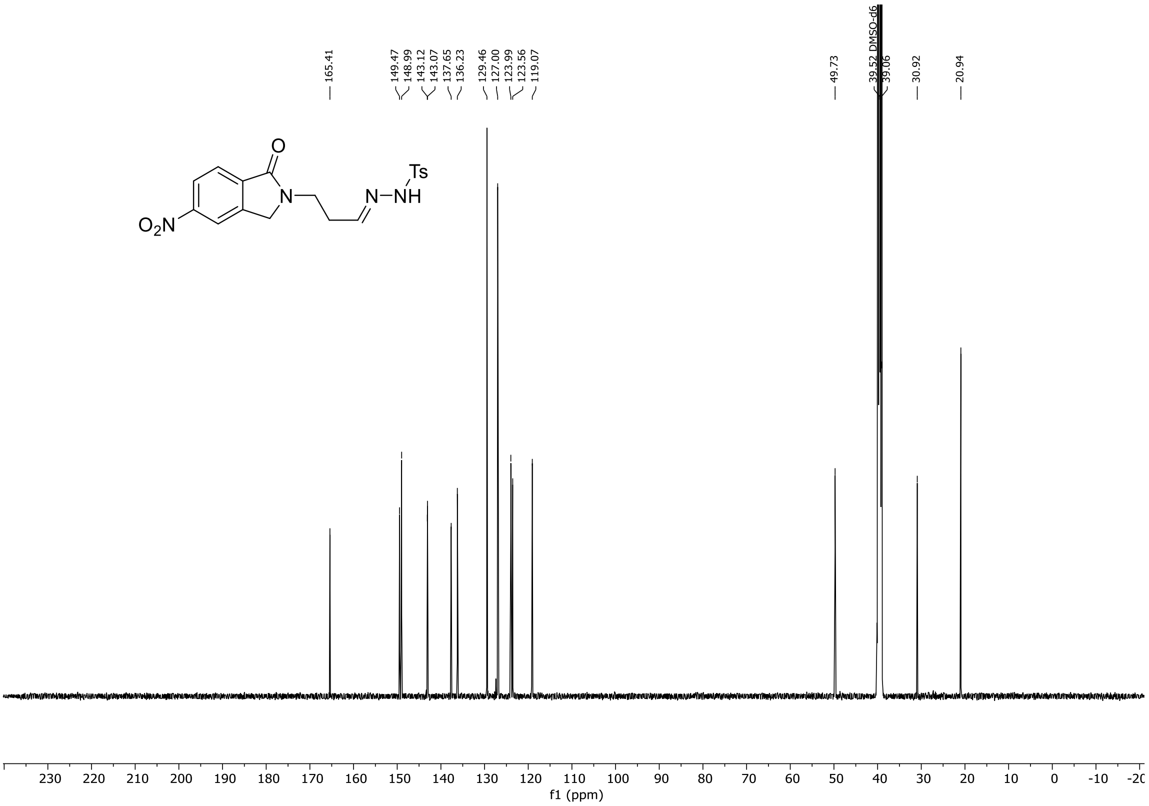

*8.2.10. N'-(3-(5-Cyano-1-oxoisoindolin-2-yl)propylidene)-4-methylbenzenesulfonohydrazide (**2j**)*

^{1}H NMR (500 MHz, DMSO-d_6):

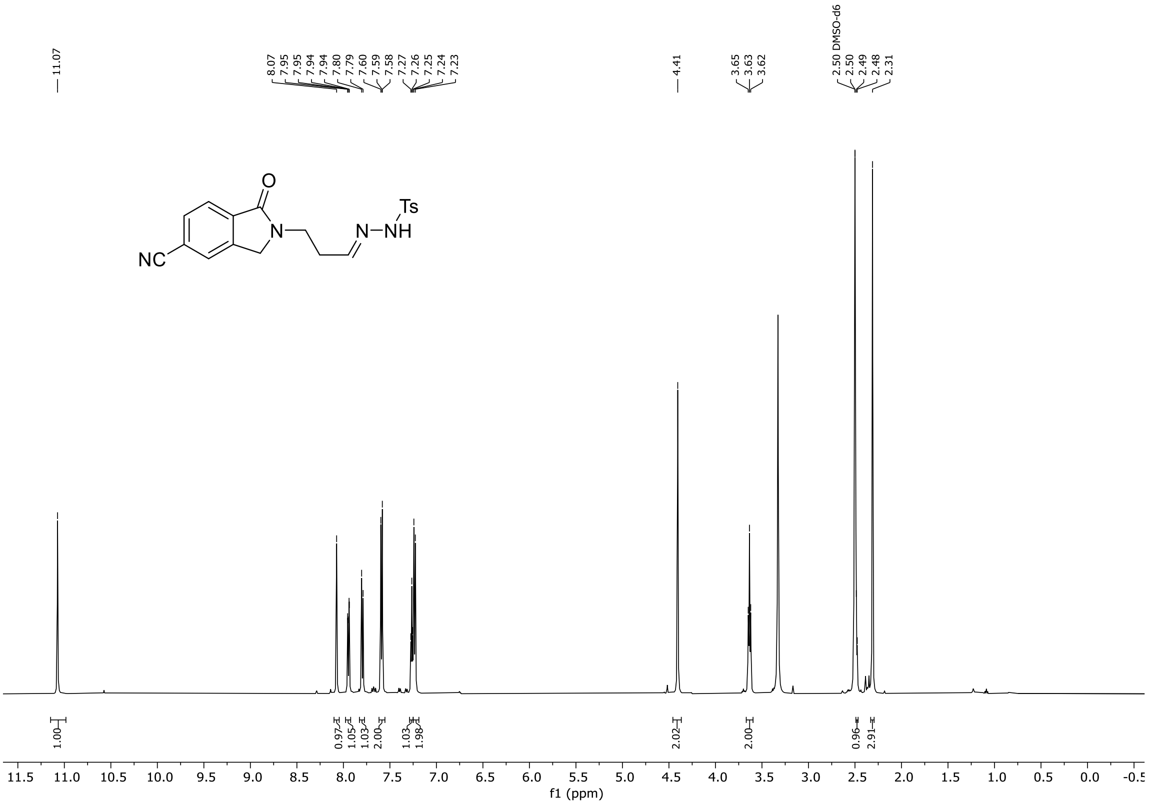

^{13}C NMR (126 MHz, DMSO-d_6):

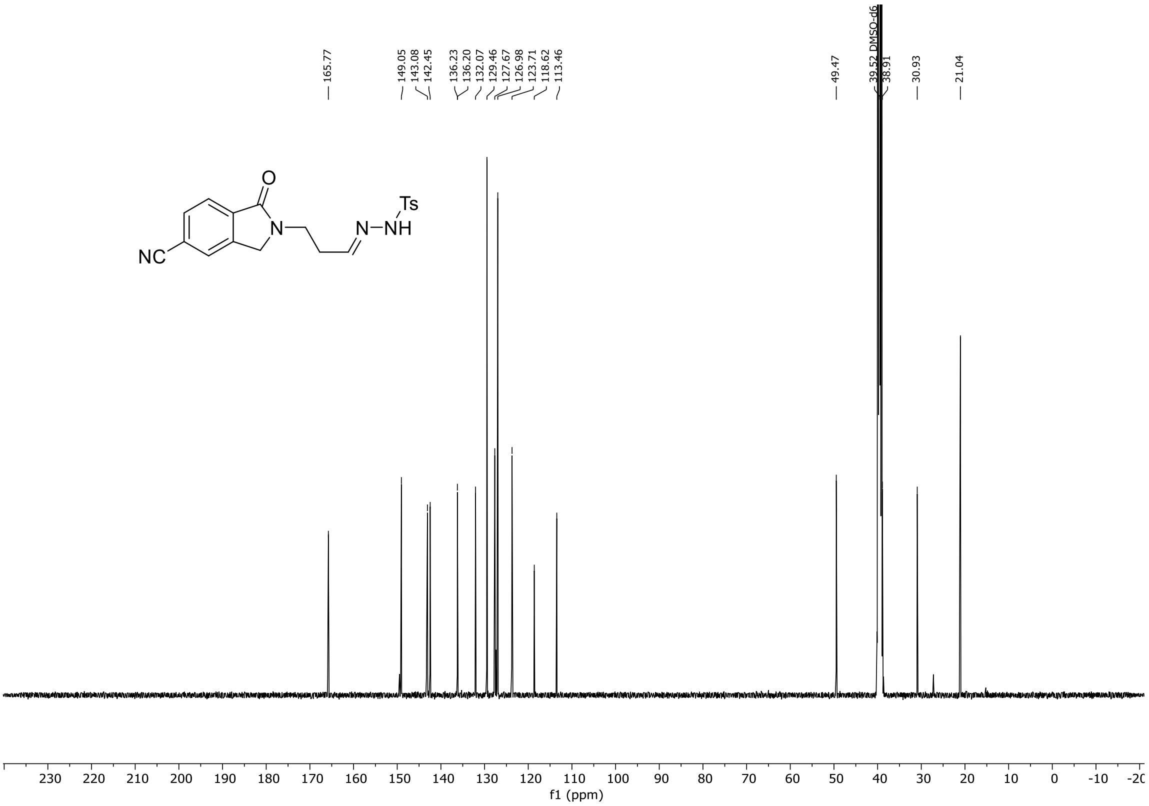

*8.2.11. Methyl 1-oxo-2-(3-(2-tosylhydrazineylidene)propyl)isoindoline-5-carboxylate (**2k**)*

^{1}H NMR (500 MHz, DMSO-d_6):

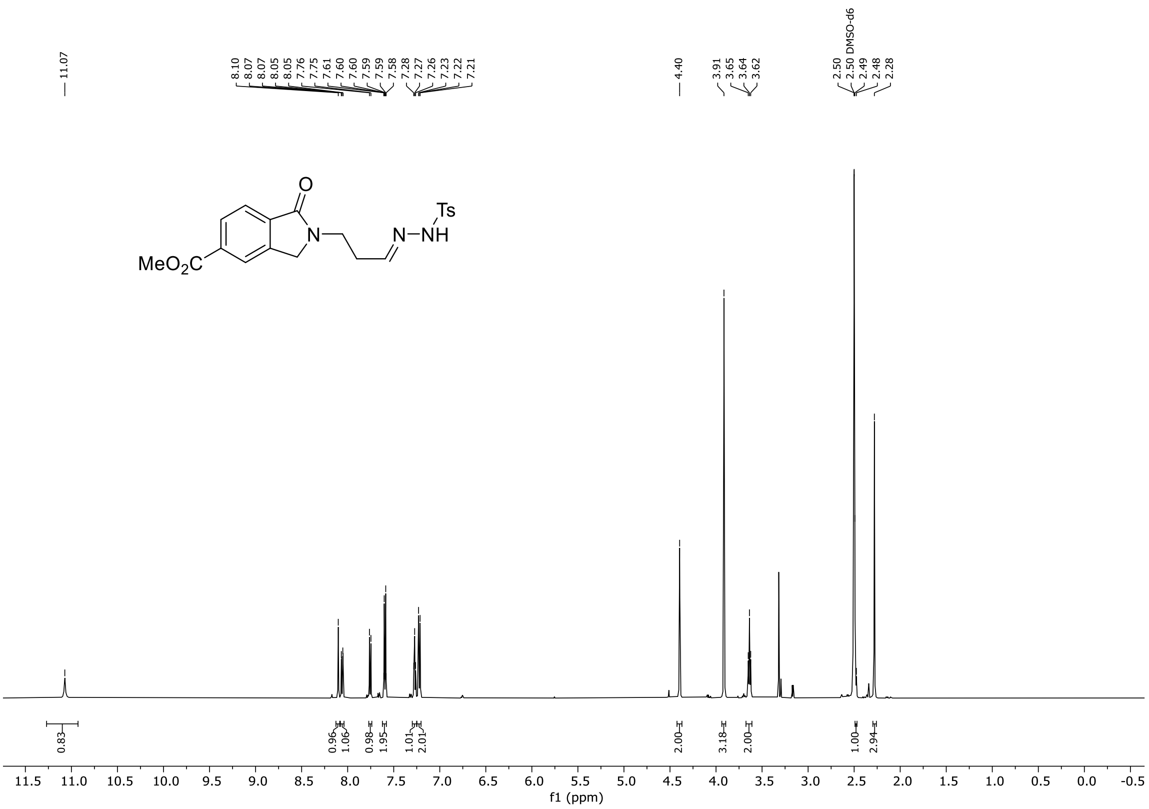

^{13}C NMR (126 MHz, DMSO-d_6):

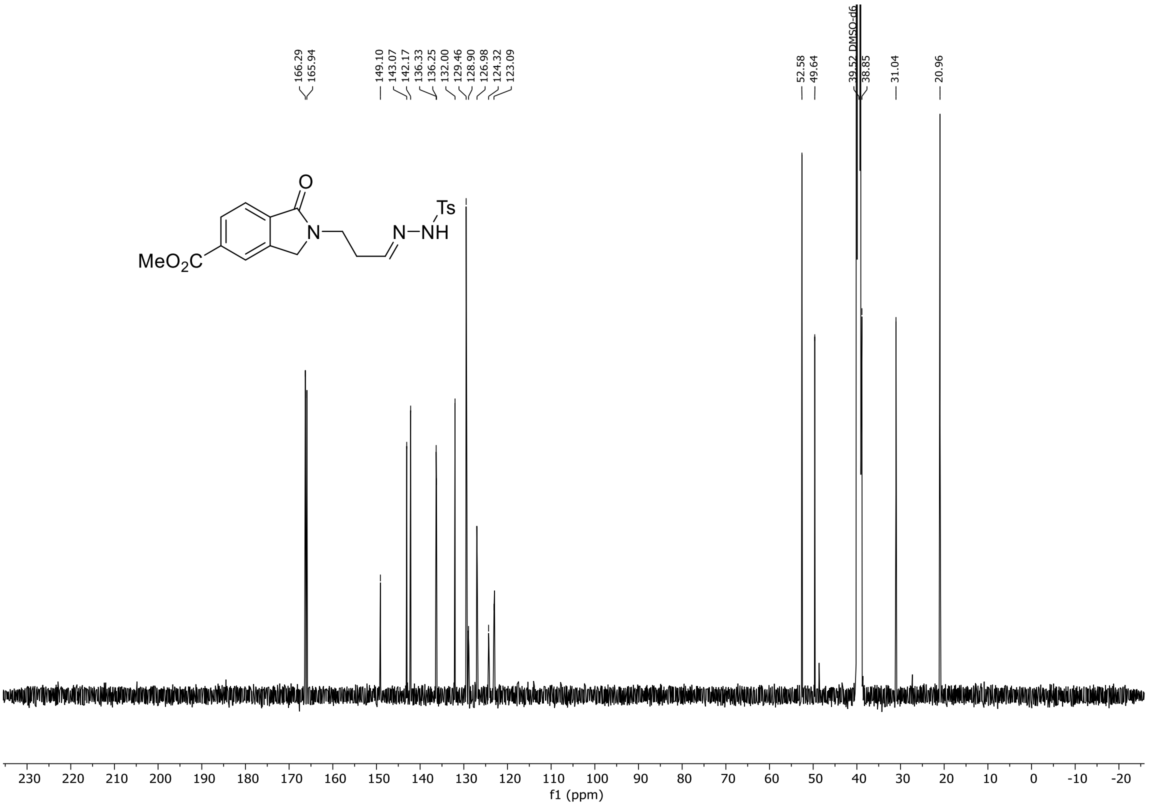

*8.2.12. N'-(3-(4-Methoxy-1-oxoisoindolin-2-yl)propylidene)-4-methylbenzenesulfonohydrazide (**2l**)*

^{1}H NMR (500 MHz, DMSO-d_6):

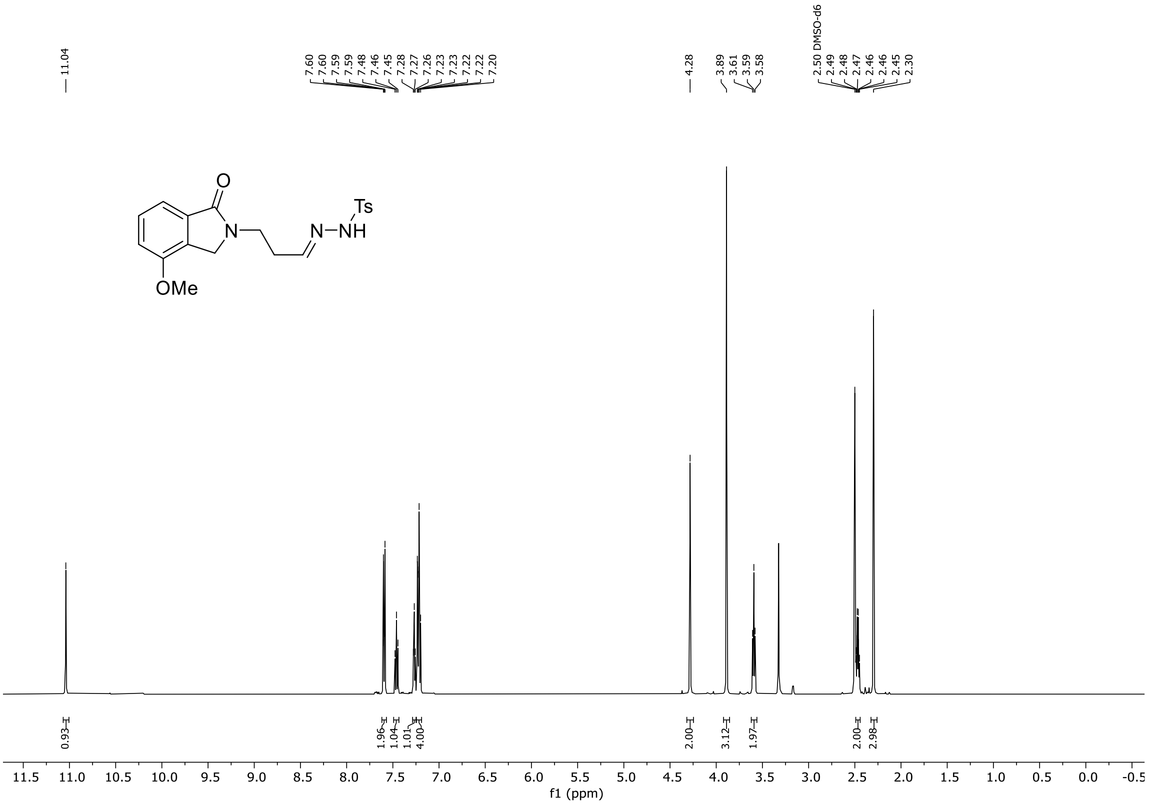

^{13}C NMR (126 MHz, DMSO-d_6):

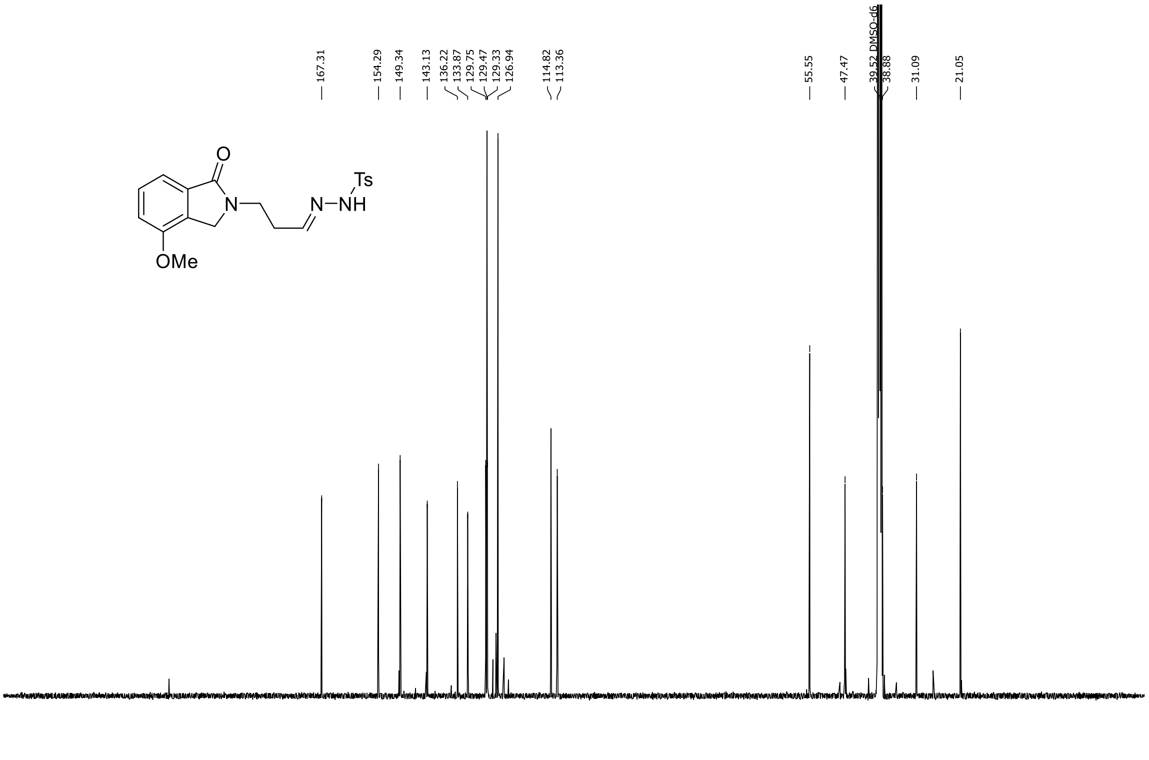

8.2.13. *N'-(3-(4-Bromo-1-oxoisoindolin-2-yl)propylidene)-4-methylbenzenesulfonohydrazide (**2m**)*

^{1}H NMR (500 MHz, DMSO-d_6):

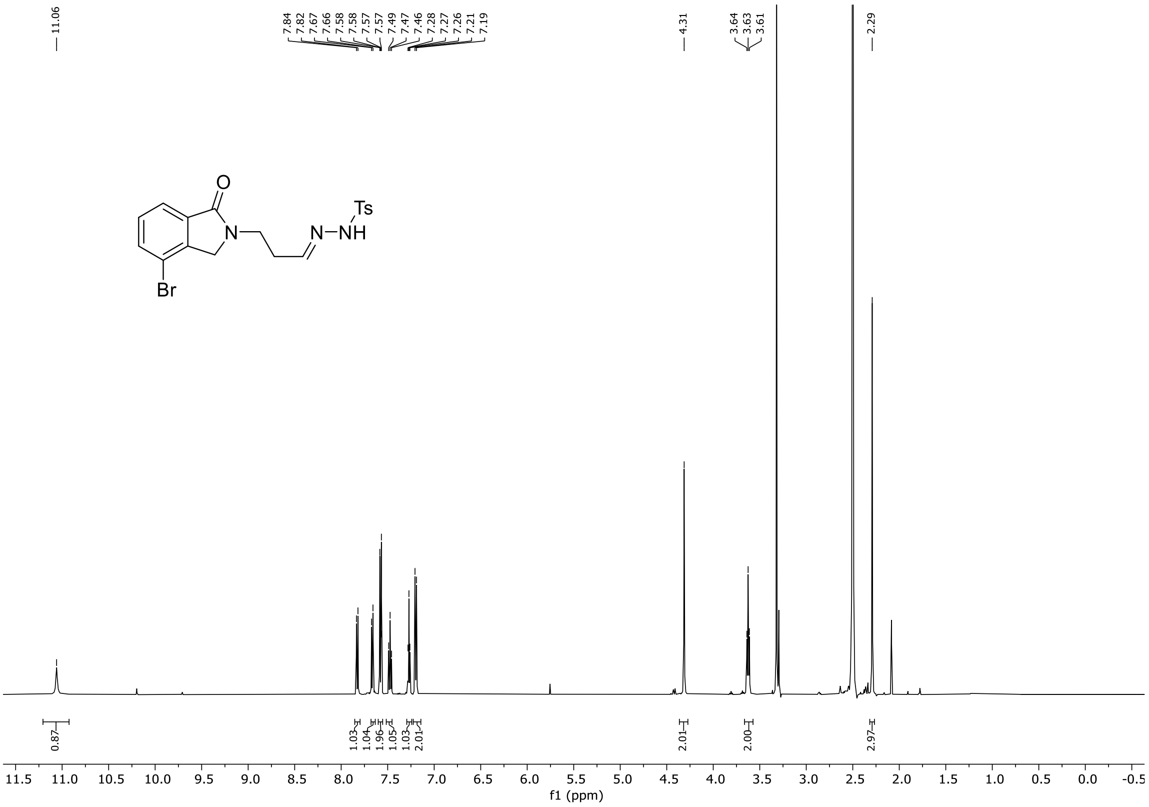

^{13}C NMR (126 MHz, DMSO-d_6):

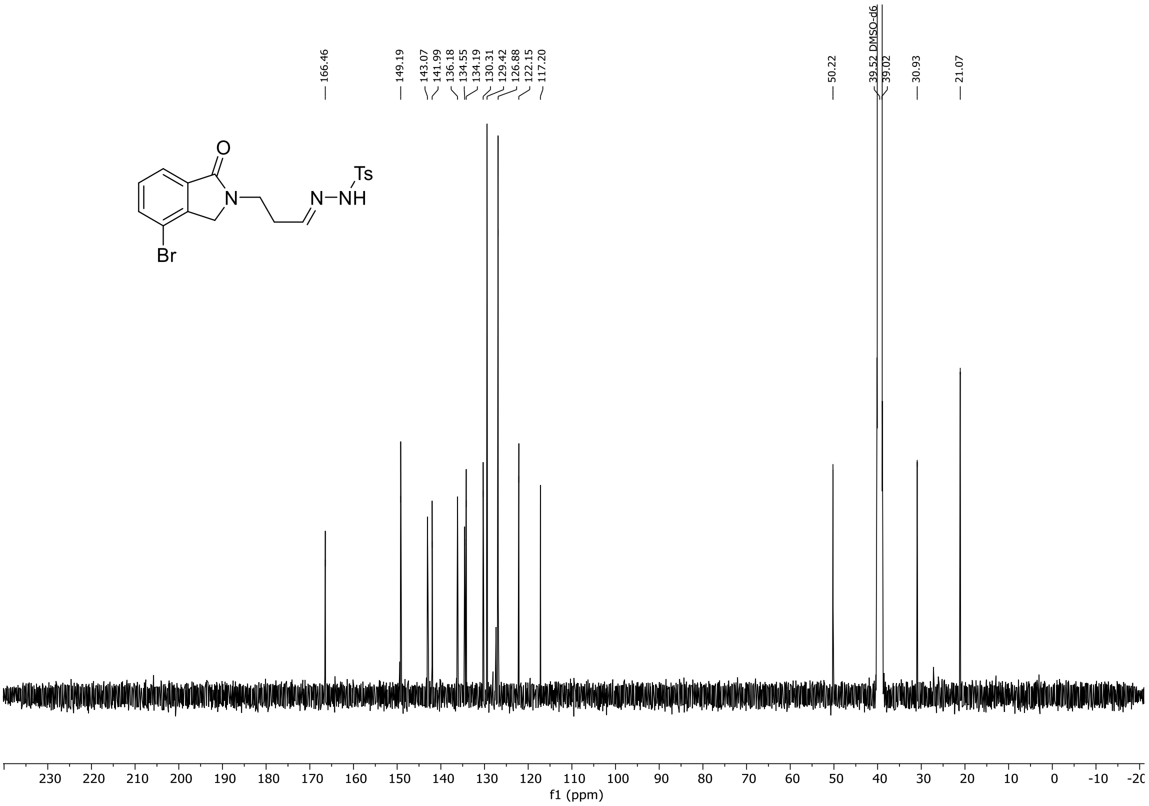

*8.2.14. N'-(3-(1-Oxo-4-(trifluoromethyl)isoindolin-2-yl)propylidene)-4-methylbenzenesulfonohydrazide (**2n**)*

^{1}H NMR (500 MHz, DMSO-d_6):

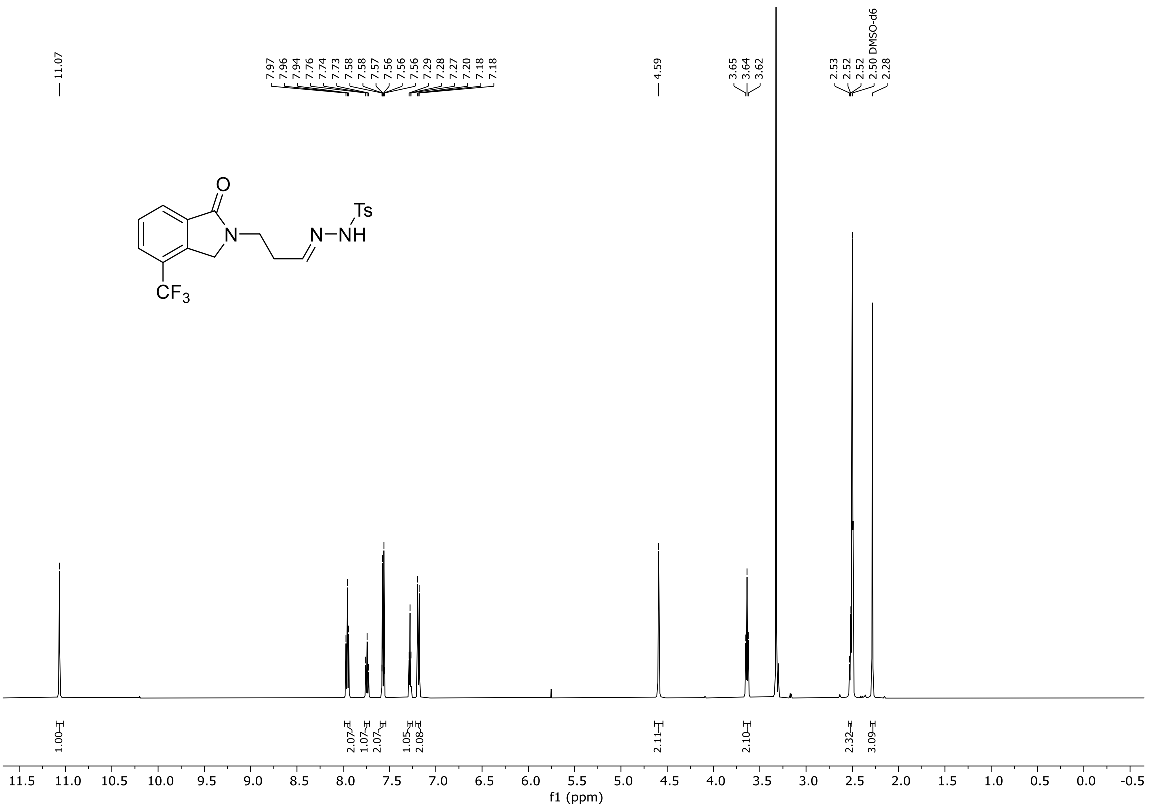

^{13}C NMR (126 MHz, DMSO-d_6):

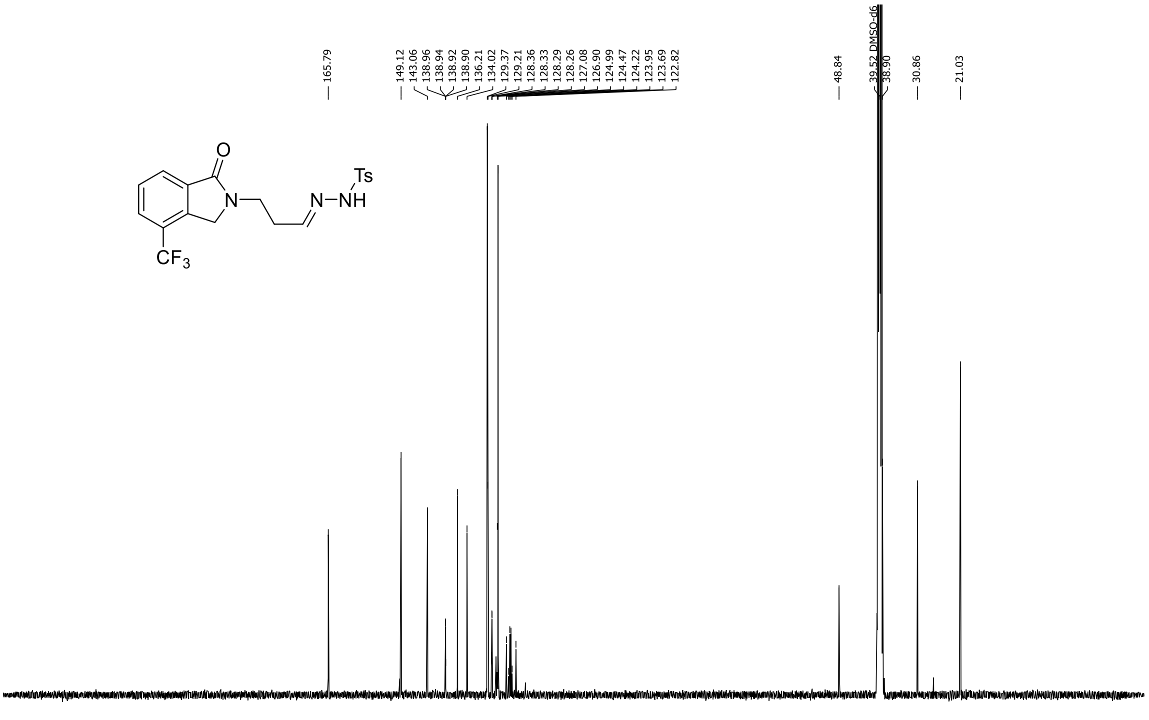

^{19}F NMR (376 MHz, DMSO-d_6):

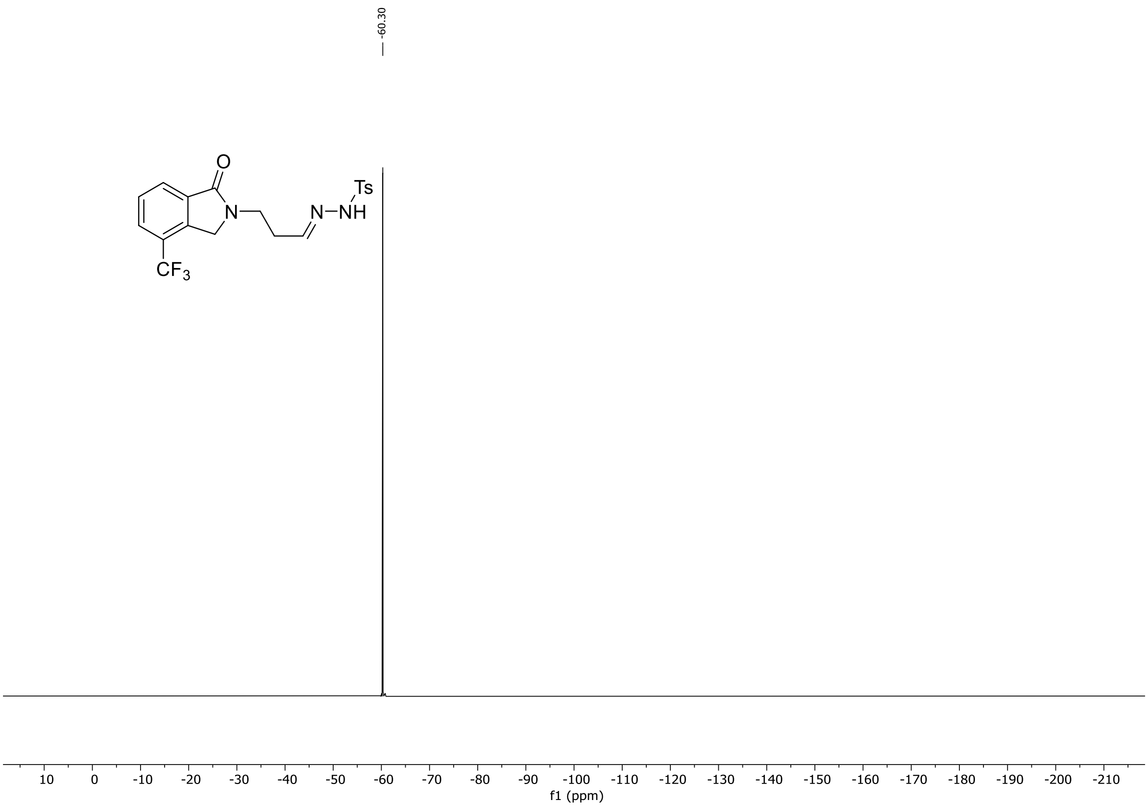

*8.2.15. N'-(3-(1-Methyl-3-oxoisoindolin-2-yl)propylidene)-4-methyl-benzenesulfonohydrazide (**2o**)*

^{1}H NMR (500 MHz, DMSO-d_6):

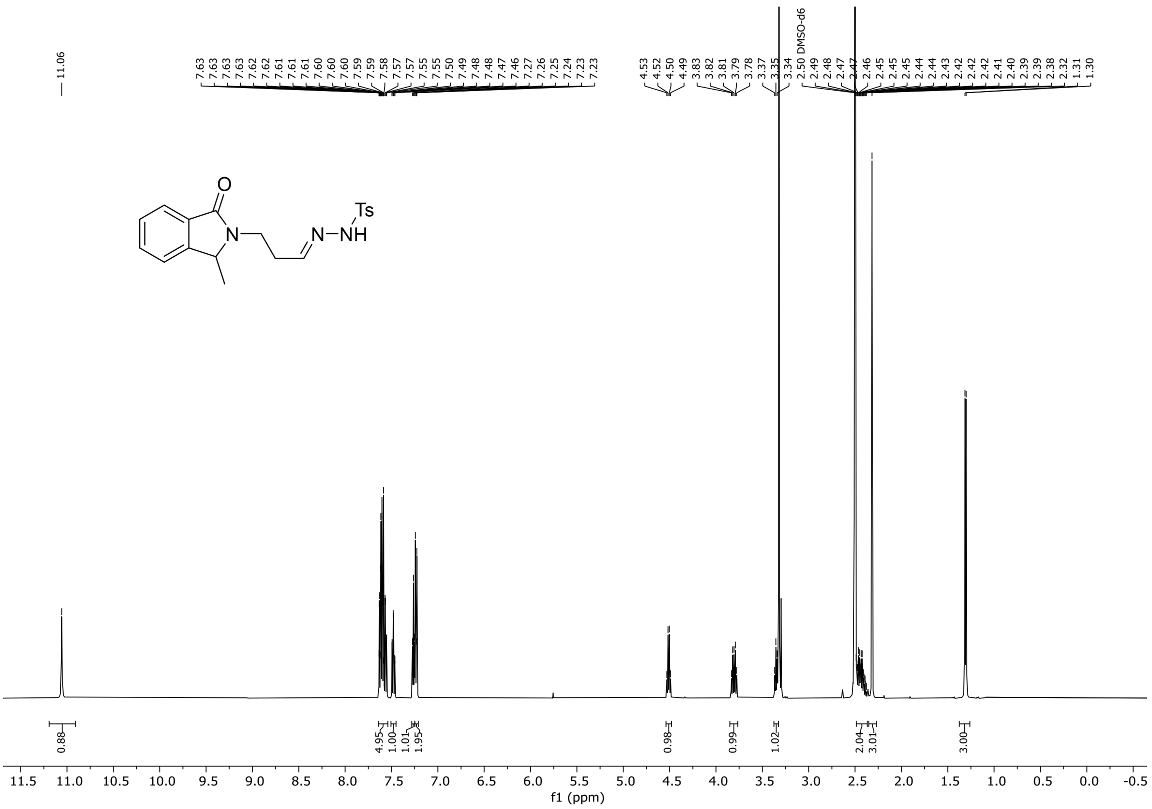

^{13}C NMR (126 MHz, DMSO-d_6):

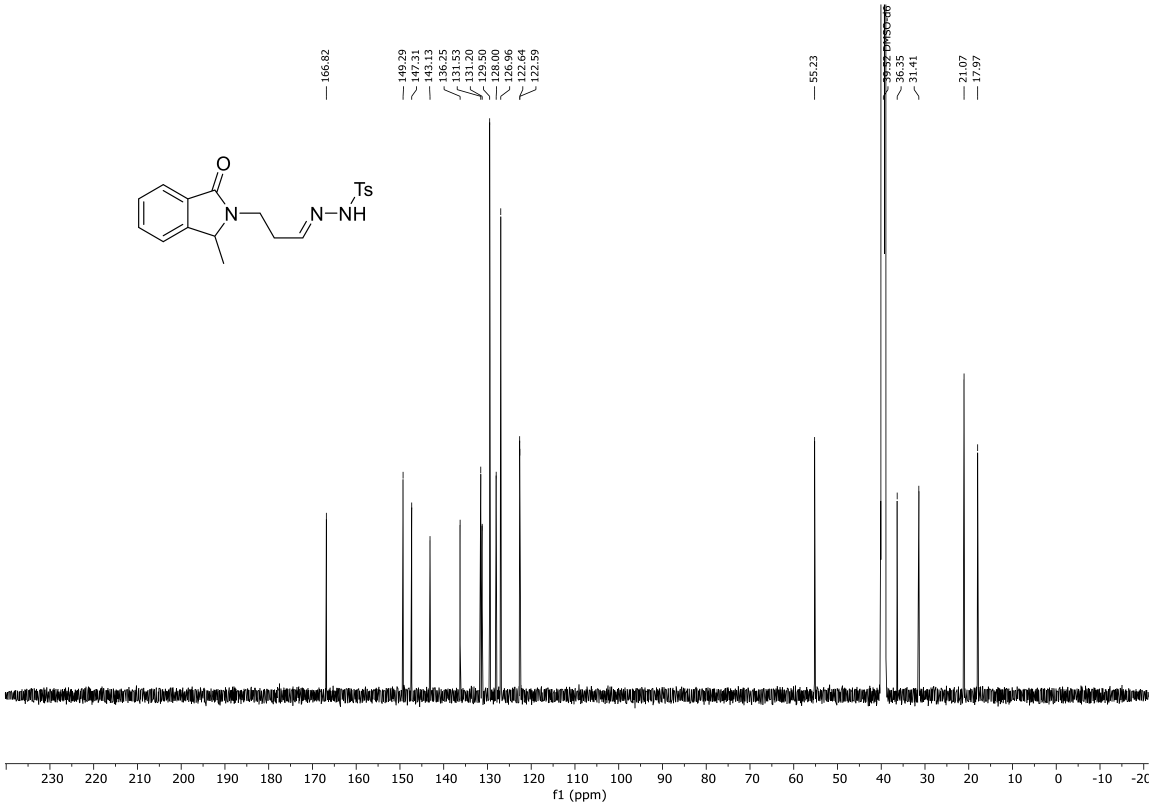

*8.2.16. N'-(3-(5-Oxo-5,7-dihydro-6H-pyrrolo[3,4-b]pyridin-6-yl)-4-methyl-propylidene)-4-methylbenzenesulfonohydrazide (**2p**)*

^{1}H NMR (500 MHz, DMSO-d_6):

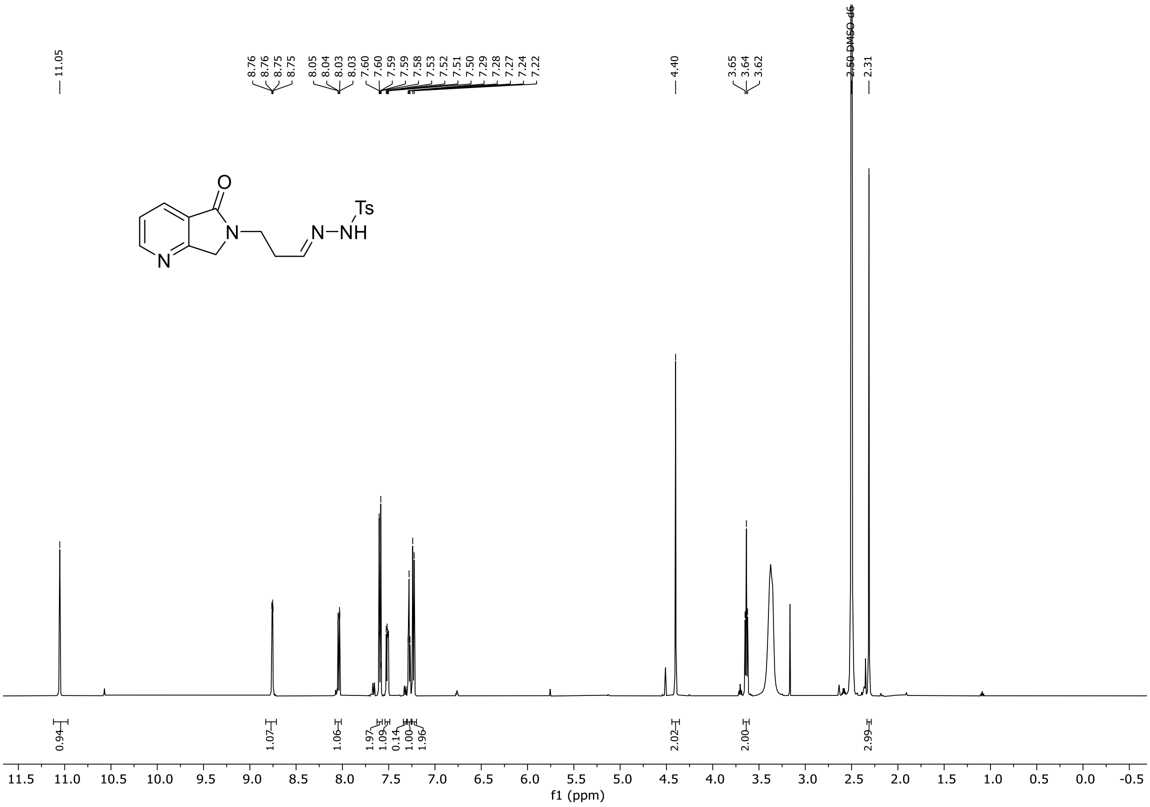

^{13}C NMR (126 MHz, DMSO-d_6):

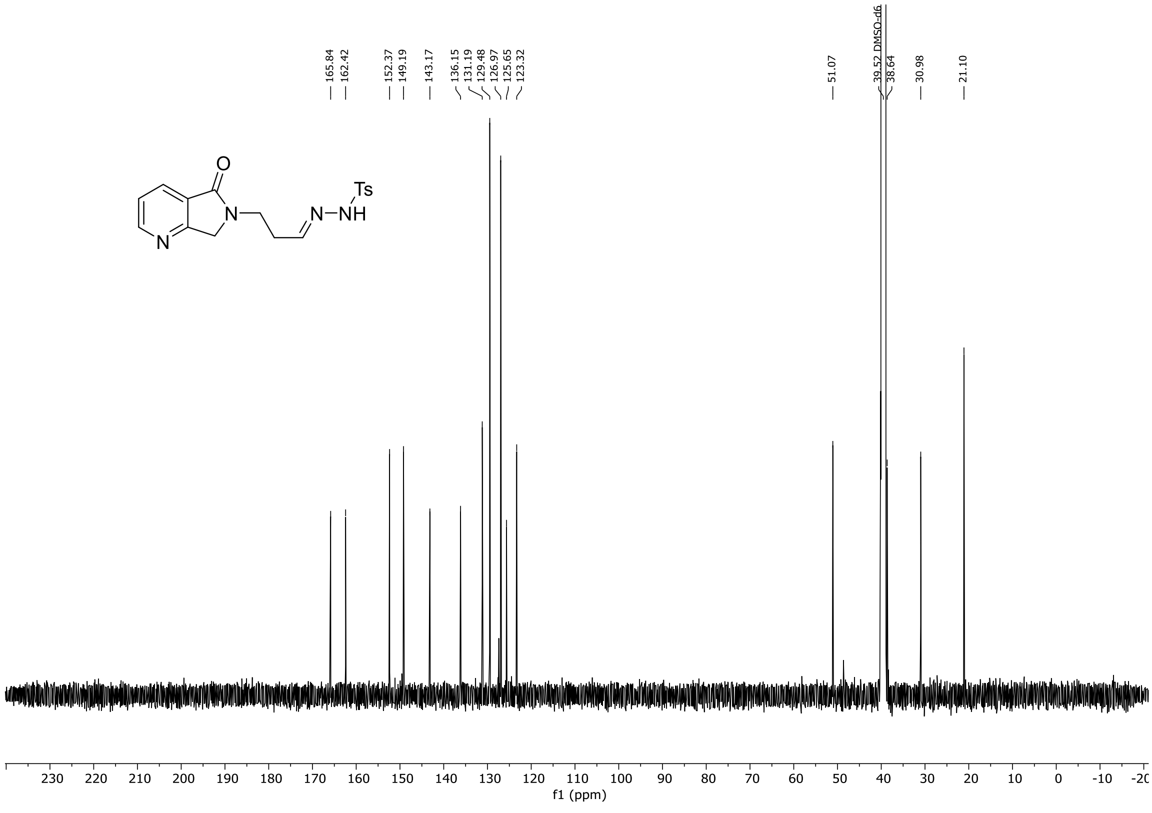

*8.2.17. N'-(4-(1-Oxoisoindolin-2-yl)butylidene)-4-methylbenzenesulfonohydrazide (**2q**)*

^{1}H NMR (500 MHz, DMSO-d_6):

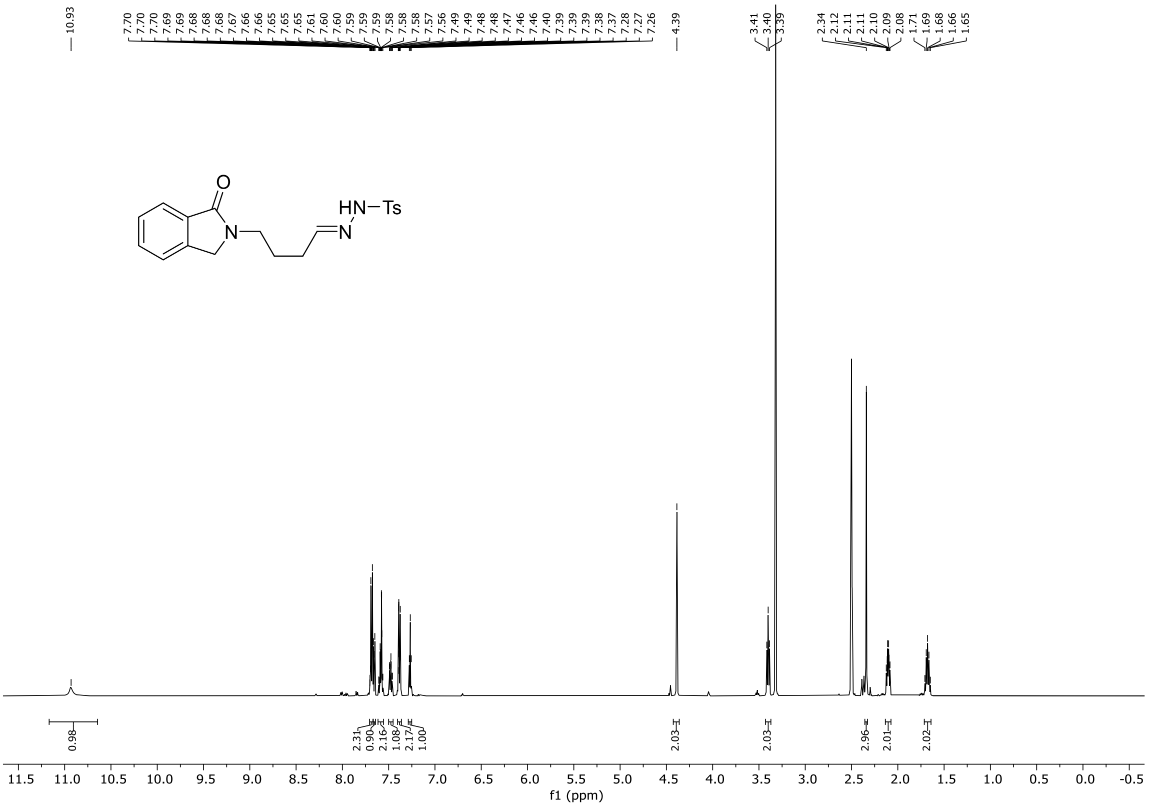

^{13}C NMR (126 MHz, DMSO-d_6):

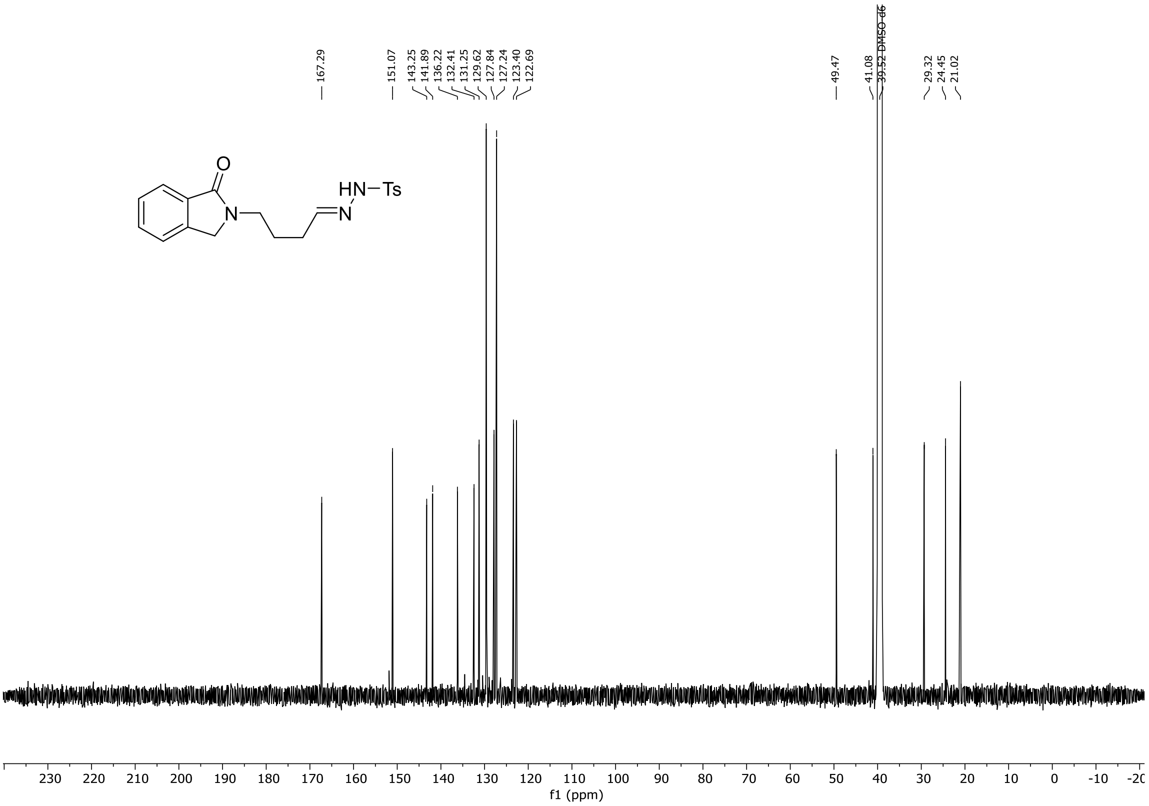

*8.2.18. N'-(2-((1-Oxoisoindolin-2-yl)methyl)benzylidene)-4-methylbenzenesulfonohydrazide (**2r**)*

^{1}H NMR (500 MHz, DMSO-d_6):

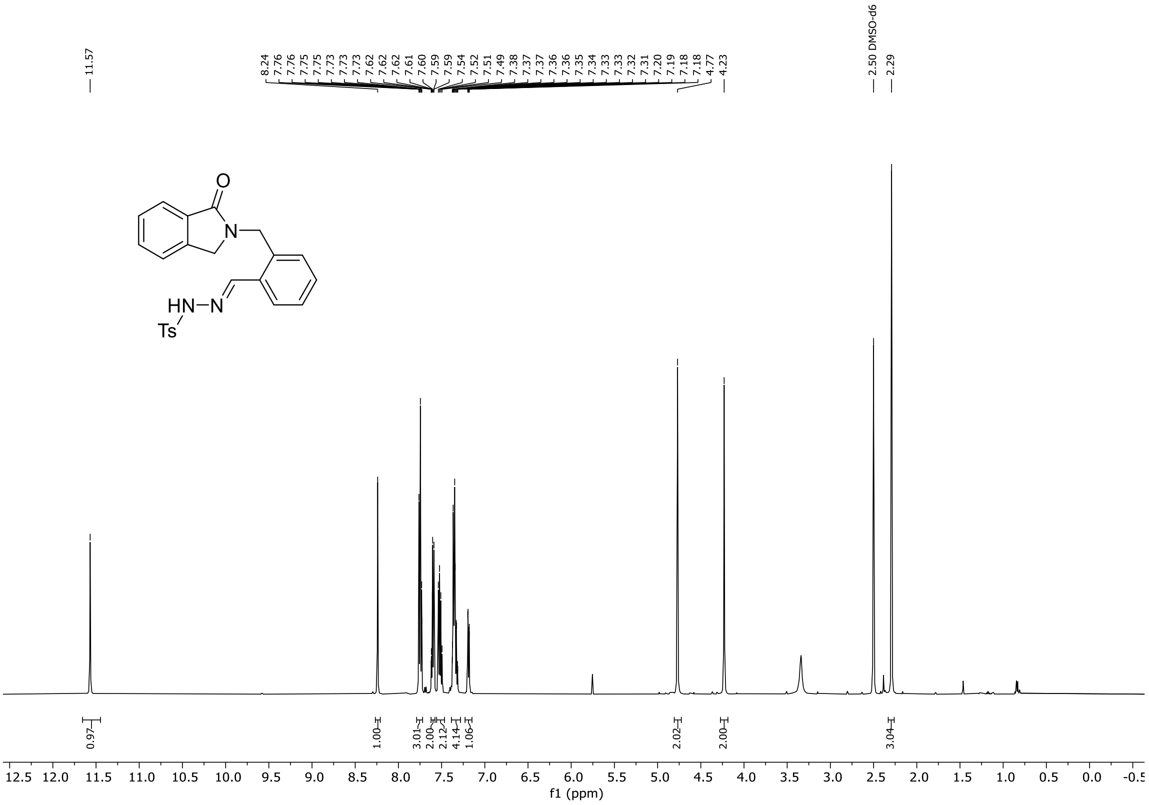

^{13}C NMR (126 MHz, DMSO-d_6):

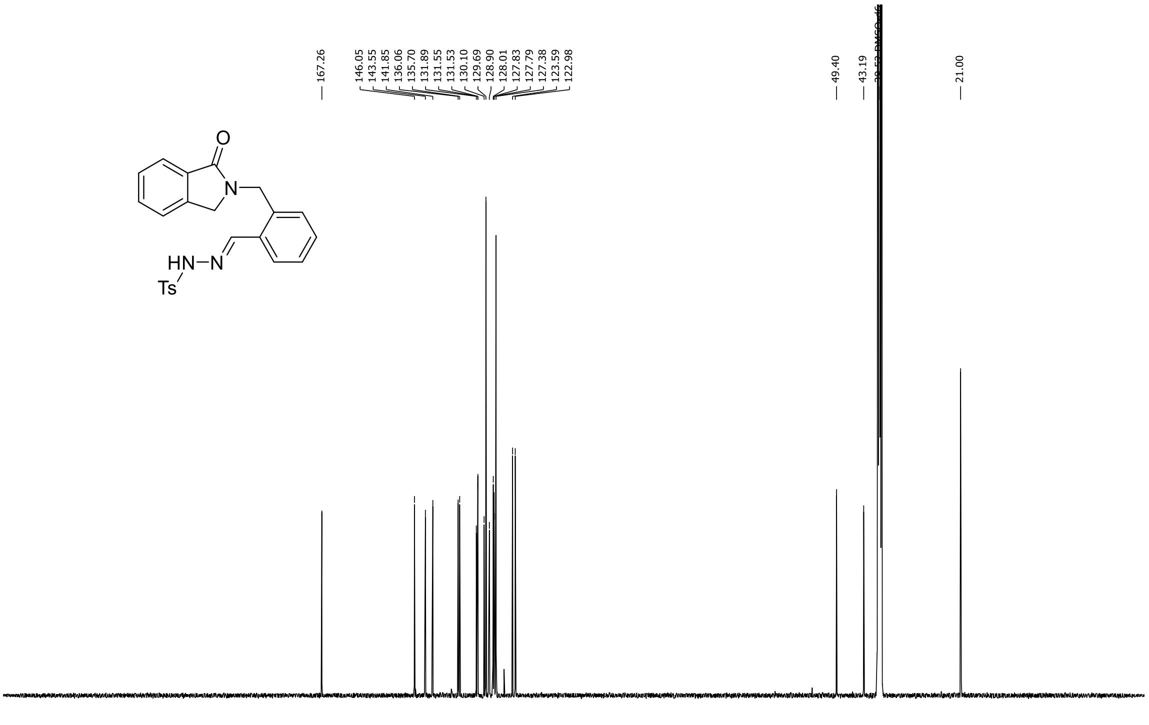

*8.2.19. N'-(2-(2-(1-Oxoisoindolin-2-yl)ethyl)benzylidene)-4-methylbenzenesulfonohydrazide (**2s**)*

^{1}H NMR (500 MHz, DMSO-d_6):

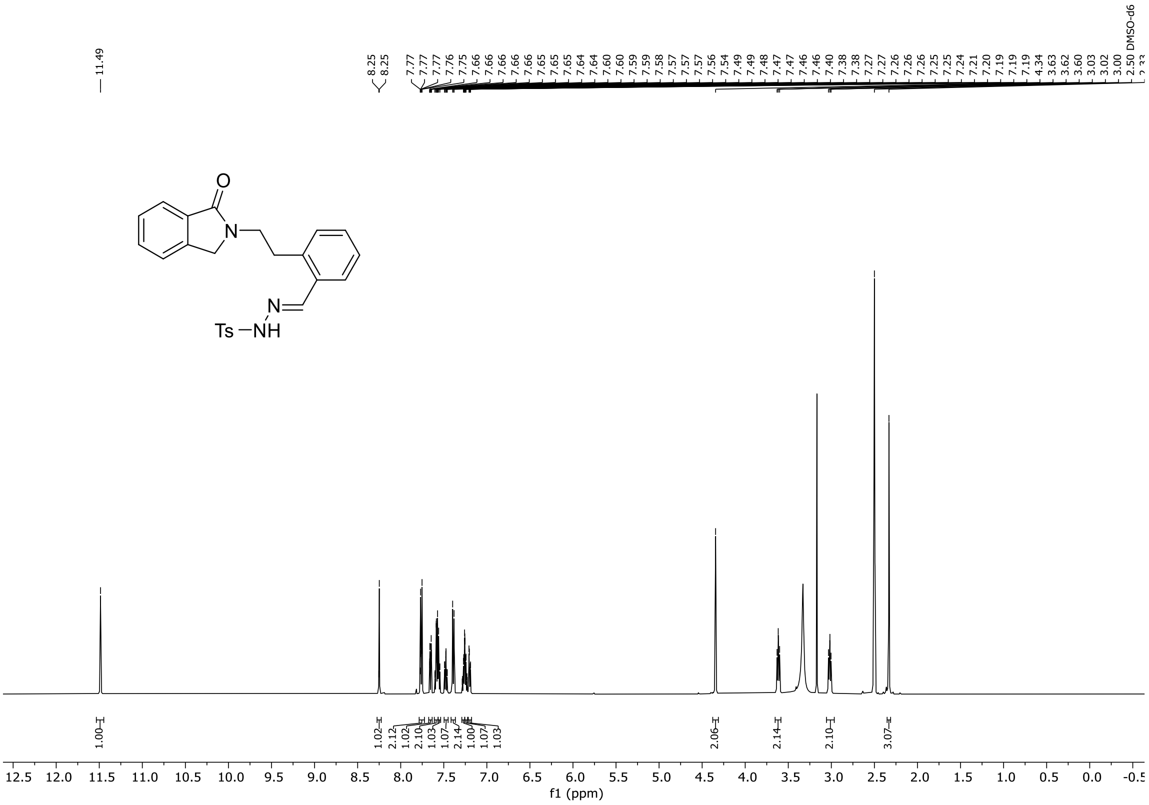

^{13}C NMR (126 MHz, DMSO-d_6):

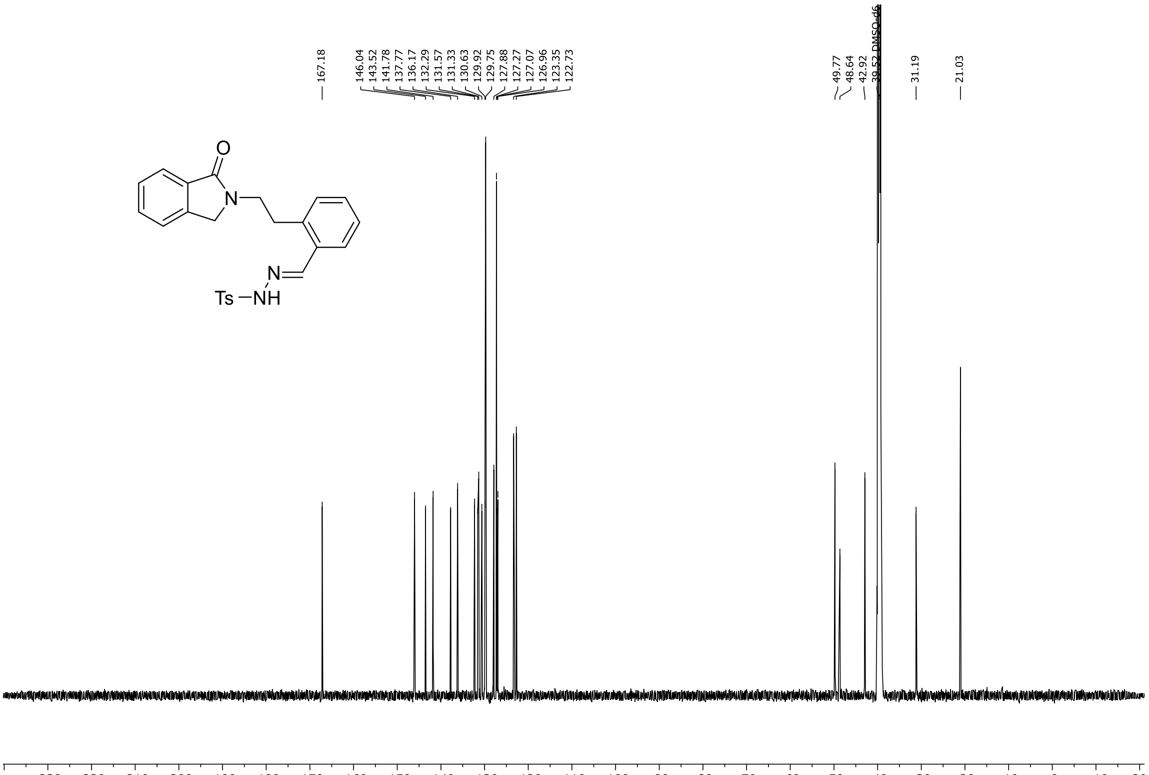

8.2.20. *N'-((6-(2-(6,7-dimethoxy-1-oxoisoindolin-2-yl)ethyl)benzo[d][1,3]dioxol-5-yl)methylene)-4-methylbenzenesulfonohydrazide (**2t**)*

^{1}H NMR (500 MHz, DMSO-d_6):

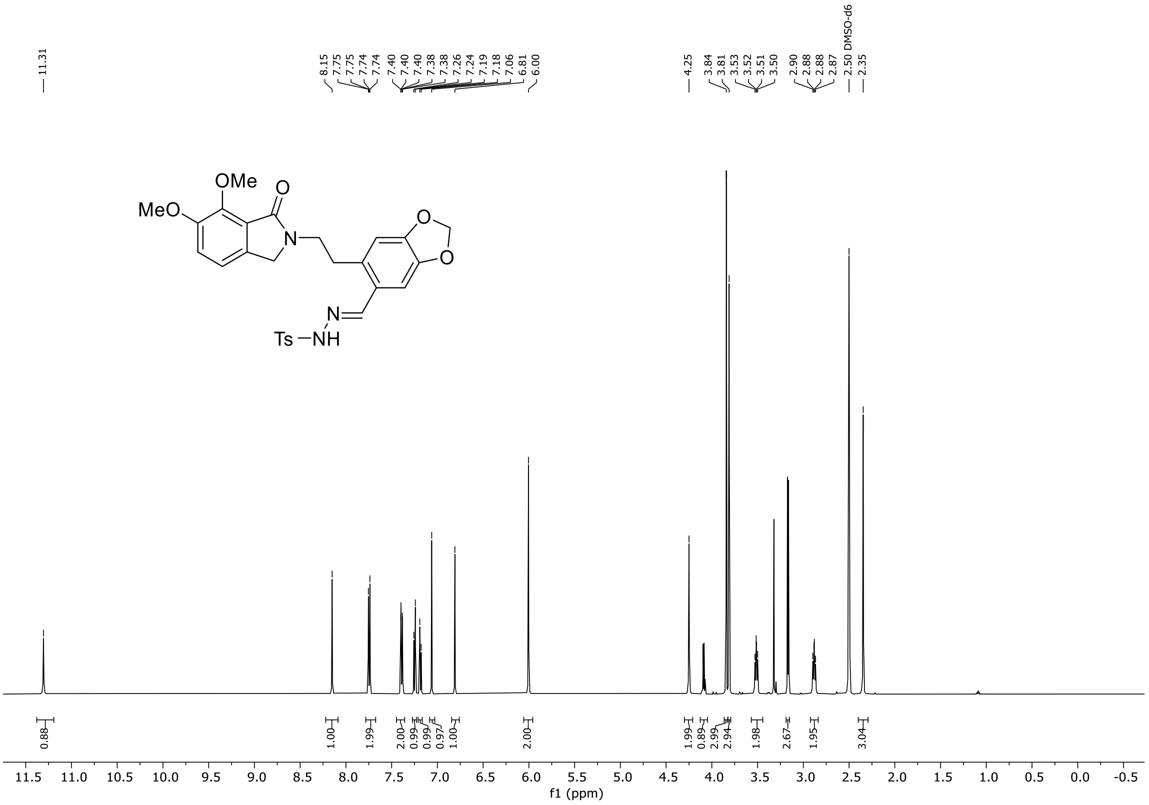

^{13}C NMR (126 MHz, DMSO-d_6):

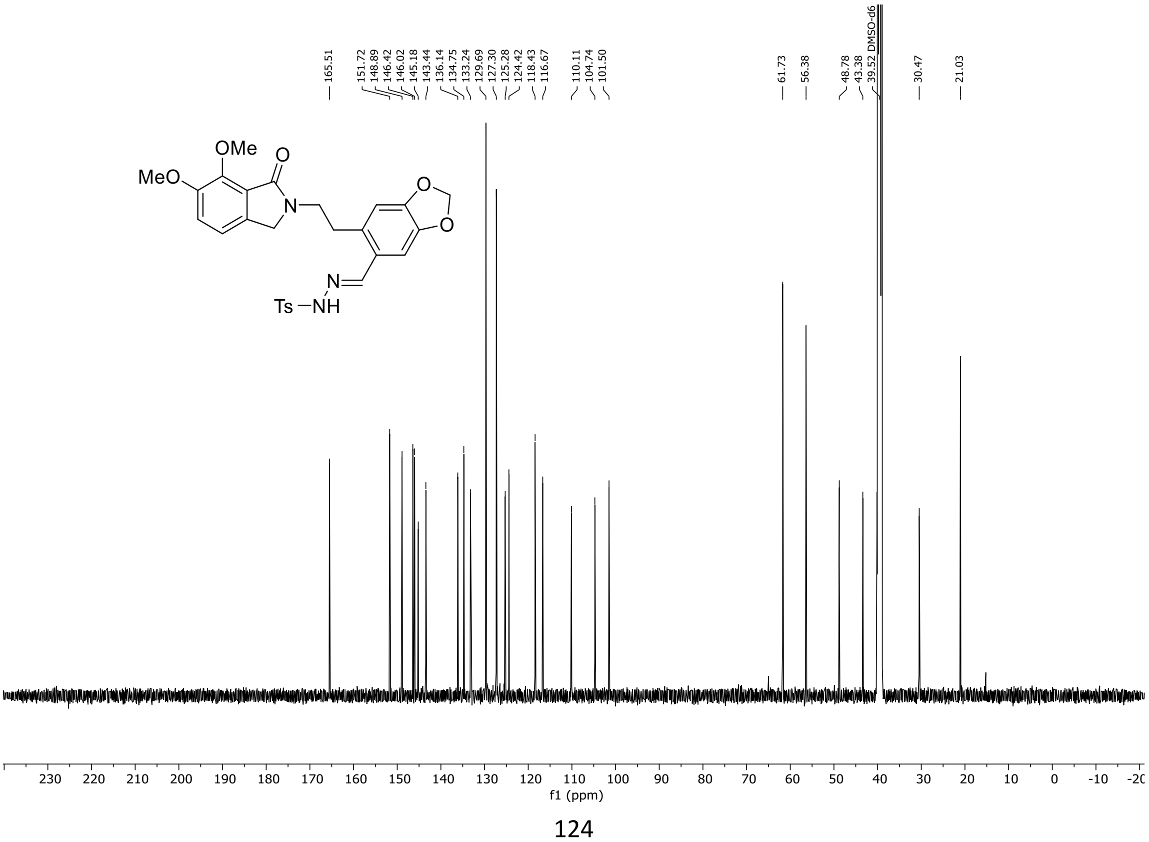

*8.2.21. N'-(3-(1-Oxoisoindolin-2-yl)butylidene)-4-methylbenzenesulfonohydrazide (**2u**)*

^{1}H NMR (500 MHz, DMSO-d_6):

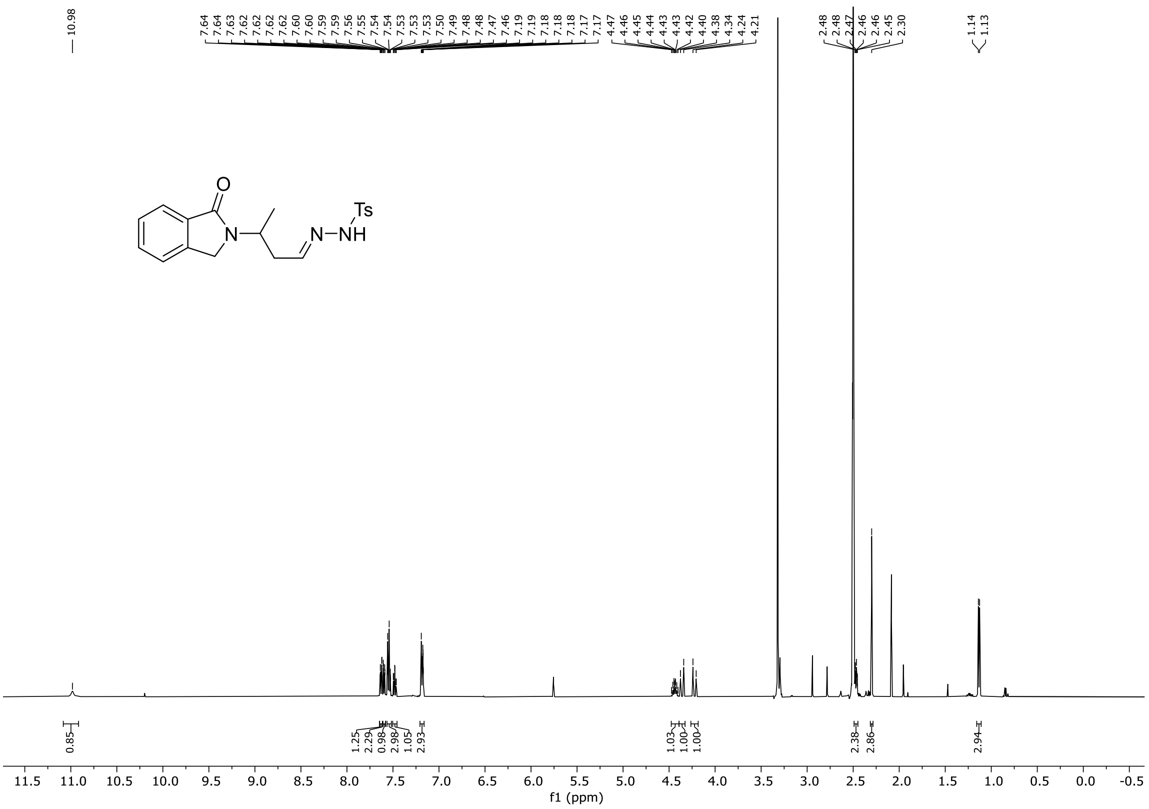

^{13}C NMR (126 MHz, DMSO-d_6):

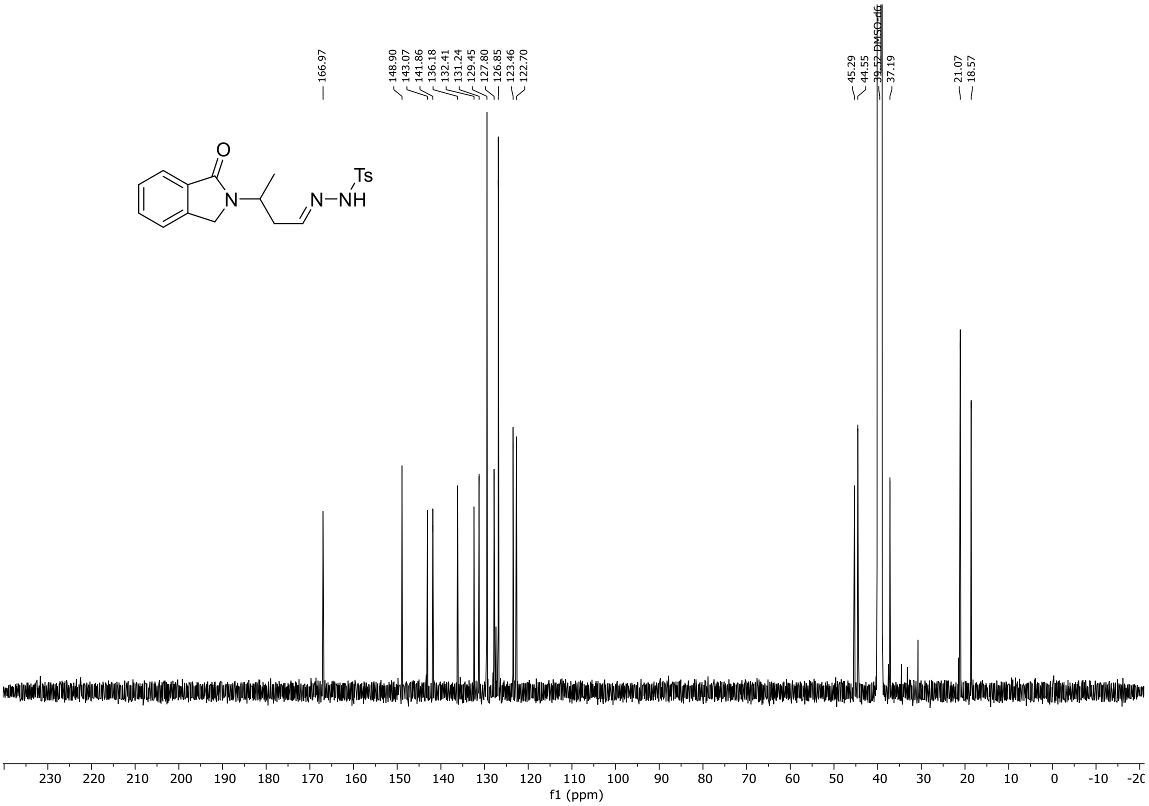

8.2.22. *N'-(3-((tert-Butyldimethylsilyl)oxy)-4-(1-oxoisoindolin-2-yl)butylidene)-4-methylbenzenesulfonohydrazide (**2v**)*

^{1}H NMR (500 MHz, DMSO-d_6):

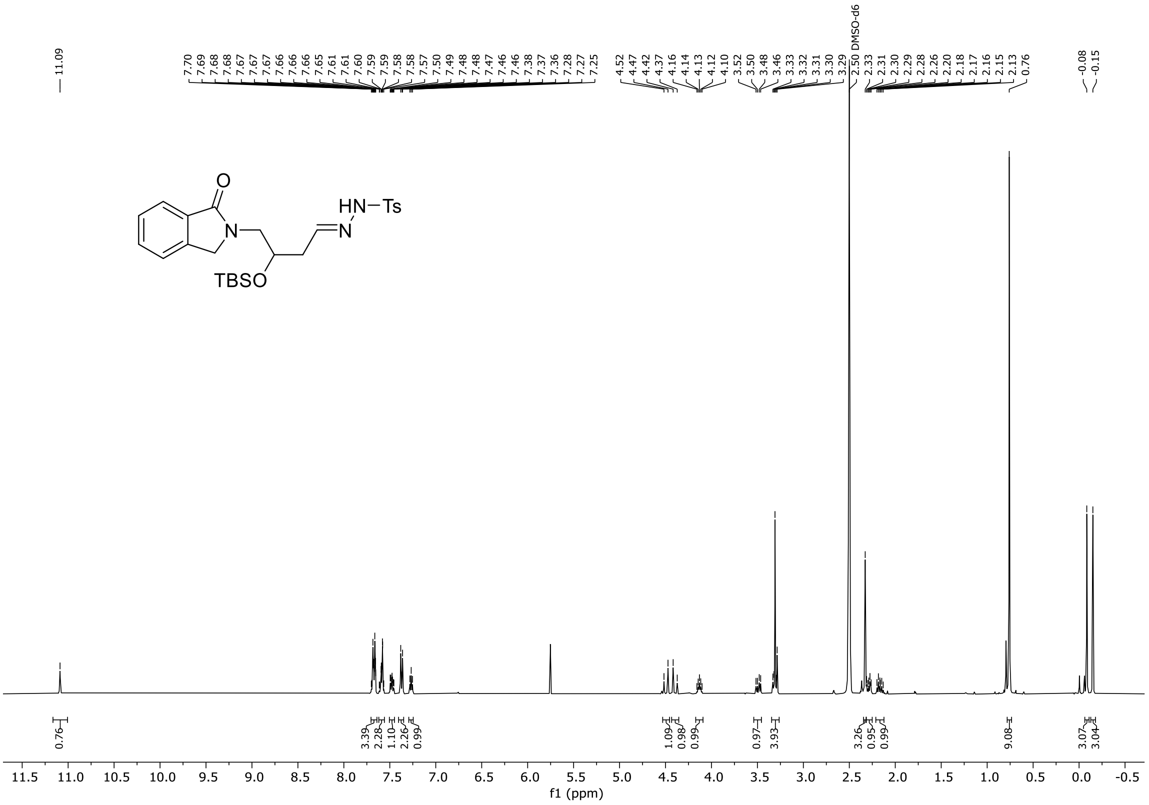

^{13}C NMR (126 MHz, DMSO-d_6):

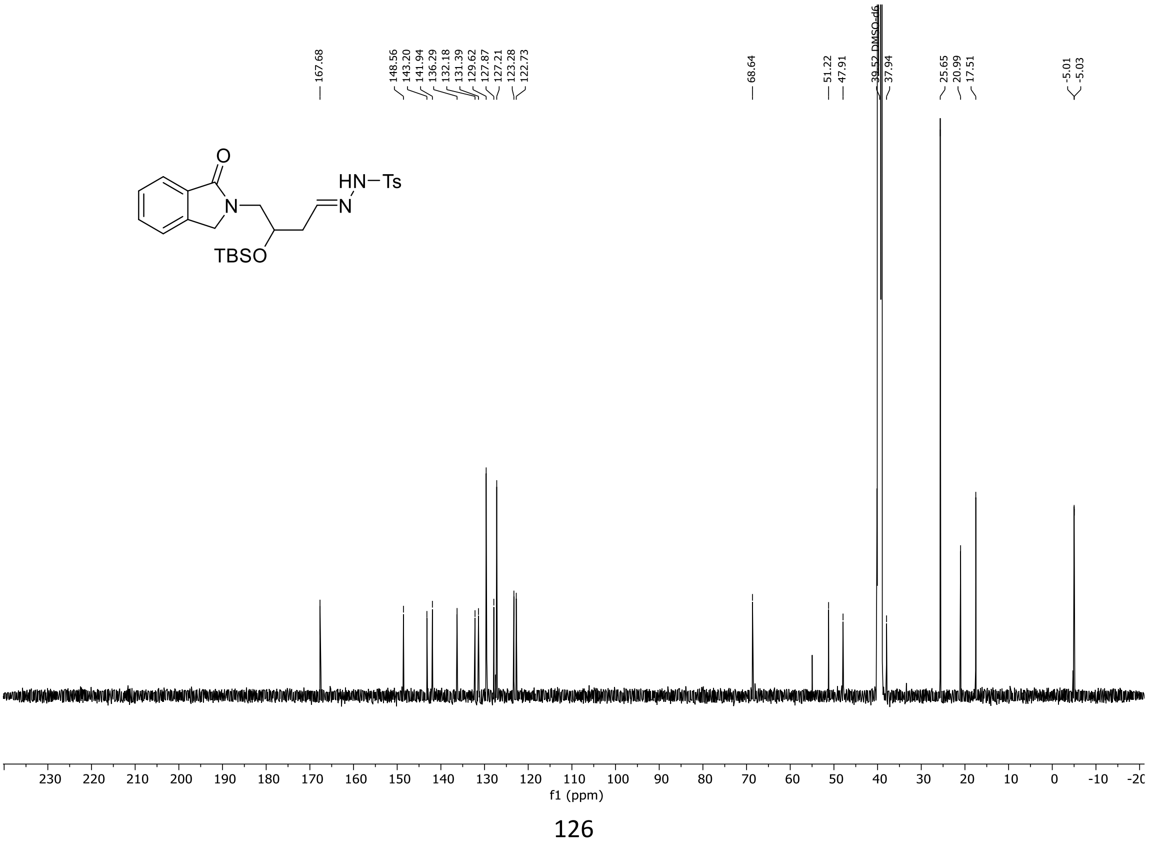

8.2.23. *N'-(3-((4-Methoxybenzyl)oxy)-4-(1-oxoisoindolin-2-yl)butylidene)-4-methylbenzenesulfonohydrazide* ***(2w)***

^{1}H NMR (500 MHz, DMSO-d_6):

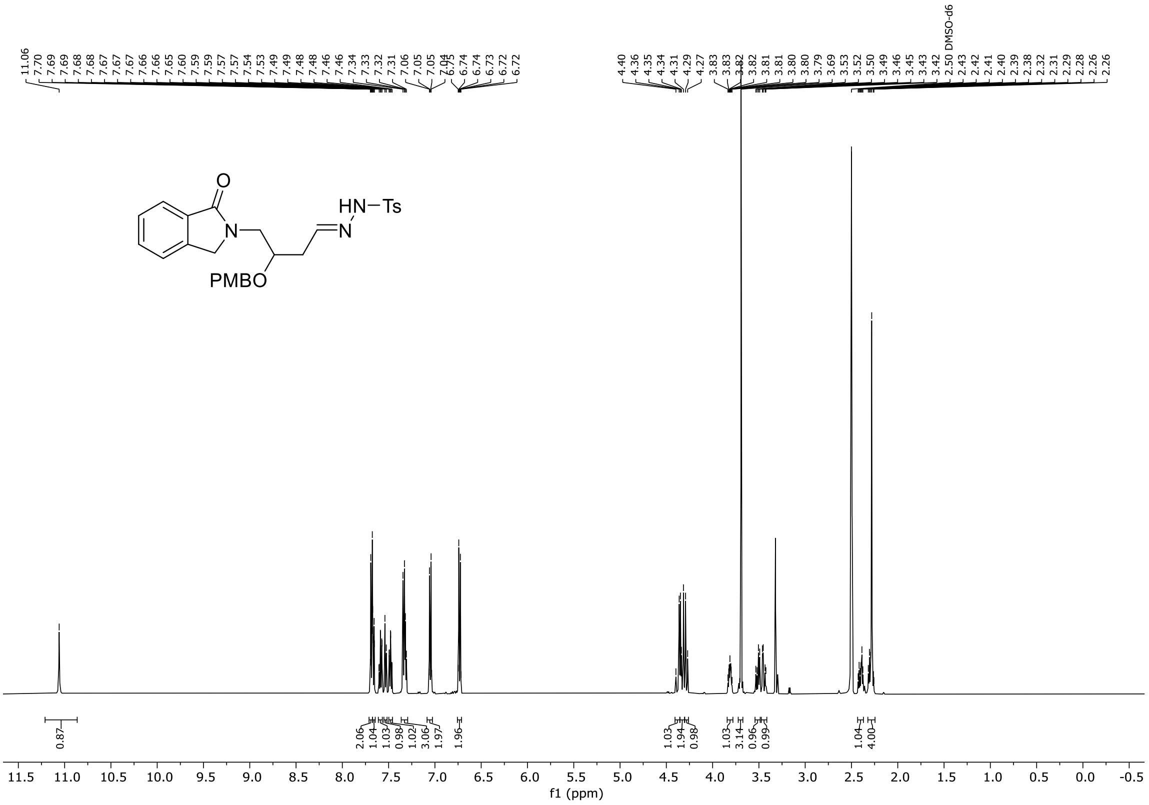

^{13}C NMR (126 MHz, DMSO-d_6):

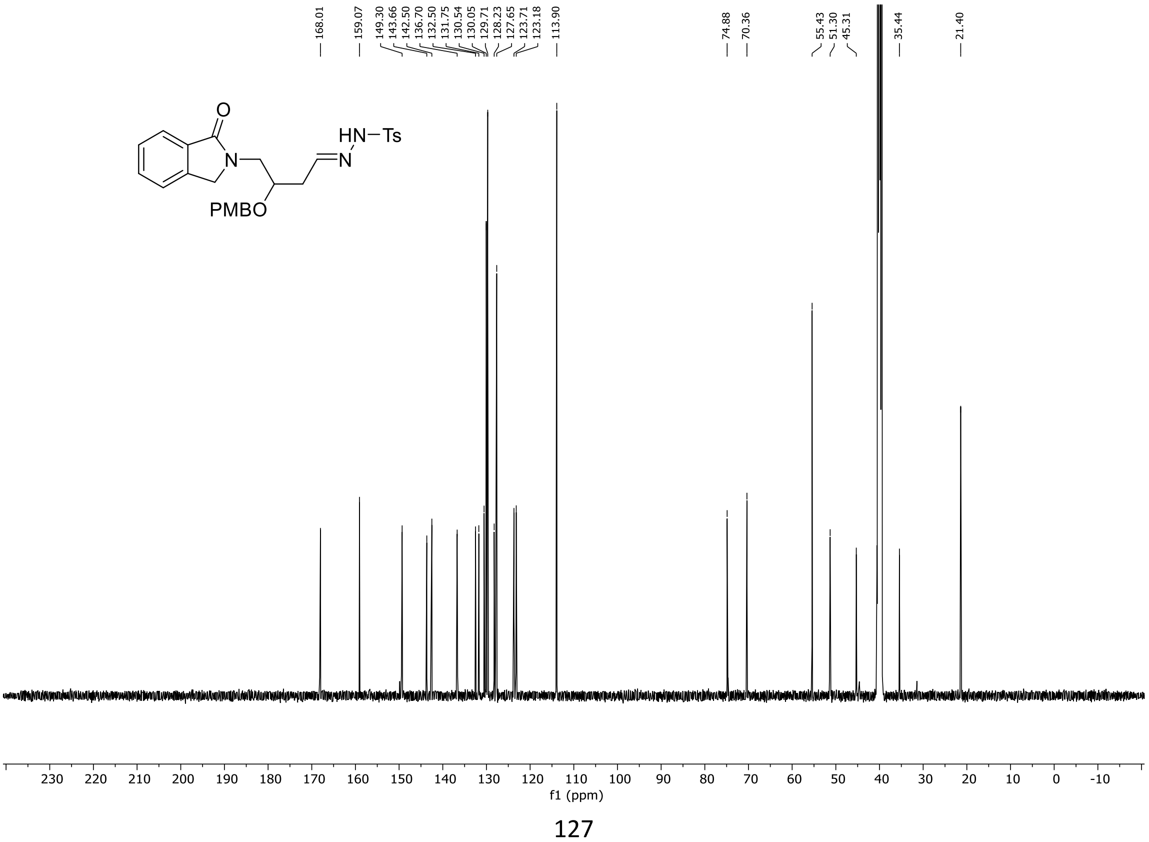

*8.2.24. N'-(2-Methyl-4-(1-oxoisoindolin-2-yl)butylidene)-4-methylbenzenesulfonohydrazide (**2x**)*

^{1}H NMR (500 MHz, DMSO-d_6):

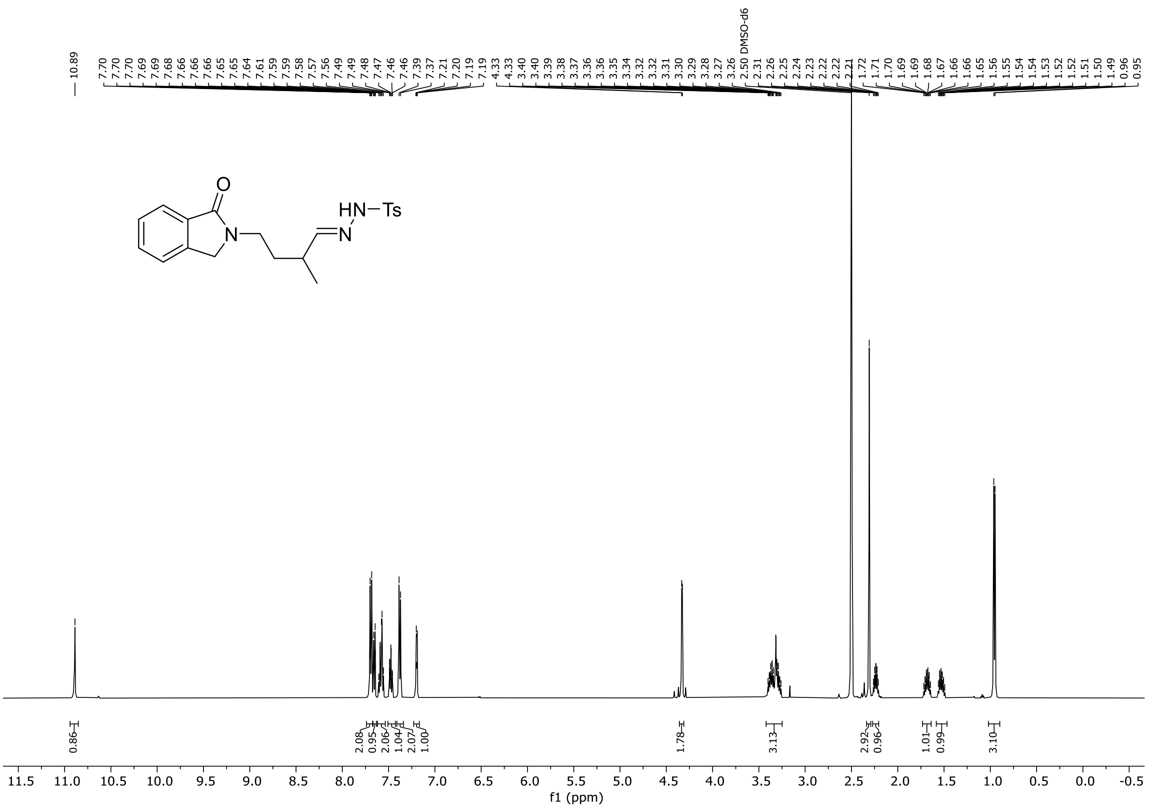

^{13}C NMR (126 MHz, DMSO-d_6):

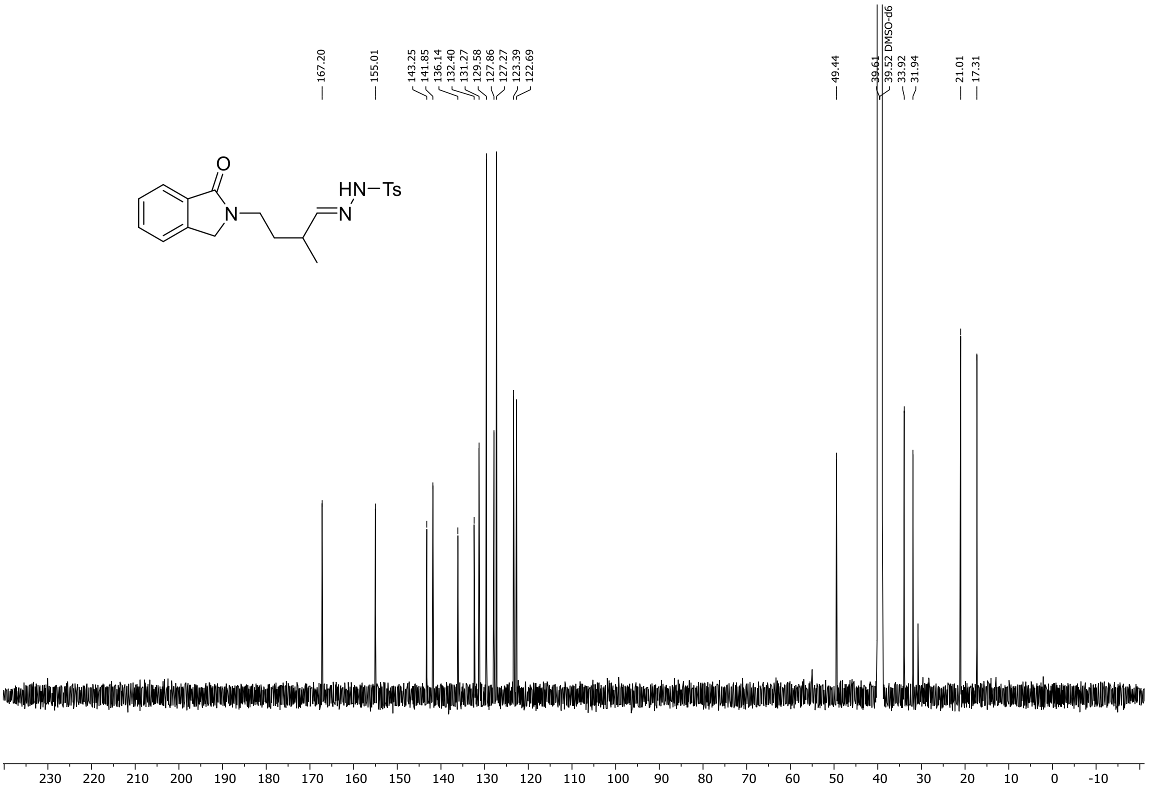

8.3. Individual Substrates

*8.3.1. Methyl 3-hydroxy-4-(1-oxoisoindolin-2-yl)butanoate (**SI-6**)*

^{1}H NMR (500 MHz, $CDCl_3$):

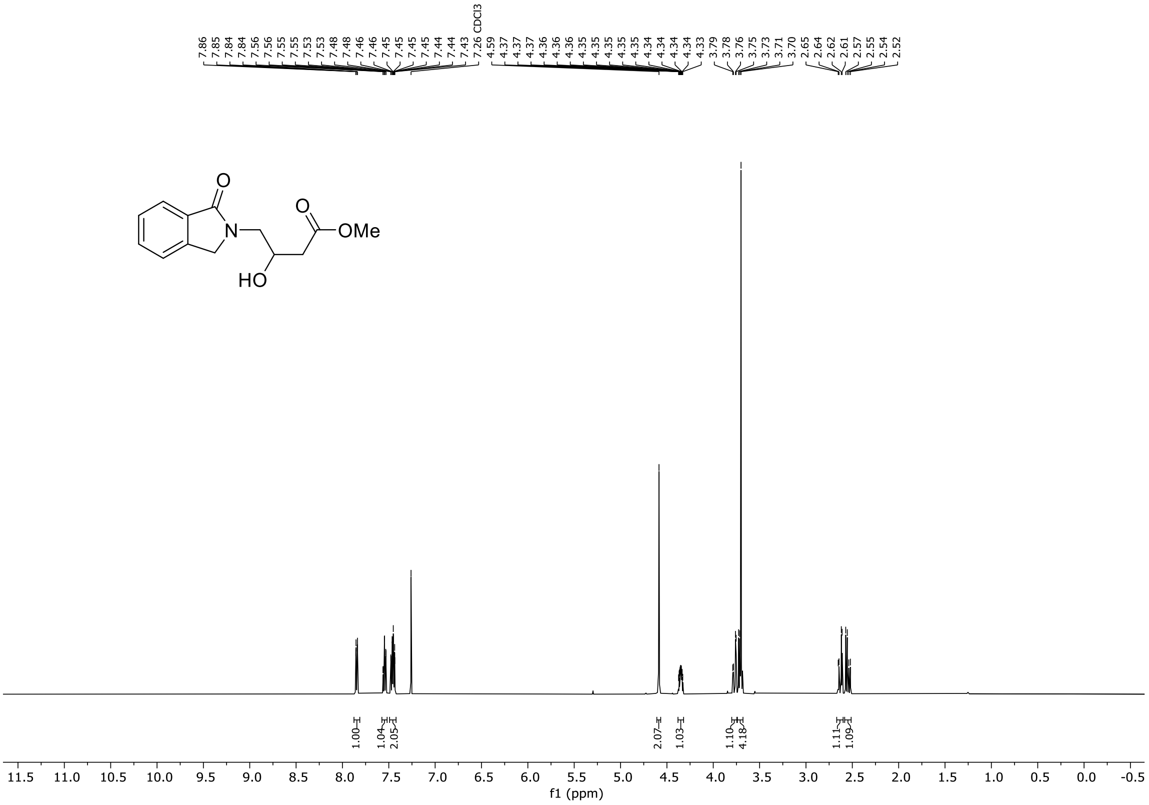

^{13}C NMR (126 MHz, $CDCl_3$):

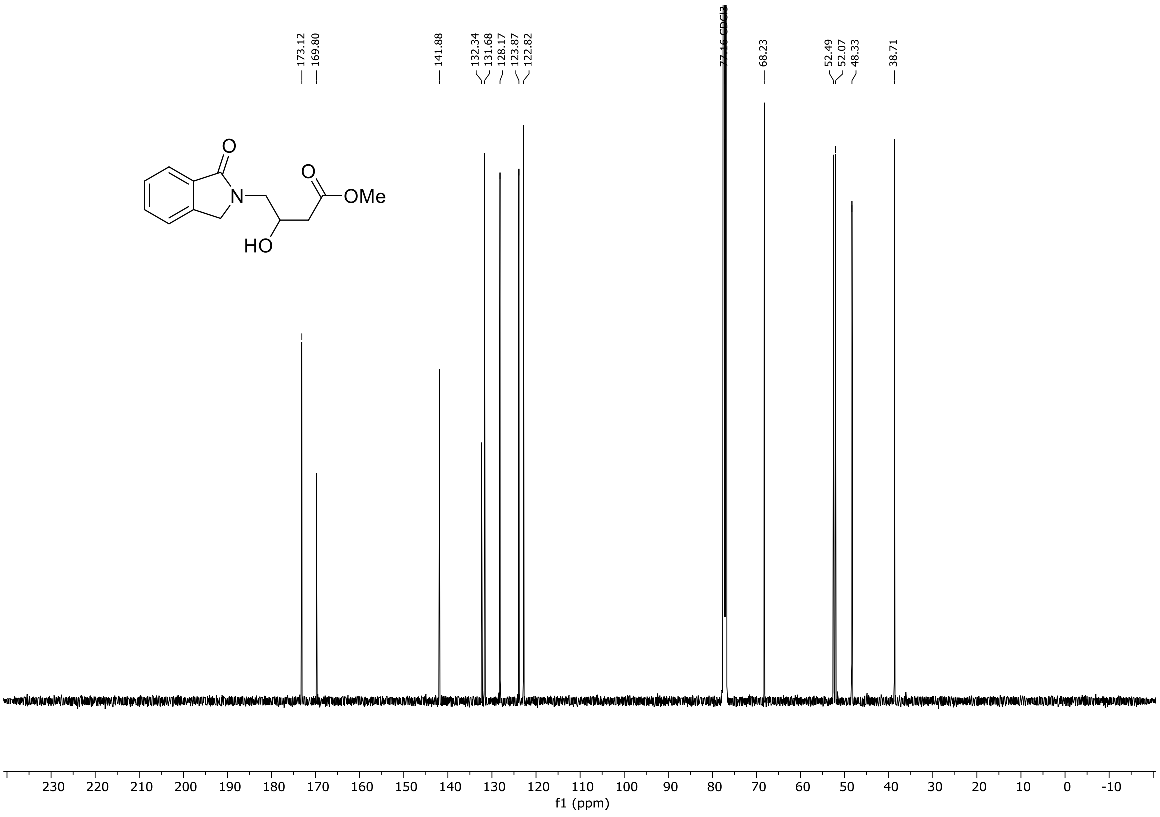

*8.3.2. Methyl 3-((tert-butyldimethylsilyl)oxy)-4-(1-oxoisoindolin-2-yl)butanoate (**SI-7a**)*

^{1}H NMR (500 MHz, $CDCl_3$):

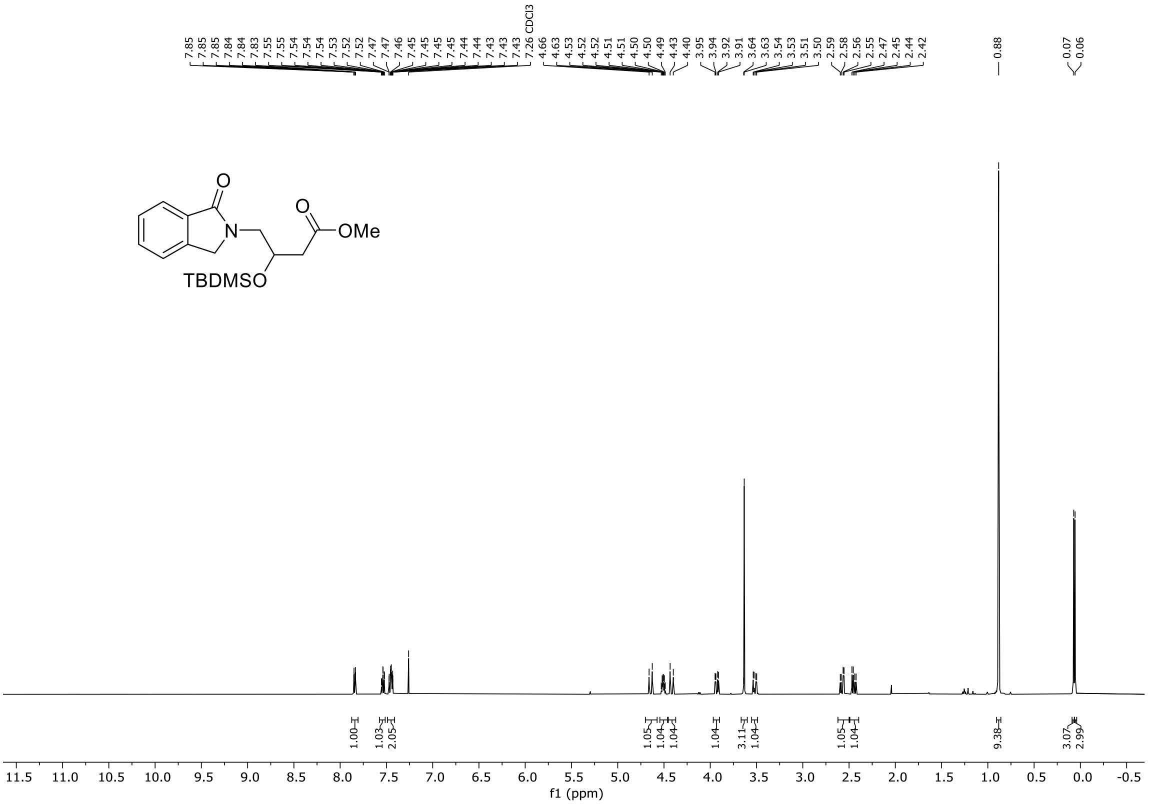

^{13}C NMR (126 MHz, $CDCl_3$):

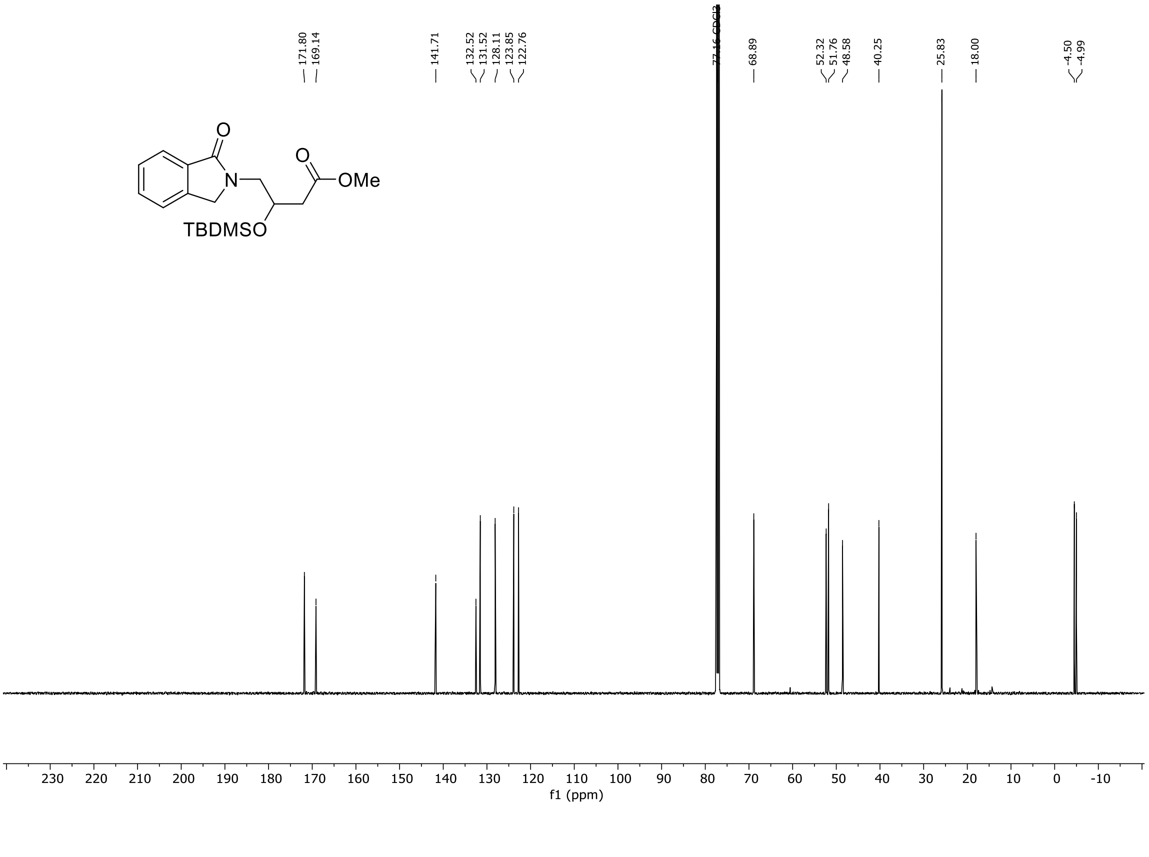

*8.3.3. Methyl 3-((4-methoxybenzyl)oxy)-4-(1-oxoisoindolin-2-yl)butanoate (**SI-7b**)*

^{1}H NMR (500 MHz, $CDCl_3$):

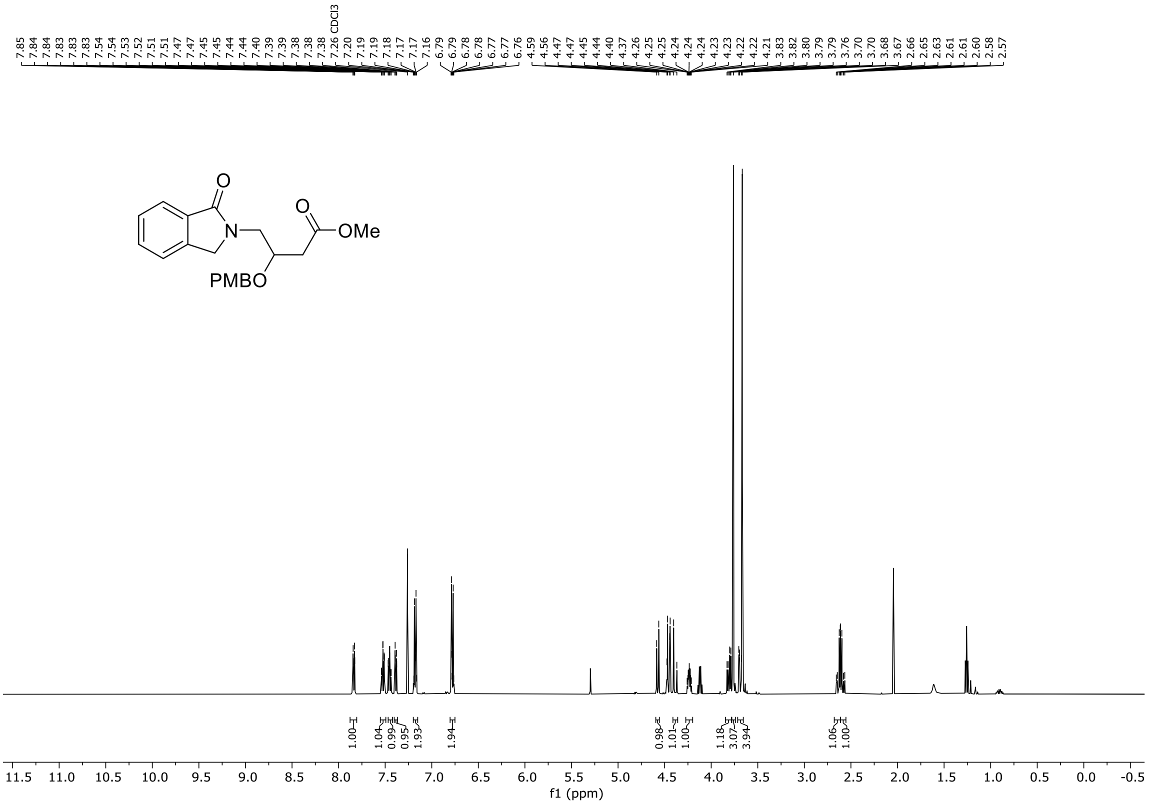

^{13}C NMR (126 MHz, $CDCl_3$):

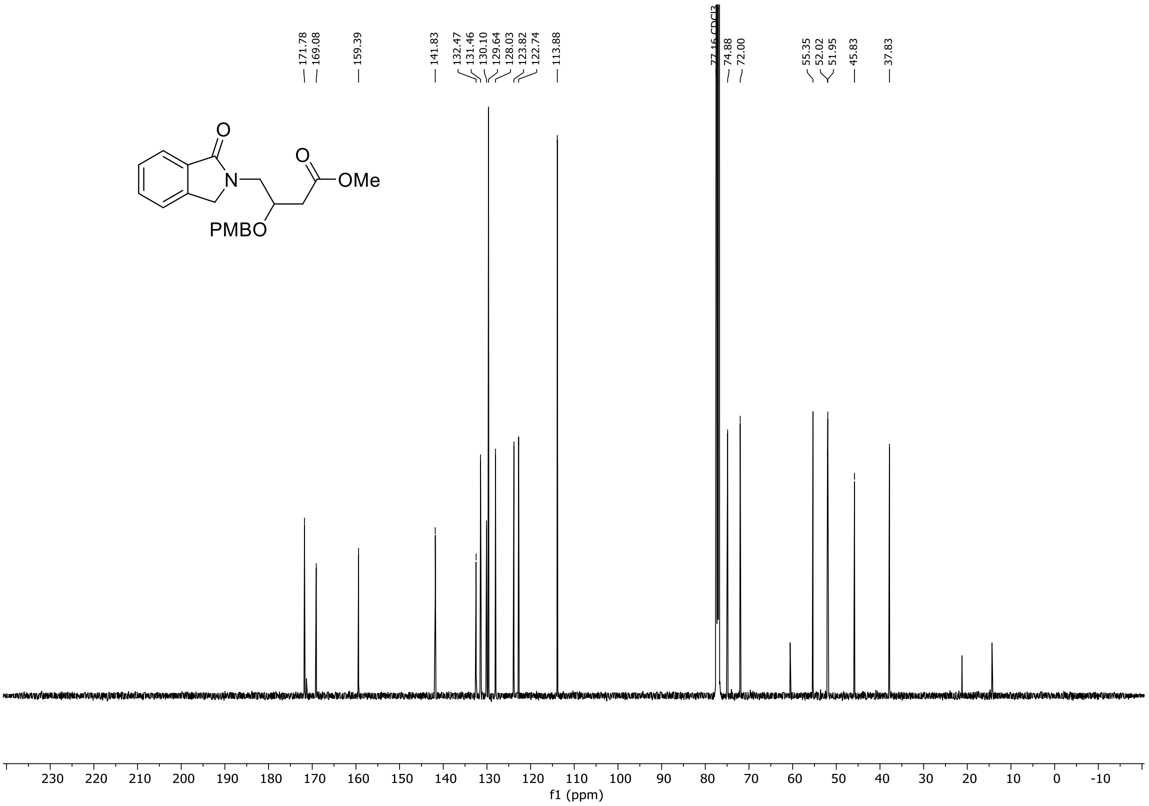

*8.3.4. Dimethyl 2-(chloromethyl)terephthalate (**SI-9**)*

^{1}H NMR (500 MHz, $CDCl_3$):

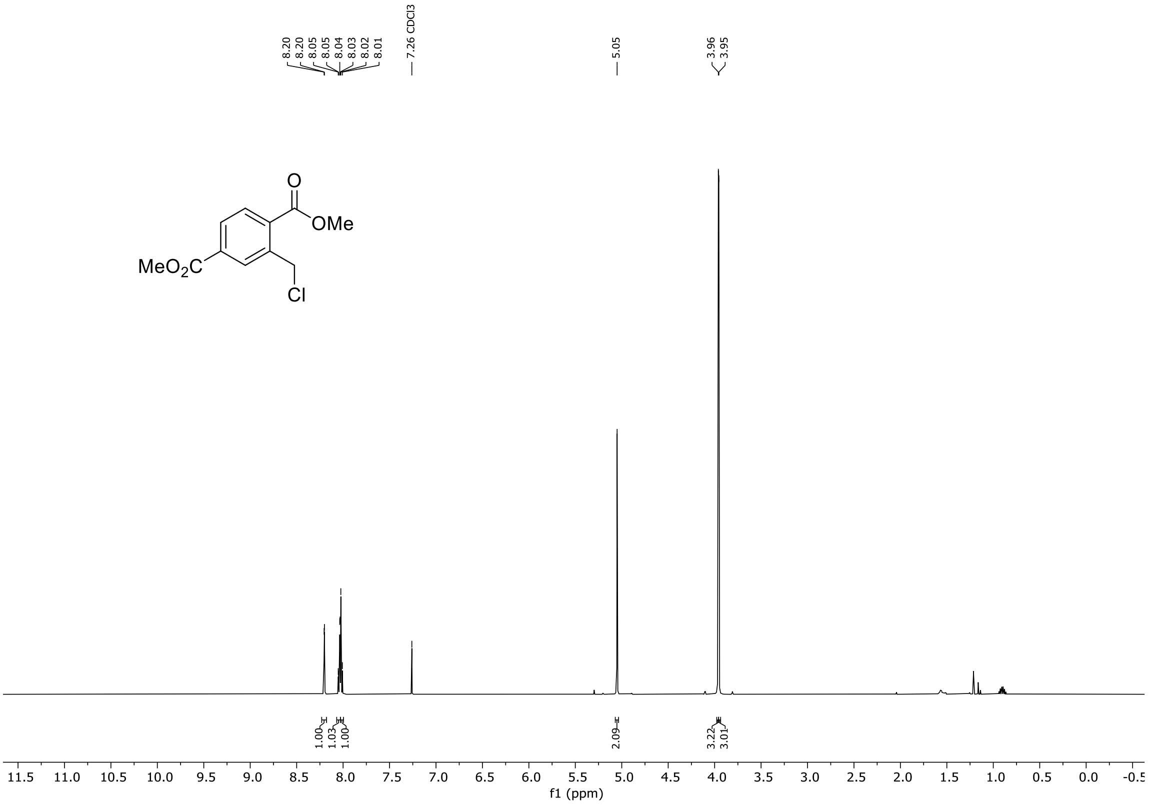

^{13}C NMR (126 MHz, $CDCl_3$):

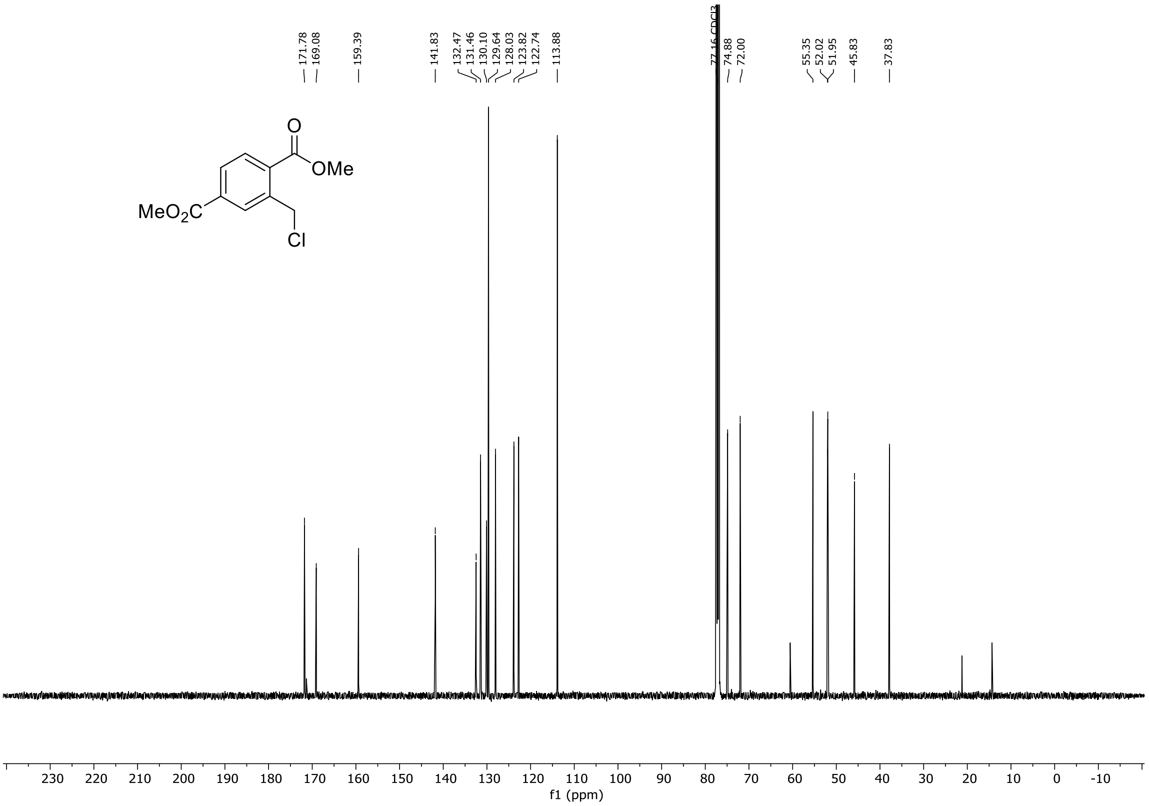

*8.3.5. 2-(2-(2-Bromoethyl)phenyl)-1,3-dioxolane (**SI-11**)*

^{1}H NMR (500 MHz, $CDCl_3$):

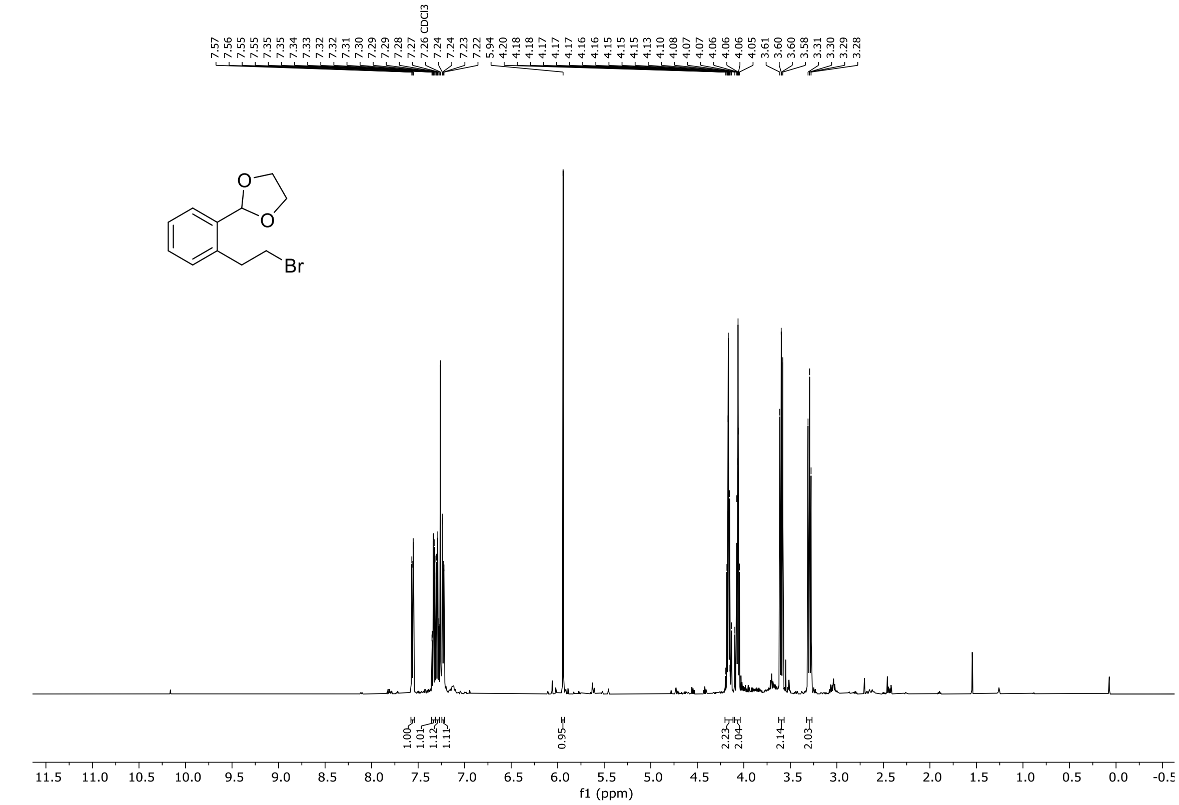

^{13}C NMR (126 MHz, $CDCl_3$):

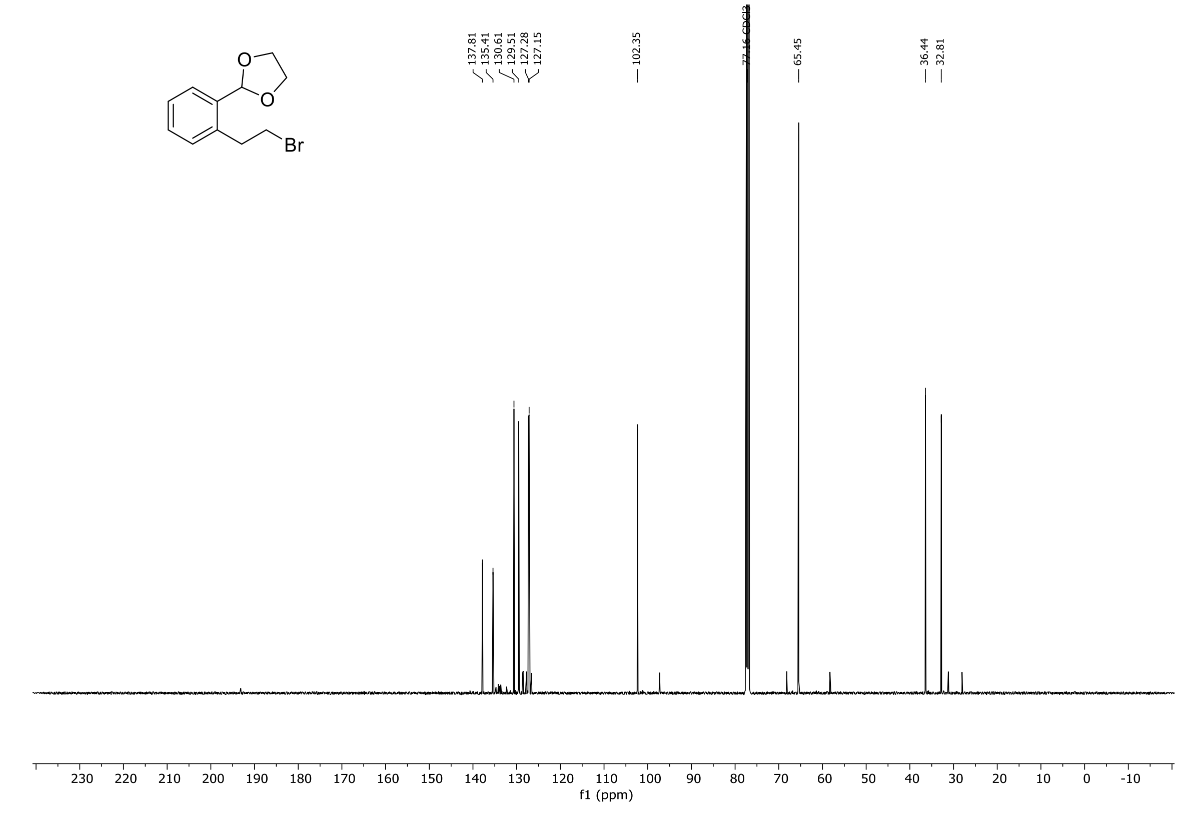

*8.3.6. 2-(2-(1,3-Dioxolan-2-yl)phenethyl)isoindolin-1-one (**SI-2s**)*

^{1}H NMR (500 MHz, DMSO-d_6):

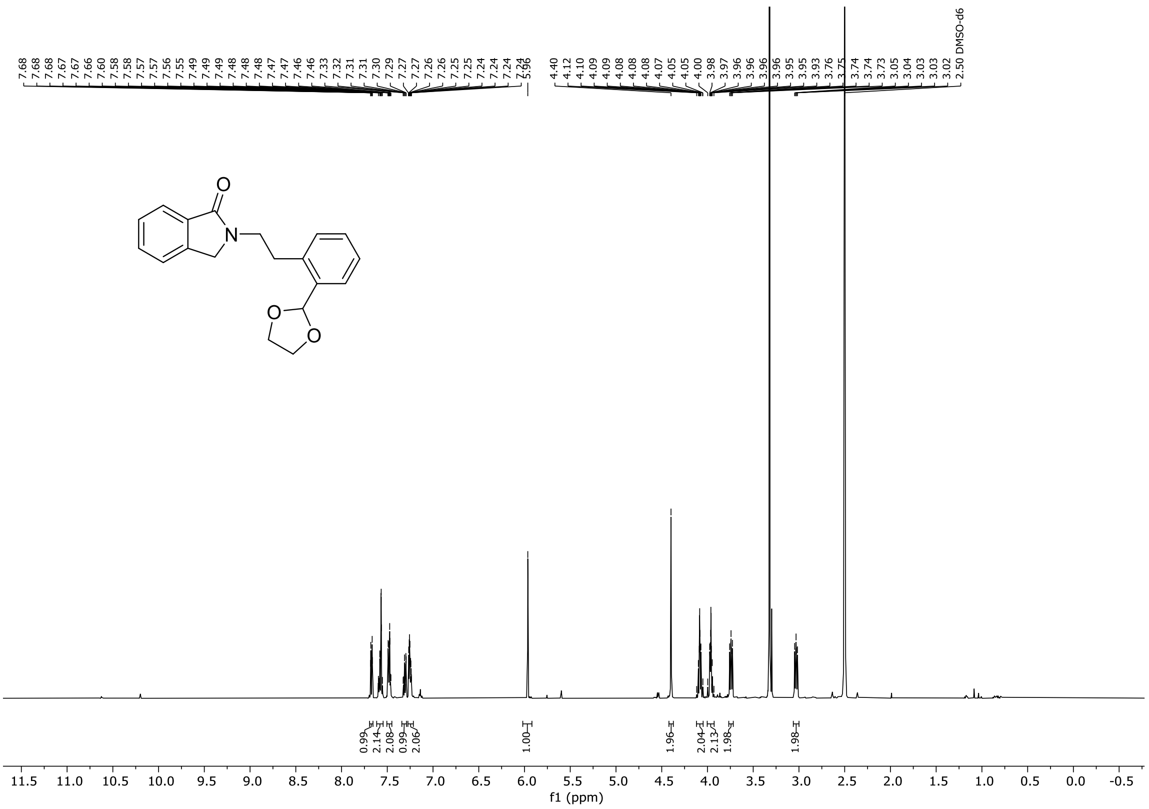

^{13}C NMR (126 MHz, DMSO-d_6):

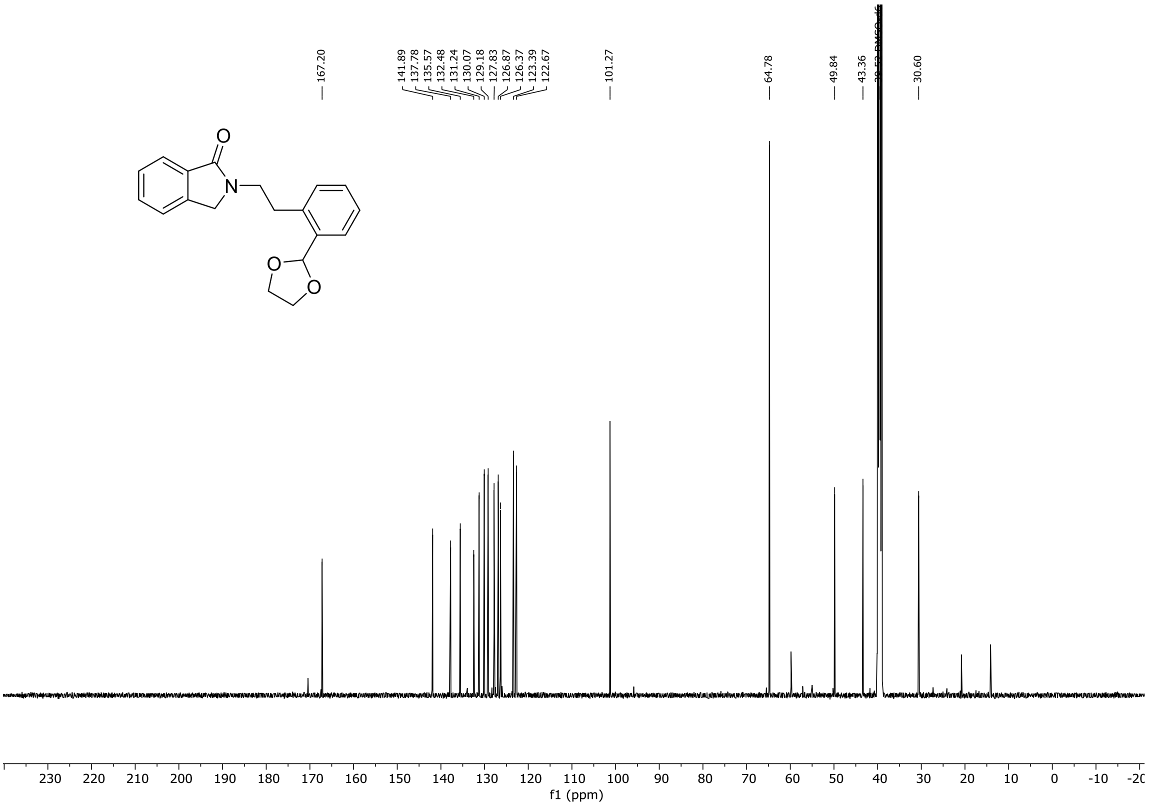

*8.3.7. 2-(2-(1-Oxoisoindolin-2-yl)ethyl)benzaldehyde (**SI-4s**)*

^{1}H NMR (500 MHz, DMSO-d_6):

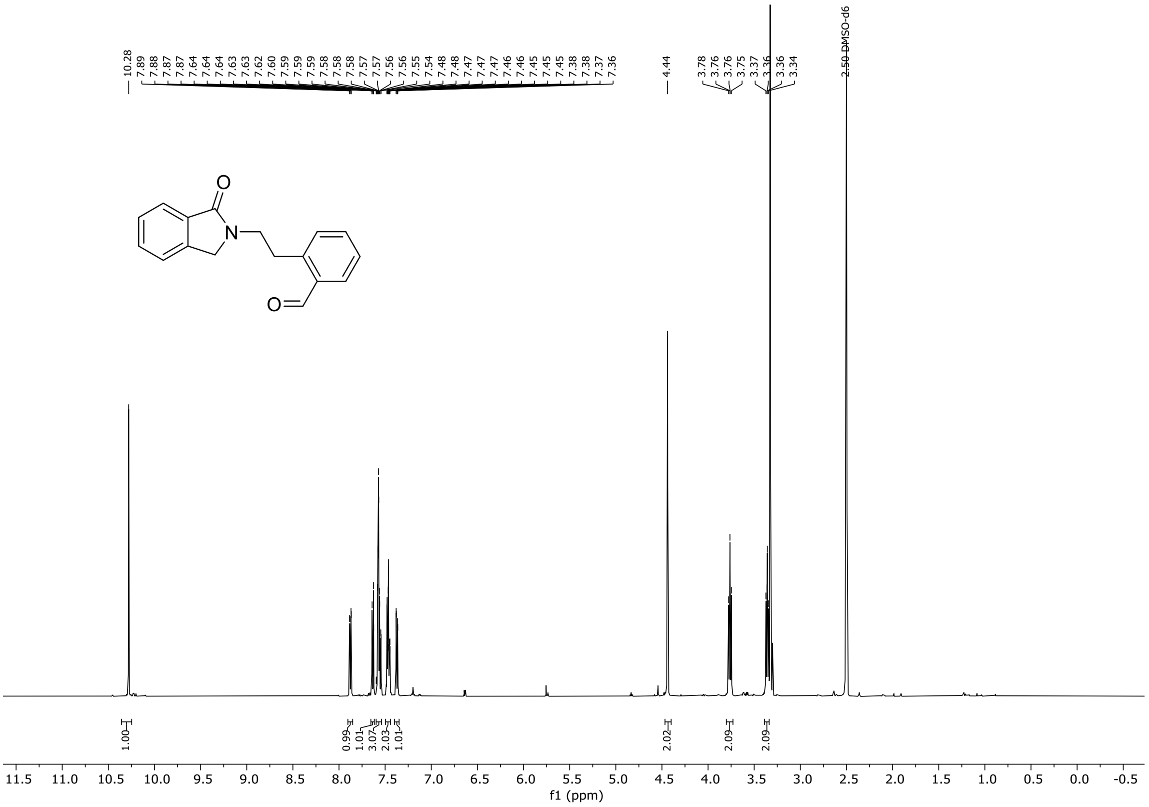

^{13}C NMR (126 MHz, DMSO-d_6):

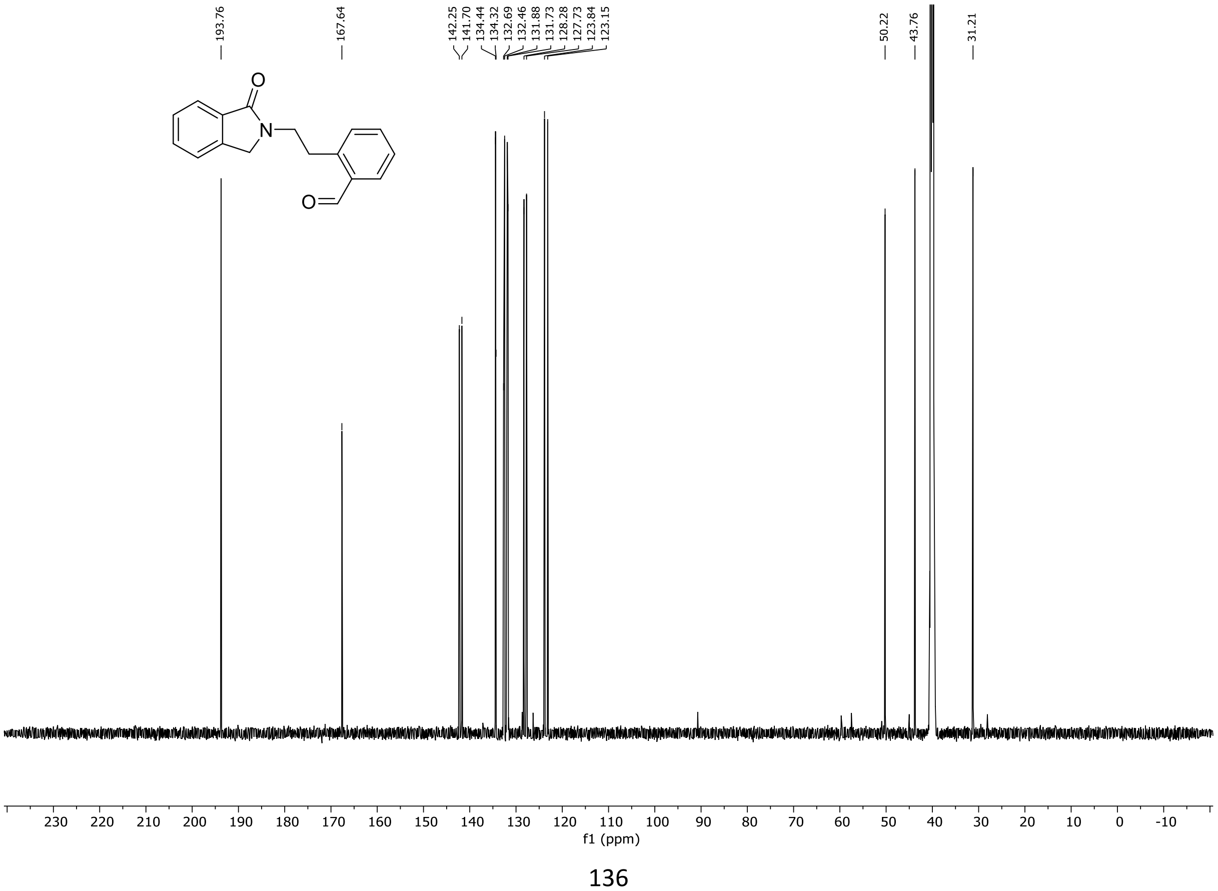

*8.3.8. 2-(2-(Bromomethyl)benzyl)isoindolin-1-one (**SI-13**)*

^{1}H NMR (500 MHz, $CDCl_3$):

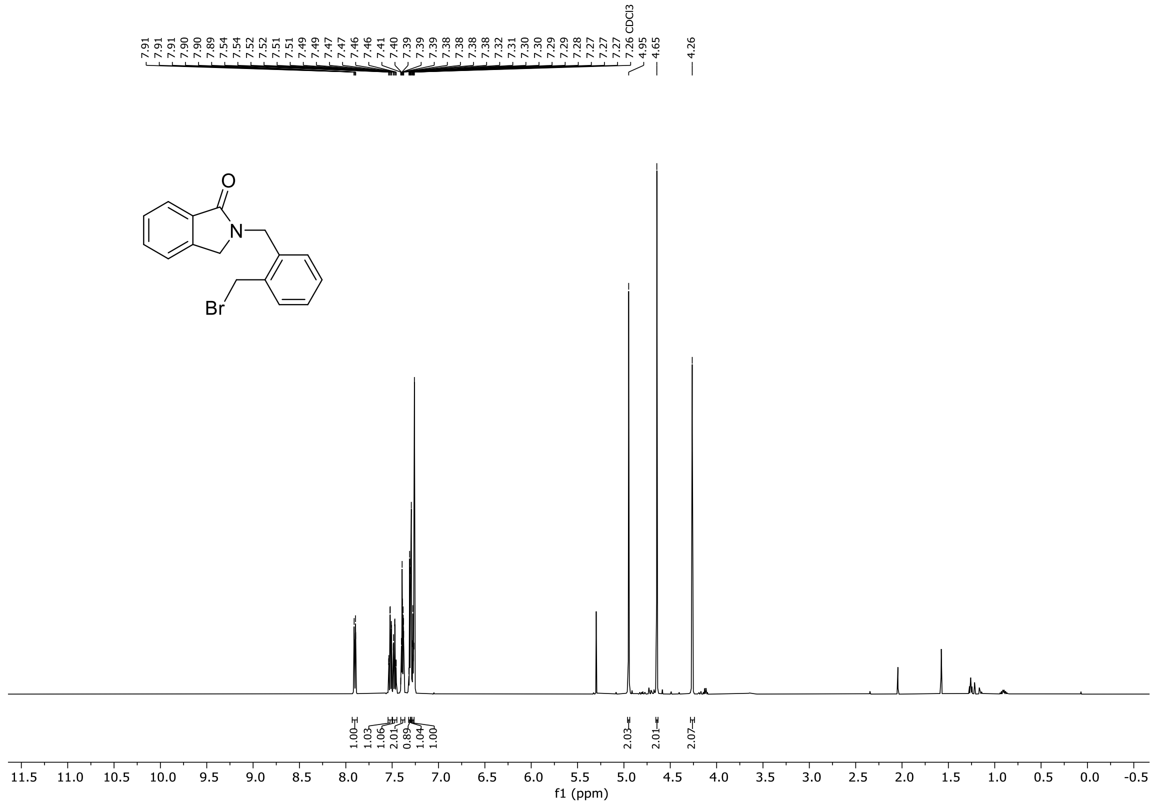

^{13}C NMR (126 MHz, $CDCl_3$):

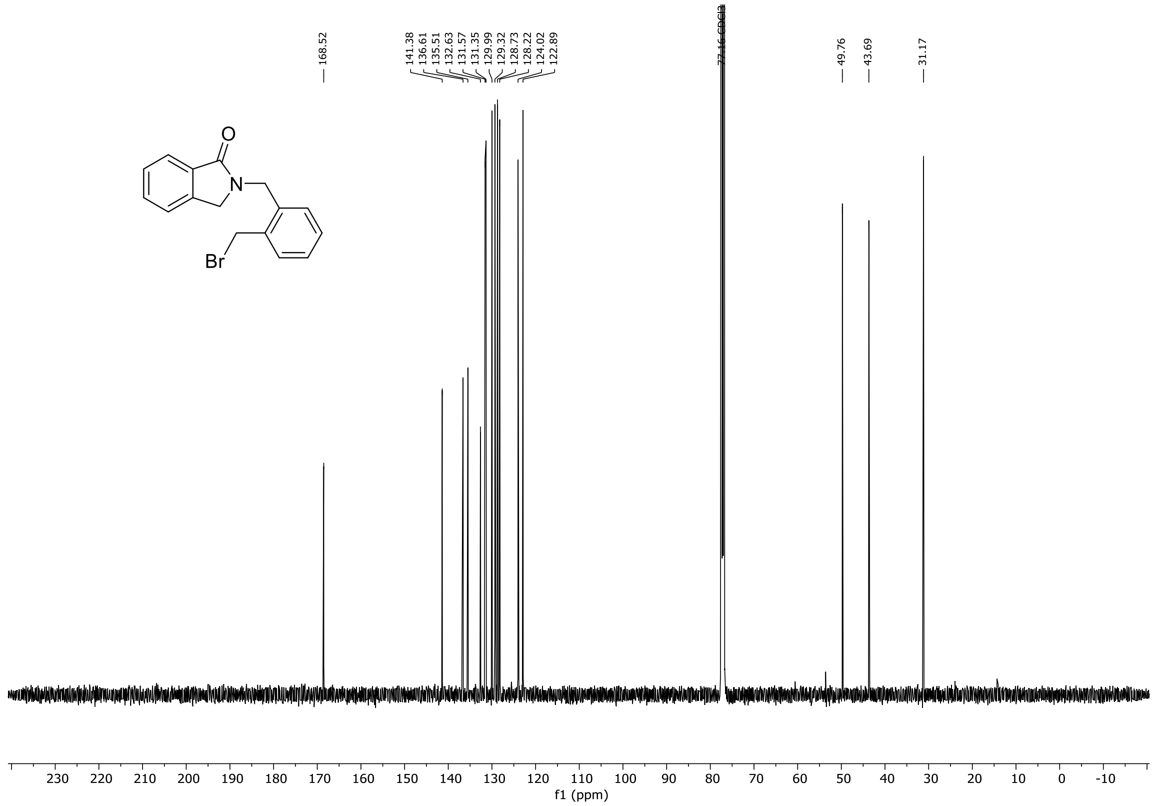

*8.3.9. 2-((1-Oxoisoindolin-2-yl)methyl)benzaldehyde (**SI-4q**)*

^{1}H NMR (500 MHz, $CDCl_3$):

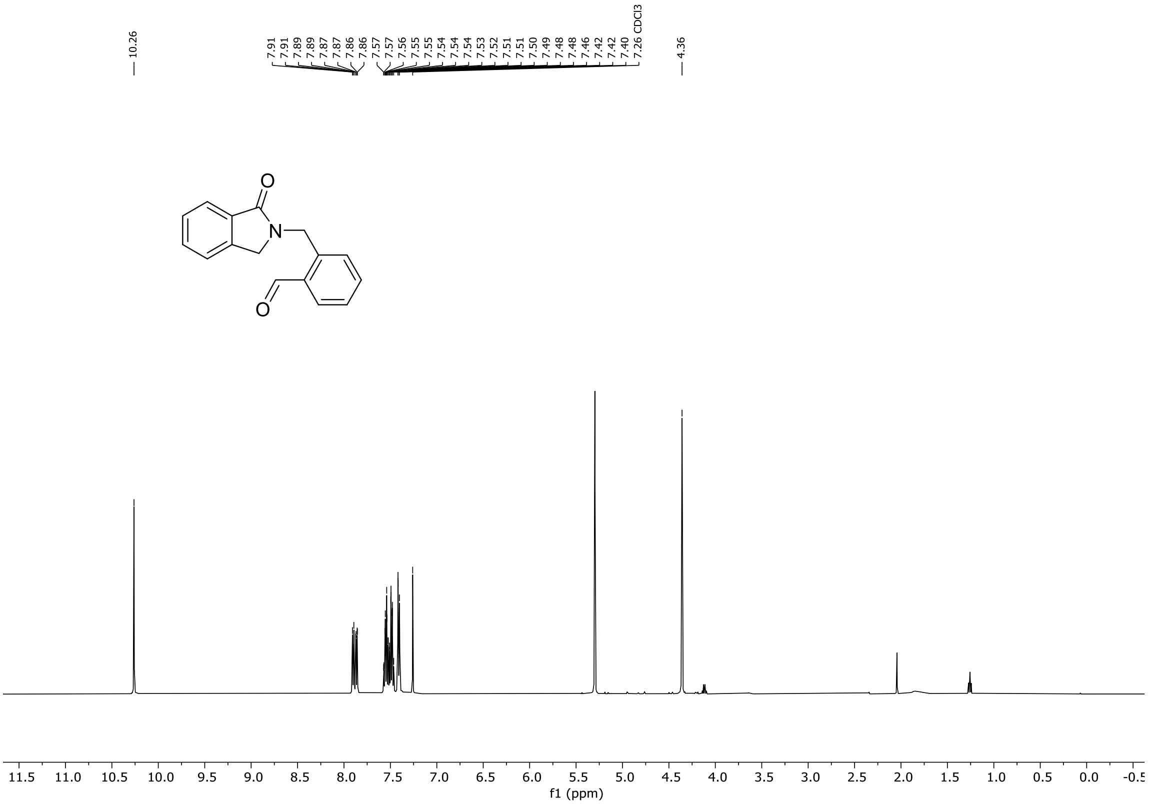

^{13}C NMR (126 MHz, $CDCl_3$):

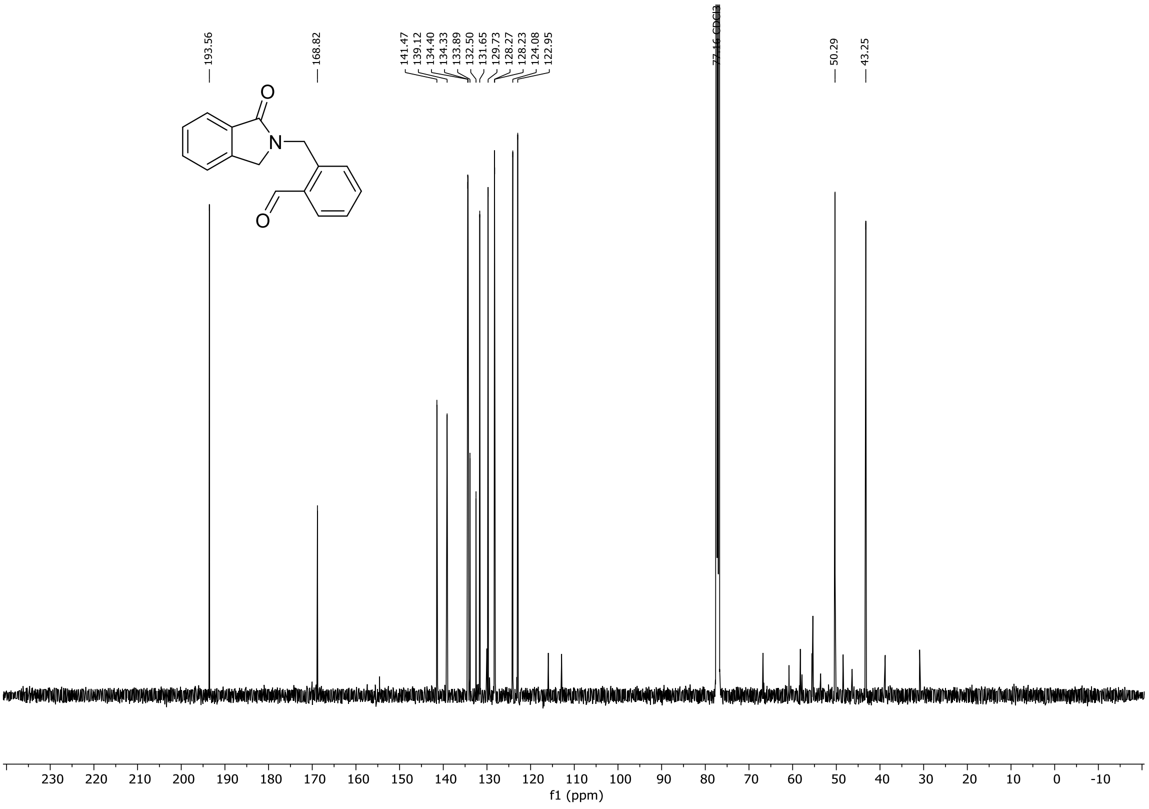

*8.3.10. 2-(3,3-Diethoxypropyl)-3-methylisoindolin-1-one (**SI-3o**)*

^{1}H NMR (500 MHz, $CDCl_3$):

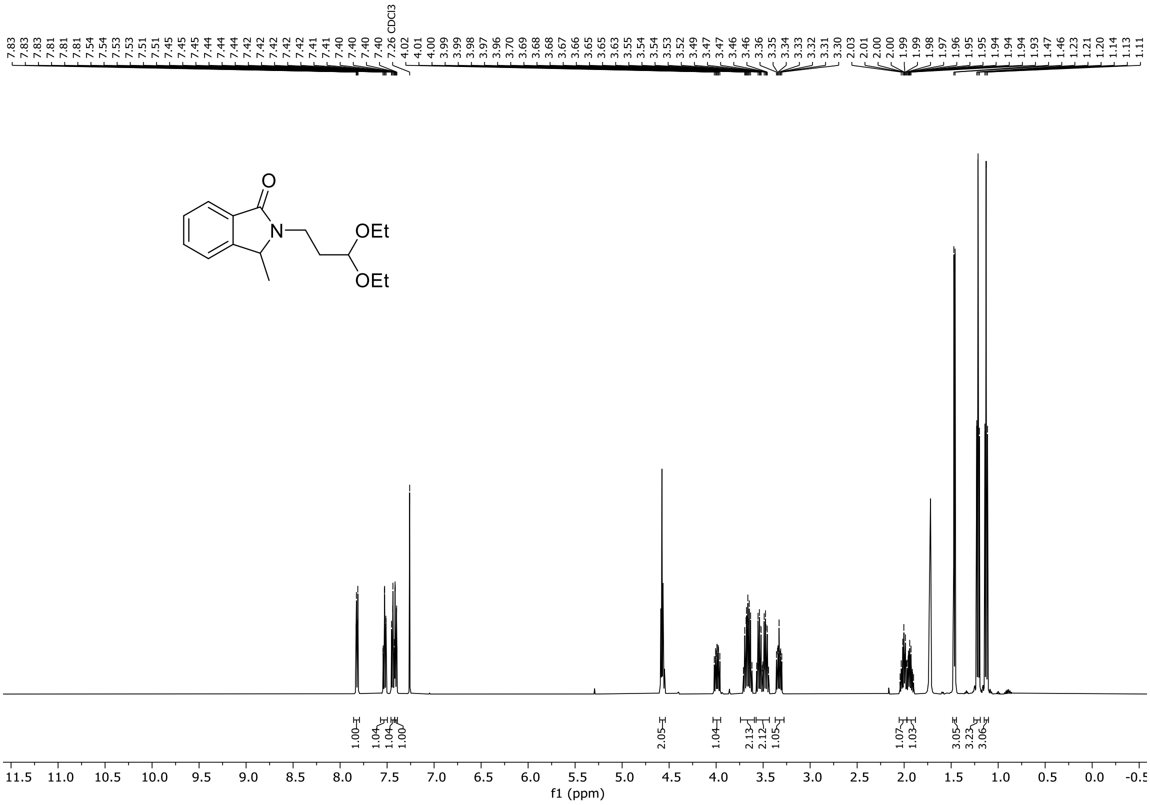

^{13}C NMR (126 MHz, $CDCl_3$):

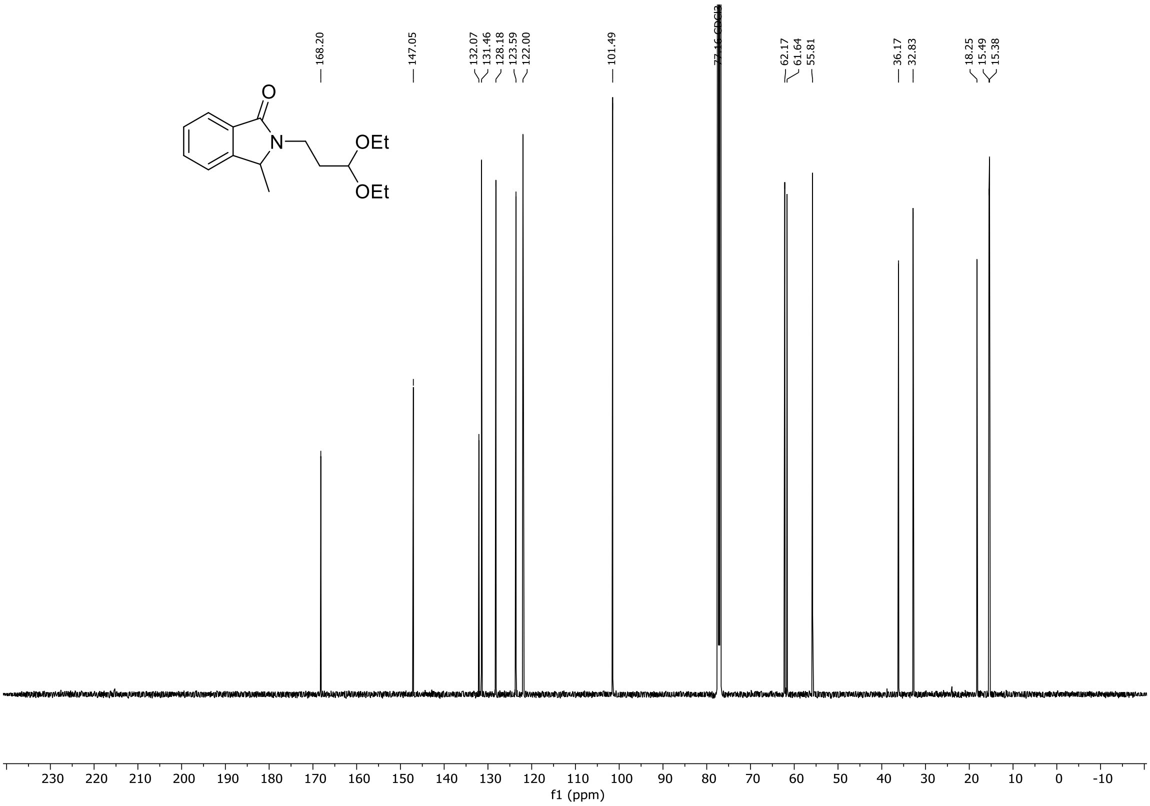

8.4. Catalysis Products

*8.4.1. 1,2,3,9b-Tetrahydro-5H-pyrrolo[2,1-a]isoindol-5-one (**1a**)*

^{1}H NMR (500 MHz, $CDCl_3$):

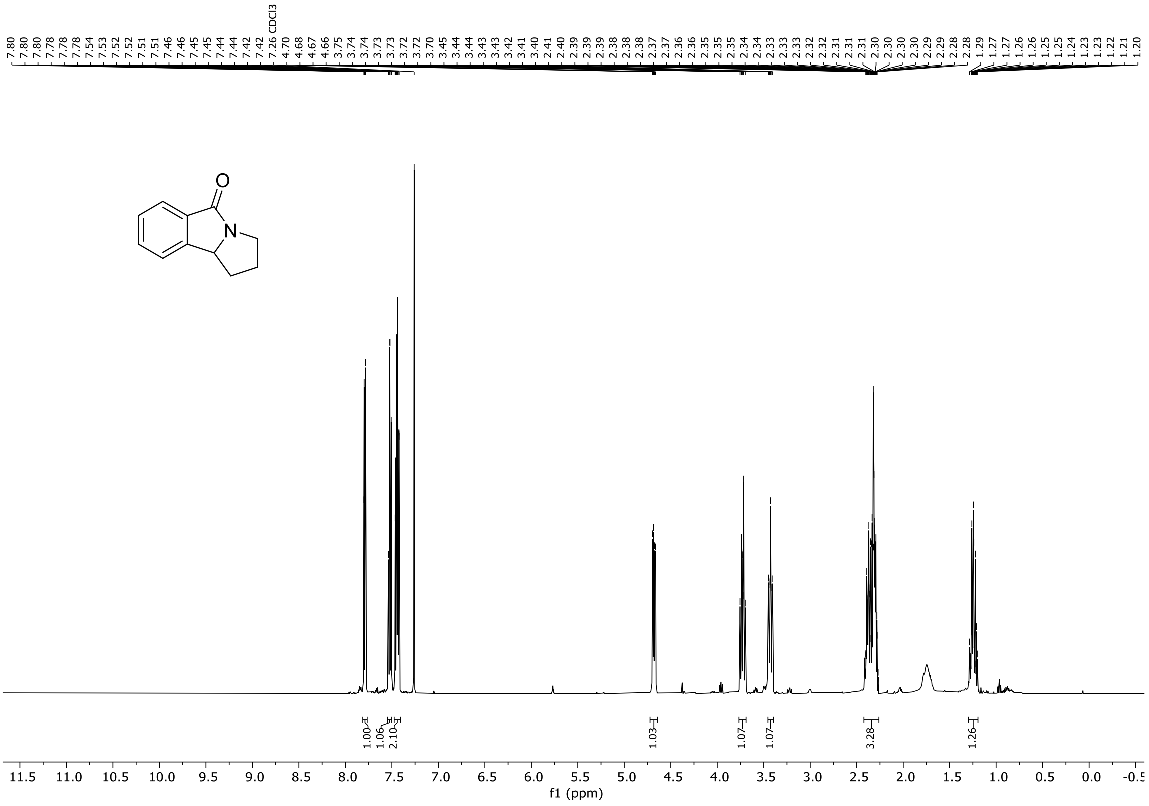

^{13}C NMR (126 MHz, $CDCl_3$):

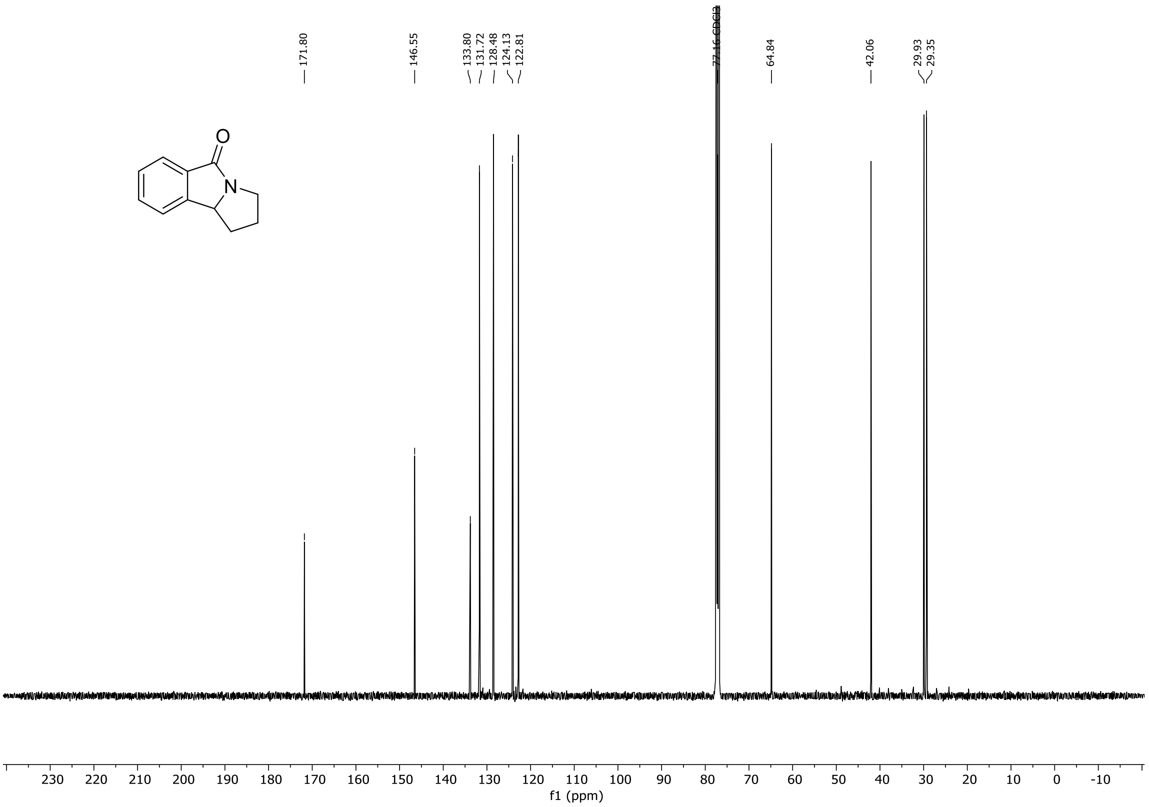

*8.4.2. 2,2-Dimethyl-1,2,3,9b-tetrahydro-5H-pyrrolo[2,1-a]isoindol-5-one (**1b**)*

^{1}H NMR (500 MHz, $CDCl_3$):

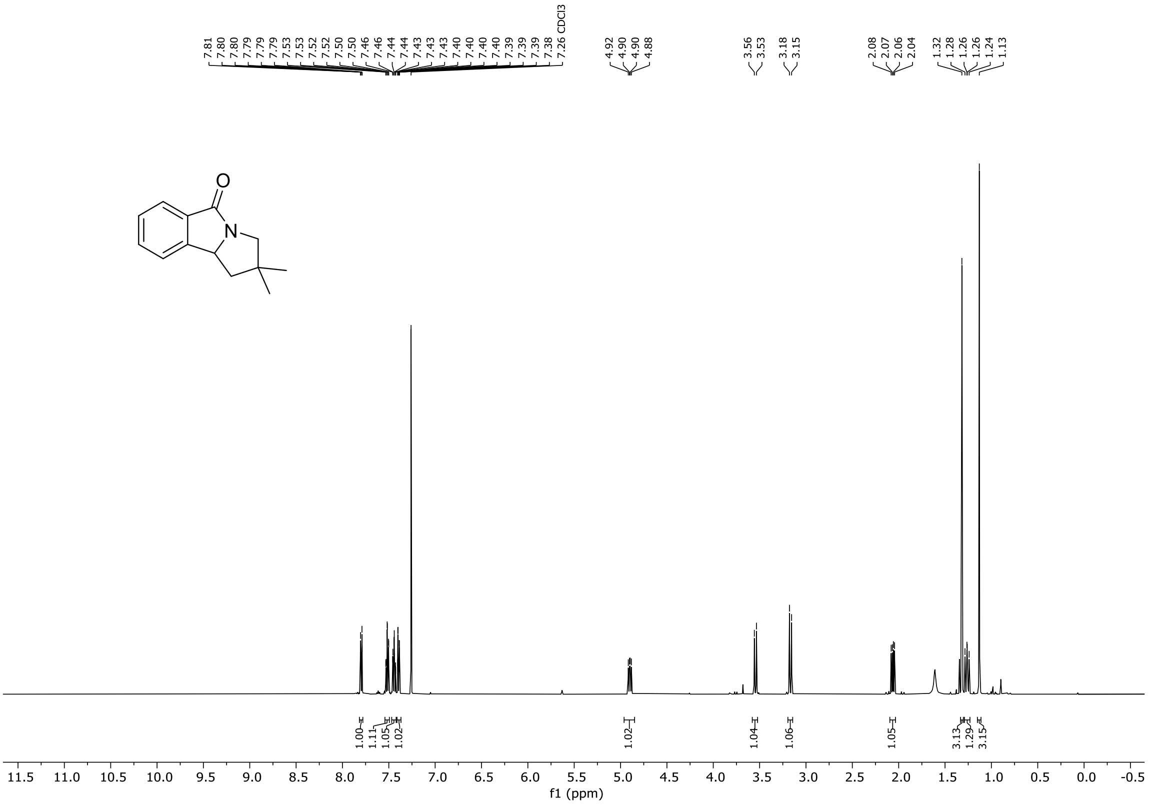

^{13}C NMR (126 MHz, $CDCl_3$):

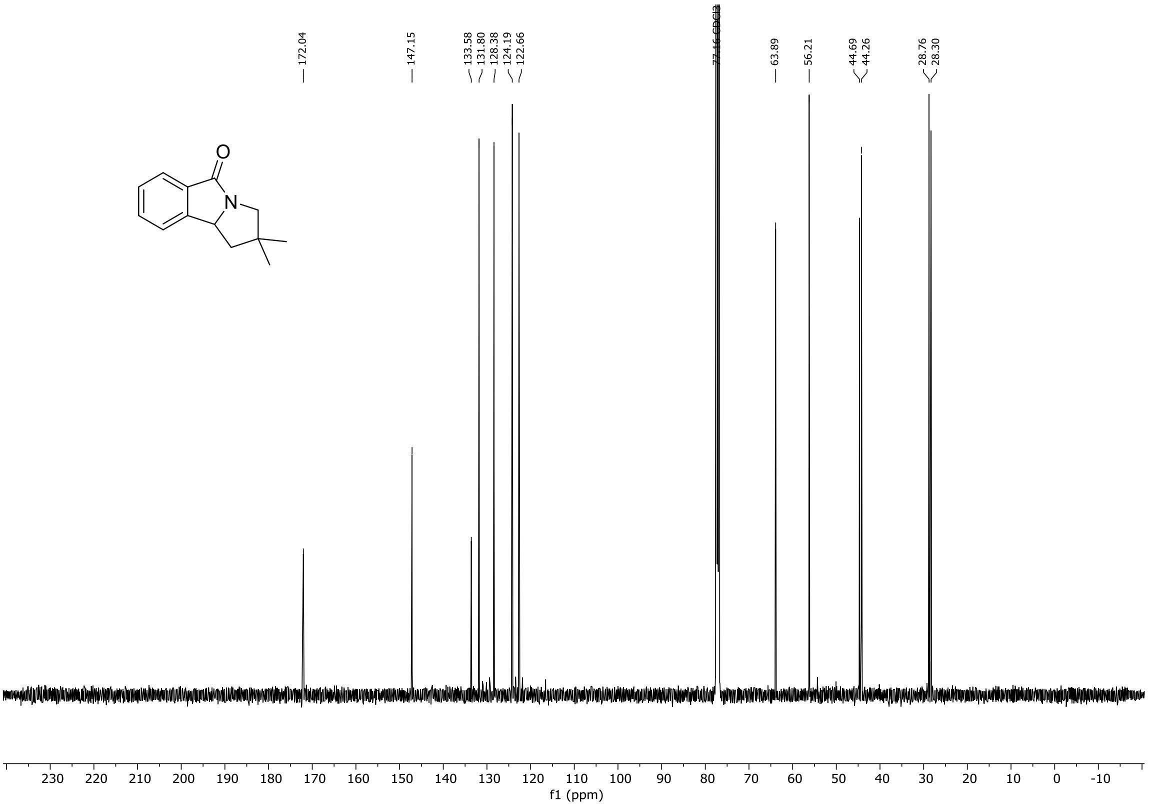

*8.4.3. 6-Bromo-1,2,3,9b-tetrahydro-5H-pyrrolo[2,1-a]isoindol-5-one (**1c**)*

^{1}H NMR (500 MHz, $CDCl_3$):

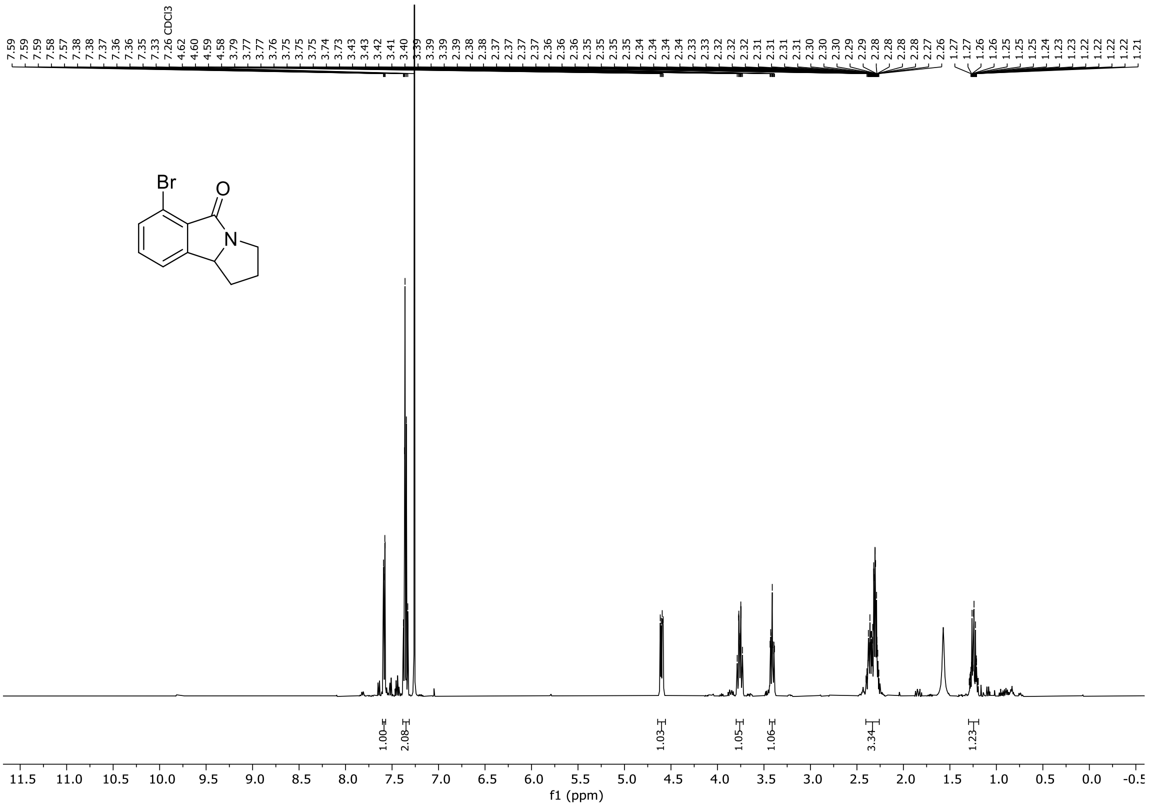

^{13}C NMR (126 MHz, $CDCl_3$):

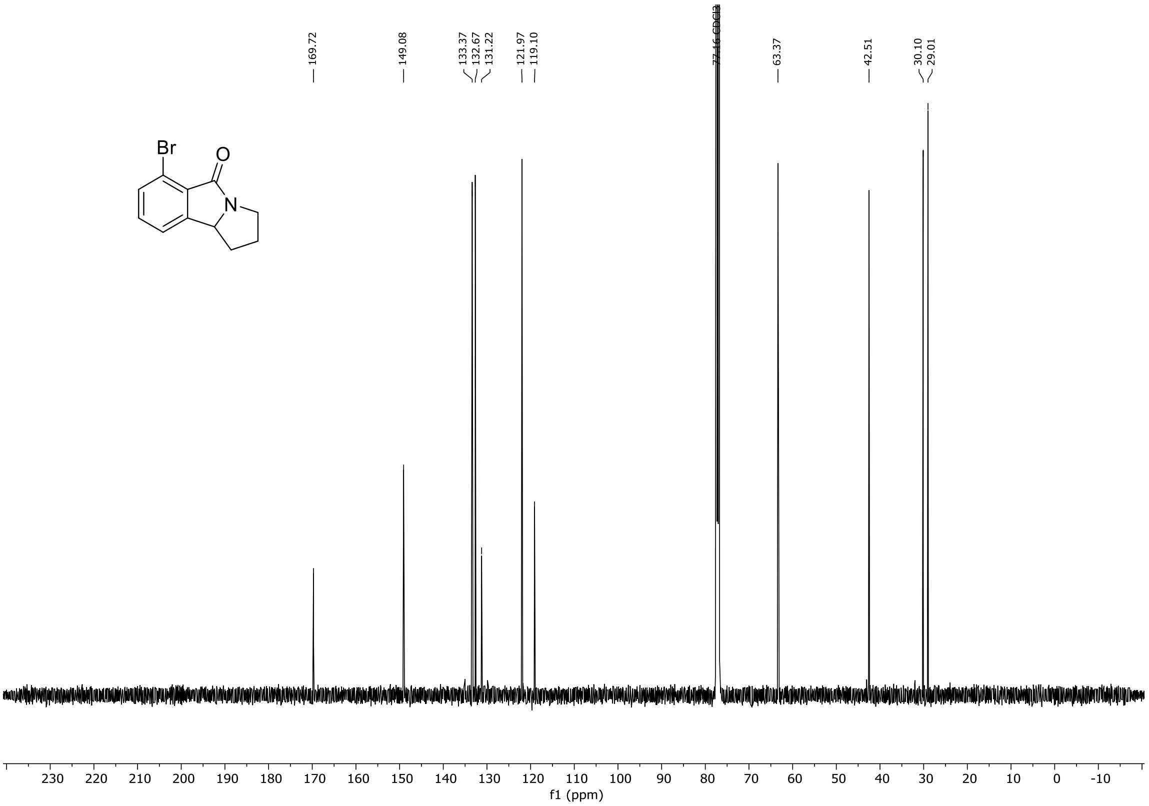

*8.4.4. 6-Methyl-1,2,3,9b-tetrahydro-5H-pyrrolo[2,1-a]isoindol-5-one (**1d**)*

^{1}H NMR (500 MHz, $CDCl_3$):

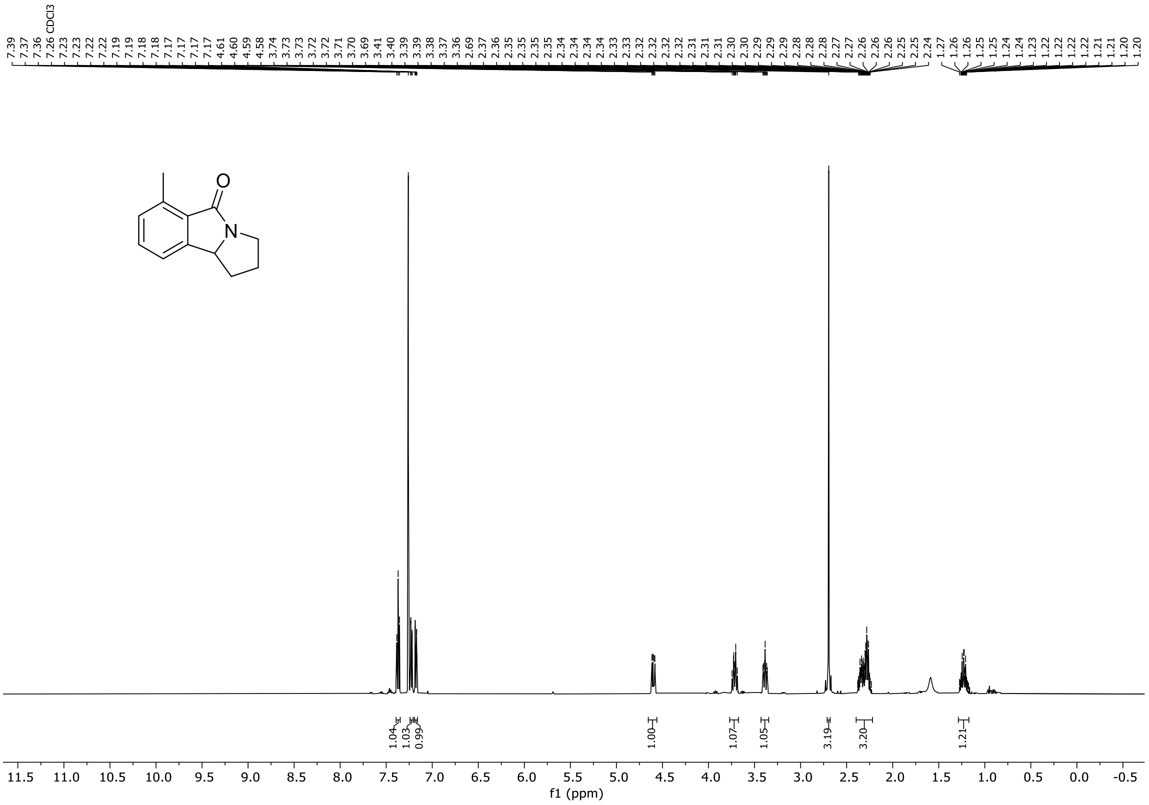

^{13}C NMR (126 MHz, $CDCl_3$):

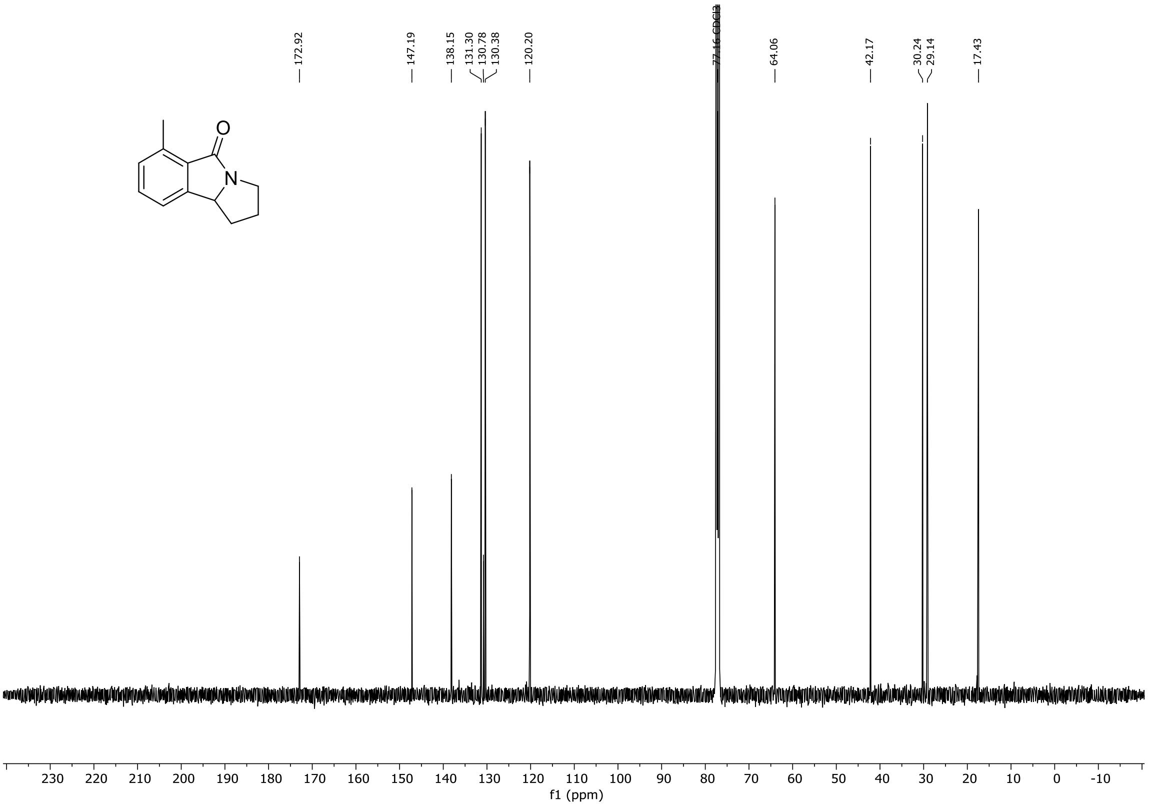

*8.4.5. 7-Chloro-1,2,3,9b-tetrahydro-5H-pyrrolo[2,1-a]isoindol-5-one (**1e**)*

^{1}H NMR (500 MHz, $CDCl_3$):

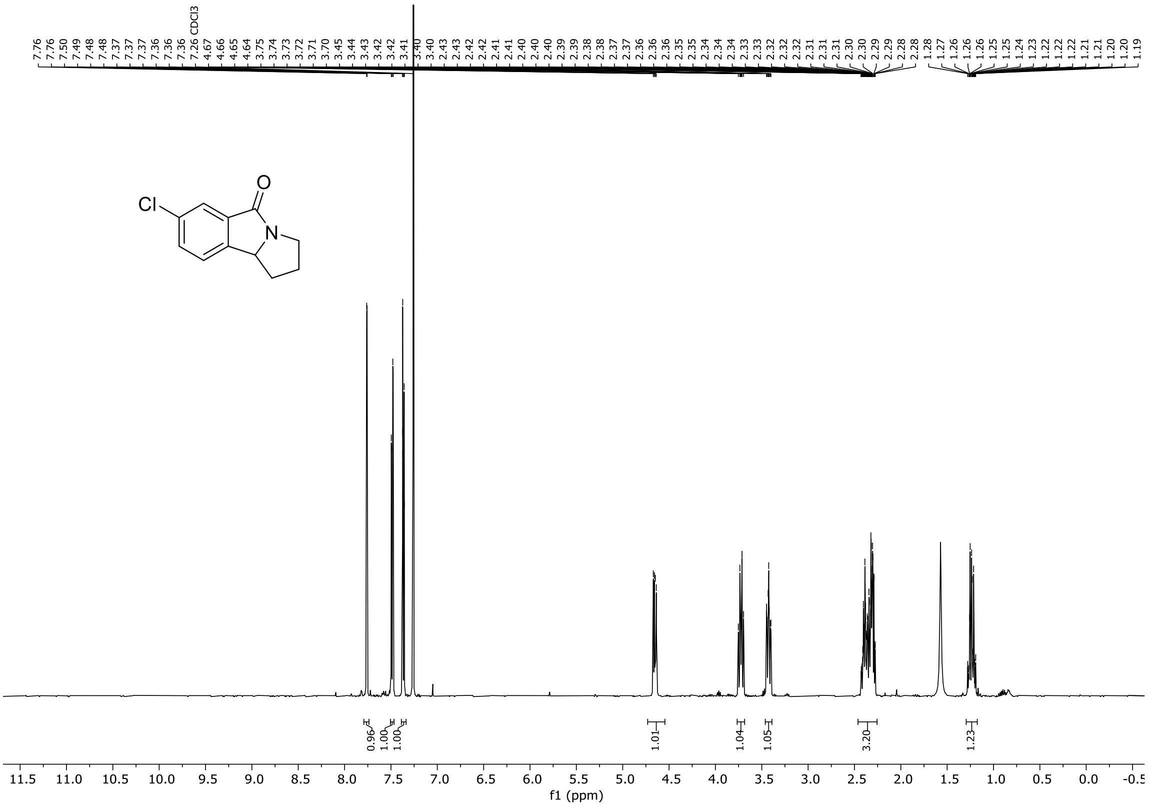

^{13}C NMR (126 MHz, $CDCl_3$):

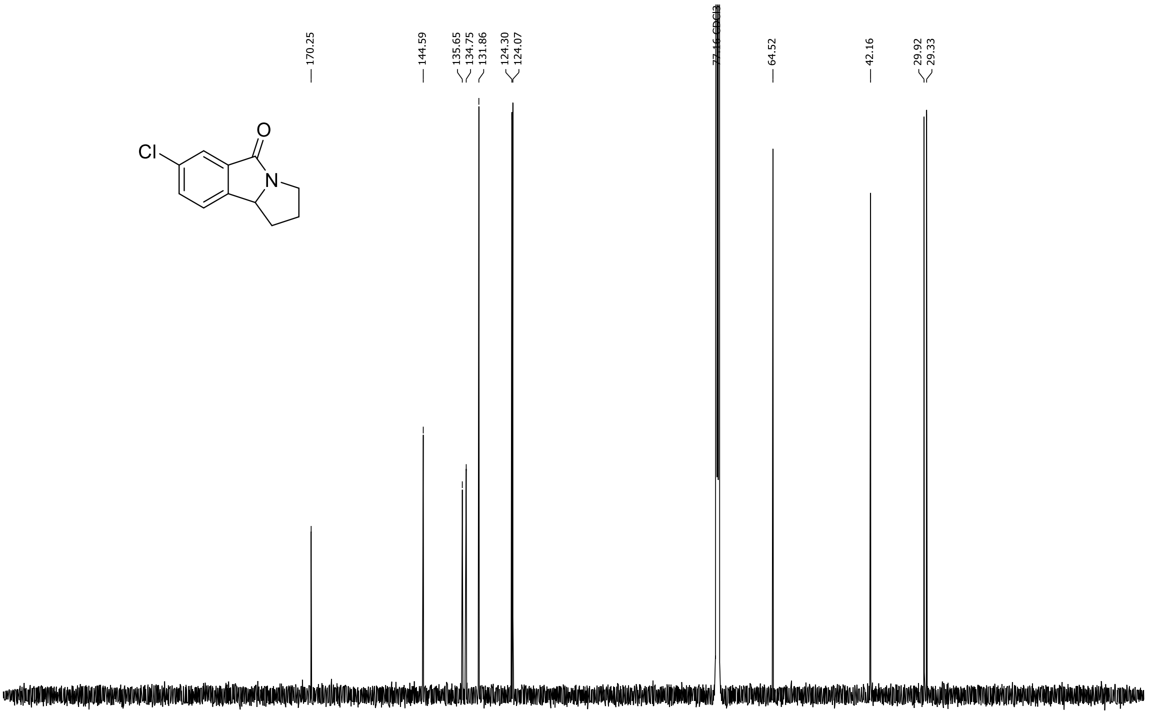

*8.4.6. 7-Bromo-1,2,3,9b-tetrahydro-5H-pyrrolo[2,1-a]isoindol-5-one (**1f**)*

^{1}H NMR (500 MHz, CDCl$_3$):

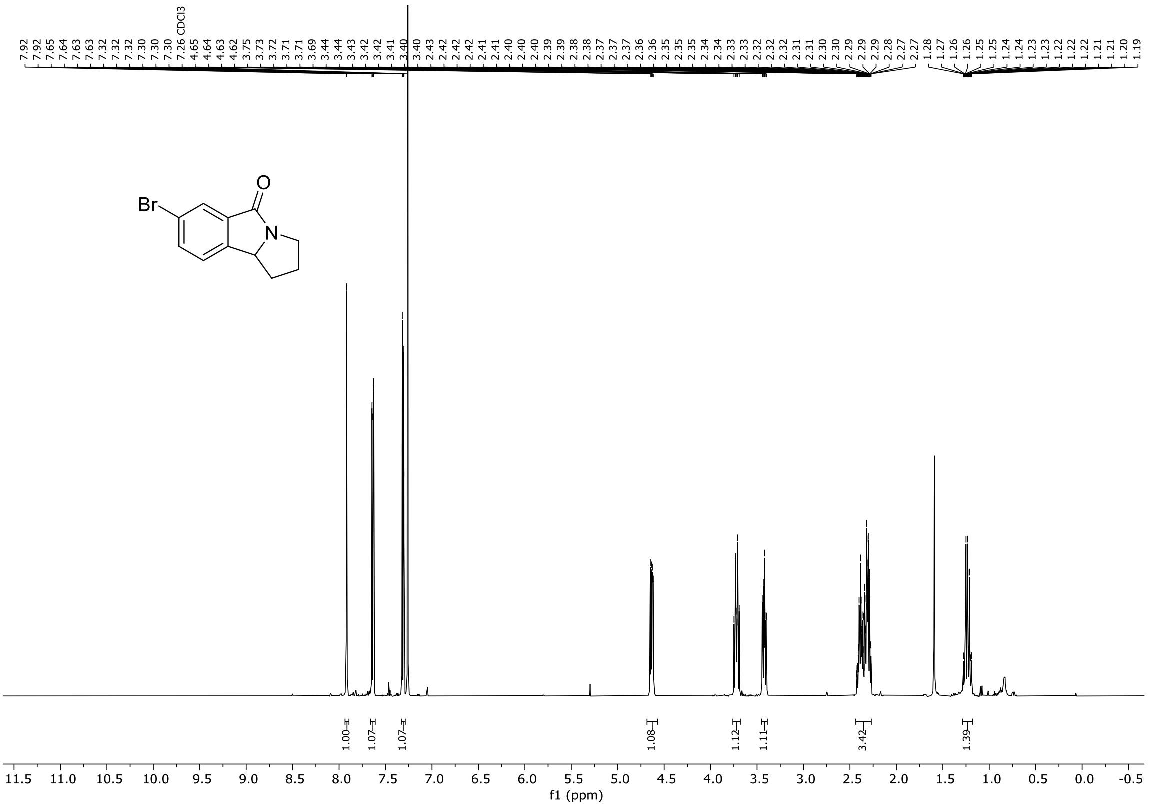

^{13}C NMR (126 MHz, CDCl$_3$):

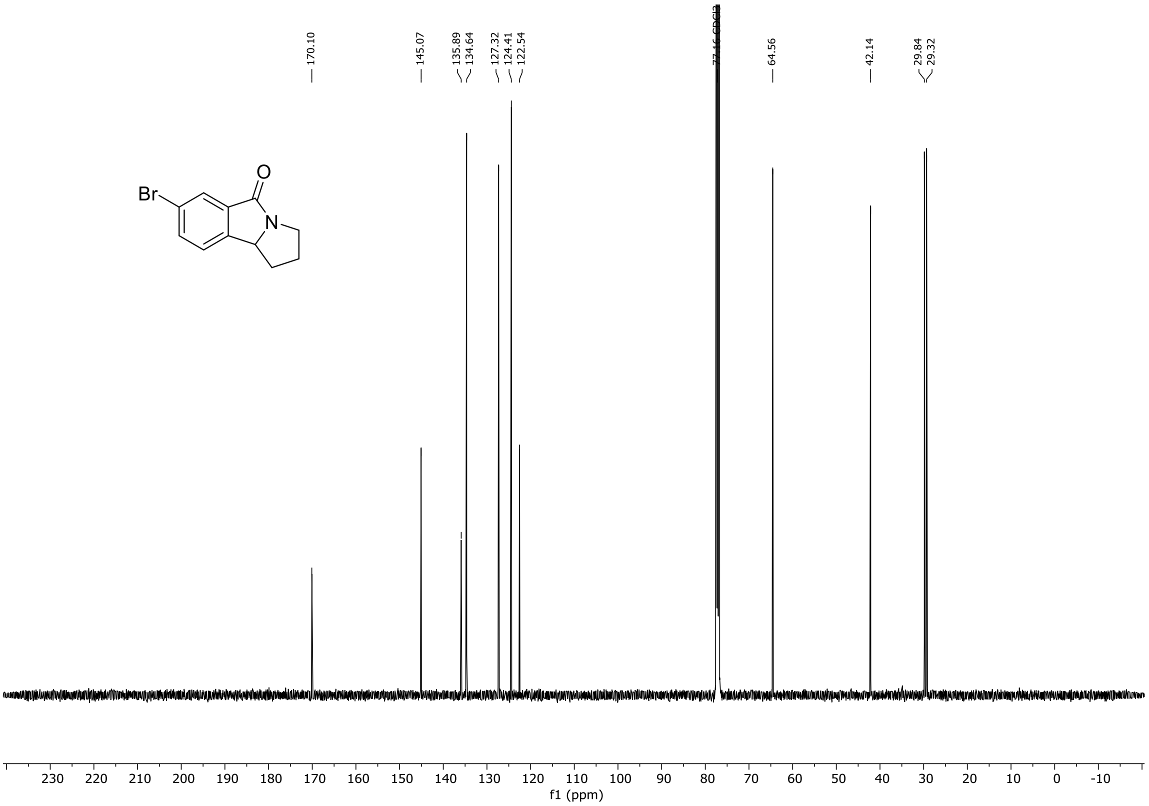

*8.4.7. 8-Fluoro-1,2,3,9b-tetrahydro-5H-pyrrolo[2,1-a]isoindol-5-one (**1g**)*

^{1}H NMR (500 MHz, CDCl$_3$):

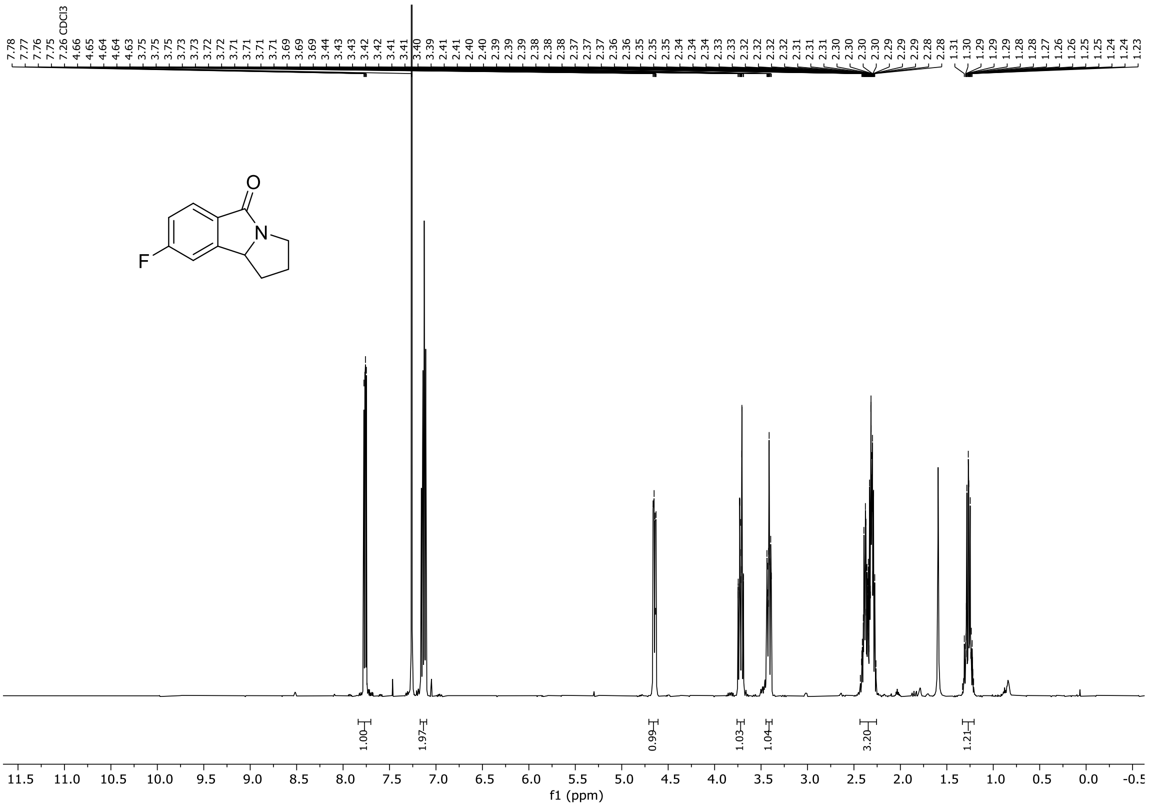

^{13}C NMR (126 MHz, CDCl$_3$):

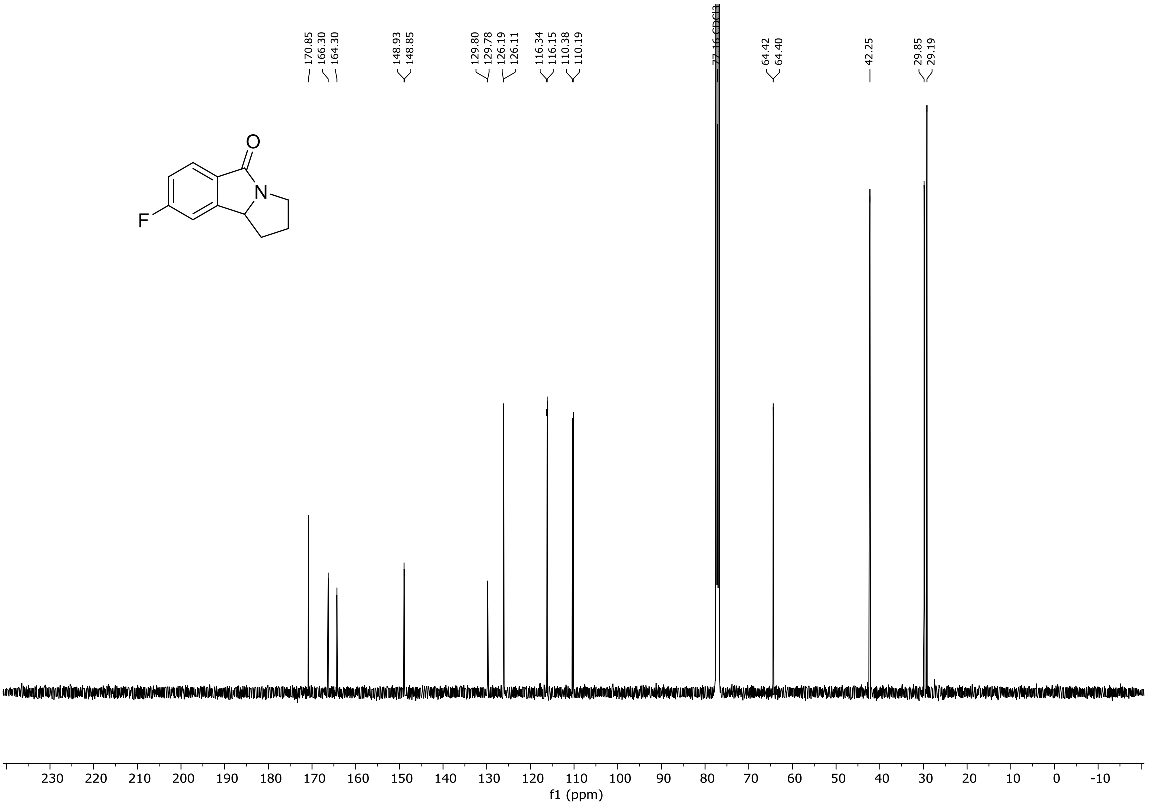

^{19}F NMR (376 MHz, $CDCl_3$):

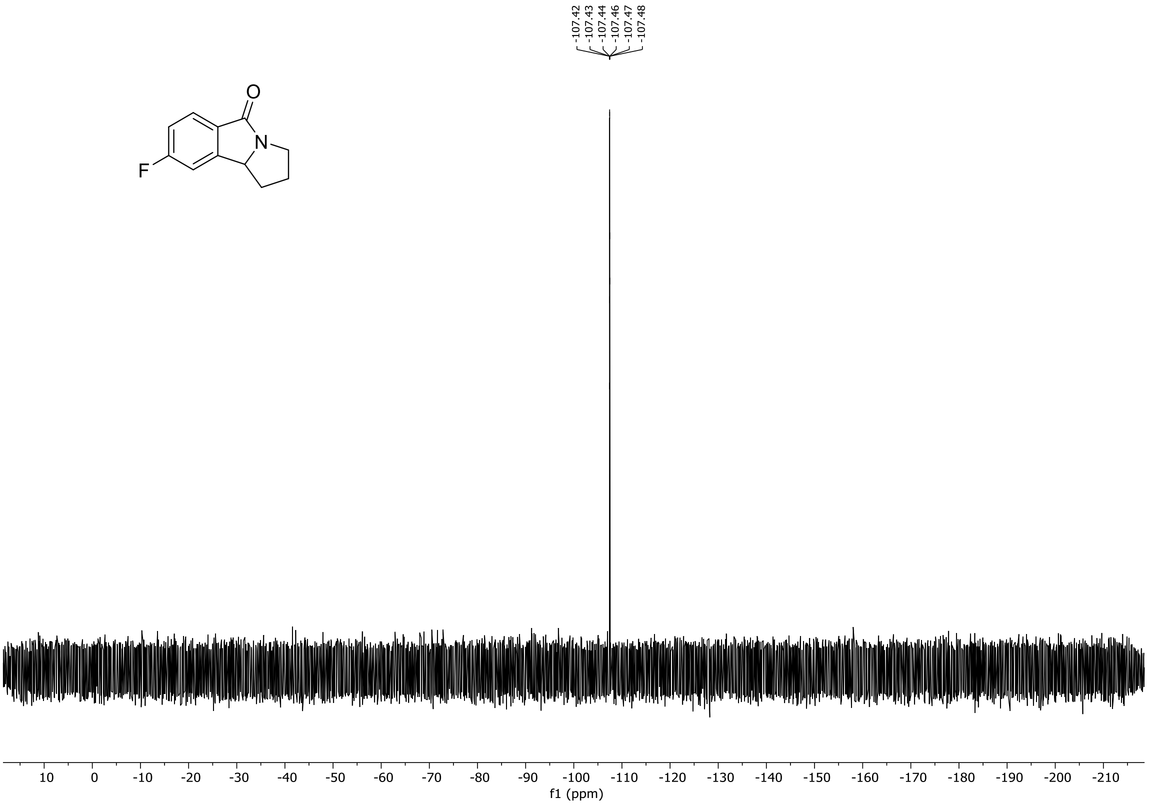

*8.4.8. 8-Bromo-1,2,3,9b-tetrahydro-5H-pyrrolo[2,1-a]isoindol-5-one (**1h**)*

^{1}H NMR (500 MHz, $CDCl_3$):

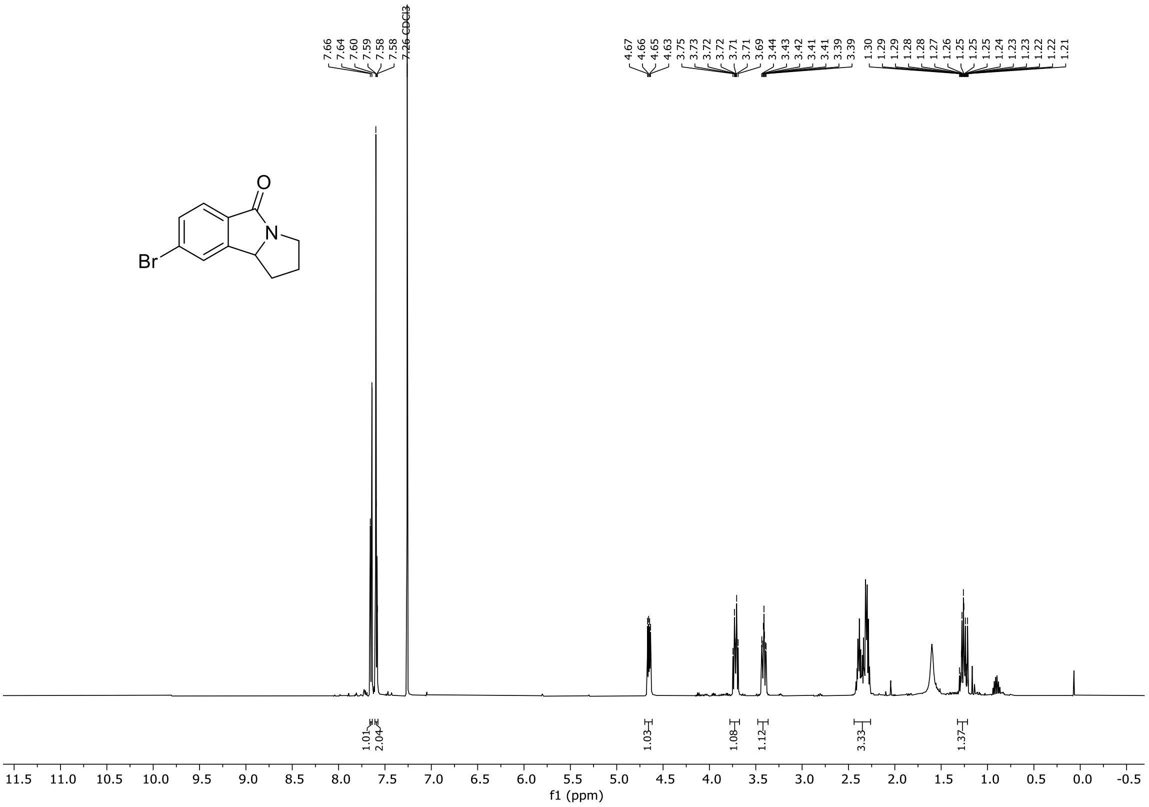

^{13}C NMR (126 MHz, $CDCl_3$):

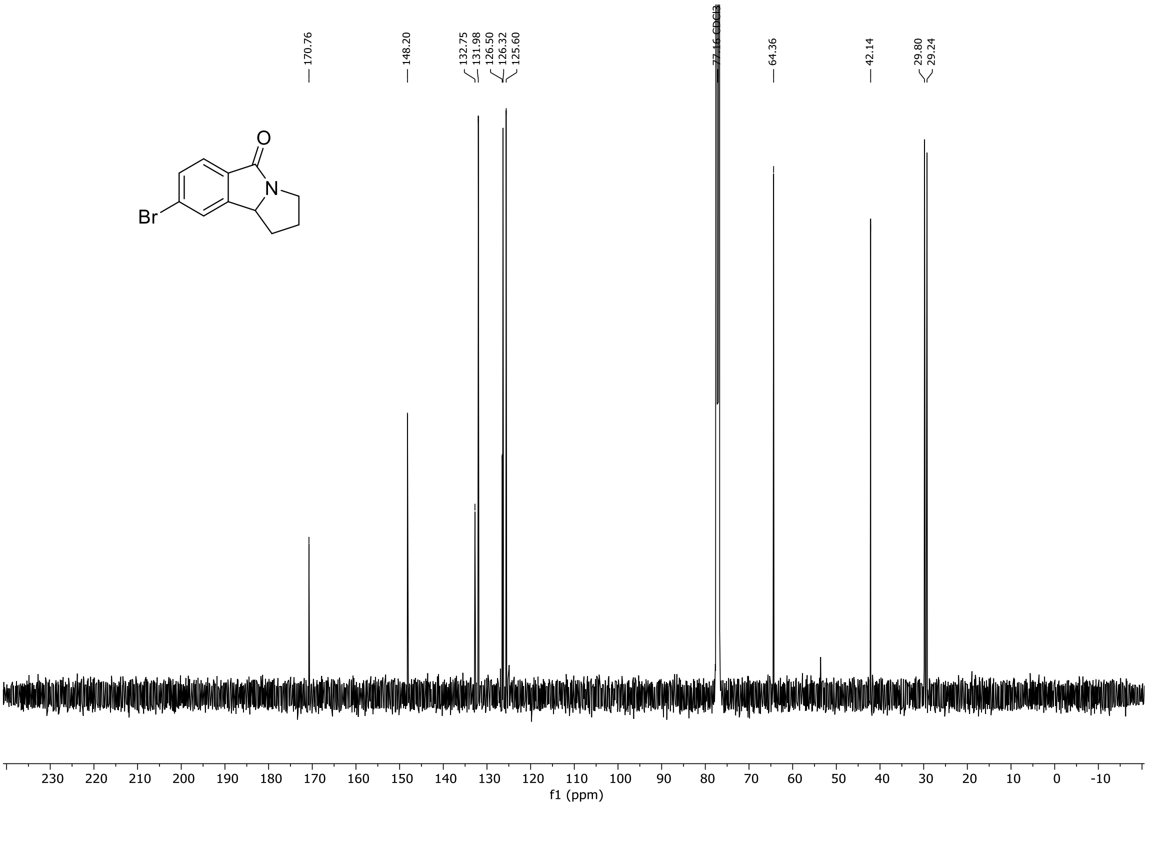

*8.4.9. 8-Nitro-1,2,3,9b-tetrahydro-5H-pyrrolo[2,1-a]isoindol-5-one (**1i**)*

^{1}H NMR (500 MHz, $CDCl_3$):

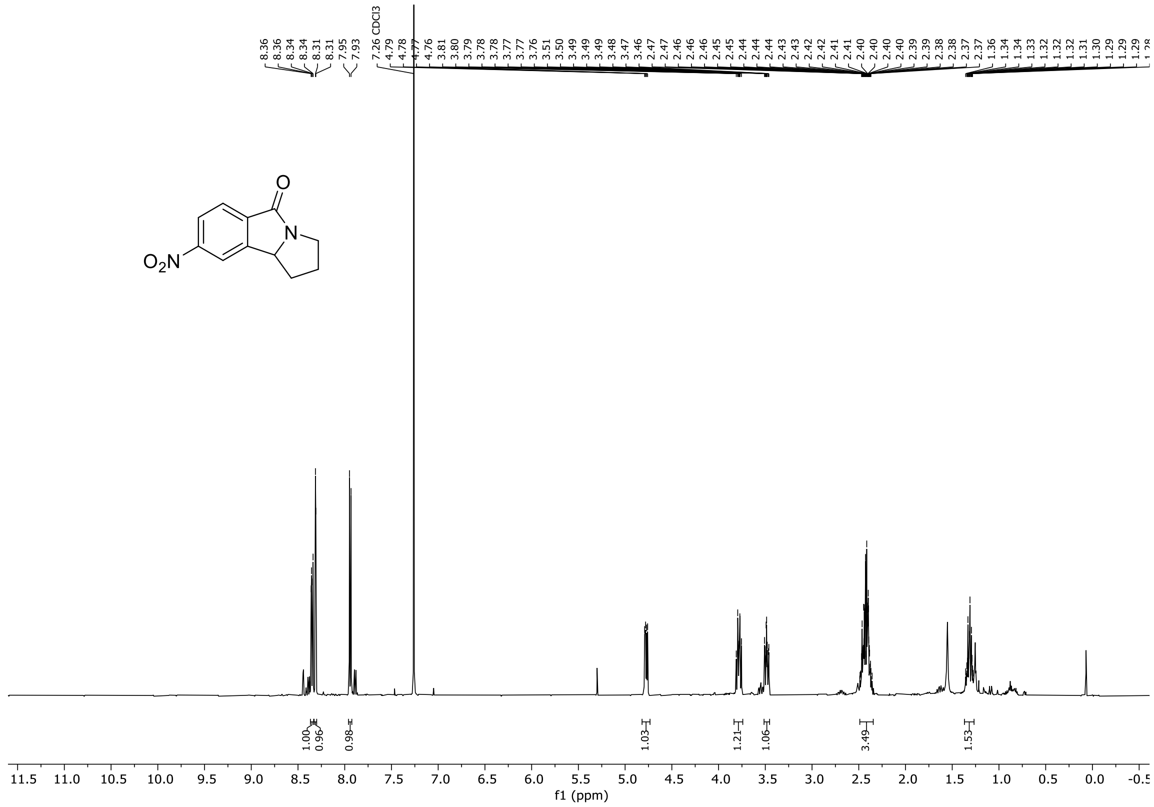

^{13}C NMR (126 MHz, $CDCl_3$):

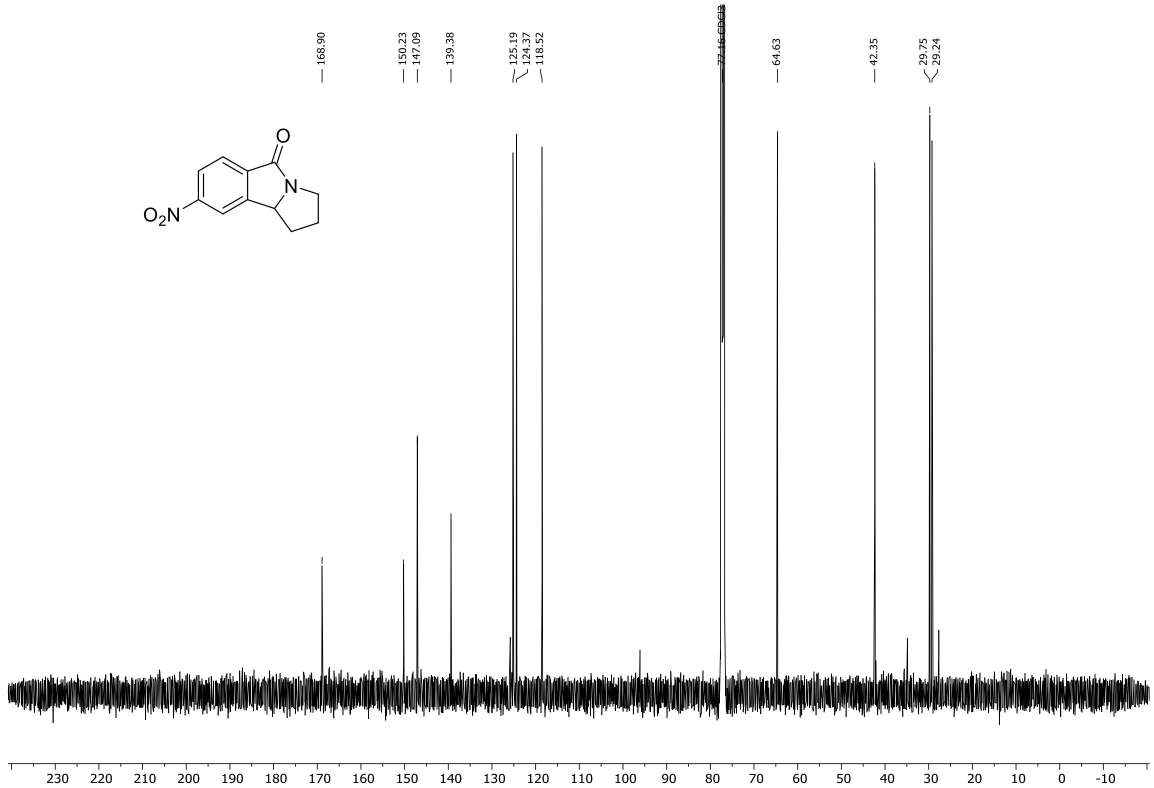

*8.4.10. 5-Oxo-2,3,5,9b-tetrahydro-1H-pyrrolo[2,1-a]isoindole-8-carbonitrile (**1j**)*

^{1}H NMR (500 MHz, $CDCl_3$):

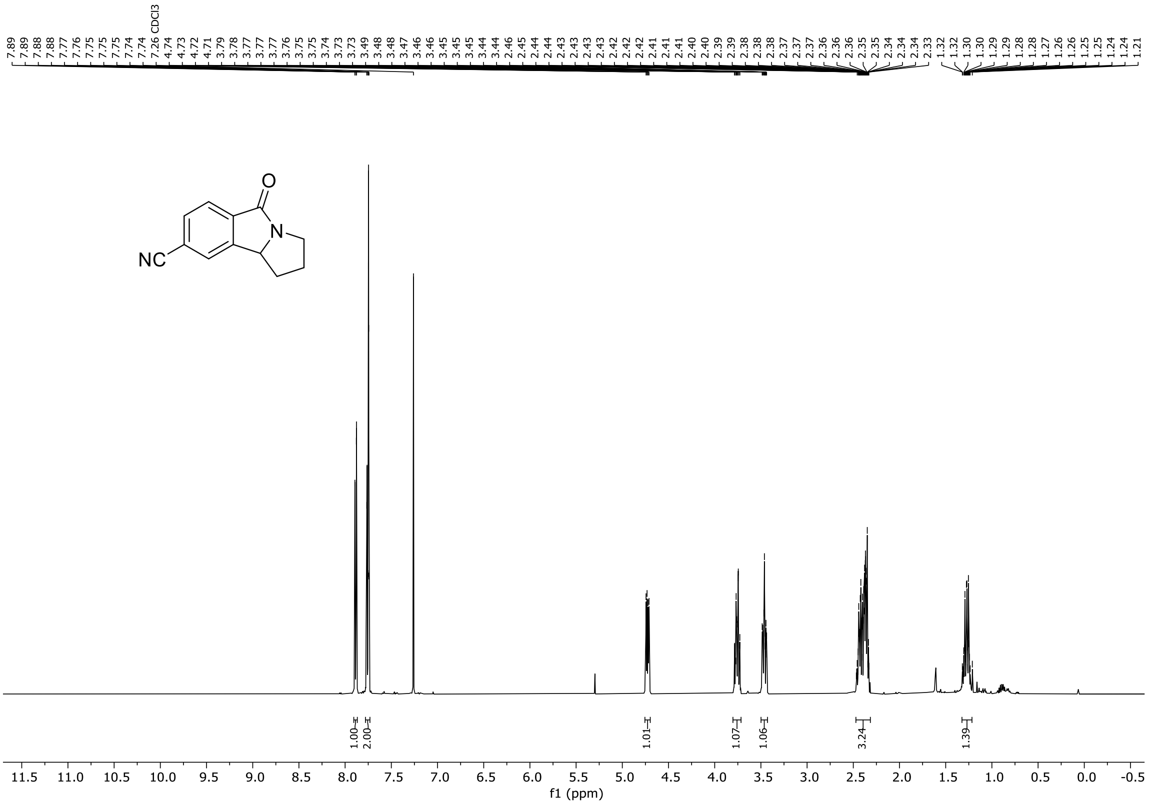

^{13}C NMR (126 MHz, $CDCl_3$):

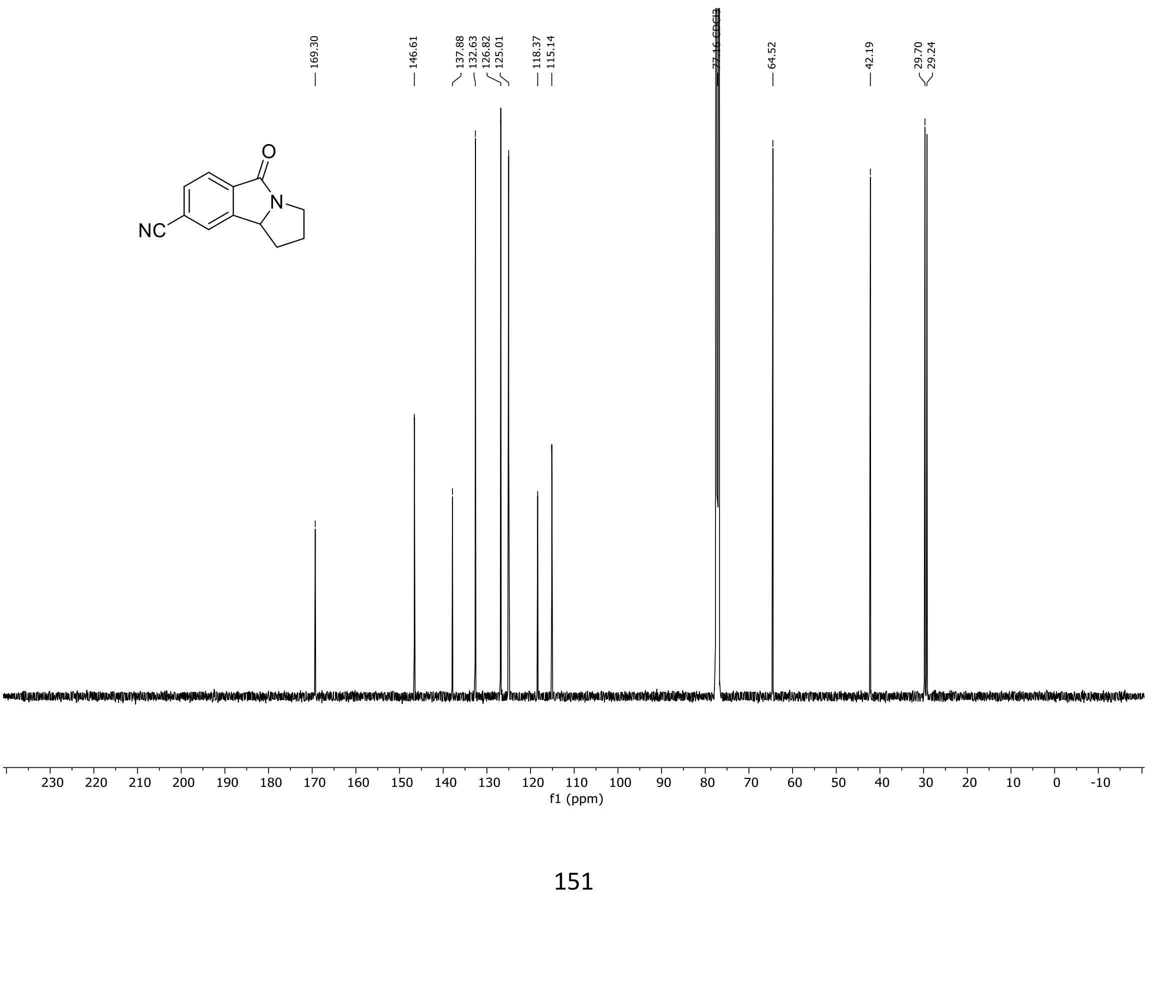

*8.4.11. Methyl 5-oxo-2,3,5,9b-tetrahydro-1H-pyrrolo[2,1-a]isoindole-8-carboxylate (**1k**)*

^1H NMR (500 MHz, $CDCl_3$):

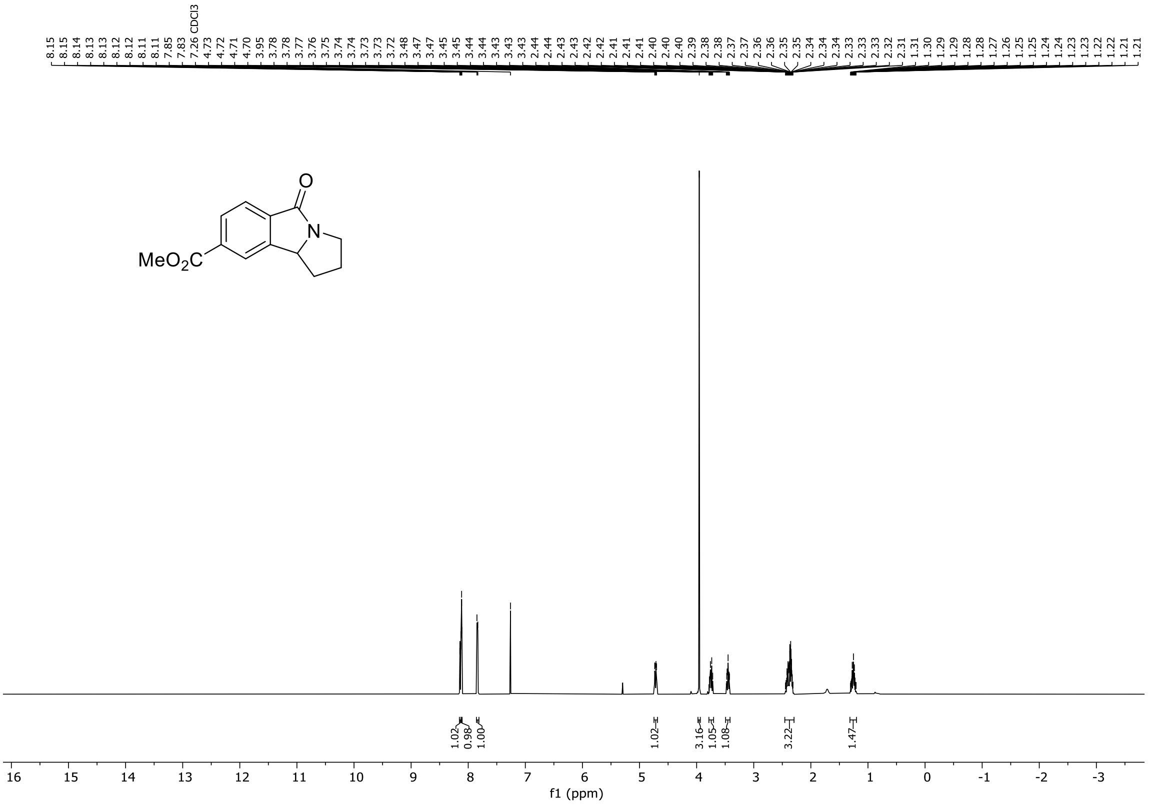

^{13}C NMR (126 MHz, $CDCl_3$):

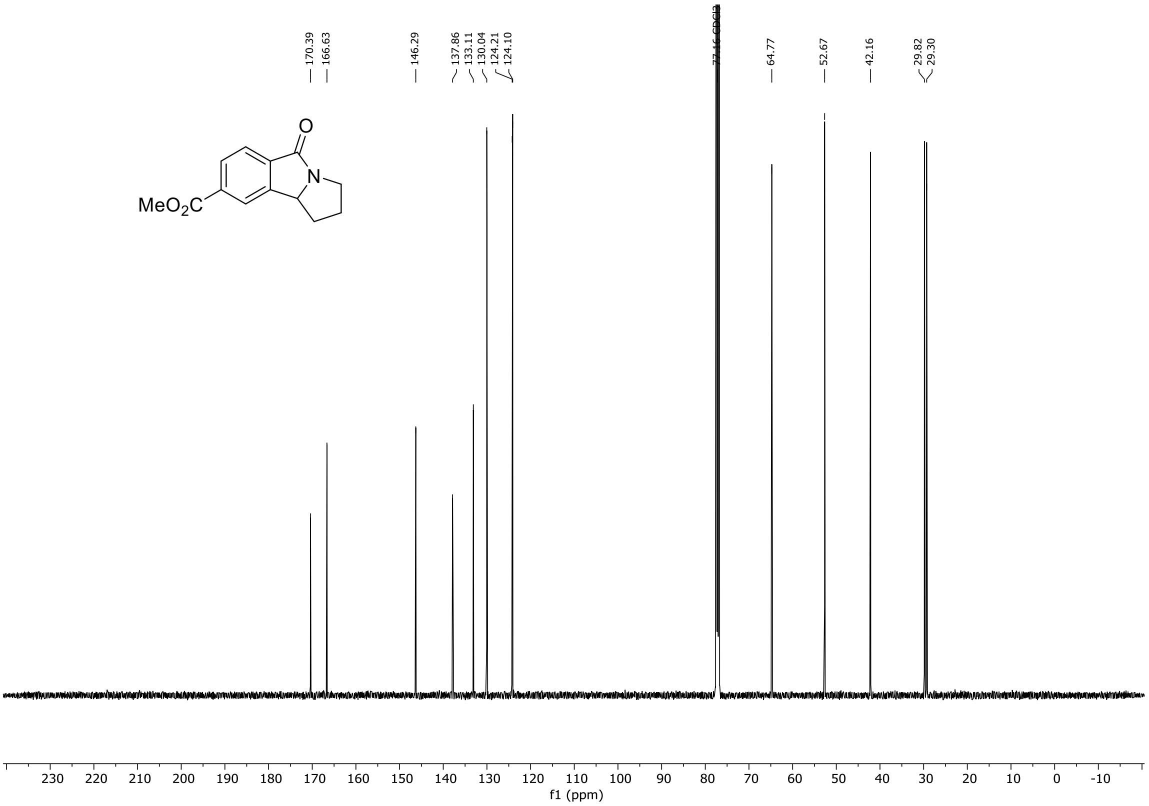

*8.4.12. 9-Methoxy-1,2,3,9b-tetrahydro-5H-pyrrolo[2,1-a]isoindol-5-one (**1l**)*

^{1}H NMR (500 MHz, $CDCl_3$):

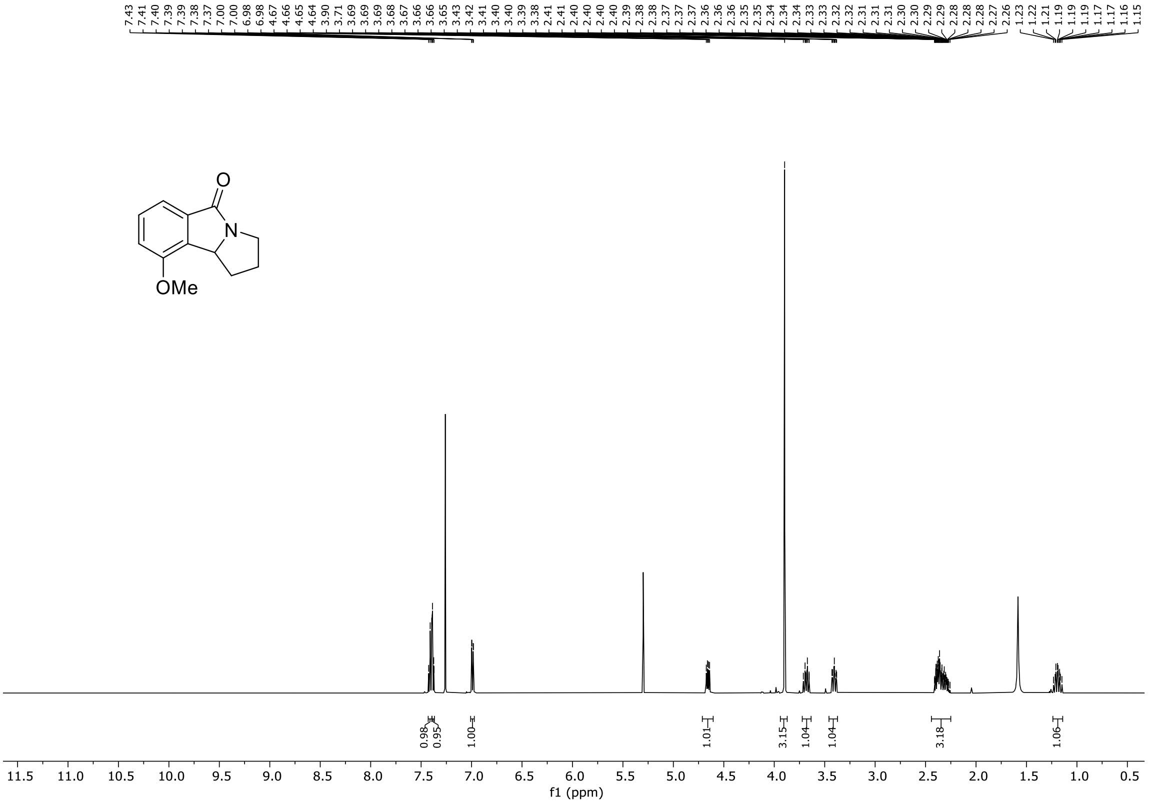

^{13}C NMR (126 MHz, $CDCl_3$):

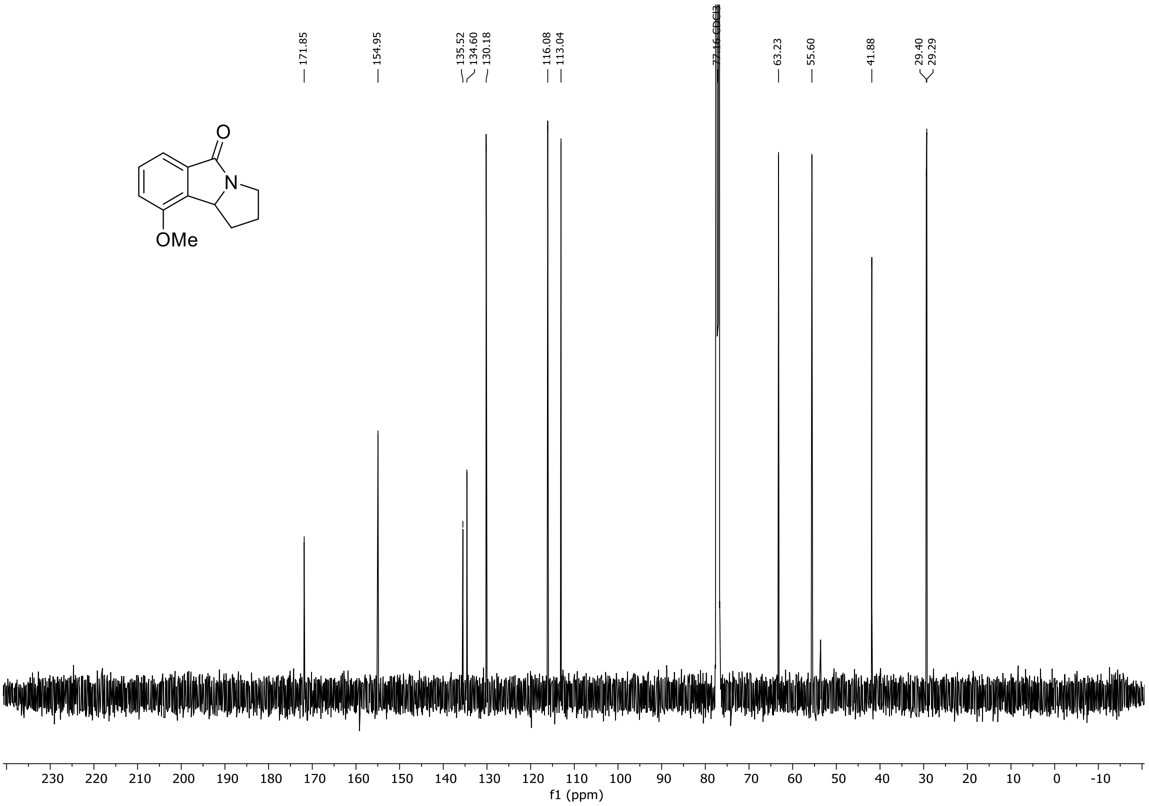

*8.4.13. 9-Bromo-1,2,3,9b-tetrahydro-5H-pyrrolo[2,1-a]isoindol-5-one (**1m**)*

^{1}H NMR (500 MHz, $CDCl_3$):

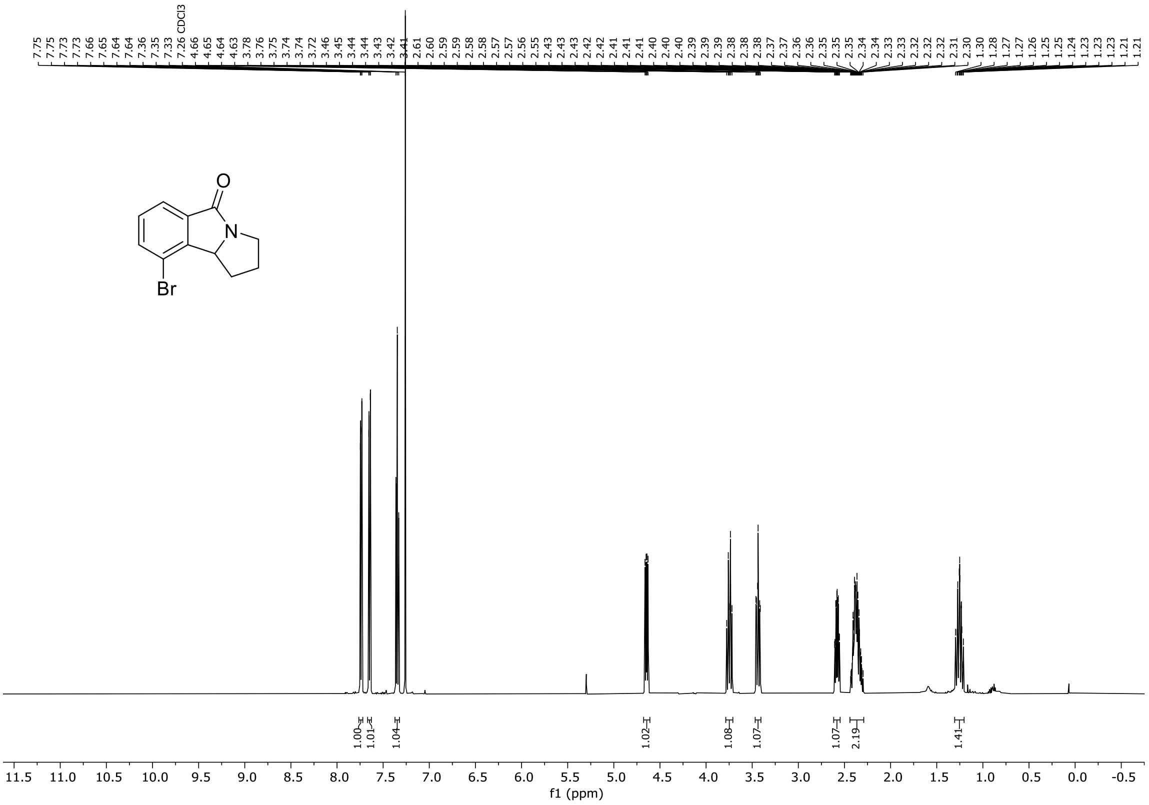

^{13}C NMR (126 MHz, $CDCl_3$):

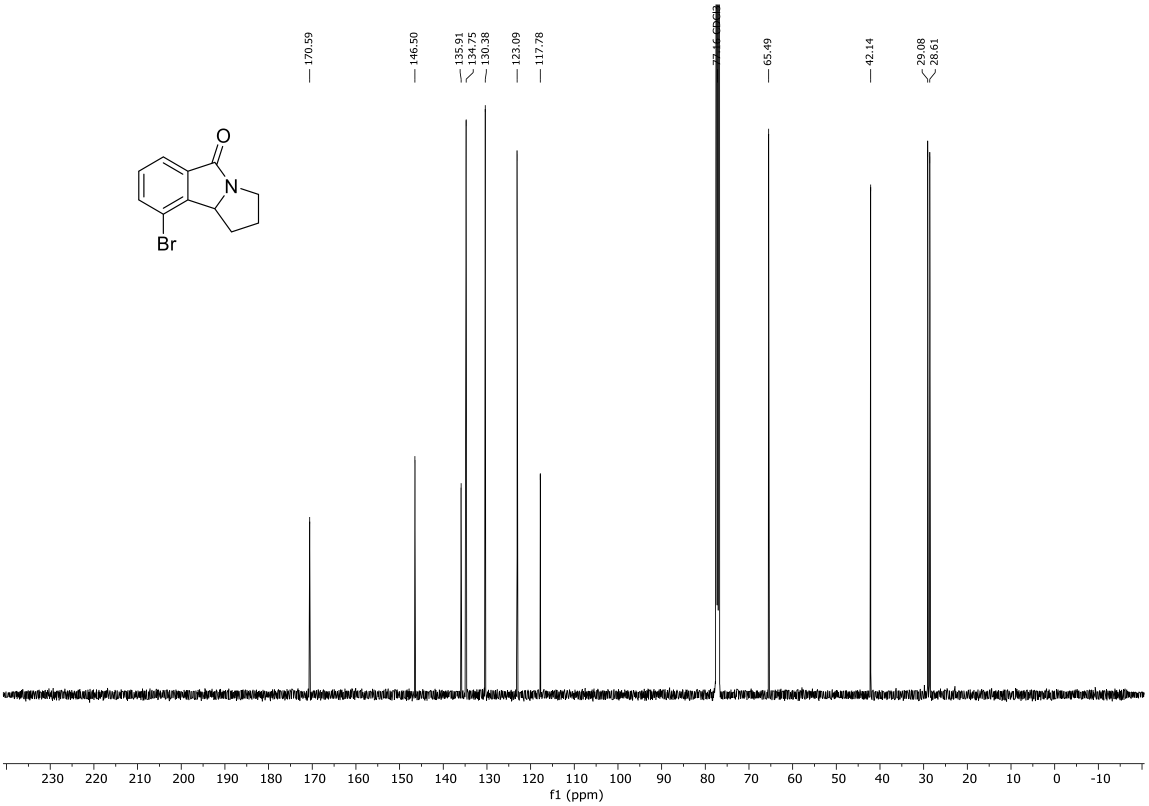

*8.4.14. 9-Trifluormethyl-1,2,3,9b-tetrahydro-5H-pyrrolo[2,1-a]isoindol-5-one (**1n**)*

^{1}H NMR (500 MHz, $CDCl_3$):

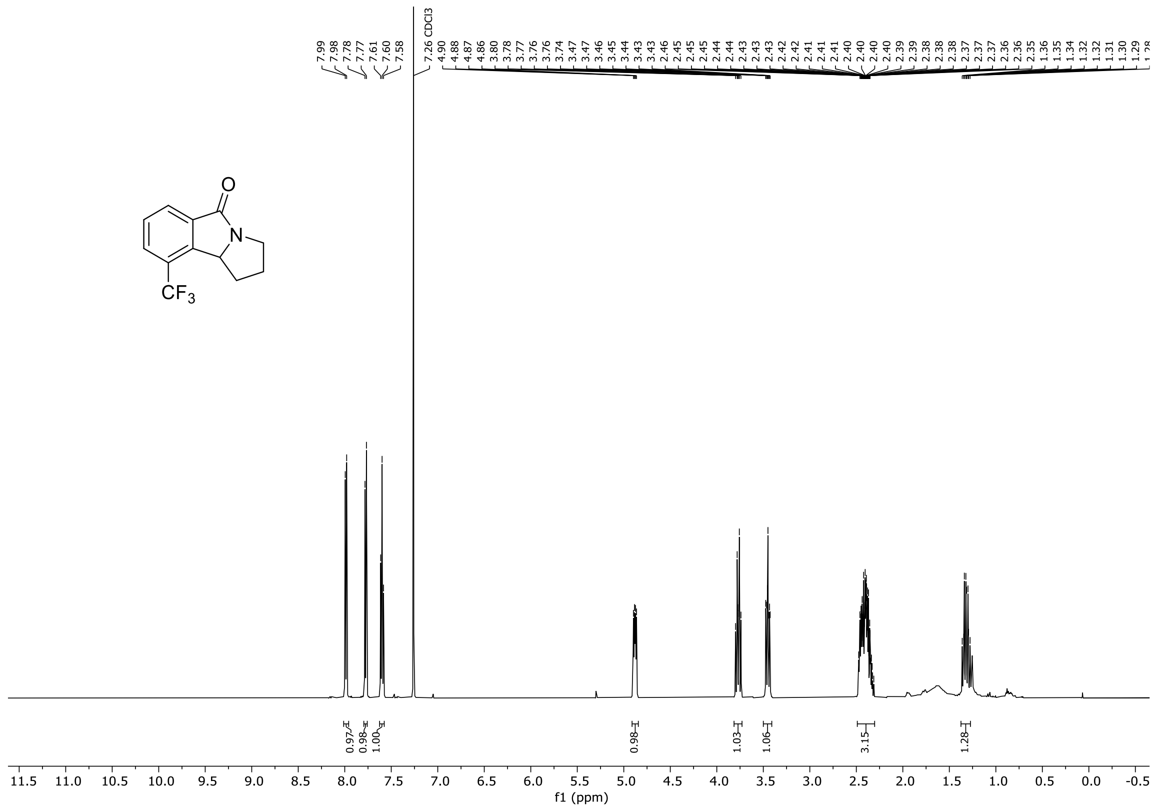

^{13}C NMR (126 MHz, $CDCl_3$):

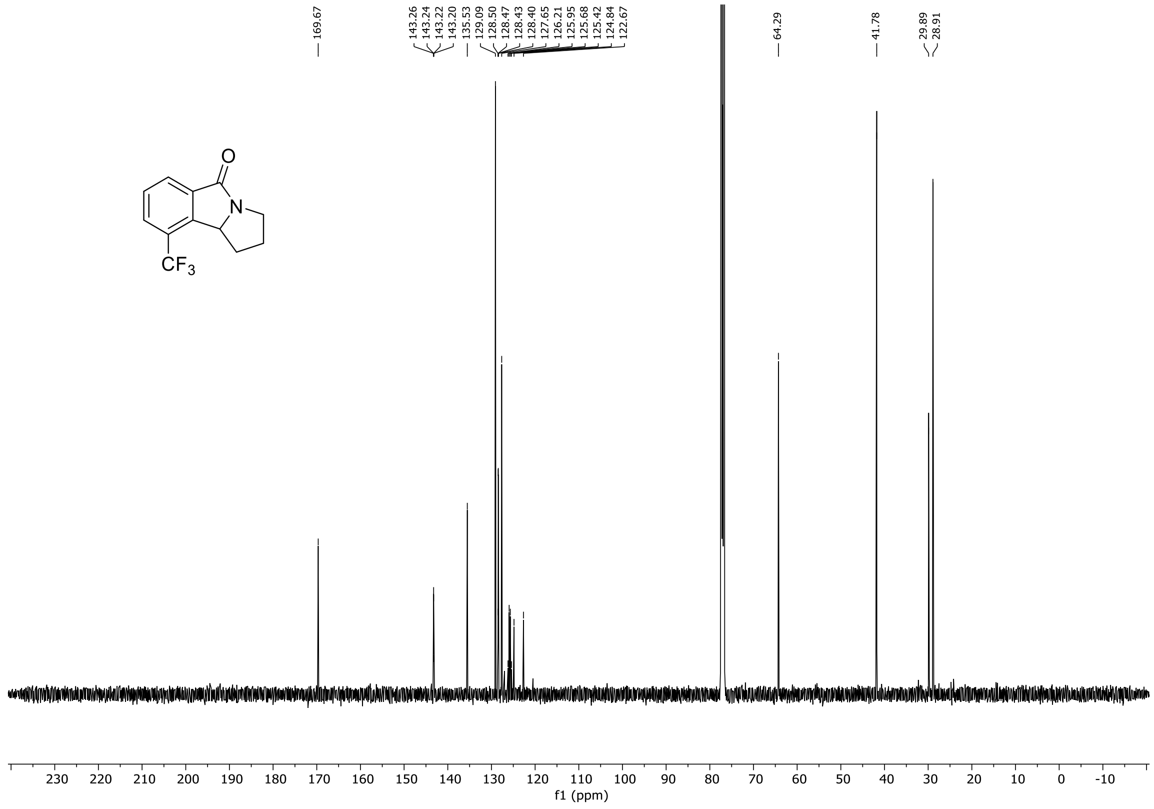

^{19}F NMR (376 MHz, $CDCl_3$):

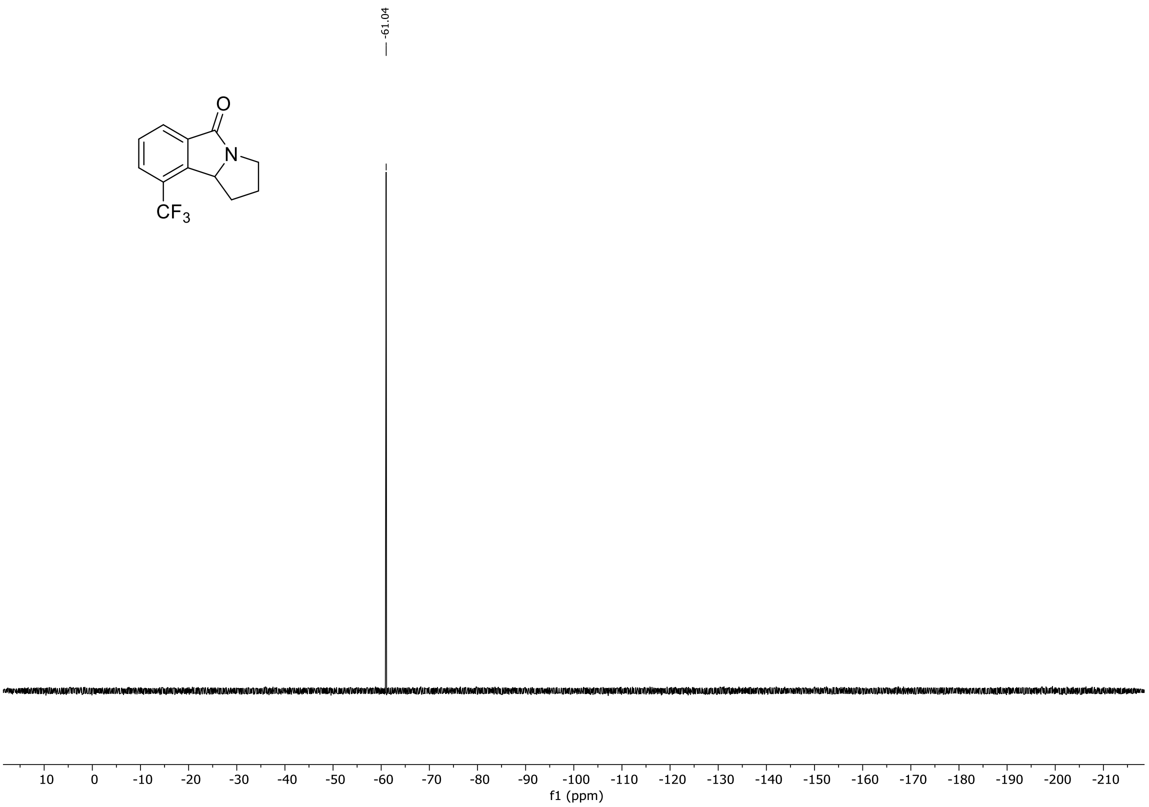

*8.4.15. 9b-Methyl-1,2,3,9b-tetrahydro-5H-pyrrolo[2,1-a]isoindol-5-one (**1o**)*

^{1}H NMR (500 MHz, CDCl$_3$):

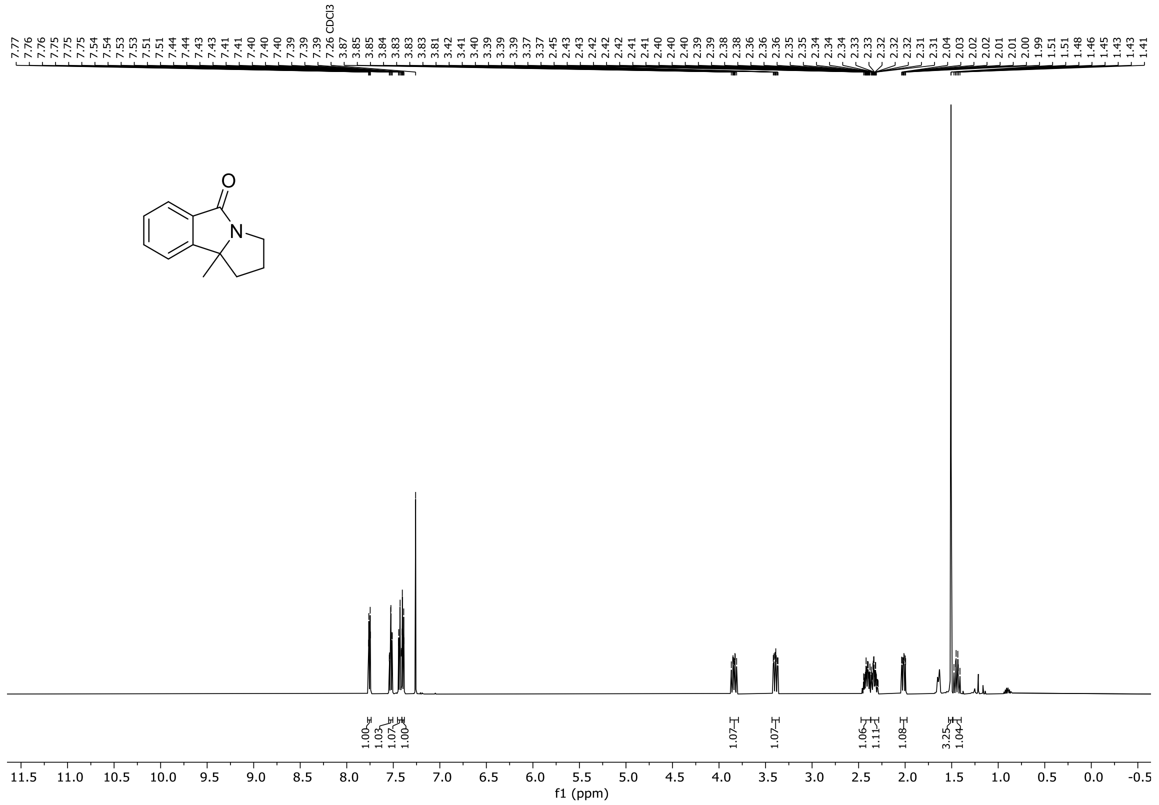

^{13}C NMR (126 MHz, CDCl$_3$):

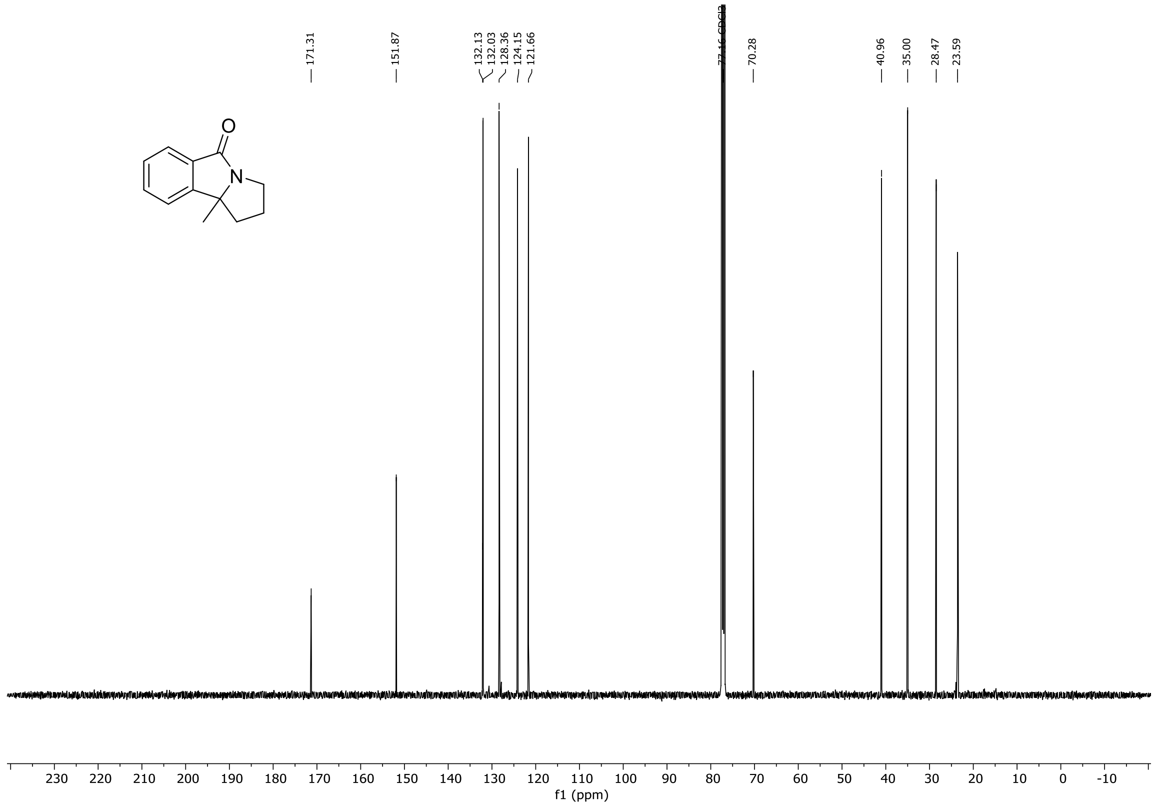

*8.4.16. 7,8,9,9a-Tetrahydro-5H-pyrido[2,3-a]pyrrolizin-5-one (**1p**)*

^{1}H NMR (500 MHz, $CDCl_3$):

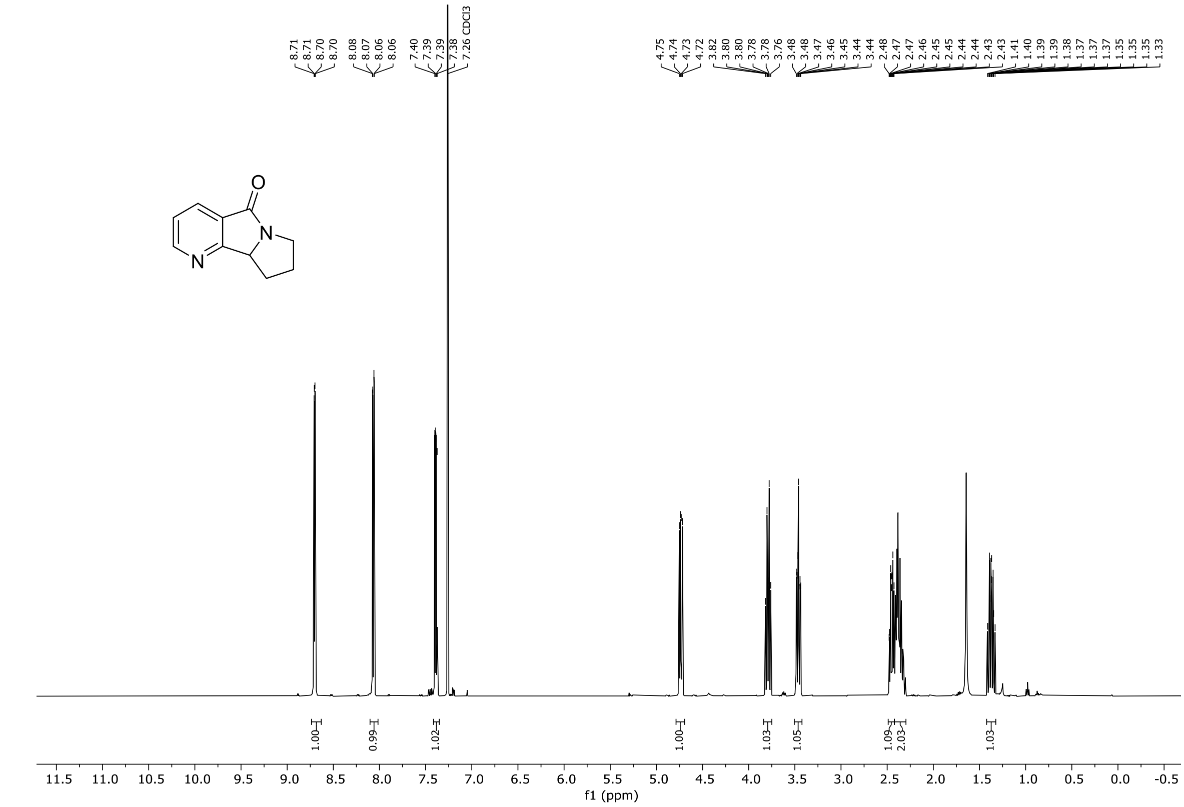

^{13}C NMR (126 MHz, $CDCl_3$):

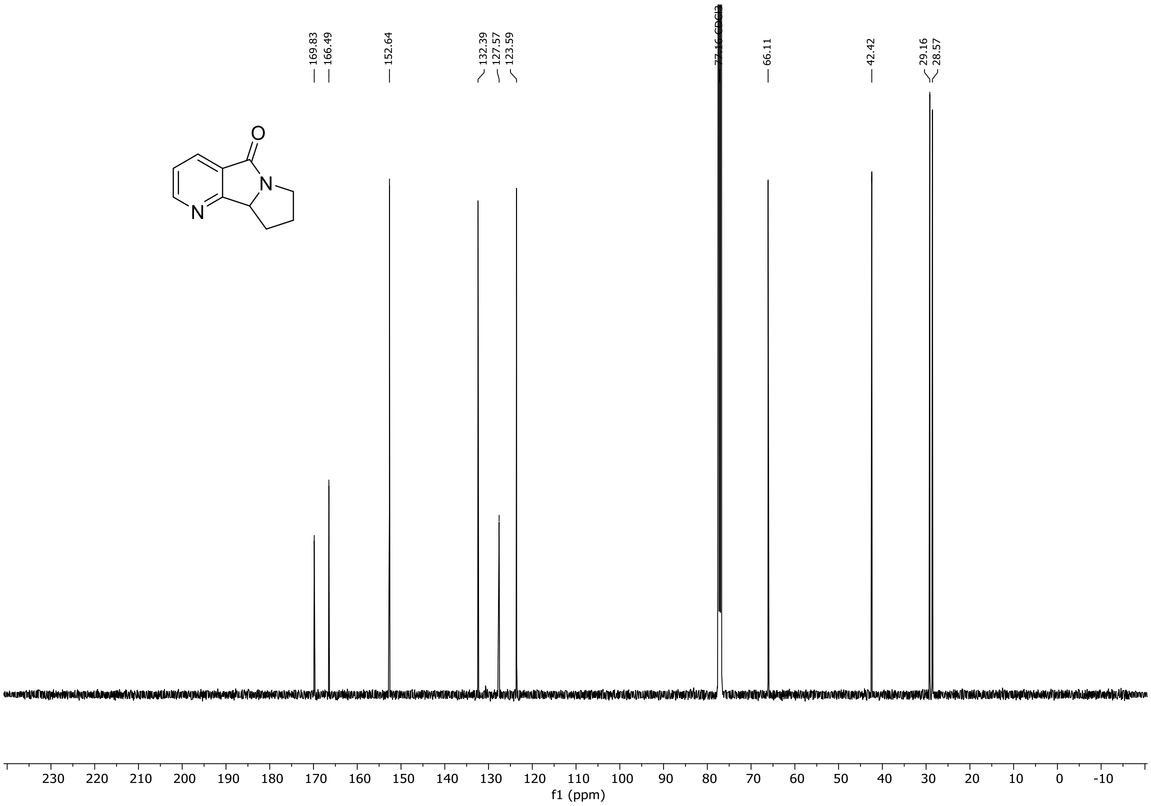

*8.4.17. 1,3,4,10b-Tetrahydropyrido[2,1-a]isoindol-6(2H)-one (**1q**)*

^{1}H NMR (500 MHz, $CDCl_3$):

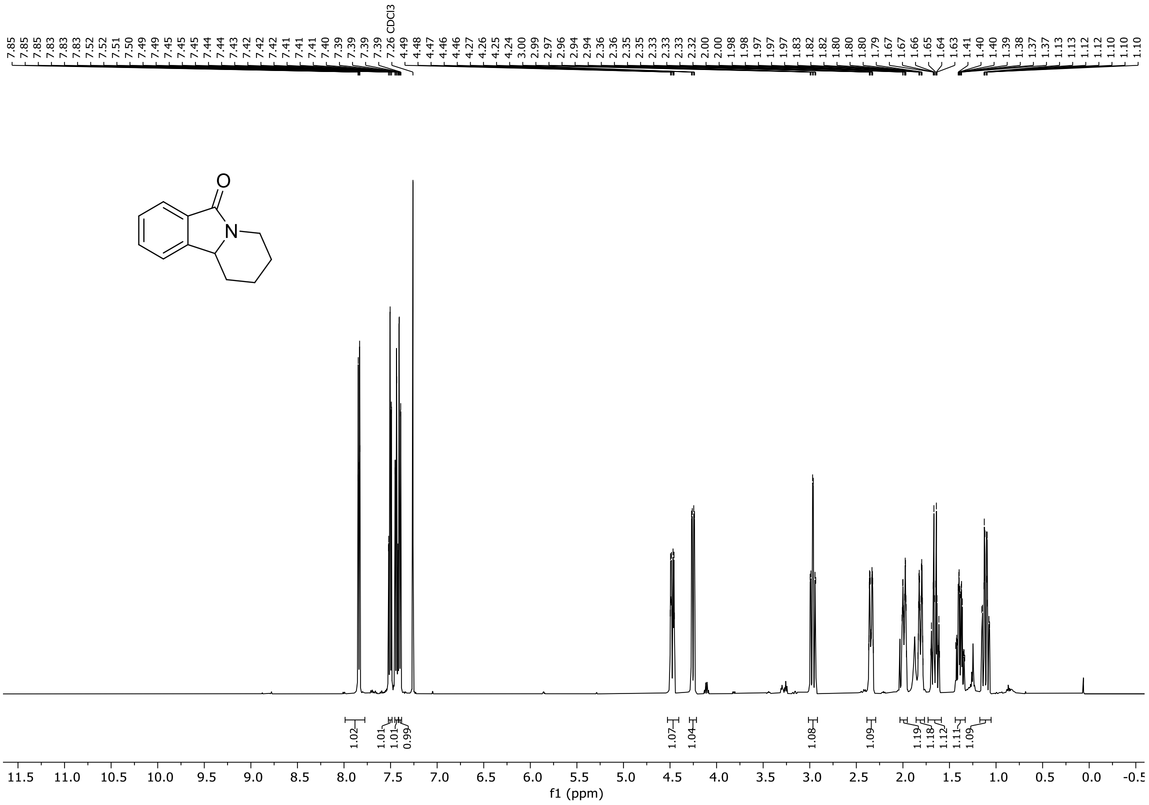

^{13}C NMR (126 MHz, $CDCl_3$):

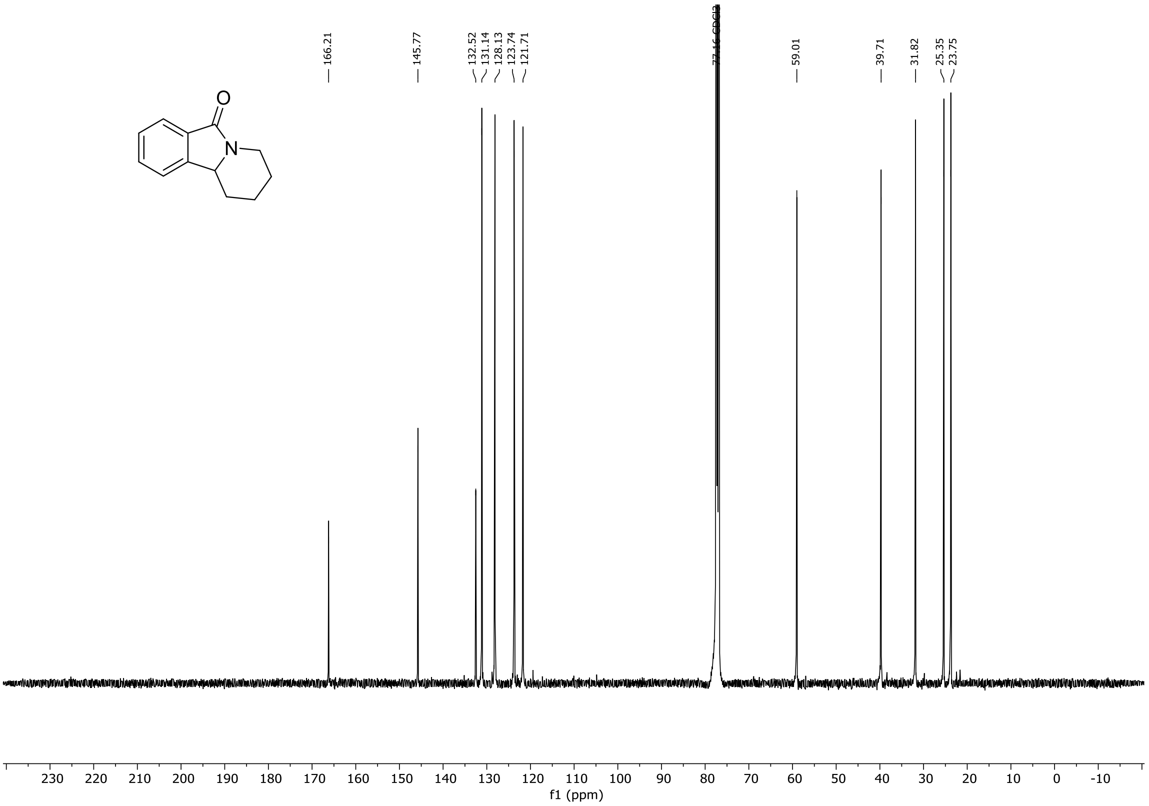

8.4.18. *11b,12-Dihydroisoindolo[2,1-b]isoquinolin-7(5H)-one (**1r**)*

^{1}H NMR (500 MHz, $CDCl_3$):

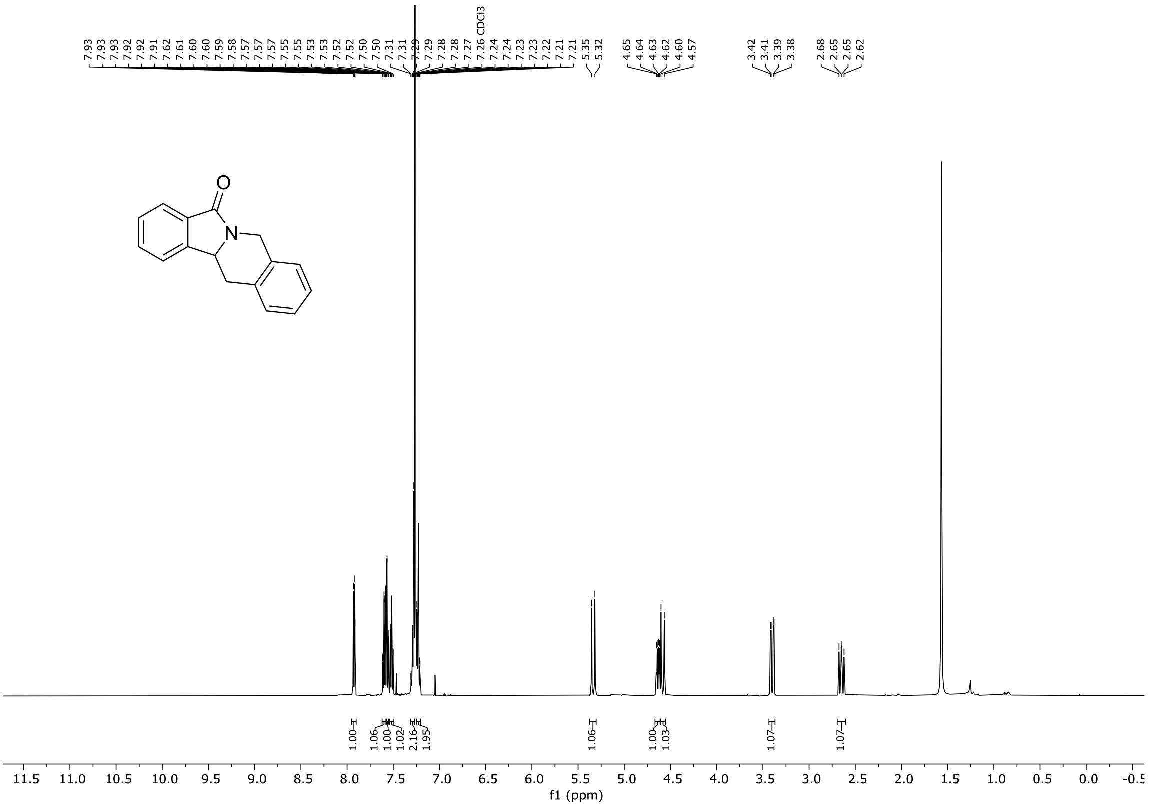

^{13}C NMR (126 MHz, $CDCl_3$):

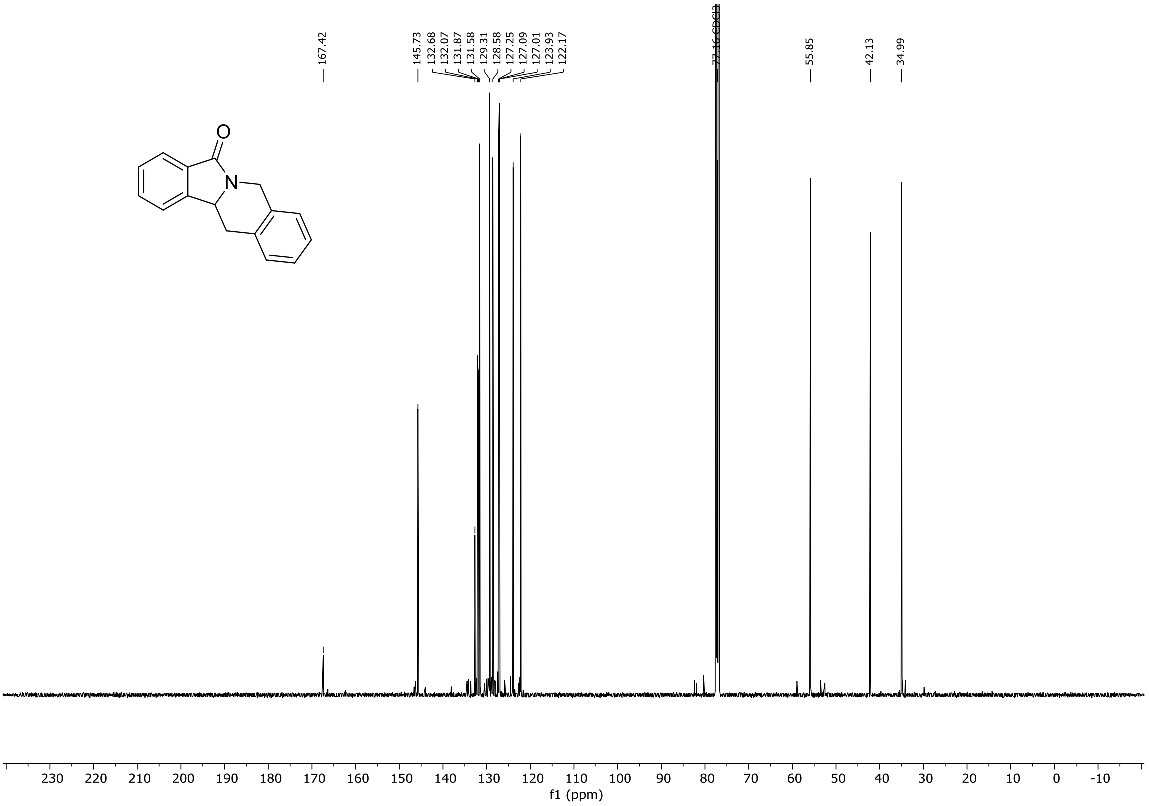

*8.4.19. 7,8,13,13a-tetrahydro-5H-benzo[4,5]azepino[2,1-a]isoindol-5-one (**1s**)*

^{1}H NMR (500 MHz, $CDCl_3$):

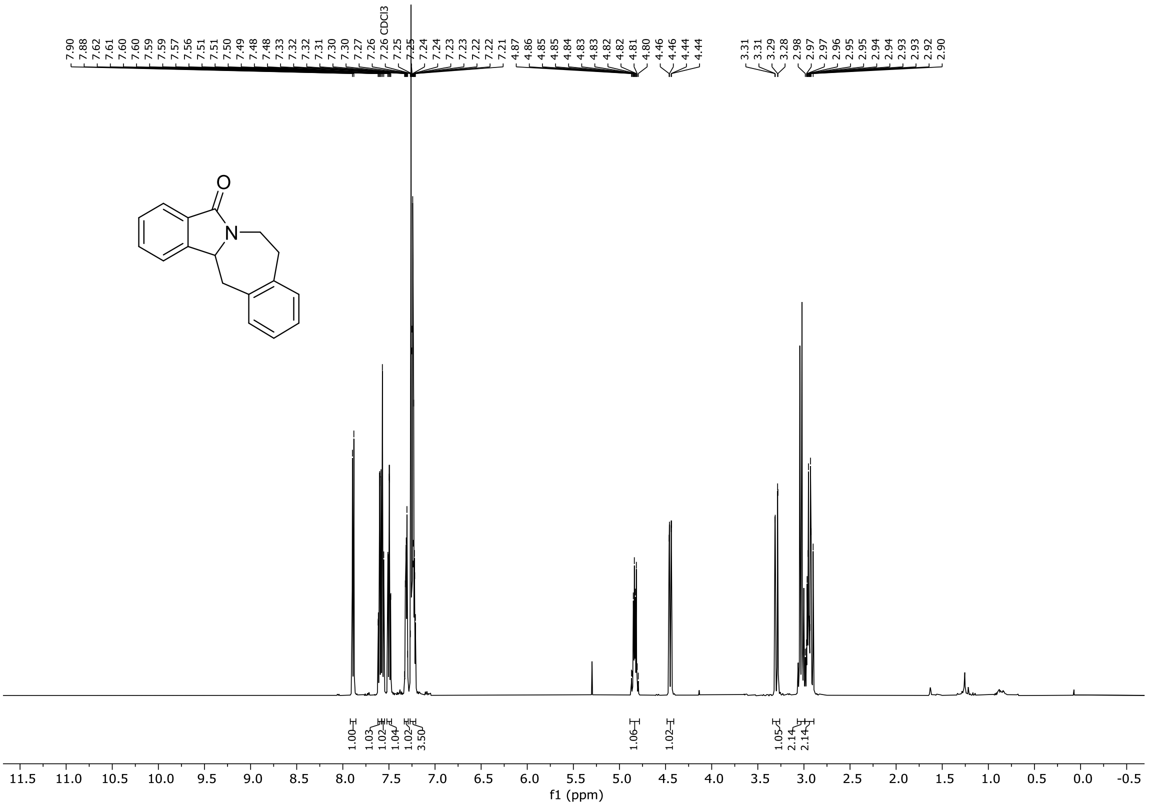

^{13}C NMR (126 MHz, $CDCl_3$):

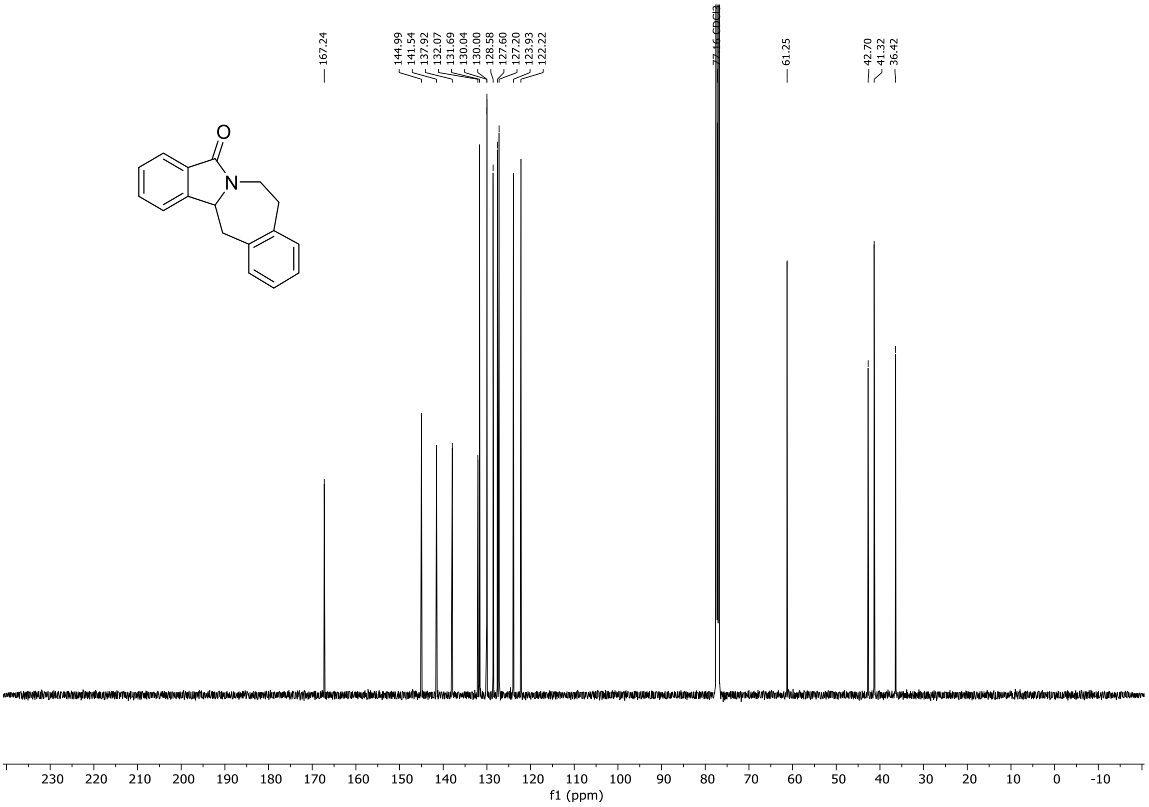

*8.4.20. 9,10-Dimethoxy-5,6,12b,13-tetrahydro-8H-[1,3]dioxolo[4'',5'':4',5']benzo[1',2':4,5]azepino[2,1-a]isoindol-8-one (Lennoxamine, **1t**)*

^{1}H NMR (500 MHz, $CDCl_3$):

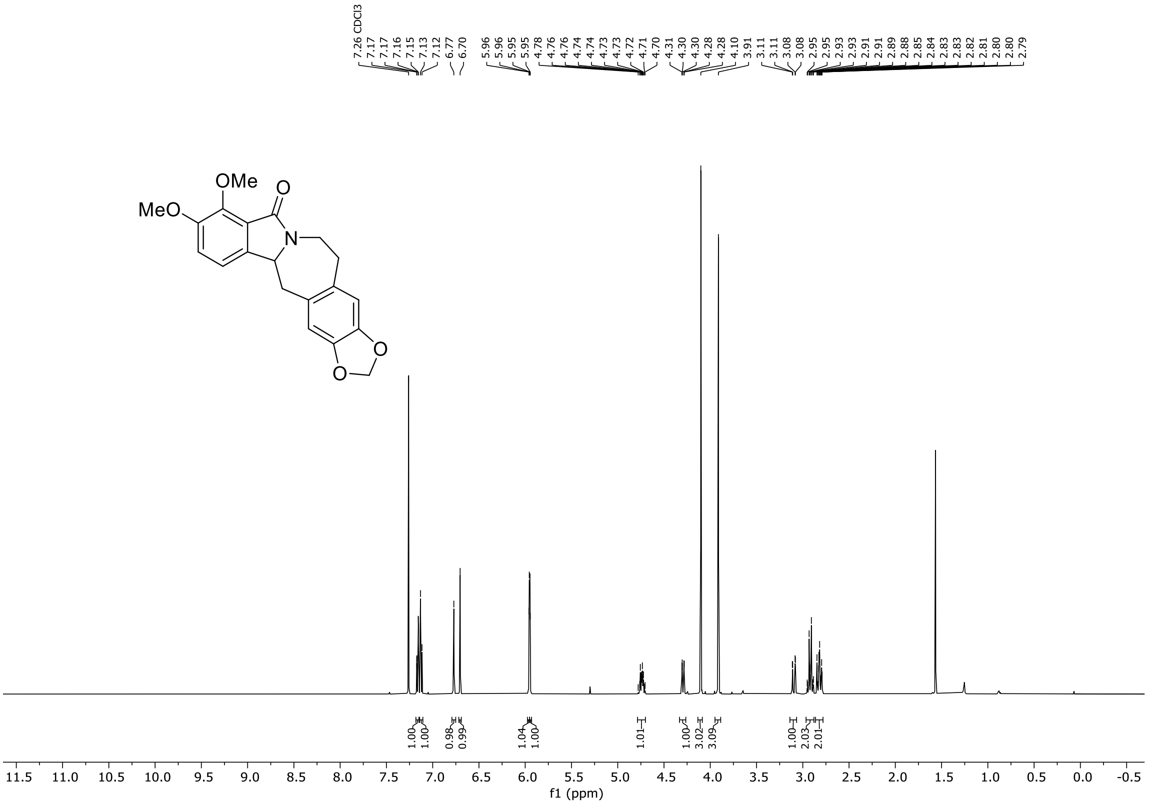

^{13}C NMR (126 MHz, $CDCl_3$):

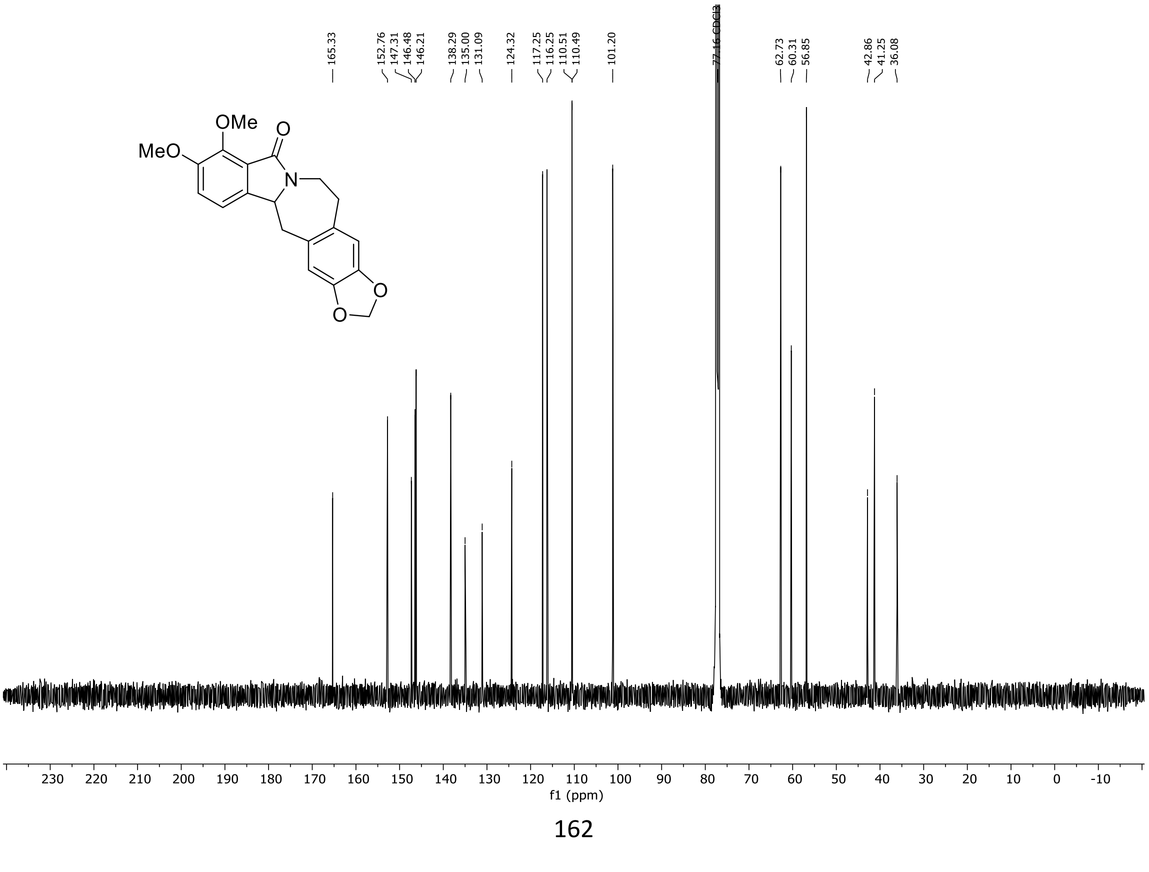

*8.4.21. 3-Methyl-1,2,3,9b-tetrahydro-5H-pyrrolo[2,1-a]isoindol-5-one (**1u**)*

^{1}H NMR (500 MHz, $CDCl_3$):

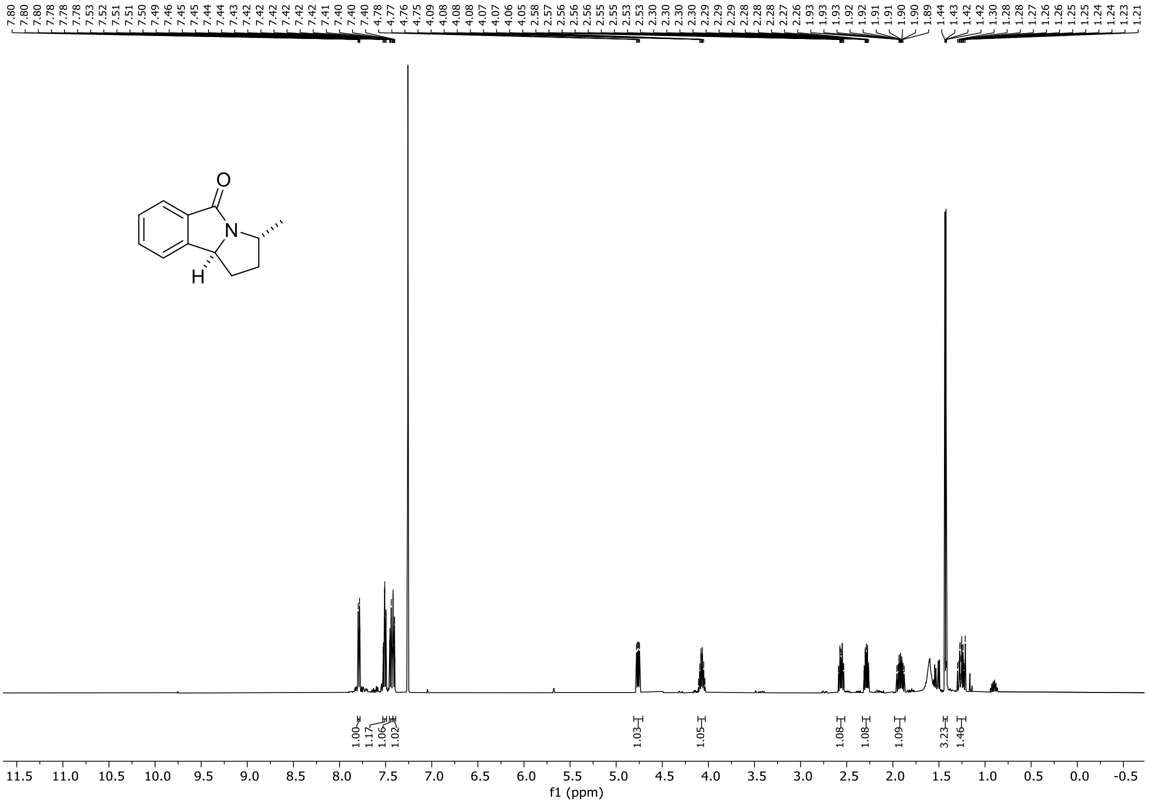

^{13}C NMR (126 MHz, $CDCl_3$):

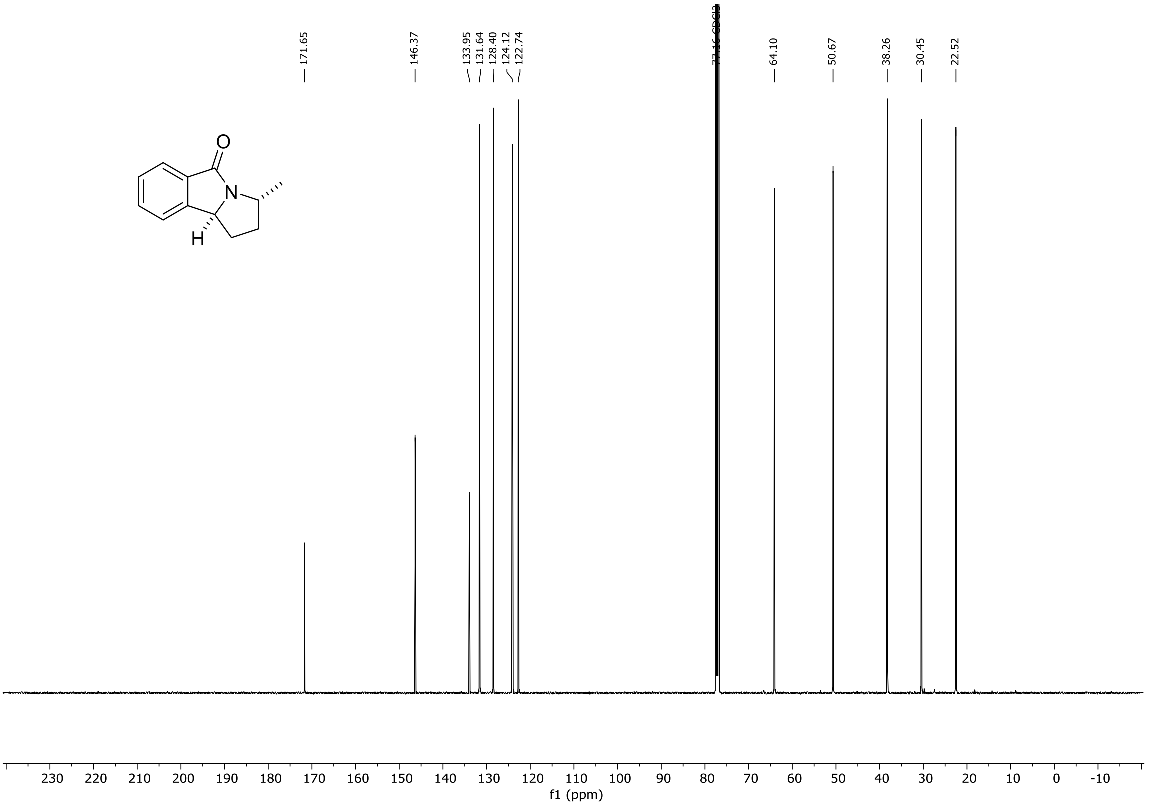

*8.4.22. 3-((tert-Butyldimethylsilyl)oxy)-1,3,4,10b-tetrahydropyrido[2,1-a]isoindol-6(2H)-one (**1v**)*

^{1}H NMR (500 MHz, CDCl$_3$):

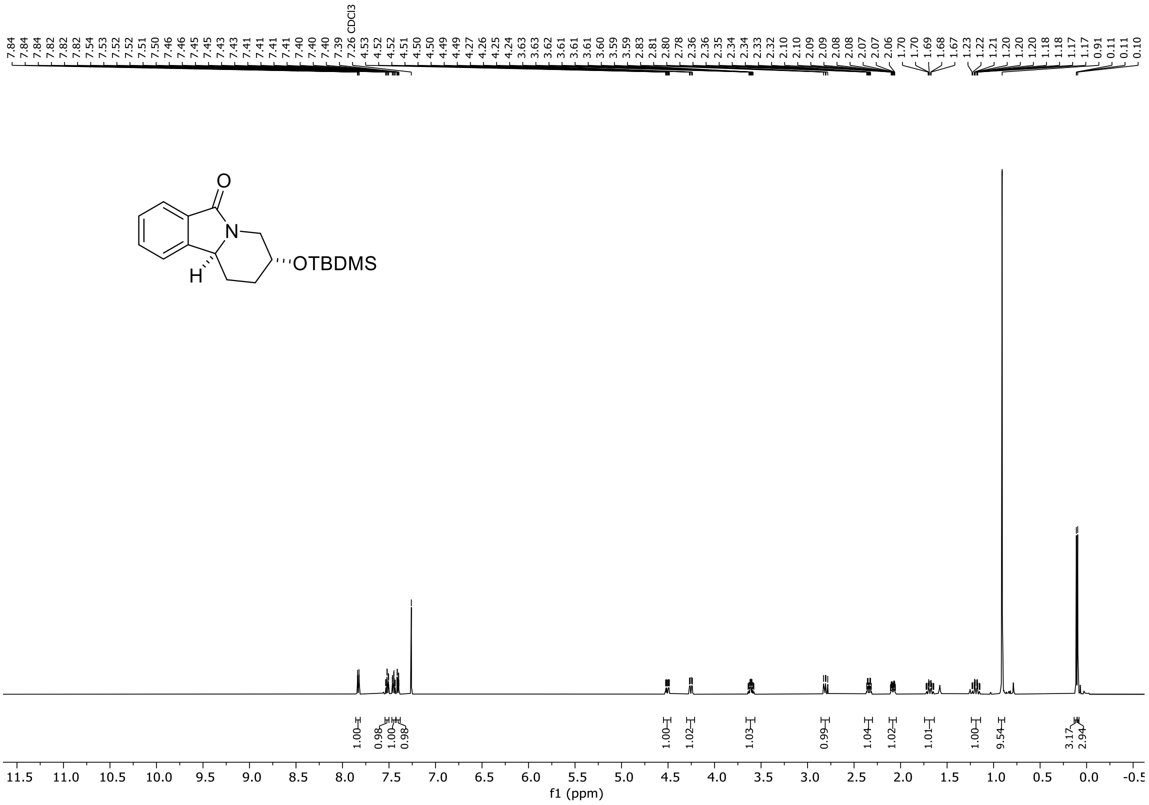

^{13}C NMR (126 MHz, CDCl$_3$):

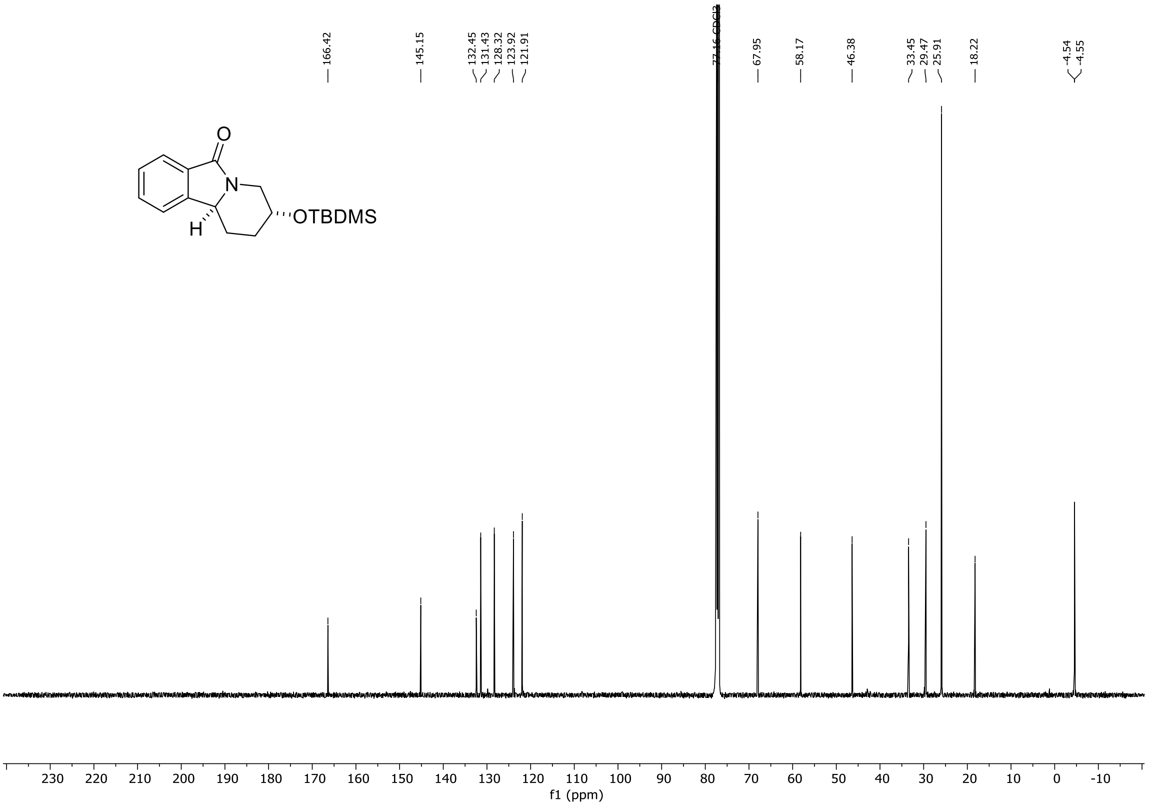

*8.4.23. 3-((tert-Butyldimethylsilyl)oxy)-1,3,4,10b-tetrahydropyrido[2,1-a]isoindol-6(2H)-one (**1w**)*

^{1}H NMR (500 MHz, $CDCl_3$):

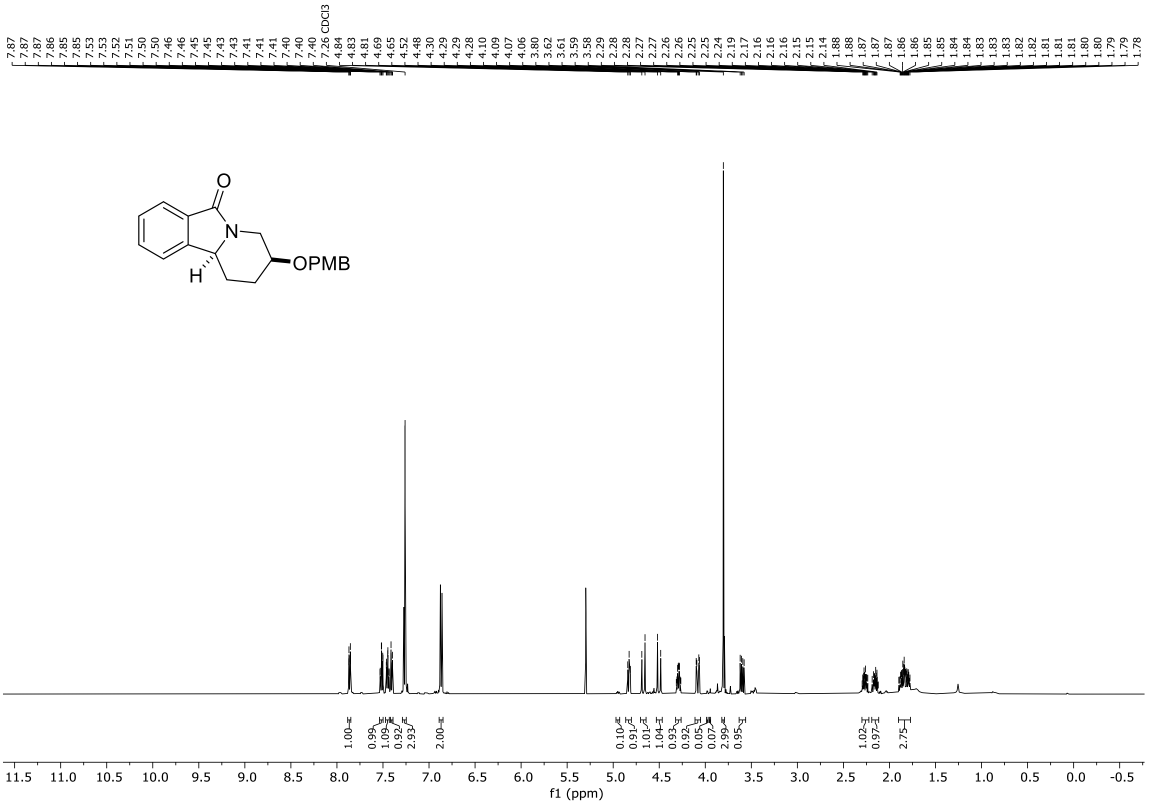

^{13}C NMR (126 MHz, $CDCl_3$):

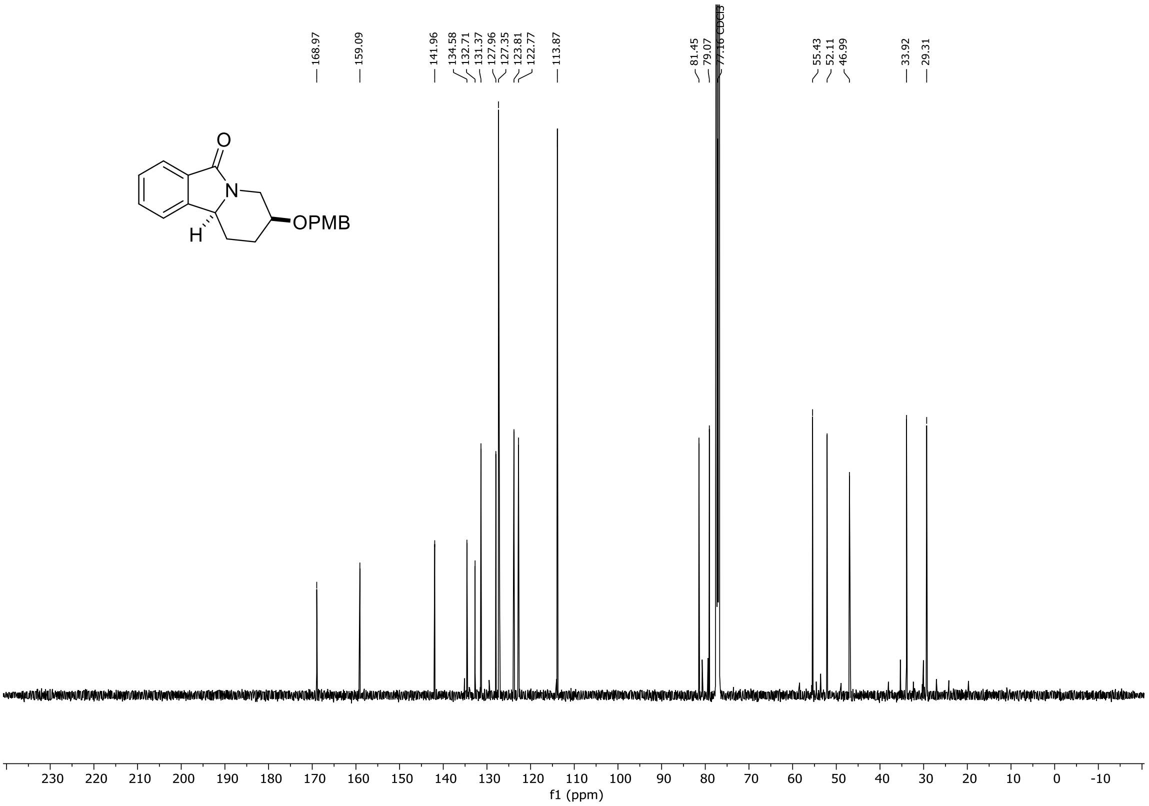

*8.4.24. 2-Methyl-1,3,4,10b-tetrahydropyrido[2,1-a]isoindol-6(2H)-one (**1x**)*

^{1}H NMR (500 MHz, $CDCl_3$):

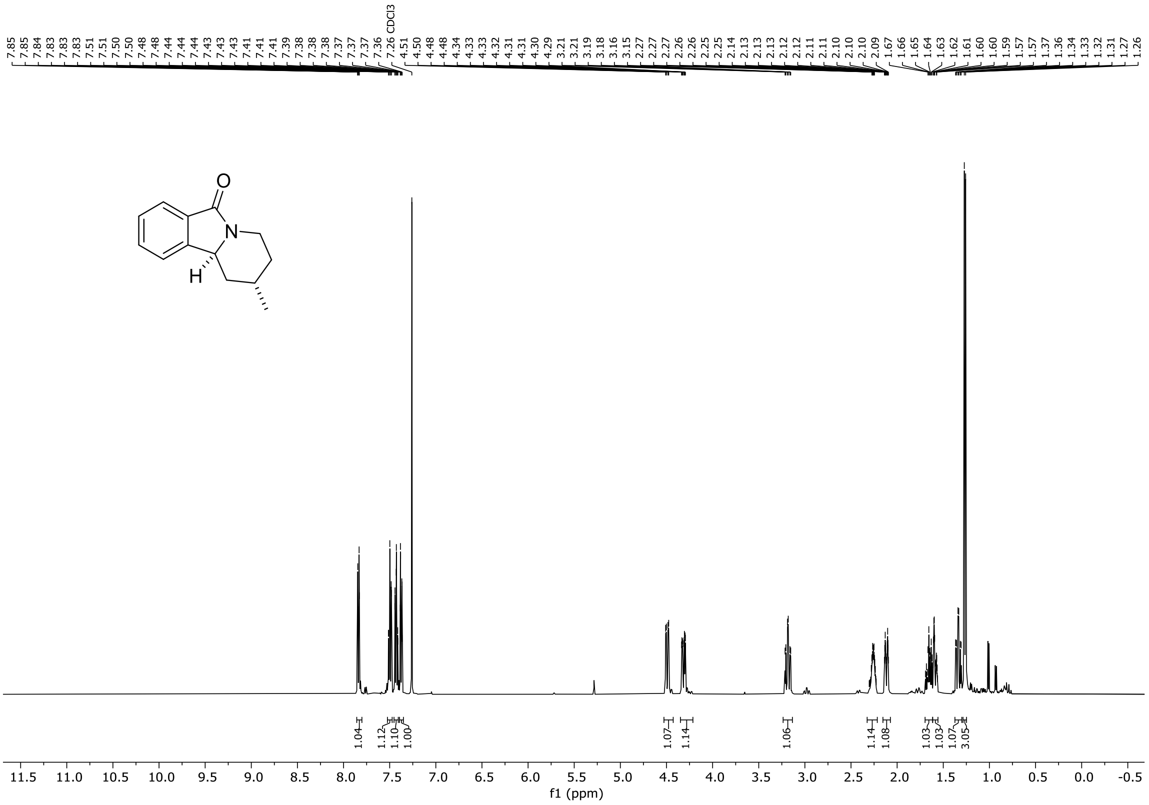

^{13}C NMR (126 MHz, $CDCl_3$):

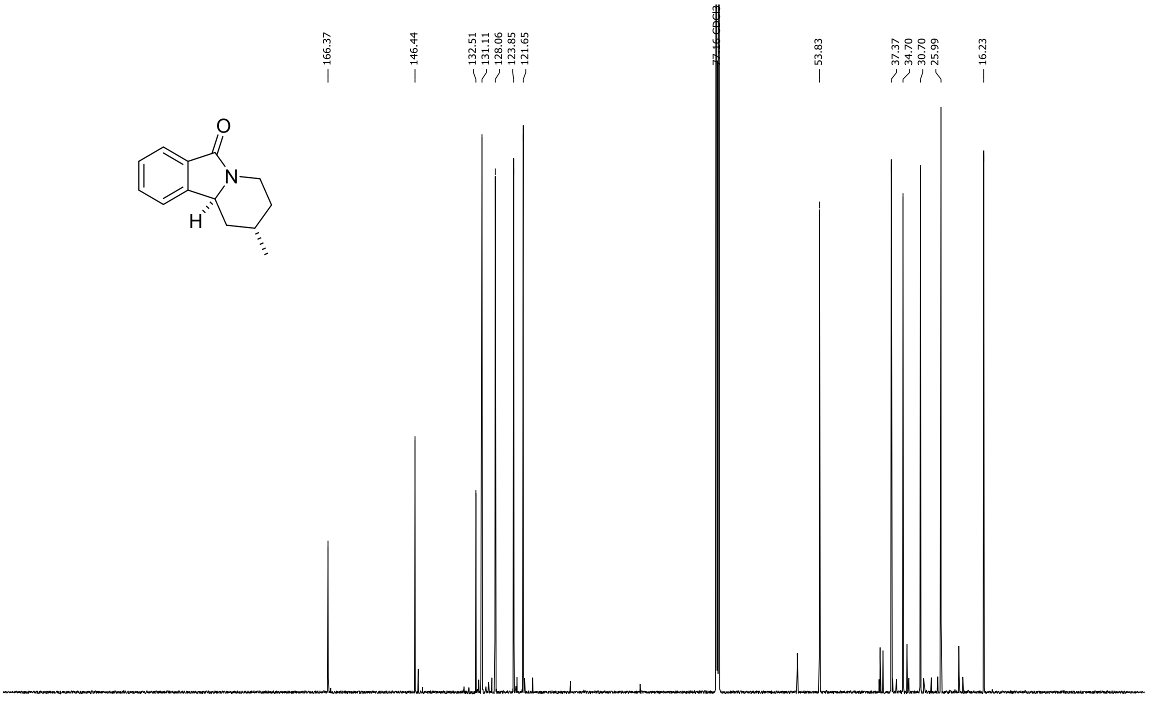

8.5. Deuteration Experiments

*8.5.1. 2-(2-(1,3-dioxolan-2-yl)ethyl)isoindoline-1,3-dione (**SI-15**)*

^{1}H NMR (500 MHz, CDCl$_3$):

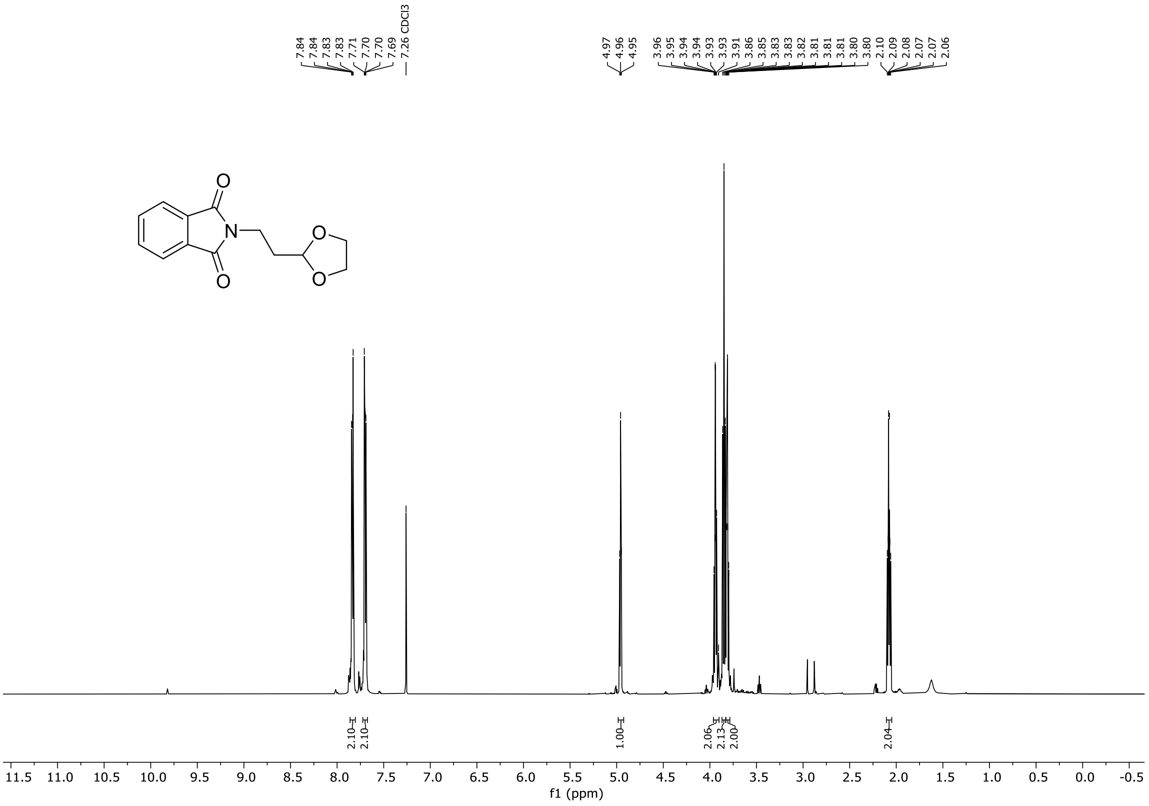

^{13}C NMR (126 MHz, $CDCl_3$):

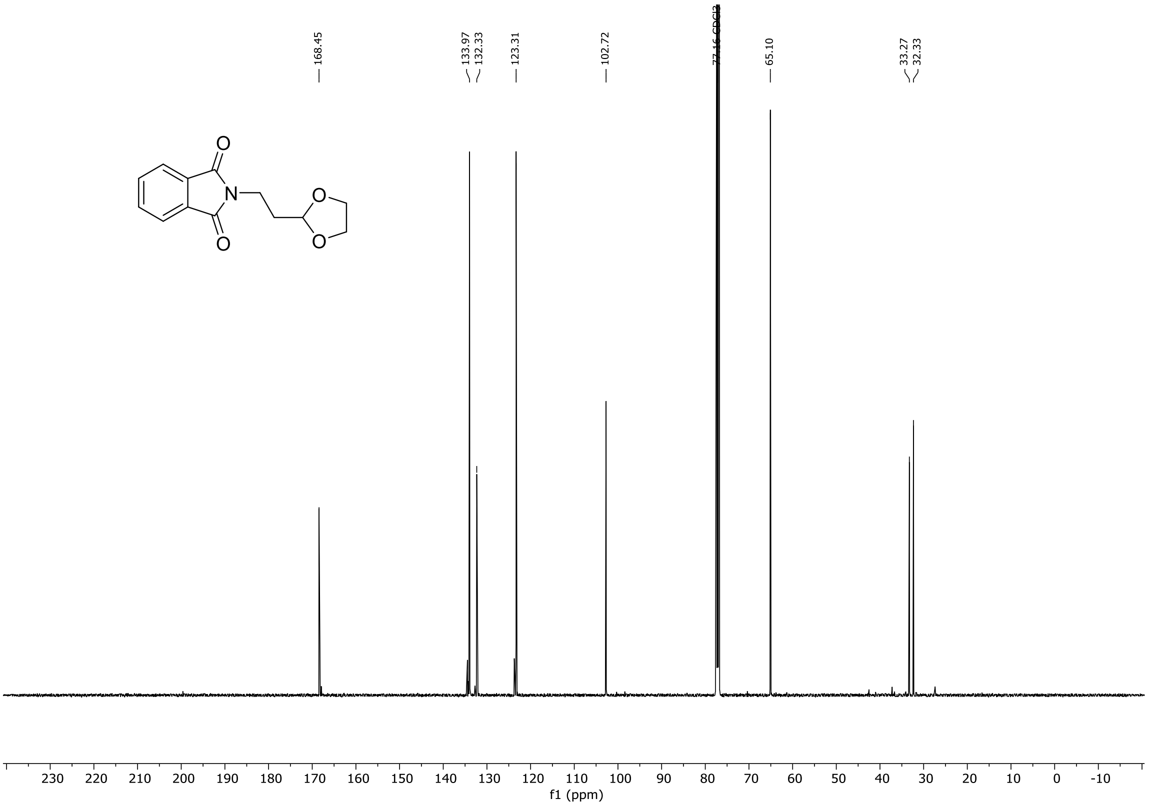

*8.5.2. N'-(3-(1-Oxoisoindolin-2-yl-3,3-d₂)propylidene)-4-methylbenzenesulfonohydrazide (**2a**-d₂)*

^{1}H NMR (500 MHz, DMSO-d_6):

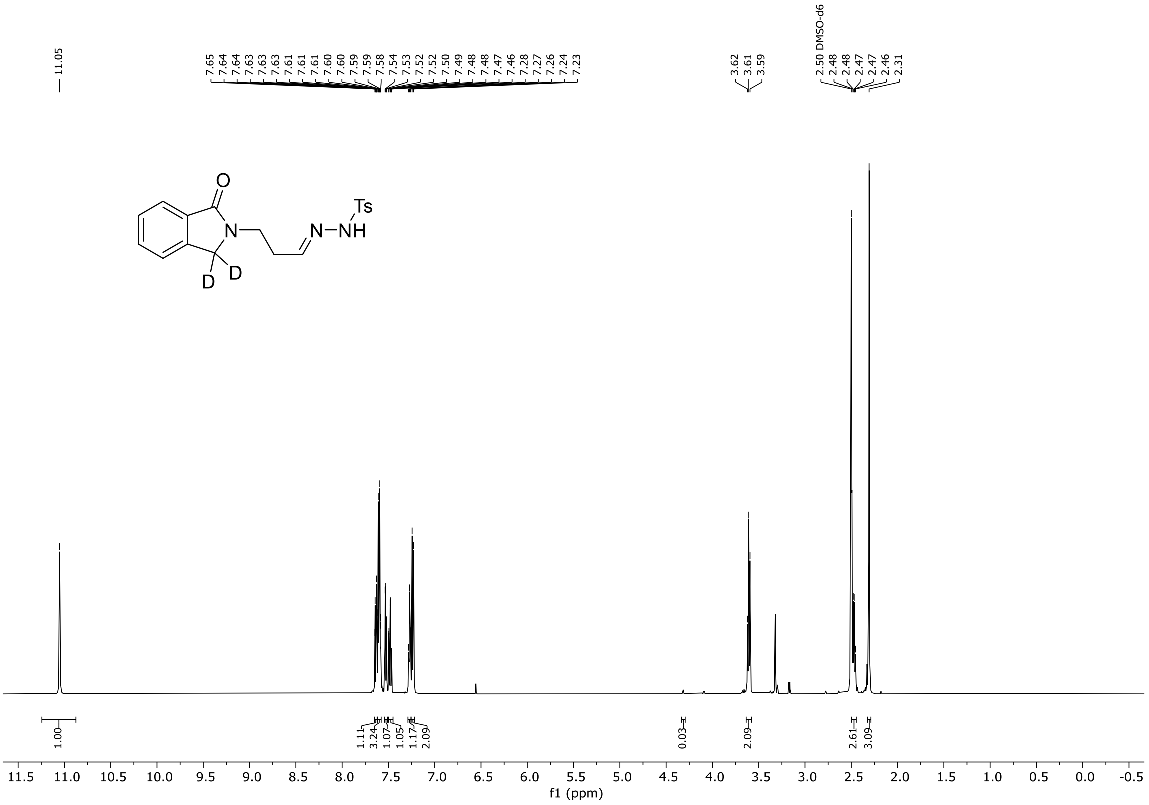

^{13}C NMR (126 MHz, DMSO-d_6):

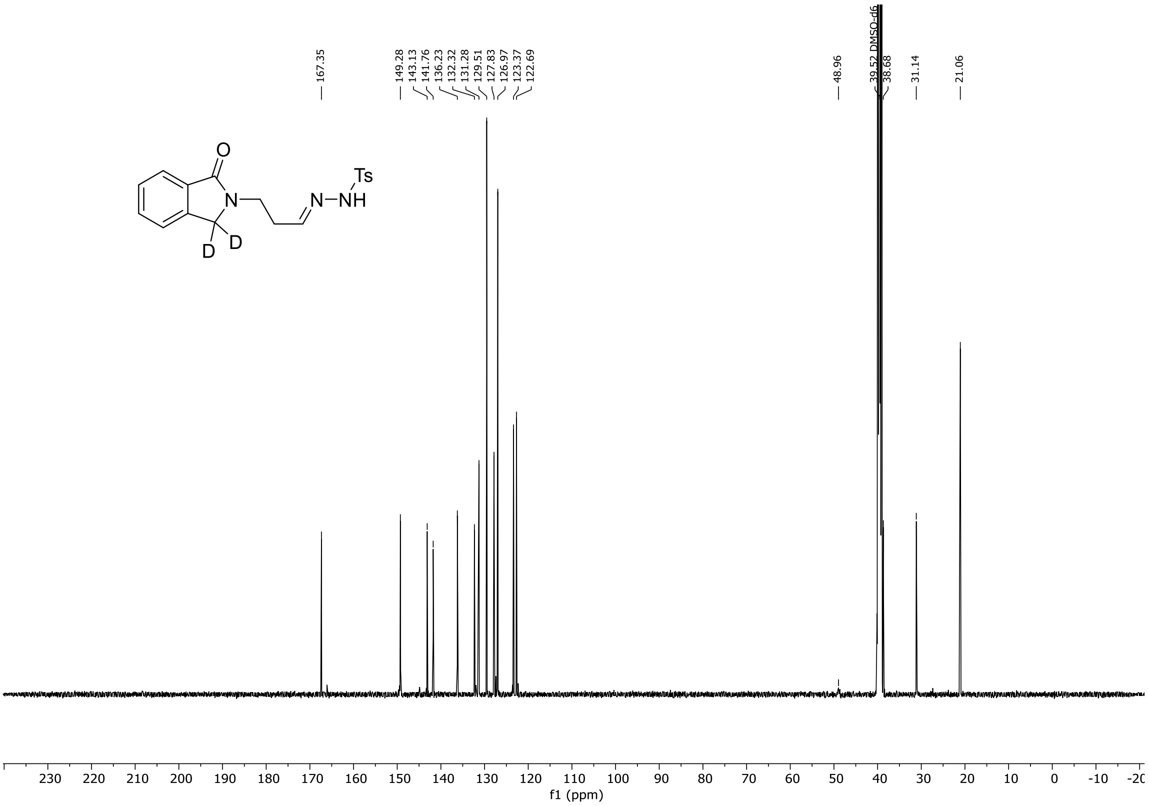

*8.5.3. 2-(3-Hydroxypropyl)isoindolin-1-one-3-d₁ (**SI-1a**-d₁)*

^{1}H NMR (500 MHz, $CDCl_3$):

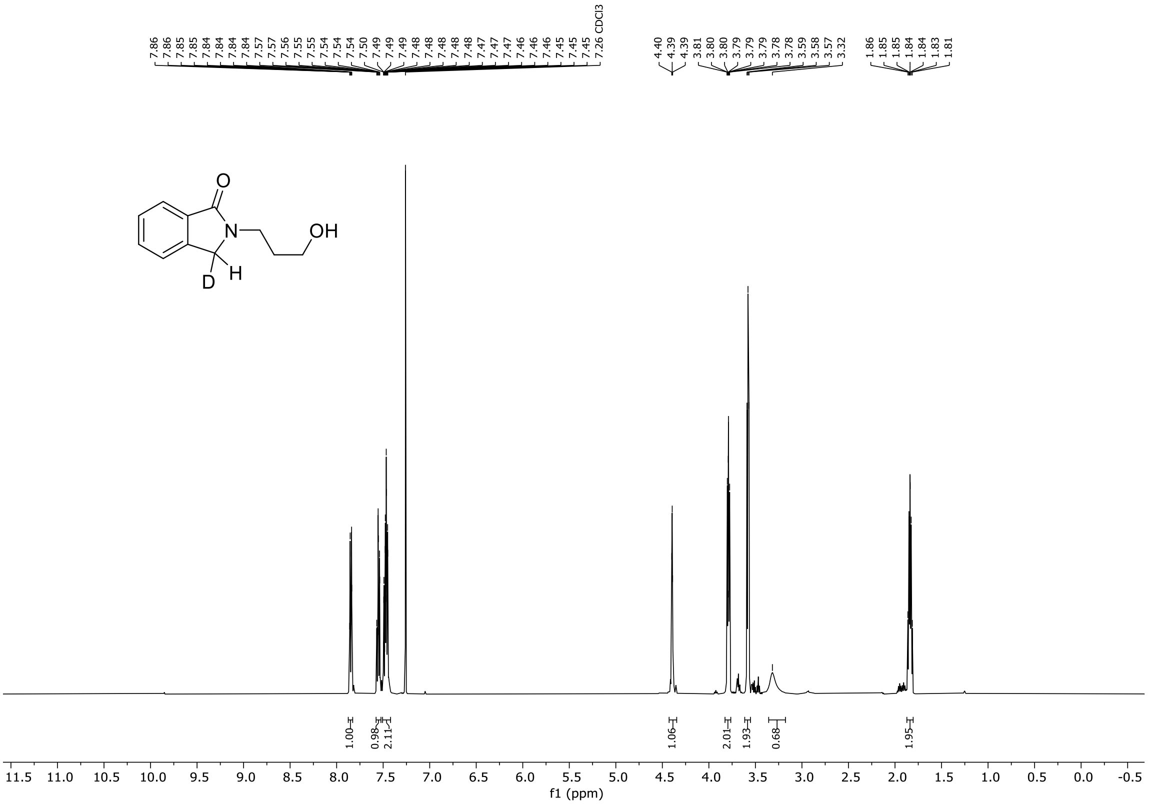

^{13}C NMR (126 MHz, $CDCl_3$):

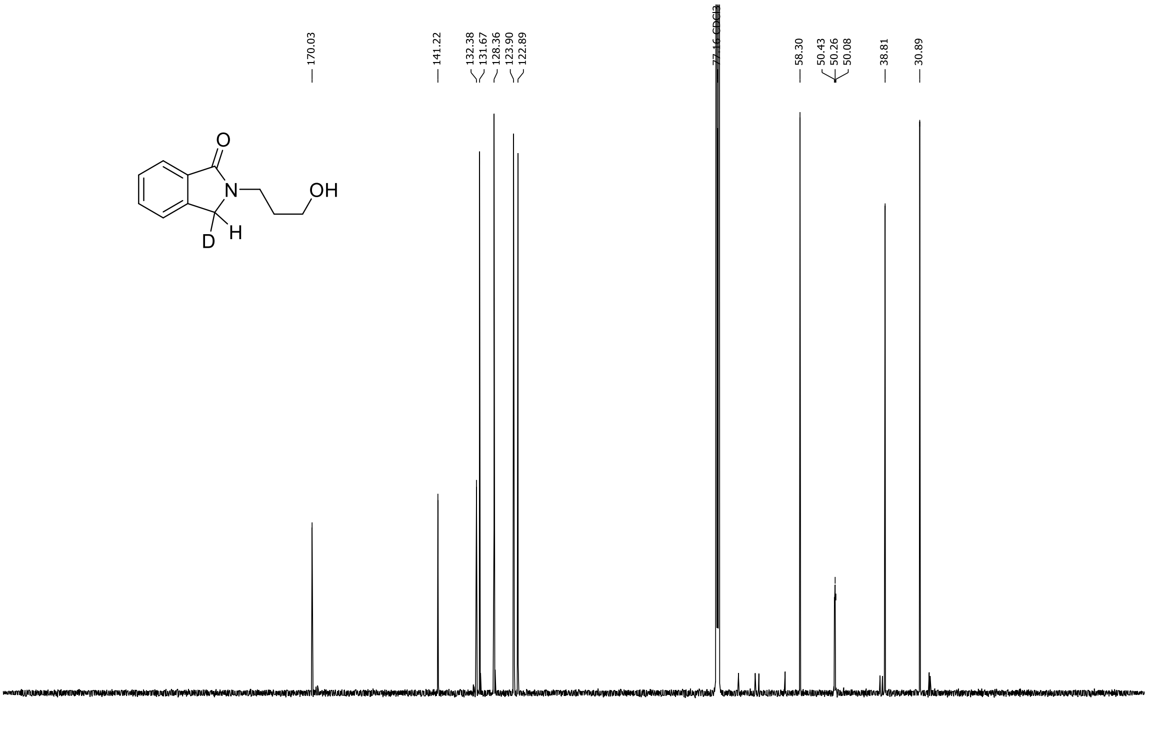

*8.5.4. N'-(3-(1-oxoisoindolin-2-yl-3-d_1)propylidene)-4-methylbenzenesulfonohydrazide (**2a**-d_1)*

^{1}H NMR (500 MHz, DMSO-d_6):

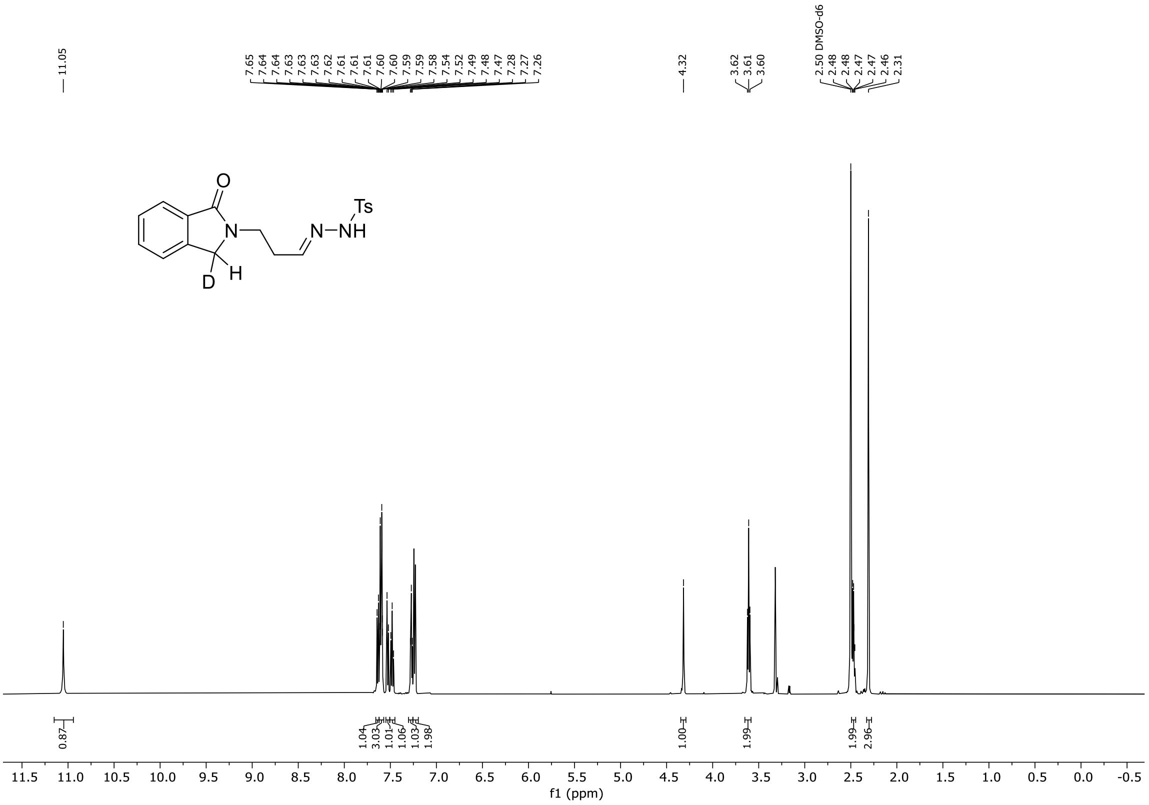

^{13}C NMR (126 MHz, DMSO-d_6):

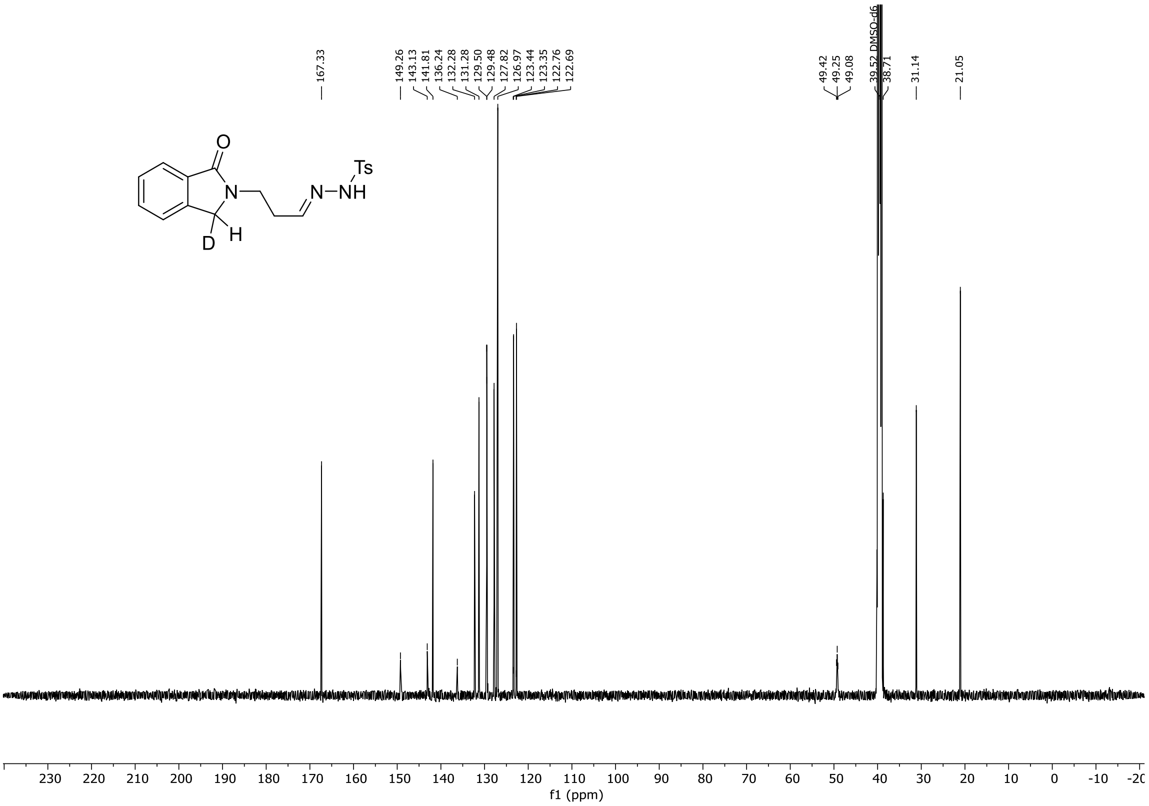

*8.5.5. 1,2,3,9b-Tetrahydro-5H-pyrrolo[2,1-a]isoindol-5-one-d₂ (**1a**-d₂)*

^{1}H NMR (500 MHz, $CDCl_3$):

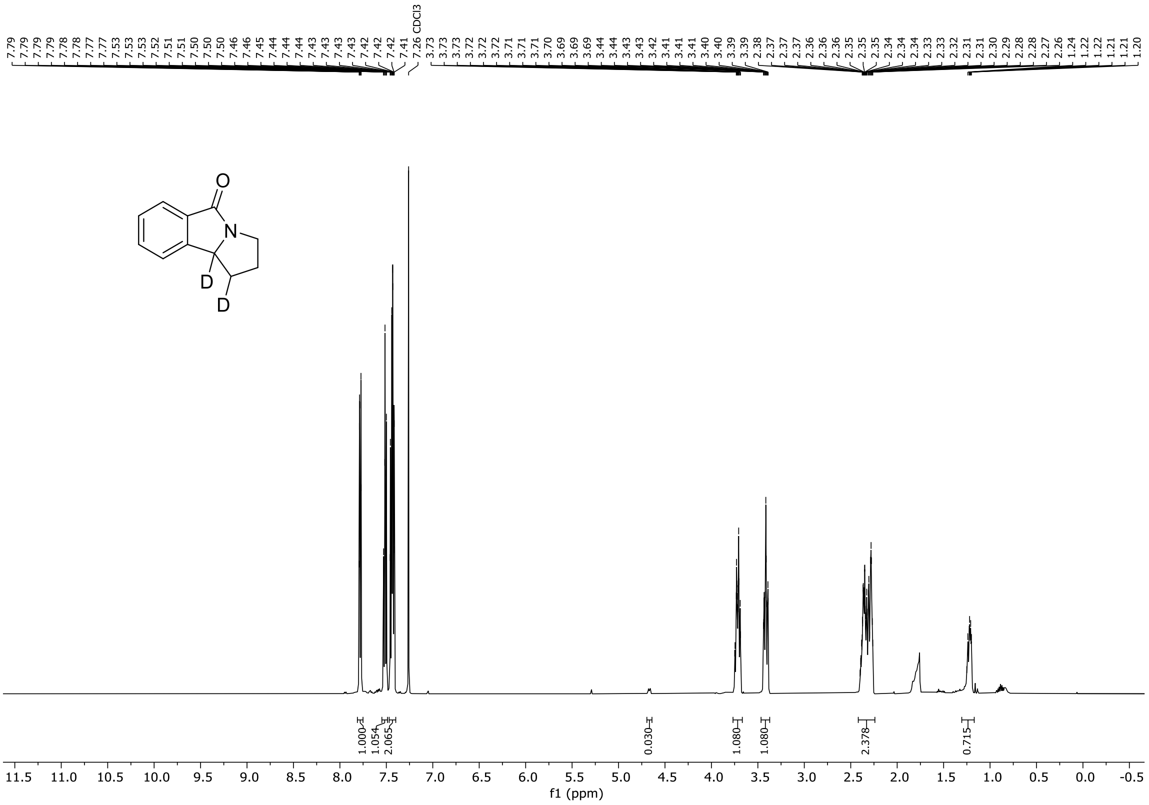

^{13}C NMR (126 MHz, $CDCl_3$):

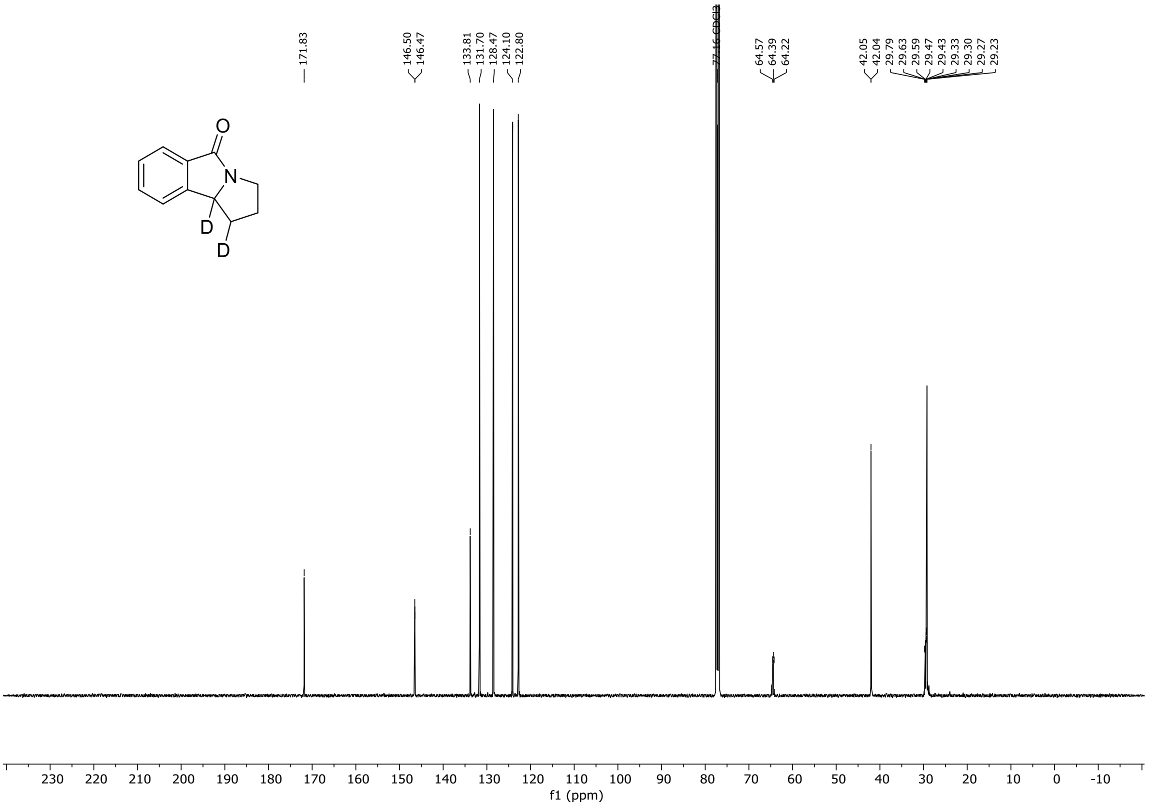

8.5.6. *1,2,3,9b-Tetrahydro-5H-pyrrolo[2,1-a]isoindol-5-one-d₂* (***1a**-d₁*)

^{1}H NMR (500 MHz, $CDCl_3$):

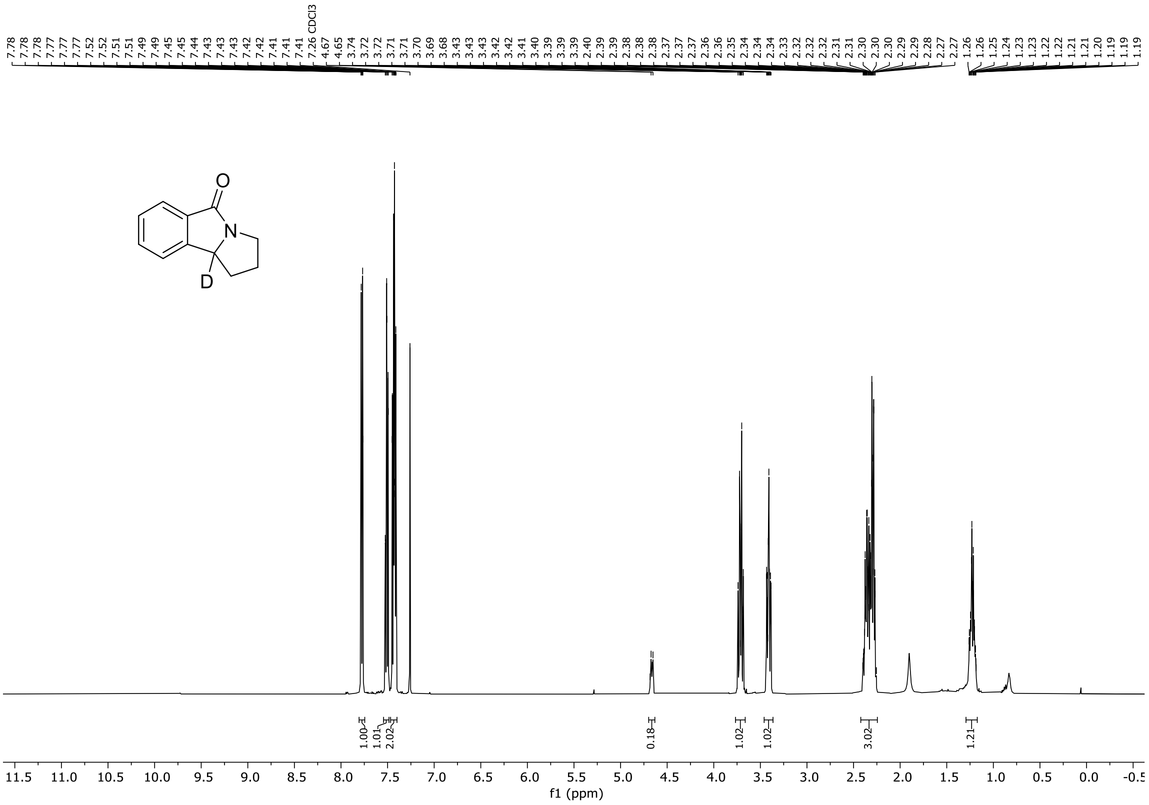

^{13}C NMR (126 MHz, $CDCl_3$):

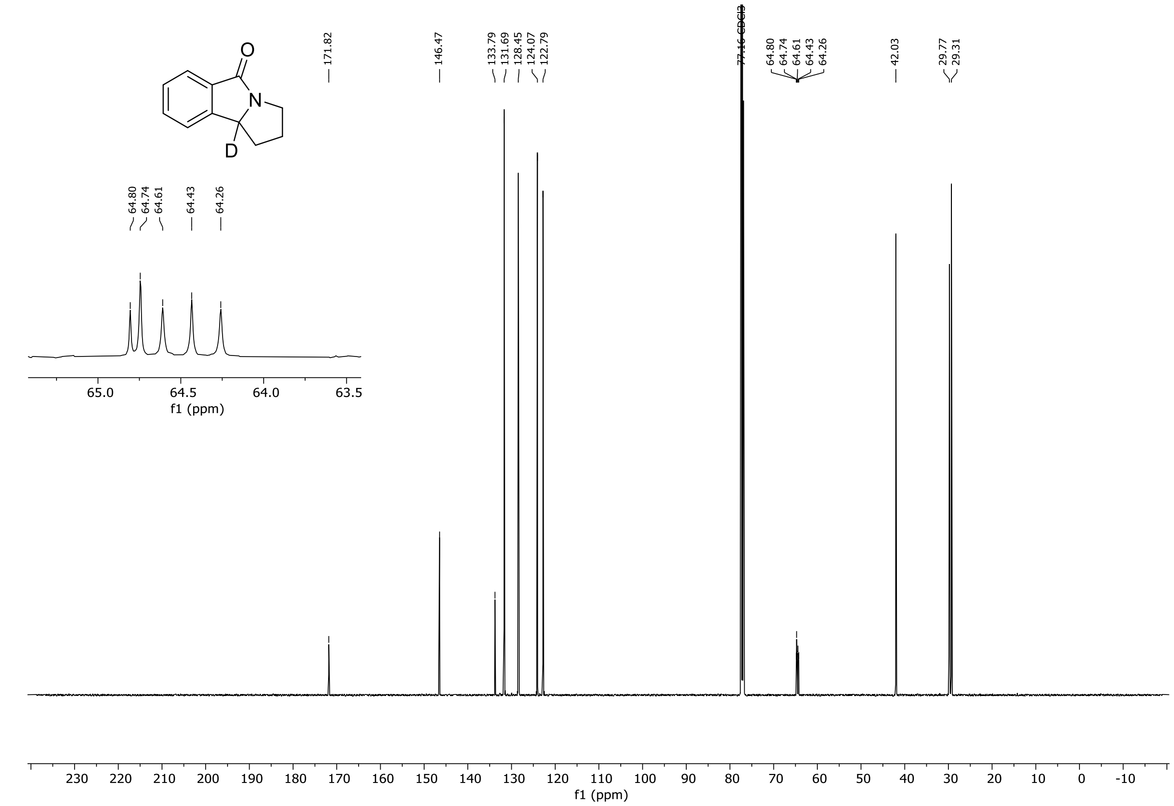

*8.5.7. 2-(2-(1,3-dioxolan-2-yl)ethyl)isoindoline-1,3-dione-d₂ (**3**-d₂)*

^{1}H NMR (500 MHz, $CDCl_3$):

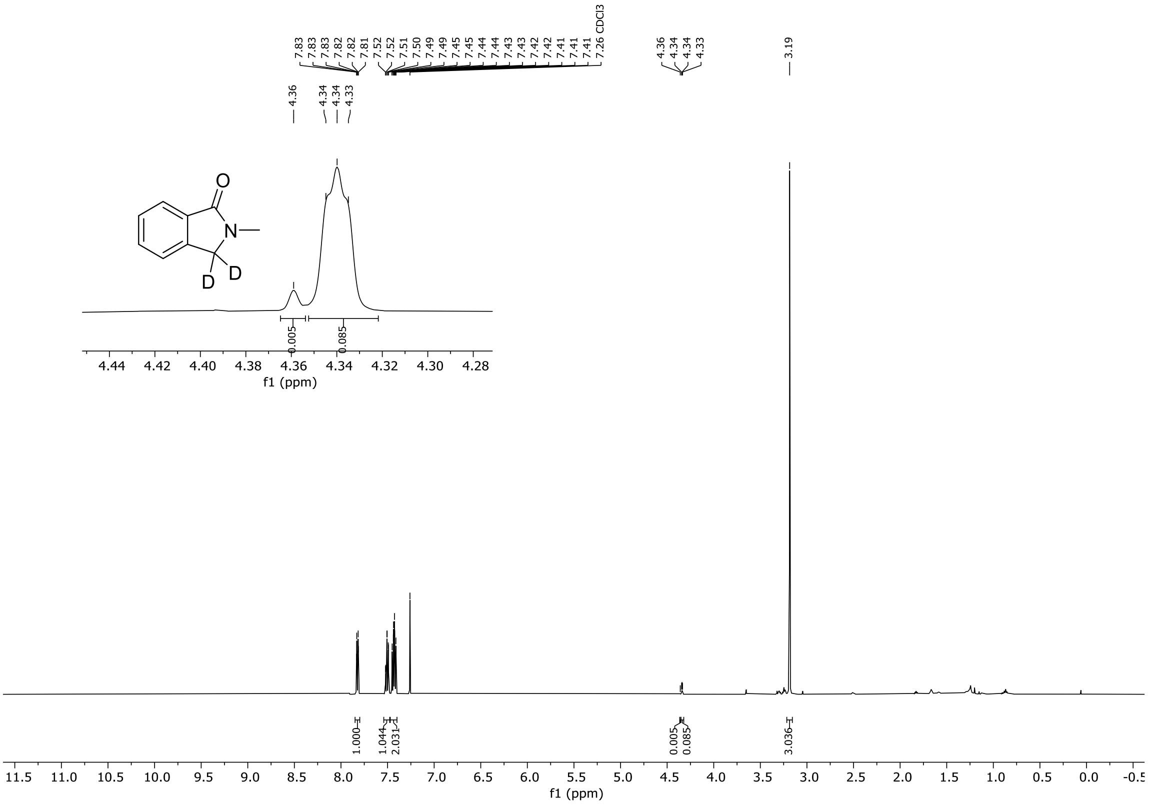

9. References

[1] Y. Chen, L. Fu, Z. Liu, *Chem. Commun.* **2015**, *51*, 16637–16640.

[2] M. H. Norman, D. J. Minick, G. C. Rigdon, *J. Med. Chem.* **1996**, *39*, 149–157.

[3] M. Ordóñez, G. Tibhe, A. Zamudio-Medina, J. Viveros-Ceballos, *Synthesis* **2012**, *2012*, 569–574.

[4] P. C. Patil, F. A. Luzzio, J. M. Ronnebaum, *Tetrahedron Lett.* **2017**, *58*, 3730–3733.

[5] A. Vermote, G. Brackman, M. D. P. Risseeuw, T. Coenye, S. van Calenbergh, *Bioorg. Med. Chem.* **2016**, *24*, 4563–4575.

[6] K. Omura, D. Swern, *Tetrahedron* **1978**, *34*, 1651–1660.

[7] J. I. Levin, S. M. Weinreb, *J. Org. Chem.* **1984**, *49*, 4325–4332.

[8] B.-H. Zhang, L.-S. Lei, S.-Z. Liu, X.-Q. Mou, W.-T. Liu, S.-H. Wang, J. Wang, W. Bao, K. Zhang, *Chem. Commun.* **2017**, *53*, 8545–8548.

[9] F. Ulgheri, P. Spanu, F. Deligia, G. Loriga, M. P. Fuggetta, I. de Haan, A. Chandgudge, M. Groves, A. Domling, *Eur. J. Med. Chem.* **2022**, *229*, 114002.

[10] A. Sravanth Kumar, K. Praneeth, P. Srihari, J. S. Yadav, *Tetrahedron Lett.* **2017**, *58*, 509–511.

[11] J.-Z. Wu, Z. Wang, C. Qiao, *Tetrahedron Lett.* **2012**, *53*, 1153–1155.

[12] X. Jiang, B. Liu, S. Lebreton, J. K. de Brabander, *J. Am. Chem. Soc.* **2007**, *129*, 6386–6387.

[13] X. Yan, J. Zheng, W.-D. Z. Li, *Tetrahedron Lett.* **2023**, *132*, 154826.

[14] T. D. D'Arcy, M. R. J. Elsegood, B. R. Buckley, *Angew. Chem. Int. Ed.* **2022**, *61*, e202205278.

[15] A. Couture, E. Deniau, D. Ionescu, P. Grandclaudon, *Tetrahedron Letters* **1998**, *39*, 2319–2320.

[16] R. C. Spitale, R. A. Flynn, Q. C. Zhang, P. Crisalli, B. Lee, J.-W. Jung, H. Y. Kuchelmeister, P. J. Batista, E. A. Torre, E. T. Kool et al., *Nature* **2015**, *519*, 486–490.

[17] M. Lankelma, A. M. Olivares, B. de Bruin, *Chemistry* **2019**, *25*, 5658–5663.

[18] C. Bressy, C. Menant, O. Piva, *Synlett* **2005**, 577–582.

[19] A. Garay-Talero, T. A. C. Goulart, R. D. C. Gallo, R. d. C. Pinheiro, C. Hoyos-Orozco, I. D. Jurberg, D. Gamba-Sánchez, *Org. Lett.* **2023**, *25*, 7940–7945.

[20] J. Sim, B. Ryou, M. Choi, C. Lee, C.-M. Park, *Org. Lett.* **2022**, *24*, 4264–4269.

[21] J. D. St Denis, A. Zajdlik, J. Tan, P. Trinchera, C. F. Lee, Z. He, S. Adachi, A. K. Yudin, *J. Am. Chem. Soc.* **2014**, *136*, 17669–17673.

[22] V. I. Vinogradova, M. S. Yunusov, A. V. Kuchin, G. A. Tolstikov, R. T. Sagandykov, K. A. Khalmuratov, A. Alimov, *Chem. Nat. Compd.* **1990**, *26*, 54–59.

[23] S. Pradhan, R. V. Sankar, C. Gunanathan, *J. Org. Chem.* **2022**, *87*, 12386–12396.

[24] P. Sahakitpichan, S. Ruchirawat, *Tetrahedron* **2004**, *60*, 4169–4172.

[25] W. Zhu, S. Tong, J. Zhu, M.-X. Wang, *J. Org. Chem.* **2019**, *84*, 2870–2878.

[26] *APEX4 Suite of Crystallographic Software, Version 2021-10.0,* Bruker AXS Inc., Madison, Wisconsin, USA, **2021**.

[27] Bruker, *SAINT*, *V8.40B*, Bruker AXS Inc., Madison, Wisconsin, USA.

[28] L. Krause, R. Herbst-Irmer, G. M. Sheldrick, D. Stalke, *J. Appl. Cryst.* **2015**, *48*, 3–10, doi:10.1107/S1600576714022985.

[29] G. M. Sheldrick, *Acta Cryst.* **2015**, *A71*, 3–8, doi:10.1107/S2053273314026370.

[30] G. M. Sheldrick, *Acta Cryst.* **2015**, *C71*, 3–8, doi:10.1107/S2053229614024218.

[31] C. B. Huebschle, G. M. Sheldrick, B. Dittrich, *J. Appl. Cryst.* **2011**, *44*, 1281–1284.

[32] Ed. E. Prince, *International Tables for Crystallography Volume C, Mathematical, Physical and Chemical Tables*, International Union of Crystallography, Chester, England, **2006**, 500–502; 219–222; 193–199.

[33] C. R. Groom, I. J. Bruno, M. P. Lightfoot, S. C. Ward, *Acta Cryst.* **2016**, *B72*, 171–179.

[34] D. Kratzert, *FinalCif*, *V139*, https://dkratzert.de/finalcif.html.
